# Enantioselective palladium(0)-catalyzed intramolecular cyclopropane functionalization: access to dihydroquinolones, dihydroisoquinolones and the BMS-791325 ring system†
†Electronic supplementary information (ESI) available: Experimental procedures and characterization of all new compounds. CCDC 1401582. For ESI and crystallographic data in CIF or other electronic format see DOI: 10.1039/c5sc01909e



**DOI:** 10.1039/c5sc01909e

**Published:** 2015-06-17

**Authors:** J. Pedroni, T. Saget, P. A. Donets, N. Cramer

**Affiliations:** a Laboratory of Asymmetric Catalysis and Synthesis , Institute of Chemical Sciences and Engineering , Ecole Polytechnique Fédérale de Lausanne , EPFL SB ISIC LCSA, BCH 4305 , CH-1015 Lausanne , Switzerland . Email: nicolai.cramer@epfl.ch

## Abstract

Taddol-based phosphoramidite ligands enable enantioselective palladium(0)-catalyzed C–H arylation of cyclopropanes. The cyclized products are obtained in high yields and enantioselectivities. The reported method provides efficient access to a broad range of synthetically attractive cyclopropyl containing dihydroquinolones and dihydroisoquinolones as well as allows for an efficient enantioselective construction of the 7-membered ring of the cyclopropyl indolobenzazepine core of BMS-791325.

## Abstract 2

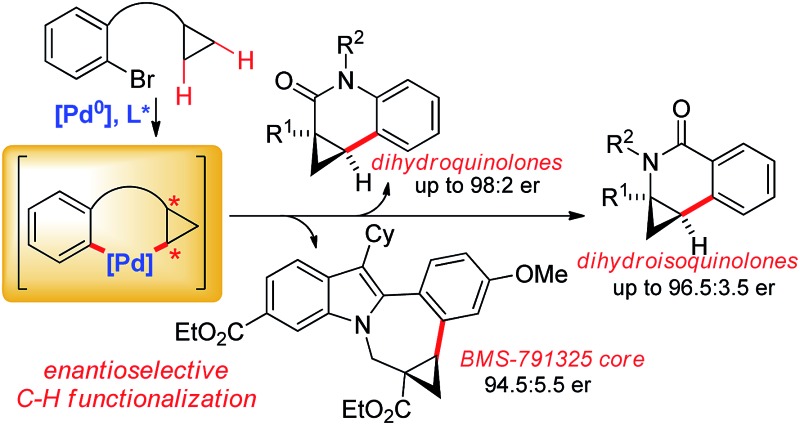
Enantioselective palladium(0)-catalyzed C–H arylations of cyclopropanes provide efficient access to dihydroquinolones, dihydroisoquinolones and the BMS-791325 indolobenzazepine core.

## Introduction

**Fig. 1:**
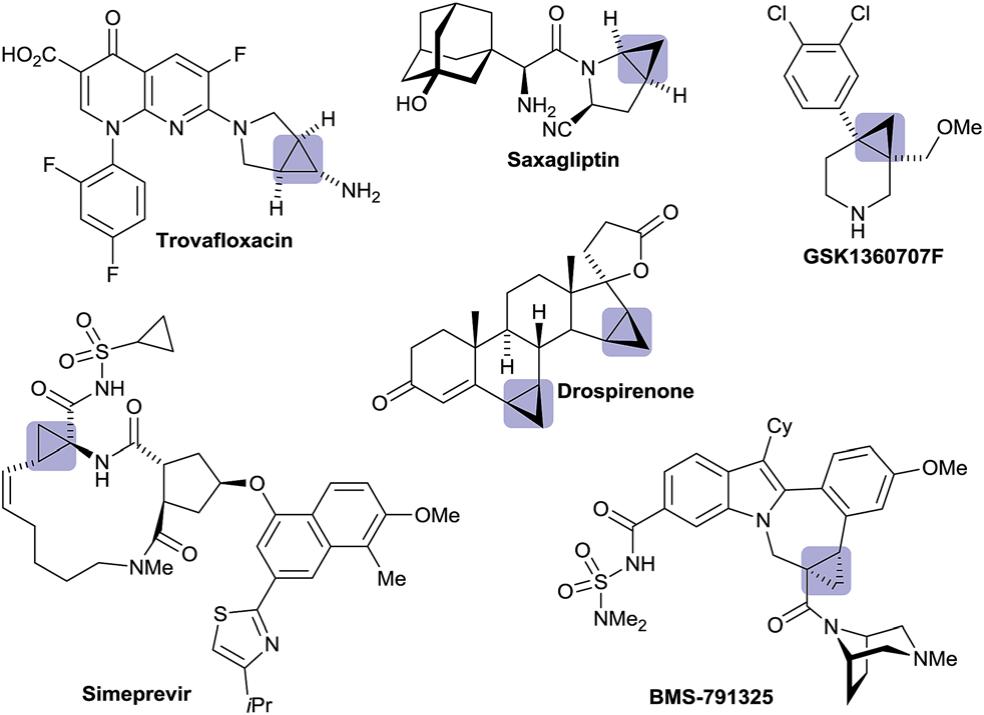
Marketed drugs and development candidates having an annulated cyclopropyl ring system.

Besides its occurrence in natural products,^1^ cyclopropane is a common structural motif in currently marketed drug or development candidates, often in conjunction with a nitrogen atom in close vicinity (Fig. 1).^2^ This prevalence can be attributed to its strategic use aiming to increase metabolic stability without a large increase in molecular weight or installation of fluorine atoms. In its own right, cyclopropane is an attractive scaffold providing opportunities to arrange pendant groups in a rigid and specific three-dimensional orientation in space.

**Scheme 1:**
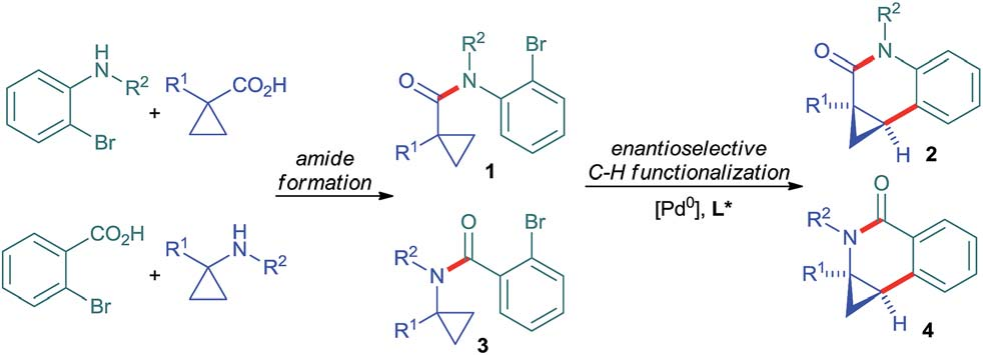
Rapid enantioselective assembly of dihydroquinolones and dihydroisoquinolones by Pd^0^-catalyzed C–H functionalization.

A broad range of methods for the construction of cyclopropane rings have been reported and are frequently used.^3^ However, the direct functionalization of an existing cyclopropane group as a complementary strategy remains underdeveloped. These comprise mainly stoichiometric metalations with Grignard reagent or organo-lithium bases.^4^ Sparteine could be used as chiral modifier for enantioselective deprotonations.^5^ Some transition-metal catalyzed cyclopropane C–H functionalizations have been reported.^6,7^ However, related enantioselective reactions are very scarce.^8,9^ Yu reported directed palladium(ii)-catalyzed processes for enantioselective C–H arylations of cyclopropyl carboxamides^8*b*^ and cyclopropylmethylamines.^8*c*^ We have reported enantioselective C–H functionalization of *N*-cyclopropylmethyl trifluoromethanesulfonamides to access tetrahydroquinolines.^8*a*^ This initial proof of concept prompted us to exploit this reaction principle further with the aim to develop a rapid access to synthetically versatile chiral building blocks (Scheme 1). We have focused on implementing amides as connectors,^7e,g,10^ which would not only significantly simplify the access to the required substrates **1** and **3**, but also largely enhance the utility of the arising products. Moreover, different ring sizes and positions of the nitrogen heteroatom were envisioned. Herein we report an enantioselective Pd^0^-catalyzed C–H arylation strategy enabling the access to chiral cyclopropylquinolones **2**, cyclopropylisoquinolones **4** and also cyclopropylindolobenzazepines.

## Results and discussion

### Enantioselective synthesis of cyclopropane containing dihydroquinolones

**Scheme 2:**
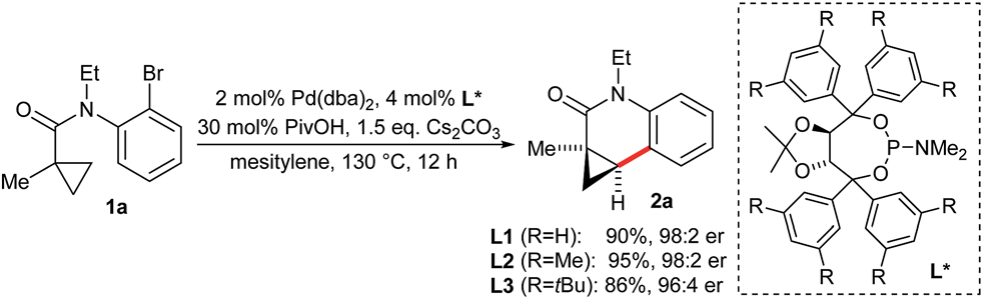
Enantioselective dihydroquinolone synthesis.

Dihydroquinolones are common structures among natural products and bioactive compounds.^11^ Cyclopropyl substituted congeners are not oxidizable to the corresponding aromatic quinolones. A rapid C–H functionalization access to this compound class would represent a significant advantage compared to the *N*-triflyl tetrahydroquinoline product range.^8*a*^ Although the *N*-triflyl group was removable under strongly reducing conditions, it represented a limiting factor. Moreover, the envisioned substrates would be readily accessible *via* an amide bond forming reaction from simple 2-bromoanilines and a cyclopropyl carboxylic acid building block. In addition, the scope would be significantly broadened by having the additional flexibility of a second substituent R^2^ on the amide nitrogen atom. In this case, the interference of R^2^ with the regio- and enantioselectivity of the C–H activation is an unknown variable. Especially the potential of arene containing groups competing with the cyclopropane C–H bonds for the activation has to be considered. Therefore, we aimed also to explore these selectivities and derive some guidelines. To our delight, model substrate **1a** could be selectively activated and cyclized to dihydroquinolone **2a** in 95% yield with a 98 : 2 er employing **L2** and a similar set of conditions as previously developed for the *N*-triflyl tetrahydroquinolines (Scheme 2). This finding underscores the robustness and reliability of the Taddol phosphoramidites for Pd^0^-catalyzed asymmetric activations.^12^

**Scheme 3:**
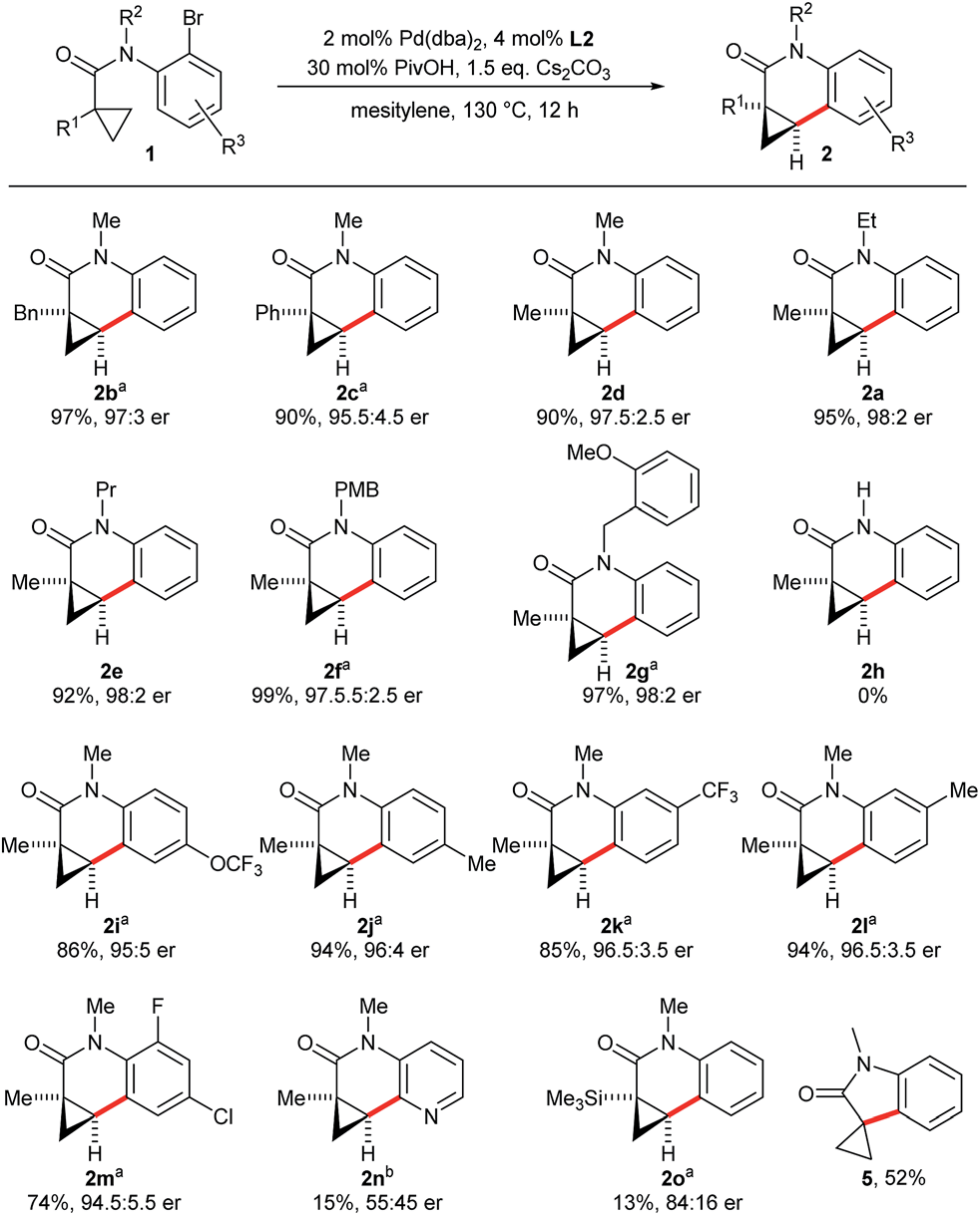
Scope of the enantioselective cyclopropanecarboxamide cyclization. Reaction conditions: **1** (0.10 mmol), Cs_2_CO_3_ (0.15 mmol), Pd(dba)_2_ (2.00 μmol), **L2** (4.00 μmol), PivOH (30 μmol), 0.30 M in mesitylene at 130 °C for 12 h. Yields of isolated products **2**; er's determined by HPLC with a chiral stationary phase. ^a^ [(η^3^-cinnamyl)Pd(Cp)] instead of Pd(dba)_2_. ^b^ With Cs_2_CO_3_ (0.20 mmol), Pd(dba)_2_ (10.0 μmol), **L2** (20.0 μmol), PivOH (50 μmol).

Concerning the scope of the reaction, we found that the size of the alkyl chain of the amide substituent R^2^ did not influence the reaction outcome and dihydroquinolinones **2d–2g** were all obtained in high yields and selectivities (Scheme 3). However, a secondary amide substrate (**1h**) did not cyclize and was almost fully recovered after the reaction. This suggests that the free N–H locks the molecule in an unfavorable conformation for the C–H bond activation. A range of different substituents R^1^ are tolerated on the cyclopropane ring. For example, methyl, benzyl and phenyl groups have negligible influence on the reaction outcome and provide dihydroquinolones **2b–2d** decorated with different quaternary stereocenters. Trimethylsilyl substituted cyclopropane **1o** afforded dihydroquinolinone **2o** in low yield and moderate selectivity. Spirooxindole **5** was formed as the major product presumably by an intramolecular Hiyama–Denmark coupling. Substrates **1i–1m** having R^3^ substituents on the aniline moiety gave the corresponding dihydroquinolinones with the same efficiency. However, a pyridyl substrate (**1n**) with the bromide group in the *ortho*-position reacted very sluggishly. Of note, an aromatic chloride substituent (**1m**) was tolerated by the employed palladium catalyst allowing further orthogonal functionalizations of **2m** by cross-coupling chemistry.

**Scheme 4:**
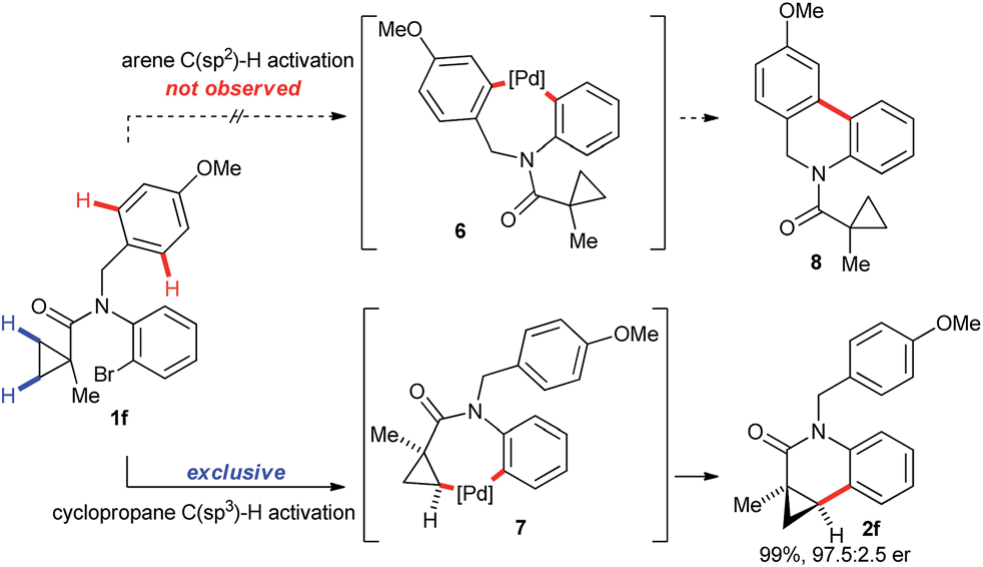
Cyclopropane C(sp^3^)–H activation over arene C(sp^2^)–H activation in **1f**. Conditions: 2.0 mol% [(η^3^-cinnamyl)Pd(Cp)], 4.0 mol% **L2**, 0.3 equiv. PivOH (30 μmol), 1.5 equiv. Cs_2_CO_3_, mesitylene, 130 °C, 12 h.

Substrate **1f** having a p-methoxybenzyl (PMB) protecting group as the R^1^ substituent on the nitrogen atom gave exclusively the cyclopropane activation product **2f** (Scheme 4). This is very remarkable as activation of the C(sp^2^)–H group of the PMB group would also proceed by the same sized 7-membered palladacycle (**6** *vs.* **7**). Generally the activation of arene C–H groups is believed to proceed more easily, making the result unexpected. One might explain this observation by a conformational bias of the amide tether favoring cyclopropane C(sp^3^)–H activation for this substrate class.

**Scheme 5:**
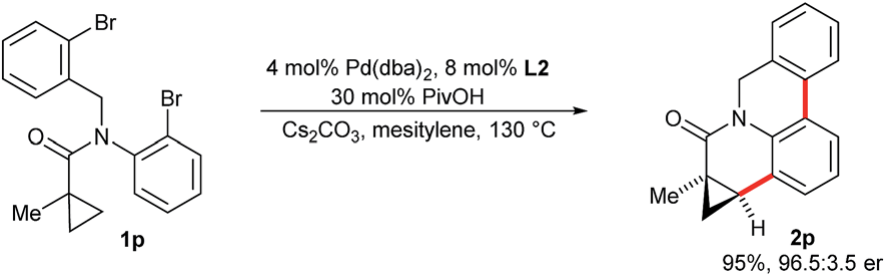
Enantioselective double C–H arylation process.

When substrate **1p** possessing two aryl bromide groups was subjected to the reaction conditions, we obtained selectively pentacyclic dihydroquinolinone **2p** in excellent yield and enantioselectivity (Scheme 5). To the best of our knowledge, this represents the only example of a tandem C–H arylation process involving an enantioselective step.

**Scheme 6:**
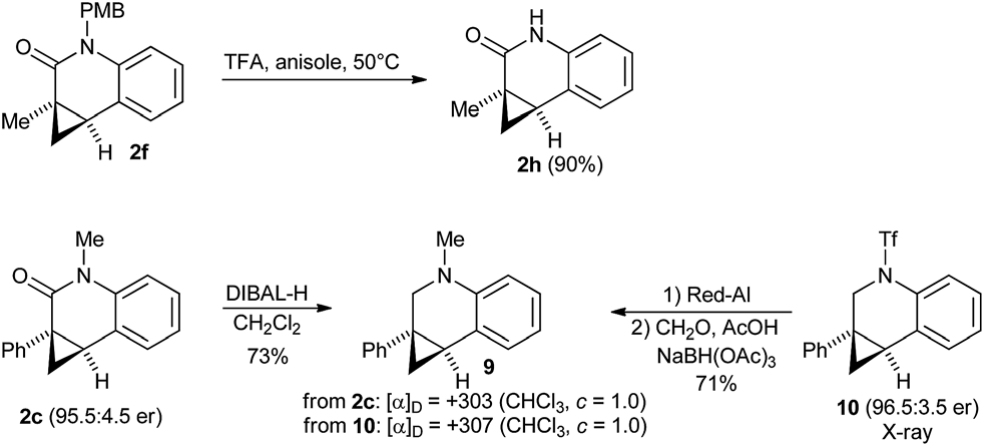
PMB group cleavage and determination of the absolute configuration.

As secondary amides fail to undergo the enantioselective cyclization, we aimed to access this product type by deprotection of **2f** (Scheme 6). The PMB group of **2f** could be efficiently removed with TFA using anisole as scavenger, giving amide **2h** in 90% yield without ring-opened byproducts of the donor–acceptor cyclopropane moiety. Finally, the absolute configuration of dihydroquinolone **2c** was established by the optical rotation of its corresponding amine **9**. **9** was independently prepared from **10**, whose absolute configuration had been previously established by X-ray crystallographic analysis.^8*a*^ Both have a comparable optical rotation (+307 prepared from **10** and +303 prepared from **2f**), thus the same absolute configuration was attributed to all lactams **2**.

### Enantioselective C–H functionalization of aminocyclopropanes

**Scheme 7:**
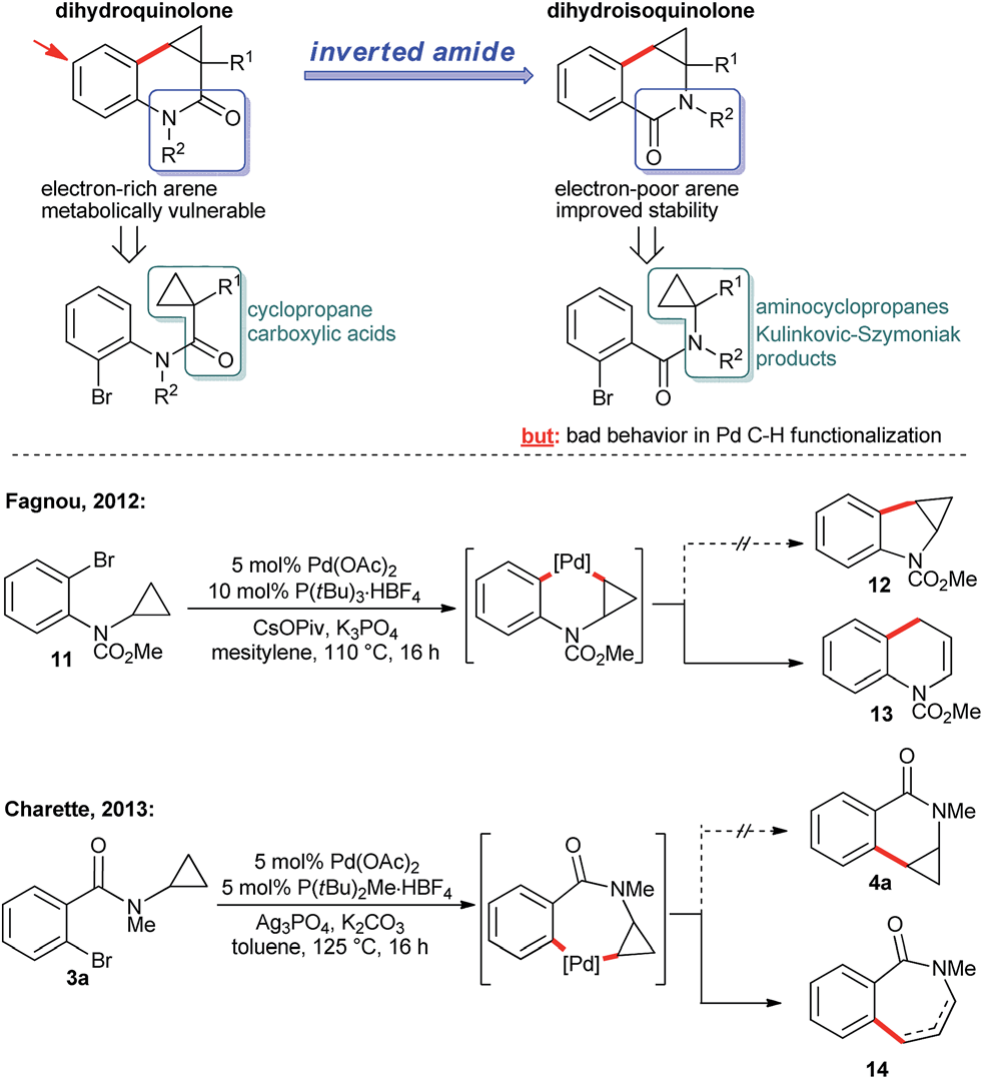
Reports on cyclopropane ring-opening under Pd^0^/Pd^II^ catalysis.

The dihydroquinolones **2** possess an electron-rich aromatic ring. Consequently, such compounds are vulnerable towards metabolic oxidation at the *para*-position with respect to the nitrogen atom. Structurally related dihydroisoquinolones **4** having an inverted amide structure (Scheme 7) are valuable building blocks^13^ and possess an electron-poorer and more stable arene core. Moreover, the required aminocyclopropane precursors for the substrate synthesis are conveniently accessible by Kulinkovich–Szymoniak reactions.^14^ Therefore, a similar enantioselective C–H functionalization access to the cyclopropane bearing dihydroisoquinolones **4** would be of high value. However, such aminocyclopropanes behave very differently in Pd(0)-catalyzed C–H bond functionalization reactions, leading to a destruction of the valuable cyclopropane moiety by C–C bond cleavage (Scheme 7). For instance, Fagnou reported the conversion of aminocyclopropane **11** into dihydroquinoline **13**.^7*i*^ Along the same lines, Charette found that **3a** is cyclized to a mixture of dihydrobenzapepinones **14**.^7*g*^ These reports point towards the strong influence of the amino group on the ring opening. Interestingly, Charette observed some amounts of the intact cyclopropane product under different conditions. Futhermore, Fagnou reported that an independently synthesized cyclopropane product proved to be stable under the reaction conditions. These two findings made us confident that a carefully chosen ligand on the palladium might prevent the ring-opening pathway and would instead provide the desirable cyclization product with an intact cyclopropane moiety.

To explore the influence of different phosphines ligands and reaction conditions on the product distribution, we chose unsubstituted aminocyclopropane **3a** as a model substrate (Table 1). The bulky and electron-rich *t*Bu_3_P leads almost exclusively to ring-opened products **14a** and **14b** which is in full agreement with the findings of Charette (entry 1). Tricyclohexylphosphine and triphenylphosphine mostly suppressed the ring-opening pathway, leading to a mixture of the desired dihydroisoquinolone **4a** and spiro-cyclic product **15** arising from a methine C–H activation^7e,f^ (entries 2 and 3). No ring-opened product was observed with xylyl ligand **L2** providing **4a** in 63% yield (entry 4). *t*Butyl ligand **L3** proved to be very unselective, giving a mixture of all four products (entry 5). Overall, there is a trend that less bulky ligands (PCy_3_ *vs. t*Bu_3_P and **L2** *vs.* **L3**) favor conservation of the cyclopropyl unit.

**Table 1:** Influence of the ligand on the product distribution^*a*^

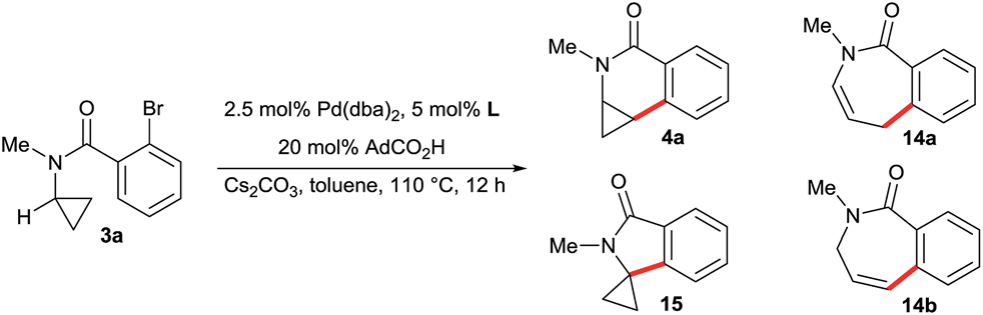					
Entry	**L**	**4a** ^*b*^ (%)	**15** ^*b*^ (%)	**14a** ^*b*^ (%)	**14b** ^*b*^ (%)
1	*t*Bu_3_P	4	<1	59	21
2	PCy_3_	58	30	8	—
3	PPh_3_	40	40	11	—
*4*	***L2***	*63*	*33*	*<1*	—
5	**L3**	12	40	28	8

^*a*^Reaction conditions: 0.05 mmol **3a**, 2.5 mol% Pd(dba)_2_, 5.0 mol% **L**, 20 mol% AdCO_2_H, 1.5 equiv. Cs_2_CO_3_ 0.25 M in toluene, 110 °C, 12 h.

^*b*^Determined by ^1^H-NMR with an internal standard.

With the above proven suitability of the Taddol phosphoramidites as ligands for the preservation of the cyclopropane moiety during the C–H functionalization process, we fine-tuned the catalyst composition for optimal enantioselectivity (Table 2). The parent ligand **L1** is not competent, giving **4b** only in trace amounts (entry 1). The already (for the above-described functionalization of amides **2**) successful xylyl version **L2** gave dihydroisoquinolone **4b** in almost quantitative yield and very good enantioselectivity of 93.5 : 6.5 (entry 2). Ligands having larger aryl or amino groups are inferior (entries 3–5). As expected from the CMD mechanism,^15^ the carboxylic acid has a critical impact on the reaction outcome. In its absence, only traces of product are formed (entry 6). Acetic or xanthene carboxylic acid are detrimental for the enantioselectivity (entries 7 and 8) and pivalic acid gives a marginally reduced er (entry 9). Notably, the reaction works equally well using K_2_CO_3_ instead of Cs_2_CO_3_ (entry 10). The catalyst loading was reduced to 2.5 mol% Pd and **4b** could be isolated in 93% yield with identical enantioselectivity (entry 11).

**Table 2:** Optimization of the enantioselective aminocyclopropane activation^*a*^

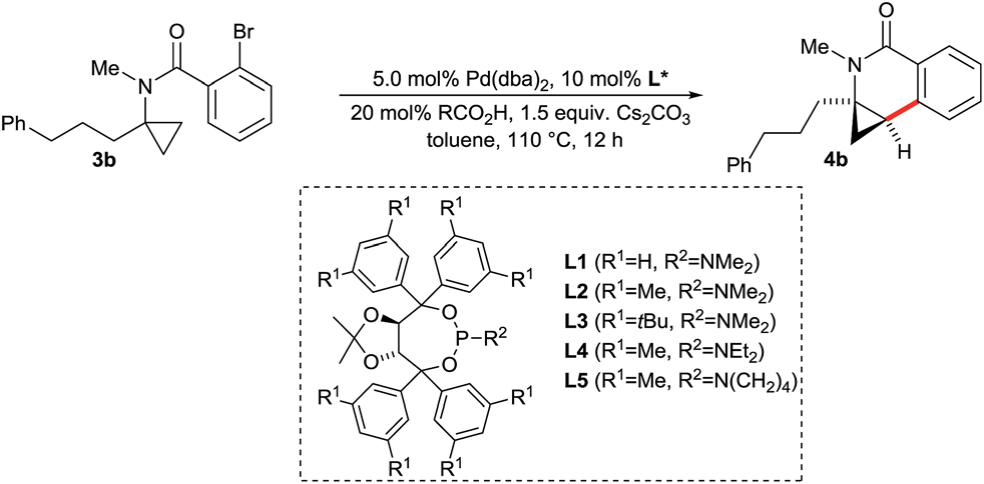				
Entry	L*	RCO_2_H	% yield of **4b** ^*b*^	er^*c*^
1	**L1**	AdCO_2_H	2	n.d.
2	**L2**	AdCO_2_H	99	93.5 : 6.5
3	**L3**	AdCO_2_H	55	85.5 : 14.5
4	**L4**	AdCO_2_H	94	90 : 10
5	**L5**	AdCO_2_H	99	93 : 7
6	**L2**	—	3	n.d.
7	**L2**	AcOH	86	60 : 40
8	**L2**	XanthCO_2_H	25	77 : 23
9	**L2**	PivOH	92	92.5 : 7.5
10^*d*^	**L2**	AdCO_2_H	99	93.5 : 6.5
*11* ^*e*^	***L2***	*AdCO*_*2*_ *H*	*96 (93)*	*93.5 : 6.5*

^*a*^Reaction conditions: 0.05 mmol **3b**, 5.0 mol% Pd(dba)_2_, 10 mol% L*, 20 mol% RCO_2_H, 1.5 equiv. Cs_2_CO_3_ 0.1 M in toluene, 110 °C, 12 h.

^*b*^Determined by ^1^H-NMR with an internal standard (isolated yield in parentheses).

^*c*^er values were determined by HPLC with a chiral stationary phase.

^*d*^With K_2_CO_3_ instead of Cs_2_CO_3_.

^*e*^With 2.5 mol% Pd(dba)_2_, 5.0 mol% **L2**, 0.25 M in toluene.

**Scheme 8:**
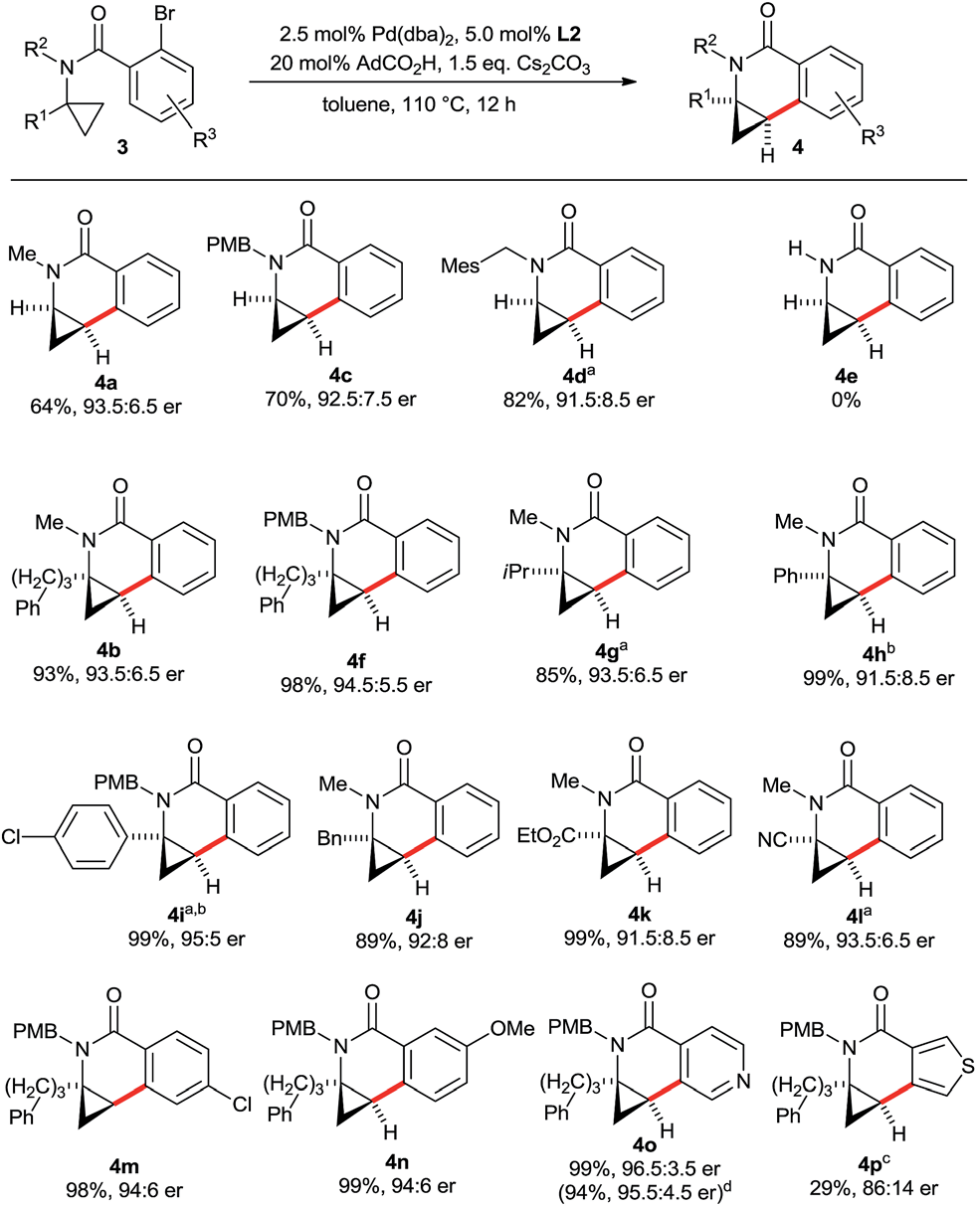
Scope of the enantioselective aminocyclopropane cyclization. Reaction conditions: **3** (0.10 mmol), Cs_2_CO_3_ (0.15 mmol), Pd(dba)_2_ (2.50 μmol), **L2** (5.00 μmol), AdCO_2_H (20.0 μmol), 0.25 M in toluene at 110 °C for 12 h. Yields of isolated products **4**; er's determined by HPLC with a chiral stationary phase. ^a^ With Pd(dba)_2_ (5.00 μmol) and **L2** (10.0 μmol) for 12 h. ^b^ 24 h reaction. ^c^ With Pd(dba)_2_ (5.00 μmol) and **L2** (10.0 μmol) in mesitylene at 130 °C for 12 h. ^d^ 1.0 mmol scale.

Next, we evaluated the scope for the aminocyclopropane activation and cyclization to dihydroisoquinolones with **L2** under the optimized conditions (Scheme 8). First, a variety of amide substituents R^2^ were checked. C–H groups of methyl, alkyl and benzyl groups are neither activated nor do significantly interfere with the efficiency of the cyclopropane activation. For substrates having no additional substituent R^1^ on the cyclopropane ring, larger groups R^2^ reduce the amount of the spirocyclic by-product (**4a**, **4c** and **4d**). A secondary amide (**3e**) is not a competent substrate and the starting material was recovered. Presumably, the unfavourable conformation of a secondary amide^16^ precludes the activation of the cyclopropane. The reaction was then evaluated for tolerance of the cyclopropane substituent R^1^. A broad variety of groups with potentially activatable C–H bonds such as linear alkyl (**4b**), branched alkyl (**4g**), aryl (**4h**, **4i**) and benzyl (**4j**) selectively react at the desired cyclopropane unit, maintaining excellent yields and high enantioselectivities. Moreover, aminocyclopropanes bearing common versatile functional groups such as ester (**4k**) or nitrile (**4l**) are compatible with the process. The substitution pattern R^3^ of the aryl portion can be varied as well, as exemplified by *p*-Cl (**4m**) and *m*-MeO (**4n**) groups. Heteroaryl bromides were also evaluated. In this respect, pyridyl containing substrate **3o** performs very well. When running the reaction at 10-fold increased scale, the product **4o** was isolated in 94% yield and 95.5 : 4.5 er. In contrast, thiophene substrate **3p** proved to be more reluctant and required a higher reaction temperature resulting in a somewhat eroded yield and enantioselectivity of **4p**.

**Scheme 9:**
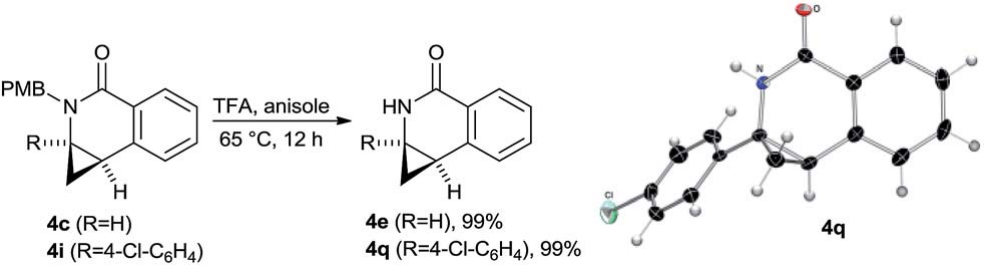
Removal of the PMB group and X-ray crystal structure of product **4q**.

To provide cyclized products of secondary amides such as **4e** which are not directly accessible, cleavage of the PMB group was targeted. It could be efficiently removed by heating the lactams in TFA with anisole as carbenium ion scavenger (Scheme 9). The secondary amides **4e** and **4q** were obtained in virtually quantitative yield. The *p*-chloro phenyl substituted lactam **4q** gave single crystals suitable for X-ray crystallographic analysis,† unambiguously providing its absolute configuration. The same preference as previously observed for the dihydroquinolones **2** was found.

### An enantioselective C–H functionalization route for the BMS-791325 ring system

**Scheme 10:**
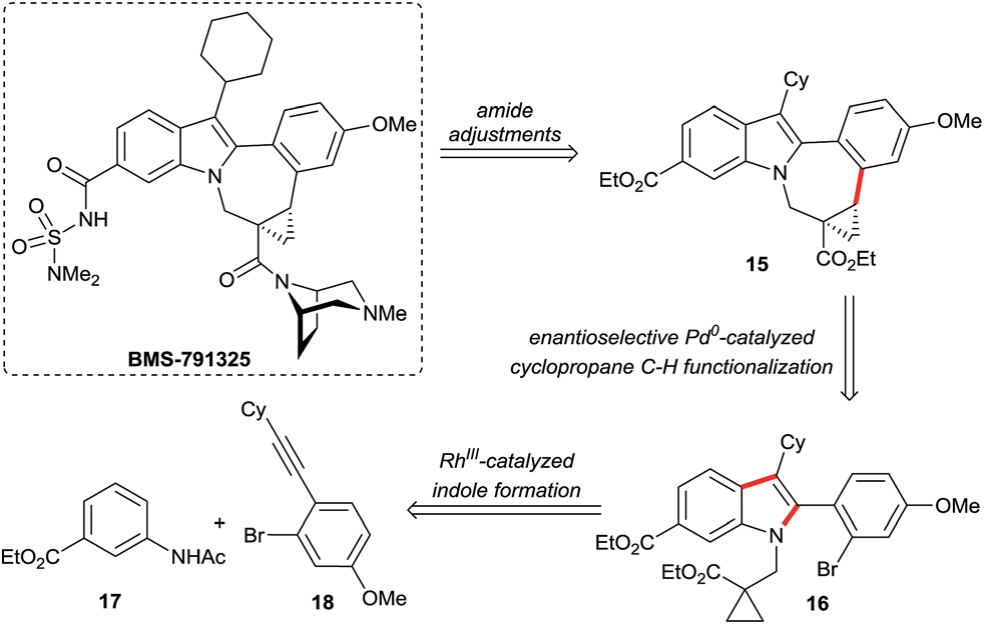
A consecutive C–H activation strategy for BMS-791325.

The attractive properties of the cyclopropane moiety as rigid^17^ and metabolically more stable surrogates of olefins^18^ made them sought after design elements in pharmaceuticals.^2^ In this respect, Bristol Myers Squibb's indolobenzazepine BMS-791325, a hepatitis C virus NS5B replicase inhibitor,^2*f*^ is a prime example (Scheme 10). The replacement of the double bond by a cyclopropane removed a potentially reactive Michael acceptor. Moreover it introduced a conformational constraint across the indolobenzazepine ring system leading to productive interactions between the cyclopropyl group and the NS5B protein.^19^ Owing to its favorable pharmacological properties, BMS-791325 is undergoing clinical evaluation in conjunction with the NS5A inhibitor daclatasvir and the NS3/4A protease inhibitor asunaprevir. From the chemical point of view, the structure of BMS-791325 has interesting and noteworthy features. The highest concentration of molecular complexity resides in its chiral trisubstituted cyclopropane ring comprising also a quaternary stereogenic center. In addition, it is part of the saturated and largest ring system of the molecule. We hypothesized that the pentacyclic core of **15** might be accessed by an enantioselective intramolecular cyclopropane arylation of substrate **16** (Scheme 10). This would allow the use of an achiral indole substrate. In turn, the required heavily substituted indole **16** could be assembled by a Rh(iii)-catalyzed C–H functionalization from simple acetanilide **17** and alkyne **18**. Such a consecutive C–H functionalization strategy represents a streamlined synthesis of **15** and allows also for the rapid synthesis of analogs.

**Scheme 11:**
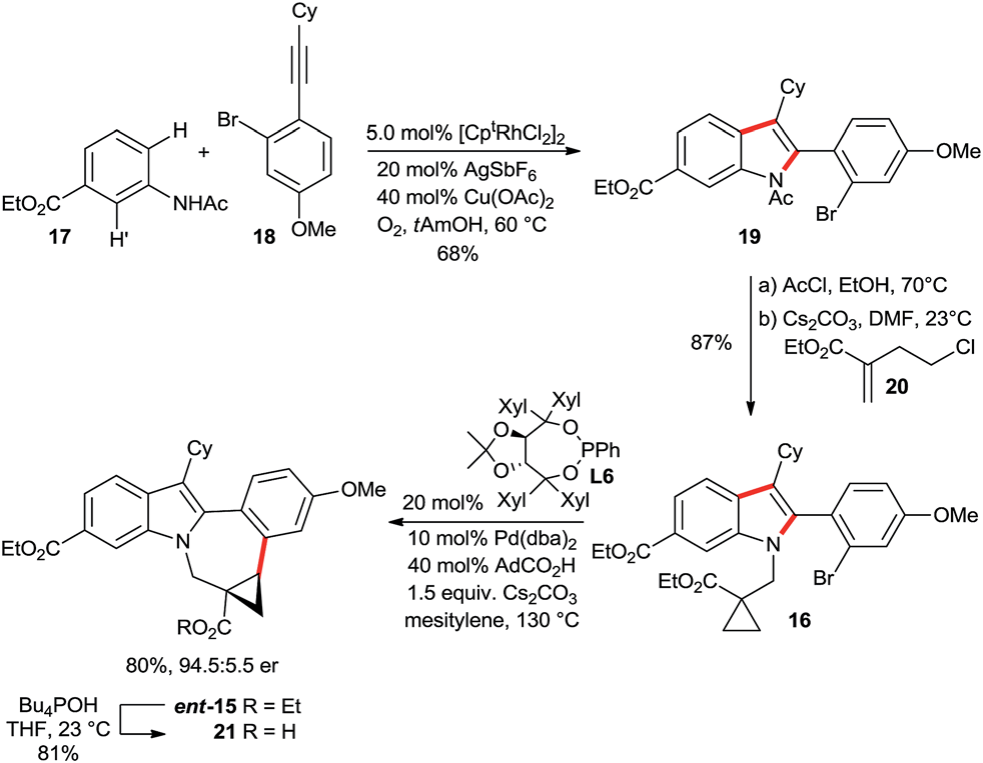
Enantioselective synthesis of the BMS-791325 ring system.

The synthesis started with a rhodium(iii)-catalyzed indole synthesis^20^ linking acetanilide **17** and alkyne **18** (Scheme 11). Both represent a highly challenging substrate combination. In consequence, the hindered nature of the alkyne in addition to regioselectivity issues (H *vs.* H′ activation and regioselectivity of the alkyne incorporation) required a modification of the original Fagnou conditions.^20*b*^ A viable solution was found using the 1,3-di-*tert*-butyl cyclopentadienyl (Cp^*t*^)^21^ instead of Cp* in combination with catalytic amounts of copper(ii) acetate and oxygen as terminal oxidant. This system provided indole **19** in 68% isolated yield without any H′ activation and an alkyne regioselectivity of 15 : 1 in favor of **19**. Subsequently, the *N*-acetyl group of the indole was cleaved under acidic conditions. The installation of the required hindered cyclopropylcarboxylate moiety was efficiently achieved with chloro-substituted acrylate **20**.^22^ In the presence of cesium carbonate in DMF, a smooth conjugate addition/intramolecular electrophilic trapping provides the desired C–H activation substrate **16** in excellent yield at ambient temperature. The hypothesized enantioselective cyclopropane activation was subsequently tested with our above described catalyst system. Pleasingly, the cyclization efficiently took place, providing ***ent*-15** in 73% yield and an enantiomeric ratio of 92.5 : 7.5 when **L2** was used. We found that the selectivity could be further enhanced using Taddol phosphonite **L6**, giving ***ent*-15** in 80% yield and 94.5 : 5.5 er.‡
‡The absolute configuration was determined based on the optical rotation of a reported derivative, see ESI† for details. To the best of our knowledge, this transformation represents the first example of enantioselective Pd(0)-catalyzed C(sp^3^)–H arylation to form a seven-membered ring. Subsequently, we could show that the ethyl esters can be differentiated. The ester adjacent to the cyclopropane moiety was selectively saponified employing Bu_4_POH.

## Conclusions

In summary, we have reported an enantioselective palladium(0)-catalyzed C–H arylative cyclization strategy of cyclopropanes. The method enables rapid access to a broad range of versatile dihydroquinolone and dihydroisoquinolone building blocks in high yields and enantioselectivities using simple Taddol-based phosphoramidite ligands. Notably, in contrast to previous work, the cyclopropane group remains intact during the functionalization of aminocyclopropane substrates. Moreover, the method is not limited to the construction of 6-membered ring systems and was successfully applied for the efficient enantioselective construction of the 7-membered ring of the cyclopropyl indolobenzazepine core of BMS-791325.

## References

[cit1] de Meijere A., Kozhushkov S. I., Schill H. (2006). Chem. Rev..

[cit2] Gootz T. D., Zaniewski R., Haskell S., Schmieder B., Tankovic J., Girard D., Courvalin P., Polzer R. J. (2006). Antimicrob. Agents Chemother..

[cit3] Lebel H., Marcoux J.-F., Molinaro C., Charette A. B. (2003). Chem. Rev..

[cit4] Eaton P. E., Lee C.-H., Xiong Y. (1989). J. Am. Chem. Soc..

[cit5] Lauru S., Simpkins N. S., Gethin D., Wilson C. (2008). Chem. Commun..

[cit6] Chen X., Engle K. M., Wang D.-H., Yu J.-Q. (2009). Angew. Chem., Int. Ed..

[cit7] Kim J., Sim M., Kim N., Hong S. (2015). Chem. Sci..

[cit8] Saget T., Cramer N. (2012). Angew. Chem., Int. Ed..

[cit9] Giri R., Shi B.-F., Engle K. M., Maugel N., Yu J.-Q. (2009). Chem. Soc. Rev..

[cit10] Rousseaux S., Gorelsky S. I., Chung B. K. W., Fagnou K. (2010). J. Am. Chem. Soc..

[cit11] Harmata M., Hong X. (2007). Org. Lett..

[cit12] Albicker M. R., Cramer N. (2009). Angew. Chem., Int. Ed..

[cit13] Fisher M. J., Gunn B. P., Harms C. S., Kline A. D., Mullaney J. T., Scarborough R. M., Skelton M. A., Um S. L., Utterback B. G., Jakubowski J. A. (1997). Bioorg. Med. Chem. Lett..

[cit14] Bertus P., Szymoniak J. (2007). Synlett.

[cit15] Ackermann L. (2011). Chem. Rev..

[cit16] Gonzalez-de-Castro A., Broughton H., Martinez-Perez J. A., Espinosa J. F. (2015). J. Org. Chem..

[cit17] Armstrong P. D., Cannon G. J., Long J. P. (1968). Nature.

[cit18] Bender D. M., Peterson J. A., McCarthy J. R., Gunaydin H., Takano Y., Houk K. N. (2008). Org. Lett..

[cit19] Zheng X., Hudyma T. W., Martin S. W., Bergstrom C., Ding M., He F., Romine J., Poss M. A., Kadow J. F., Chang C.-H., Wan J., Witmer M. R., Morin P., Camac D. M., Sheriff S., Beno B. R., Rigat K. L., Wang Y.-K., Fridell R., Lemm J., Qiu D., Liu M., Voss S., Pelosi L., Roberts S. B., Gao M., Knipe J., Gentles R. G. (2011). Bioorg. Med. Chem. Lett..

[cit20] Huestis M. P., Chan L., Stuart D. R., Fagnou K. (2011). Angew. Chem., Int. Ed..

[cit21] Hyster T. K., Dalton D. M., Rovis T. (2015). Chem. Sci..

[cit22] Lachia M., Iriart S., Baalouch M., De Mesmaeker A., Beaudegnies R. (2011). Tetrahedron Lett..

