# Supplementary material for: Enantioselective palladium(0)-catalyzed intramolecular cyclopropane functionalization: access to dihydroquinolones, dihydroisoquinolones and the BMS-791325 ring system
Source: Chem Sci. 2015 Jun 17;6(9):5164–71. doi: 10.1039/c5sc01909e (PMC5667185; doi:10.1039/c5sc01909e)
Supplement: Supplementary file 1 [file SC-006-C5SC01909E-s001.pdf]

Electronic Supplementary Material (ESI) for Chemical Science.
This journal is © The Royal Society of Chemistry 2015

Electronic Supplementary Information

Table of Contents:

**General Methods**	S2
**Experimental procedures and characterization data**	S3
**^{1}H- and ^{13}C-NMR Spectra**	S60

General Methods

All reactions were carried out under an atmosphere of nitrogen in oven-dried glassware with magnetic stirring, unless otherwise indicated. Toluene, dichloromethane and THF were purified by a Innovative Technology Solvent Delivery System. Chemicals were used as obtained from the suppliers. Flash chromatography was performed with Silicycle silica gel 60 (0.040-0.063 μm grade). Analytical thin-layer chromatography was performed with commercial glass plates coated with 0.25 mm silica gel (E. Merck, Kieselgel 60 F254). Compounds were either visualised under UV-light at 254 nm or by dipping the plates in an aqueous potassium permanganate solution followed by heating. Proton nuclear magnetic resonance (^{1}H NMR) data were acquired at 400 MHz on a Bruker AV400 spectrometer. Chemical shifts (δ) are reported in parts per million (ppm) relative to residual chloroform (s, 7.26 ppm). Splitting patterns are designated as s, singlet; d, doublet; t, triplet; q, quartet; sept, septet; m, multiplet, br, broad. Proton decoupled Carbon-13 nuclear magnetic resonance (^{13}C NMR) data were acquired at 100 MHz on a Bruker AV400 spectrometer. Chemical shifts (δ) are reported in parts per million (ppm) relative to residual chloroform (77.16±0.06 ppm). Unless otherwise stated, all NMR spectra were measured at 298 K. Infrared (IR) data were recorded on an Alpha-P Bruker FT-IR Spectrometer. Absorbance frequencies are reported in reciprocal centimeters (cm^{-1}). HR-MS measurements were performed by an Agilent LC-MS TOF. High resolution mass are given in *m/z*. Optical rotations were measured on a Polartronic M polarimeter using a 0.5 cm cell with a Na 589 nm filter. X-ray analysis was performed by Dr. R. Scopelliti at the EPF Lausanne.

Experimental procedures and characterization data

Synthesis of $Pd(dba)_2$

$Pd(dba)_2$ was synthesized following a modified literature procedure (Y. Takahashi *et al.*, *J. Chem. Soc. D.* 1970, **17**, 1065):

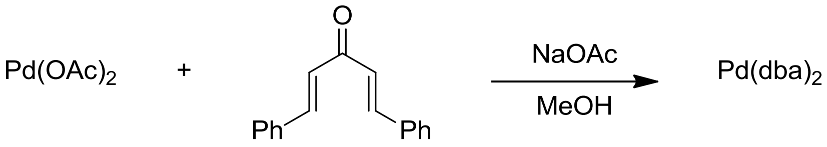

Dibenzyledeneacetone (1.72 g, 7.35 mmol, 3.3 eq) and sodium acetate (1.46 g, 17.8 mmol, 8.0 eq) were dissolved in degassed MeOH (56 ml) at 50 °C and the mixture let cool down to rt. Palladium acetate (0.50 g, 2.23 mmol) was added in one portion under a flow of nitrogen and the reaction mixture stirred at 40 °C for 5 h. After cooling down to rt, the mixture was filtered, the solid washed copiously with water and acetone and dried under high vacuum at rt for 24 h. Elemental analysis: C (%) calculated 71.02 found 74.12, H (%) calculated 4.91, found 5.12.

Ligand synthesis

Ligands were synthesized following literature procedures:

L1: A. Alexakis *et al.*, *Eur. J. Org. Chem.* 2000, **24**, 4011.
L2 and **L4**: P. van Leeuwen *et al.*, *Chem. Eur. J.* 2004, **10**, 6232.
L3: J. Morken *et al.*, *Org. Lett.* 2005, **7**, 5505.
L5: T. Rovis *et al.*, *J. Am. Chem. Soc.* 2008, **130**, 3262.
L6: J. Morken *et al.*, *J. Am. Chem. Soc.* 2008, **130**, 4978.

General procedures for the synthesis of *N*-(2-bromophenyl)cyclopropylamides (1)

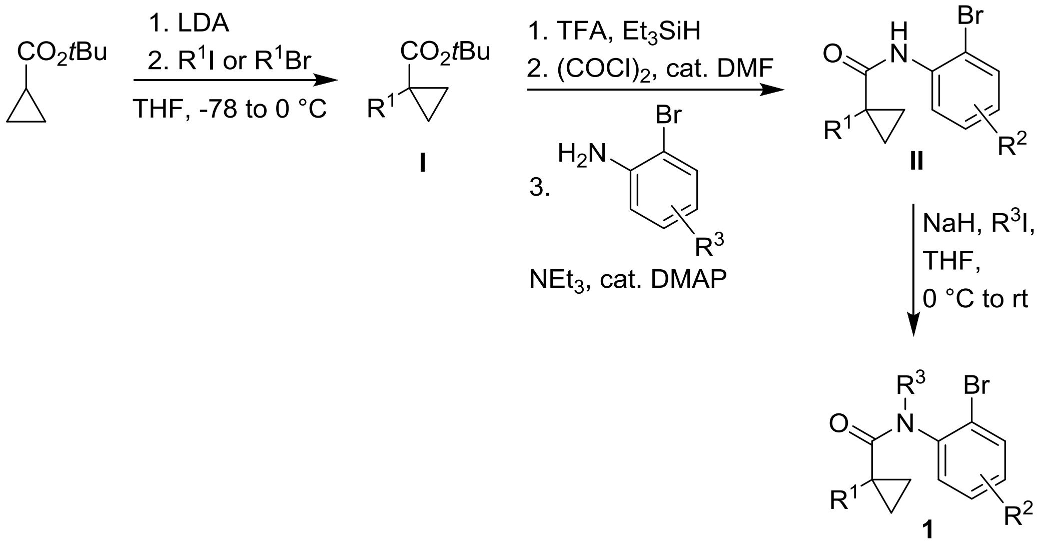

Synthesis of **I** – Substituted cyclopropyl esters were synthesized according to a procedure described in literature (R. Häner, T. Maetzke, D. Seebach, *Helv. Chim. Acta* 1986, **69**, 1655.)

Synthesis of **II** – Removal of the *tert*-butyl group was performed according to a literature report (A. Mehta, R. Jaouhari, T. J. Benson, K. T. Douglas, *Tetrahedron Lett.* 1992, **33**, 5441.) The thus obtained carboxylic acid (1.0 eq.) was dissolved in DCM (0.3 M). Two drops of DMF were added and the reaction mixture was cooled to 0 °C. Oxalyl chloride (1.1 eq.) was added and the reaction mixture stirred at 23 °C until gas evolution ceased. This mixture was then added to a stirred solution of *ortho*-bromoaniline (1.2 eq.) and triethylamine (2.5 eq.) in DCM (0.3 M) at 0 °C. After stirring at 0 °C for 5 minutes, DMAP (5-10 mol%) was added. The reaction mixture was stirred overnight at 23 °C and quenched by pouring into 0.5 M aq. HCl. Et_2O was added and the layers were separated. The organic layer was washed with 0.5 M aq. HCl and then with a 1/1 mixture of brine and aq. sat. $NaHCO_3$. The organic layer was dried over $MgSO_4$ and the solvent removed under reduced pressure. Purification by column chromatography on silica gel afforded **II**.

Synthesis of **1** – A suspension of NaH (2.0 eq.) in dry THF was cooled to 0 °C and a solution of amide **II** (1.0 eq.) in THF (0.2 M) was added dropwise. After 5 minutes, the corresponding alkyl iodide (4.0 eq.) was added and the reaction mixture was stirred overnight at 23 °C. When an alkyl bromide or chloride was used, KI (1.0 eq.) was initially added. The reaction was quenched by slowly pouring the reaction mixture to aq. sat. $NaHCO_3$ at 0 °C and the aq. phase extracted with Et_2O. The combined org. phases were washed with aq. sat. $Na_2S_2O_3$, brine, dried over $MgSO_4$ and the solvent removed under reduced pressure. Purification by column chromatography on silica gel afforded the tertiary amides **1**.

<u>*N*-(2-bromophenyl)-*N*-ethyl-1-methylcyclopropanecarboxamide (**1a**):</u>

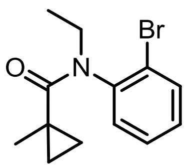

^{1}H NMR (400 MHz, $CDCl_3$) δ = 7.68 (d, *J* = 8.1 Hz, 1H), 7.41–7.33 (m, 1H), 7.26–7.17 (m, 2H), 4.20 (dq, *J* = 14.1, 7.0 Hz, 1H), 3.44–2.79 (m, 1H), 1.32–1.18 (m, 1H), 1.09 (t, *J* = 7.1 Hz, 3H), 1.03–0.60 (m, 4H), 0.51–0.19 (m, 2H) ppm; **^{13}C{^{1}H} NMR** (101 MHz, $CDCl_3$) δ = 174.0, 141.2, 133.8, 131.9, 129.1, 127.9, 124.0, 44.4, 21.6, 21.1, 16.4, 14.8, 12.4 ppm; **IR (ATR):** $\tilde{\nu}$ = 3062, 2974, 2933, 2873, 1646, 1584, 1474, 1448, 1391, 1324, 1271, 1246, 1227, 1134, 1119, 1058, 1029, 910, 762, 727, 646, 589 cm^{-1}; **HRMS (ESI)** calculated. for $[C_{13}H_{17}{}^{79}BrNO]^+$ 282.0488, found 282.0487; ***R*$_f$**: 0.40 (pentane/EtOAc 5/1); **m.p.**: 69-70 °C.

<u>1-benzyl-*N*-(2-bromophenyl)-*N*-methylcyclopropanecarboxamide (**1b**):</u>

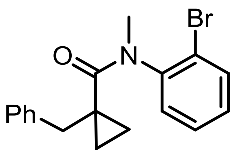

^{1}H NMR (400 MHz, $CDCl_3$) δ = 7.64 (d, *J* = 8.6 Hz, 1H), 7.55–6.69 (m, 8H), 3.19 (s, 3H), 2.95 (d, *J* = 14.5 Hz, 1H), 2.65–1.98 (m, 1H), 1.40–0.13 (m, 4H) ppm; **^{13}C{^{1}H} NMR** (101 MHz, $CDCl_3$) δ = 173.5, 142.9, 138.5, 133.7, 130.3, 129.4, 129.2, 128.4, 128.2, 126.4, 123.0, 40.4, 38.0, 25.8, 14.4, 11.8 ppm; **IR (ATR):** $\tilde{\nu}$ = 3084, 3061, 3025, 3004, 2921, 2861, 1647, 1583, 1494, 1477, 1453, 1439, 1369, 1331, 1301, 1241, 1117, 1089, 1055, 1029, 989, 917, 764, 728, 701, 647, 586, 520, 484, 463, 429 cm^{-1}; **HRMS (ESI)** calculated for $[C_{18}H_{19}{}^{79}BrNO]^+$ 344.0645, found 344.0633; ***R*$_f$**: 0.20 (pentane/EtOAc 5/1).

<u>*N*-(2-bromophenyl)-*N*-methyl-1-phenylcyclopropanecarboxamide (**1c**):</u>

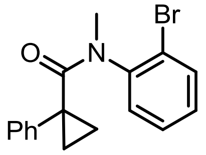

^{1}H NMR (400 MHz, $CDCl_3$) δ = 7.68–6.58 (m, 9H), 3.17 (s, 3H), 1.77–0.80 (m, 4H) ppm; **^{13}C{^{1}H} NMR** (101 MHz, $CDCl_3$) δ = 172.9, 142.3, 140.3, 133.3, 131.2, 128.8, 128.0, 128.0, 127.8, 126.0, 123.5, 37.8, 30.8, 15.7, 14.3 ppm; **IR (ATR):** $\tilde{\nu}$ = 3083, 3058, 3024, 3010, 2931, 1649, 1600, 1583, 1495, 1477, 1443, 1418, 1358, 1301, 1228, 1132, 1118, 1097, 1075, 1056, 1029, 951, 937, 760, 739, 727, 698, 650, 607, 544, 489, 463, 395 cm^{-1}; **HRMS (ESI)** calculated for $[C_{17}H_{17}{}^{79}BrNO]^+$ 330.0488, found 330.0496; ***R*$_f$**: 0.50 (pentane/EtOAc 3/1).

N-(2-bromophenyl)-*N*,1-dimethylcyclopropanecarboxamide (**1d**):

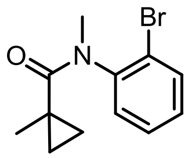

^{1}H NMR (400 MHz, $CDCl_3$) δ = 7.67 (dd, *J* = 8.0, 1.0 Hz, 1H), 7.37 (td, *J* = 7.6, 1.4 Hz, 1H), 7.33–7.27 (m, 1H), 7.22 (td, *J* = 8.0, 1.5 Hz, 1H), 3.21 (s, 3H), 1.35–0.25 (m, 7H) ppm; **^{13}C{^{1}H} NMR** (101 MHz, $CDCl_3$) δ = 174.6, 143.2, 133.8, 130.3, 129.2, 128.4, 123.3, 37.9, 21.5, 20.9, 16.4, 14.9 ppm; **IR (ATR):** $\tilde{\nu}$ = 3062, 3005, 2965, 2931, 2874, 1647, 1584, 1477, 1419, 1386, 1369, 1357, 1235, 1130, 1118, 1090, 1055, 1029, 766, 747, 729, 586 cm^{-1}; **HRMS (ESI)** calculated. for $[C_{12}H_{15}{}^{79}BrNO]^+$ 268.0332, found 268.0329; ***R*$_f$**: 0.35 (pentane/EtOAc 5/1); **m.p.**: 70-71 °C.

N-(2-bromophenyl)-1-methyl-*N*-propylcyclopropanecarboxamide (**1e**):

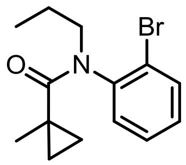

^{1}H NMR (400 MHz, $CDCl_3$) δ = 7.70 (d, *J* = 7.9 Hz, 1H), 7.38 (t, *J* = 7.6 Hz, 1H), 7.30–7.22 (m, 2H), 4.27–4.00 (m, 1H), 3.21–2.81 (m, 1H), 1.67–0.71 (m, 10H), 0.49–0.20 (m, 2H) ppm; **^{13}C{^{1}H} NMR** (126 MHz, $CDCl_3$) δ = 174.0, 141.5, 133.8, 131.9, 129.1, 127.9, 124.0, 51.2, 21.7, 21.2, 20.5, 16.5, 14.8, 11.3 ppm; **IR (ATR):** $\tilde{\nu}$ = 3063, 2963, 2933, 2874, 1648, 1583, 1474, 1433, 1390, 1327, 1282, 1223, 1140, 1121, 1061, 1029, 944, 769, 745, 729, 610, 450 cm^{-1}; **HRMS (ESI)** calculated for $[C_{14}H_{19}{}^{79}BrNO]^+$ 296.0645, found 296.0644; ***R*$_f$**: 0.45 (pentane/EtOAc 5/1); **m.p.**: 34-35 °C.

N-(2-bromophenyl)-*N*-(4-methoxybenzyl)-1-methylcyclopropanecarboxamide (**1f**):

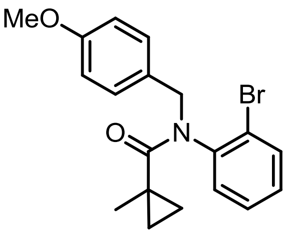

^{1}HNMR (400 MHz, $CDCl_3$) δ = 7.67 (dd, *J* = 5.8, 3.4 Hz, 1H), 7.23–7.09 (m, 2H), 7.10–7.01 (m, 2H), 6.83–6.69 (m, 3H), 5.60 (d, *J* = 14.3 Hz, 1H), 4.09–3.87 (m, 1H), 3.76 (s, 3H), 1.38–1.25 (m, 1H), 1.18–0.50 (m, 4H), 0.49–0.13 (m, 2H) ppm; **^{13}C{^{1}H} NMR** (101 MHz, $CDCl_3$) δ = 174.1, 158.9, 140.7, 133.7, 132.6, 130.6, 129.4, 129.2, 127.6, 123.8, 113.6, 55.2, 52.0, 21.7, 21.2, 16.4, 14.9 ppm; **IR (ATR):** $\tilde{\nu}$ = 3063, 3001, 2960, 2934, 2876, 2835, 1645, 1611, 1584, 1511, 1473, 1439, 1389, 1323, 1302, 1246, 1229, 1175, 1109, 1060, 1031, 991, 946, 847, 801, 760, 741, 729, 706, 646, 616, 569, 549, 521, 451, 396 cm^{-1}; **HRMS (ESI)** calculated for $[C_{19}H_{21}{}^{79}BrNO_2]^+$ 374.0750, found 374.0737; ***R*$_f$**: 0.10 (pentane/EtOAc 5/1); **m.p.**: 93-94 °C.

<u>*N*-(2-bromophenyl)-*N*-(2-methoxybenzyl)-1-methylcyclopropanecarboxamide (**1g**):</u>

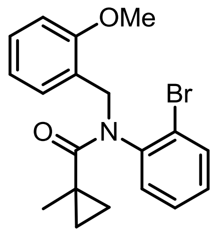

^{1}H NMR (400 MHz, $CDCl_3$) δ = 7.73–7.55 (m, 1H), 7.39–7.23 (m, 1H), 7.22–7.16 (m, 1H), 7.16–7.04 (m, 2H), 6.97–6.79 (m, 2H), 6.72 (d, *J* = 8.2 Hz, 1H), 5.47 (d, *J* = 14.5 Hz, 1H), 4.63–4.19 (m, 1H), 3.54 (s, 3H), 1.35–1.22 (m, 1H), 1.22–0.54 (m, 4H), 0.52–0.09 (m, 2H) ppm; **^{13}C{^{1}H} NMR** (101 MHz, $CDCl_3$) δ = 174.1, 157.6, 141.1, 133.2, 132.1, 130.8, 128.9, 128.6, 127.3, 125.3, 124.3, 120.4, 110.0, 54.9, 46.9, 21.8, 21.3, 16.1, 14.8 ppm; **IR (ATR):** $\tilde{\nu}$ = 3065, 3003, 2960, 2936, 2836, 1646, 1601, 1586, 1492, 1474, 1438, 1389, 1325, 1288, 1245, 1201, 1174, 1161, 1116, 1049, 1029, 987, 945, 807, 754, 726, 705, 626, 590, 453 cm^{-1}; **HRMS (ESI)** calculated for $[C_{19}H_{21}{}^{79}BrNO_2]^+$ 374.0750, found 374.0740; ***R*$_f$**: 0.45 (pentane/EtOAc 5/1); **m.p.**: 81-82 °C.

<u>*N*-(2-bromophenyl)-1-methylcyclopropanecarboxamide (**1h**):</u>

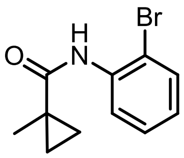

^{1}H NMR (400 MHz, $CDCl_3$) δ = 8.41 (dd, *J* = 8.3, 1.6 Hz, 1H), 8.18 (s, 1H), 7.56 (dd, *J* = 8.0, 1.5 Hz, 1H), 7.33 (ddd, *J* = 8.0, 7.5, 1.5 Hz, 1H), 6.98 (ddd, *J* = 8.0, 7.5, 1.6 Hz, 1H), 1.56 (s, 3H), 1.36 (q, *J* = 4.0 Hz, 2H), 0.76 (q, *J* = 3.9 Hz, 2H) ppm; **^{13}C{^{1}H} NMR** (101 MHz, $CDCl_3$) δ = 173.3, 135.9, 132.1, 128.4, 124.8, 121.4, 113.3, 20.3, 19.6, 17.0 ppm; **IR (ATR):** $\tilde{\nu}$ = 3418, 3006, 2965, 2875, 1685, 1587, 1522, 1470, 1432, 1388, 1316, 1282, 1244, 1163, 1120, 1023, 929, 796, 751, 663, 566, 533, 436 cm^{-1}; **HRMS (ESI)** calculated for $[C_{11}H_{13}{}^{79}BrNO]^+$ 254.0175, found 254.0173; ***R*$_f$**: 0.50 (pentane/EtOAc 10/1).

<u>*N*-(2-bromo-4-(trifluoromethoxy)phenyl)-*N*,1-dimethylcyclopropanecarboxamide (**1i**):</u>

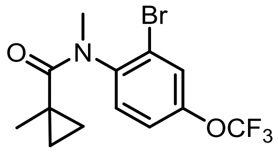

^{1}H NMR (400 MHz, $CDCl_3$) δ = 7.55 (s, 1H), 7.37–7.19 (m, 2H), 3.24 (s, 3H), 1.52–0.21 (m, 7H) ppm; **^{13}C{^{1}H} NMR** (101 MHz, $CDCl_3$) δ = 174.4, 148.3, 141.9, 130.9, 126.2, 123.8, 120.7, 120.2 (q, $J_{C\text{-}F}$ = 258.9 Hz), 37.9, 21.4, 20.8, 15.8, 14.7 ppm; **IR (ATR):** $\tilde{\nu}$ = 3086, 2971, 2935, 2880, 1655, 1595, 1575, 1489, 1424, 1387, 1357, 1253, 1217, 1170, 1124, 1090, 1052, 943, 910, 881, 832, 677, 596, 457 cm^{-1}; **HRMS (ESI)** calculated for $[C_{13}H_{14}{}^{79}BrF_3NO_2]^+$ 352.0155, found 352.0173; ***R*$_f$**: 0.55 (pentane/EtOAc 2/1); **m.p.**: 37-38 °C.

<u>*N*-(2-bromo-4-methylphenyl)-*N*,1-dimethylcyclopropanecarboxamide (**1j**):</u>

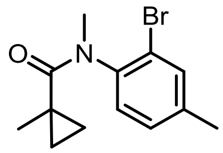

^{1}H NMR (400 MHz, $CDCl_3$) δ = 7.48 (s, 1H), 7.20–7.10 (m, 2H), 3.17 (s, 3H), 2.36 (s, 3H), 1.39–0.70 (m, 5H), 0.56–0.11 (m, 2H) ppm; **^{13}C{^{1}H} NMR** (101 MHz, $CDCl_3$) δ = 174.7, 140.5, 139.6, 134.1, 129.9, 129.1, 122.9, 38.0, 21.6, 20.9, 20.8, 16.4, 14.9 ppm; **IR (ATR):** $\tilde{\nu}$ = 3006, 2963, 2927, 2873, 1649, 1600, 1494, 1469, 1420, 1386, 1360, 1316, 1282, 1237, 1126, 1089, 1055, 1021, 946, 914, 871, 847, 826, 748, 671, 587, 515, 455, 418 cm^{-1}; **HRMS (ESI)** calculated for $[C_{13}H_{17}{}^{79}BrNO]^+$ 282.0488, found 282.0491; ***R*f**: 0.40 (pentane/EtOAc 2/1); **m.p.**: 46-47 °C.

<u>*N*-(2-bromo-5-(trifluoromethyl)phenyl)-*N*,1-dimethylcyclopropanecarboxamide (**1k**):</u>

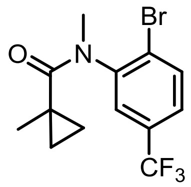

^{1}H NMR (400 MHz, $CDCl_3$) δ = 7.81 (d, *J* = 8.3 Hz, 1H), 7.55 (s, 1H), 7.48 (d, *J* = 8.3 Hz, 1H), 3.28 (s, 3H), 1.38–0.31 (m, 7H) ppm; **^{13}C{^{1}H} NMR** (101 MHz, $CDCl_3$) δ = 174.4, 143.8, 134.5, 131.1 (q, $J_{C\text{-}F}$ = 33.5 Hz), 127.4, 127.3, 125.7 (q, $J_{C\text{-}F}$ = 3.7 Hz), 123.4 (q, $J_{C\text{-}F}$ = 272.6 Hz), 37.8, 21.3, 20.8, 16.0, 14.6 ppm; **IR (ATR):** $\tilde{\nu}$ = 3068, 2972, 2934, 2913, 2883, 1655, 1603, 1575, 1480, 1415, 1387, 1333, 1297, 1257, 1235, 1172, 1128, 1078, 1048, 1029, 935, 897, 829, 748, 734, 711, 570, 519, 458, 418 cm^{-1}; **HRMS (ESI)** calculated for $[C_{13}H_{14}{}^{79}BrF_3NO]^+$ 336.0205, found 336.0211; ***R*f**: 0.50 (pentane/EtOAc 2/1); **m.p.**: 86-87 °C.

<u>*N*-(2-bromo-5-methylphenyl)-*N*,1-dimethylcyclopropanecarboxamide (**1l**):</u>

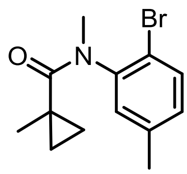

^{1}H NMR (400 MHz, $CDCl_3$) δ = 7.51 (d, *J* = 8.1 Hz, 1H), 7.09 (s, 1H), 7.02 (d, *J* = 8.1 Hz, 1H), 3.19 (s, 3H), 2.33 (s, 3H), 1.49–0.71 (m, 5H), 0.64–0.09 (m, 2H) ppm; **^{13}C{^{1}H} NMR** (101 MHz, $CDCl_3$) δ = 174.5, 142.8, 138.7, 133.3, 130.9, 130.0, 119.6, 37.9, 21.6, 20.9, 20.8, 16.4, 14.8 ppm; **IR (ATR):** $\tilde{\nu}$ = 3006, 2963, 2925, 2873, 1649, 1594, 1571, 1477, 1421, 1405, 1385, 1358, 1316, 1283, 1262, 1238, 1197, 1123, 1089, 1056, 1028, 941, 849, 811, 749, 730, 597, 563, 508, 456 cm^{-1}; **HRMS (ESI)** calculated for $[C_{13}H_{17}{}^{79}BrNO]^+$ 282.0488, found 282.0492; ***R*f**: 0.45 (pentane/EtOAc 2/1); **m.p.**: 66-67 °C.

<u>*N*-(2-bromo-4-chloro-6-fluorophenyl)-*N*,1-dimethylcyclopropanecarboxamide (**1m**):</u>

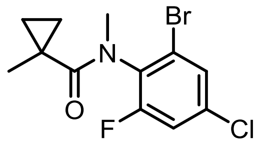

^{1}H NMR (400 MHz, $CDCl_3$) (mixture of rotamers in ratio = 2/1) δ = 7.51 (s, 0.7H), 7.44 (s, 0.3H), 7.21 (dd, *J* = 8.7, 1.9 Hz, 0.7H), 7.17 – 7.11 (m, 0.3H), 3.41 (s, 1H), 3.12 (s, 2H), 1.49 (s, 1H), 1.28-1.07 (m, 2H), 0.90 (s, 2H), 0.69 (s, 0.7H), 0.37 (s, 1.3H) ppm; **^{13}C{^{1}H} NMR** (101 MHz, $CDCl_3$) δ = 174.7, 173.8, 159.0 (d, J_{C-F} = 255.5 Hz), 158.8 (d, J_{C-F} = 255.5 Hz), 135.1 (d, J_{C-F} = 11.2 Hz), 134.5 (d, J_{C-F} = 11.2 Hz), 131.0 (d, J_{C-F} = 15.7 Hz), 130.0 (d, J_{C-F} = 15.7 Hz), 129.0 (d, J_{C-F} = 3.7 Hz), 128.7 (d, J_{C-F} = 3.7 Hz), 125.7, (d, J_{C-F} = 1.3 Hz), 124.5 (d, J_{C-F} = 2.5 Hz), 116.8 (d, J_{C-F} = 24.5 Hz), 116.8 (d, J_{C-F} = 24.2 Hz), 37.0, 36.8 (d, J_{C-F} = 1.1 Hz), 21.3 (d, J_{C-F} = 0.7 Hz), 20.9, 20.7, 20.5, 15.6 (d, J_{C-F} = 3.6 Hz), 15.3, 13.2, 12.8 ppm; **IR (ATR):** $\tilde{\nu}$ = 3083, 3000, 2967, 2933, 2904, 1666, 1606, 1583, 1485, 1460, 1387, 1374, 1354, 1247, 1194, 1119, 1107, 1081, 992, 925, 903, 869, 846, 800, 760, 733, 663, 578, 526, 429 cm^{-1}; **HRMS (ESI)** calculated for $[C_{12}H_{13}{}^{79}BrClFNO]^+$ 319.9848, found 319.9853; ***R*$_f$**: 0.25 (pentane/EtOAc 10/1).

<u>*N*-(2-bromopyridin-3-yl)-*N*,1-dimethylcyclopropanecarboxamide (**1n**):</u>

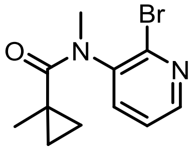

^{1}H NMR (400 MHz, $CDCl_3$) δ = 8.36 (dd, *J* = 4.7, 1.8 Hz, 1H), 7.60 (dd, *J* = 7.7, 1.7 Hz, 1H), 7.35 (dd, *J* = 7.7, 4.7 Hz, 1H), 3.28 (s, 3H), 1.33–0.81 (m, 5H), 0.64–0.31 (m, 2H) ppm; **^{13}C{^{1}H} NMR** (101 MHz, $CDCl_3$) δ = 174.3, 148.9, 143.3, 140.6, 138.3, 123.4, 37.8, 21.4, 20.7, 15.5, 14.5 ppm; **IR (ATR):** $\tilde{\nu}$ = 3050, 3006, 2968, 2931, 2875, 1650, 1470, 1449, 1399, 1368, 1281, 1242, 1202, 1137, 1117, 1090, 1070, 1053, 1022, 943, 908, 812, 747, 729, 695, 659, 639, 600, 561, 513, 481, 431 cm^{-1}; **HRMS (ESI)** calculated for $[C_{11}H_{14}{}^{79}BrN_2O]^+$ 269.0284, found 269.0275; ***R*$_f$**: 0.35 (pentane/EtOAc 1/1).

<u>*N*-(2-bromophenyl)-*N*-methyl-1-(trimethylsilyl)cyclopropanecarboxamide (**1o**):</u>

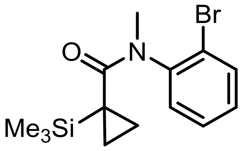

^{1}H NMR (400 MHz, $CDCl_3$) δ = 7.62 (dd, *J* = 8.0, 1.2 Hz, 1H), 7.34 (td, *J* = 7.7, 1.4 Hz, 1H), 7.25–7.12 (m, 2H), 3.57–3.05 (m, 3H), 1.42–0.99 (m, 2H), 0.98–0.65 (m, 2H), 0.15 (s, 9H) ppm; **^{13}C{^{1}H} NMR** (101 MHz, $CDCl_3$) δ = 176.5, 145.2, 135.7, 131.8, 131.0, 130.8, 124.9, 40.6, 16.9, 12.8, 12.8, 0.0 ppm; **IR (ATR):** $\tilde{\nu}$ = 3067, 2988, 2956, 2900, 1638, 1583, 1478, 1429, 1394, 1363, 1284, 1250, 1197, 1114, 1081, 1040, 1028, 936, 840, 761, 729, 691, 653, 615, 479 cm^{-1}; **HRMS (ESI)** calculated for $[C_{14}H_{21}{}^{79}BrNOSi]^+$ 326.0570, found 326.0567; ***R*$_f$**: 0.20 (pentane/EtOAc 5/1).

N-(2-bromobenzyl)-*N*-(2-bromophenyl)-1-methylcyclopropanecarboxamide (**1p**):

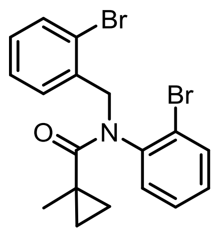

^{1}H NMR (400 MHz, $CDCl_3$) δ = 7.71–7.62 (m, 1H), 7.43 (dd, *J* = 8.0, 1.1 Hz, 1H), 7.39 (dd, *J* = 7.7, 1.6 Hz, 1H), 7.21 (td, *J* = 7.5, 1.2 Hz, 1H), 7.19–7.11 (m, 2H), 7.07 (td, *J* = 7.7, 1.7 Hz, 1H), 6.94–6.88 (m, 1H), 5.65 (d, *J* = 14.8 Hz, 1H), 4.45 (d, *J* = 13.9 Hz, 1H), 1.37–1.28 (m, 1H), 1.11–0.79 (m, 4H), 0.52–0.21 (m, 2H) ppm; **^{13}C{^{1}H} NMR** (101 MHz, $CDCl_3$) δ = 174.2, 140.4, 136.4, 133.6, 132.6, 132.2, 131.1, 129.4, 129.0, 127.7, 127.5, 124.5, 124.4, 51.6, 21.8, 21.3, 16.1, 14.9 ppm; **IR (ATR):** $\tilde{\nu}$ = 3064, 3007, 2978, 2963, 2874, 1649, 1584, 1473, 1439, 1388, 1321, 1278, 1227, 1191, 1028, 988, 766, 747, 732, 624, 578, 451 cm^{-1}; **HRMS (ESI)** calculated for $[C_{18}H_{18}{}^{79}Br_2NO]^+$ 421.9750, found 421.9750; ***R*$_f$**: 0.45 (pentane/EtOAc 5/1); **m.p.**: 89-90 °C.

General procedure for the enantioselective synthesis of cyclopropane containing dihydroquinolones (2):

$Pd(dba)_2$ (1.15 mg, 2.00 µmol, 2.0 mol%), **L2** (2.60 mg, 4.00 µmol, 4.0 mol%), *N*-(2-bromoaryl)cyclopropanecarboxamide (**1**, 100 µmol), PivOH (3.07 mg, 30.0 µmol, 30 mol%) and Cs_2CO_3 (49.0 mg, 0.15 mmol, 1.5 eq.) were weighed into a vial equipped with a magnetic stirring bar. The vial was sealed with a rubber septum, evacuated and then backfilled with nitrogen three times. Mesitylene (330 µL, 0.30 M) was added. The reaction mixture was degassed by three freeze-pump-thaw cycles and then stirred at 130 °C. After 12 h, the reaction mixture was cooled to rt and directly purified by chromatography column on silica gel to afford the desired dihydroquinolone **2**.

(1a*R*,7b*R*)-3-ethyl-1a-methyl-3,7b-dihydro-1*H*-cyclopropa[c]quinolin-2(1a*H*)-one (**2a**):

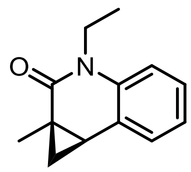

95% isol. yield. ^{1}**H NMR** (400 MHz, $CDCl_3$) δ = 7.33 (dd, *J* = 7.4, 1.6 Hz, 1H), 7.21 (ddd, *J* = 8.3, 7.5, 1.6 Hz, 1H), 7.01 (td, *J* = 7.4, 1.0 Hz, 1H), 6.94 (d, *J* = 8.3 Hz, 1H), 4.03 (dq, *J* = 14.2, 7.1 Hz, 1H), 3.93 (dq, *J* = 14.2, 7.1 Hz, 1H), 2.35 (dd, *J* = 8.5, 5.1 Hz, 1H), 1.48 (s, 3H), 1.36 (dd, *J* = 8.6, 4.3 Hz, 1H), 1.23 (t, *J* = 7.1 Hz, 3H), 0.70 (t, *J* = 4.7 Hz, 1H) ppm; ^{13}**C{^{1}H} NMR** (101 MHz, $CDCl_3$) δ = 170.8, 136.0, 127.0, 126.7, 125.0, 122.0, 114.1, 37.1, 27.8, 24.2, 20.5, 19.1, 12.3 ppm; **IR (ATR):** $\tilde{\nu}$ = 2971, 2932, 1650, 1601, 1501, 1465, 1448, 1395, 1371, 1330, 1303, 1263, 1206, 1135, 1116, 1091, 1015, 910, 820, 792, 749, 679, 485, 452 cm^{-1}; **HRMS (ESI)** calculated for $[C_{13}H_{16}NO]^+$ 202.1225, found 202.1226; ***R*$_f$**: 0.40 (pentane/EtOAc 5/1); **[α]$_D^{20}$** = +78.5 (*c* = 1.0, $CHCl_3$). Chiral HPLC: (Chiralpak ID, 4.6 x 250 mm; 2% *i*-PrOH / hexane, 1.0 mL/min, 254 nm; t_r (minor) = 14.1 min, t_r (major) = 15.5 min), 98.0/2.0 *er.*

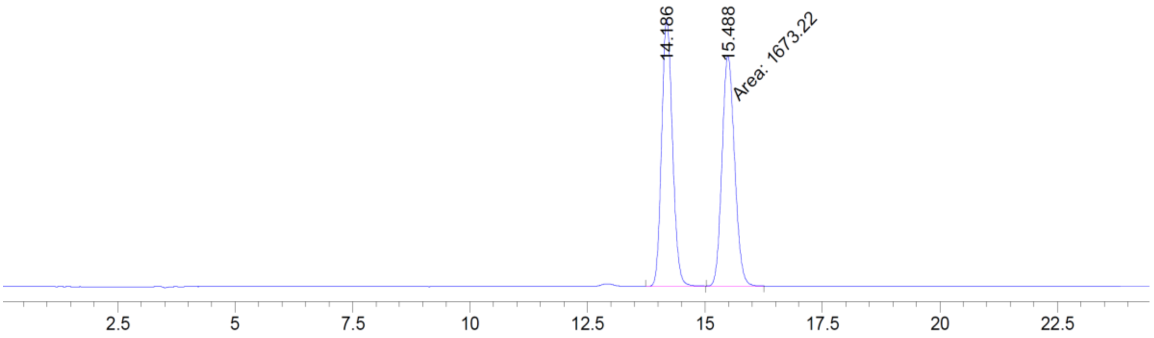

Peak #	RetTime [min]	Type	Width [min]	Area [mAU*s]	Height [mAU]	Area %
1	14.186	BB	0.2493	1669.97644	104.02184	49.9515
2	15.488	MF	0.3115	1673.21606	89.52665	50.0485

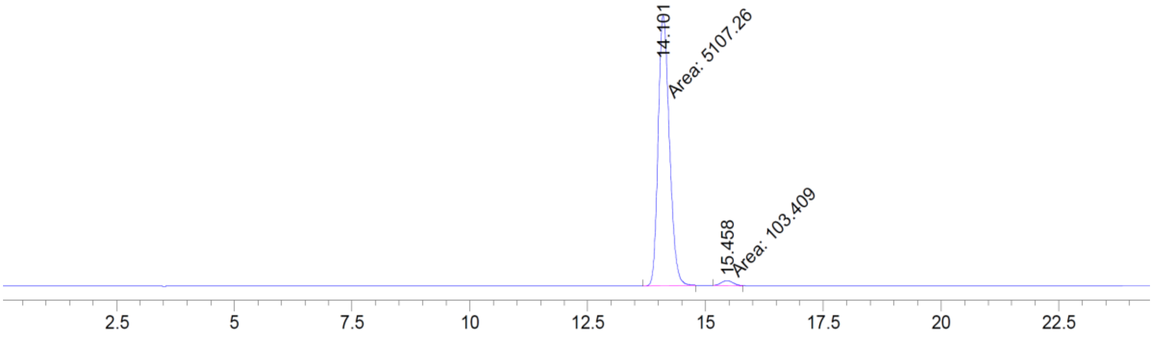

#	[min]		[min]	[mAU*s]	[mAU]	%
1	14.101	MF	0.2759	5107.26367	308.49371	98.0154
2	15.458	FM	0.3030	103.40873	5.68769	1.9846

(1a*S*,7b*R*)-1a-benzyl-3-methyl-3,7b-dihydro-1*H*-cyclopropa[c]quinolin-2(1a*H*)-one (**2b**):

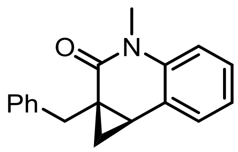

using [(η^3-cinnamyl)Pd(Cp)] as the palladium source, 97% isol. yield. **^{1}H NMR** (400 MHz, $CDCl_3$) δ = 7.33–7.16 (m, 7H), 7.02 (td, *J* = 7.4, 1.0 Hz, 1H), 6.93 (d, *J* = 8.2 Hz, 1H), 3.77 (d, *J* = 14.7 Hz, 1H), 3.34 (s, 3H), 2.73 (d, *J* = 14.7 Hz, 1H), 2.42 (dd, *J* = 8.7, 5.3 Hz, 1H), 1.42 (dd, *J* = 8.8, 4.5 Hz, 1H), 0.78 (dd, *J* = 5.2, 4.6 Hz, 1H) ppm; **^{13}C{^{1}H} NMR** (101 MHz, $CDCl_3$) δ = 170.3, 138.4, 137.3, 129.5, 128.3, 127.9, 126.8, 126.4, 124.3, 122.3, 114.2, 38.9, 29.8, 28.8, 25.5, 17.2 ppm; **IR (ATR):** $\tilde{\nu}$ = 3060, 3027, 2939, 2917, 1655, 1601, 1504, 1495, 1474, 1454, 1417, 1365, 1331, 1303, 1268, 1223, 1132, 1105, 1076, 1046, 1018, 912, 750, 700, 680, 630, 599, 570, 528, 488 cm^{-1}; **HRMS (ESI)** calculated for $[C_{18}H_{18}NO]^+$ 264.1383, found 264.1373; ***R*$_f$**: 0.50 (pentane/EtOAc 3/1); **$[\alpha]_D^{20}$** = +7.8 (*c* = 1.0, $CHCl_3$). Chiral HPLC: (Chiralpak ID, 4.6 x 250 mm; 10% *i*-PrOH / hexane, 1.0 mL/min, 214 nm; t_r (minor) = 12.4 min, t_r (major) = 14.4 min), 96.9/3.1 *er.*

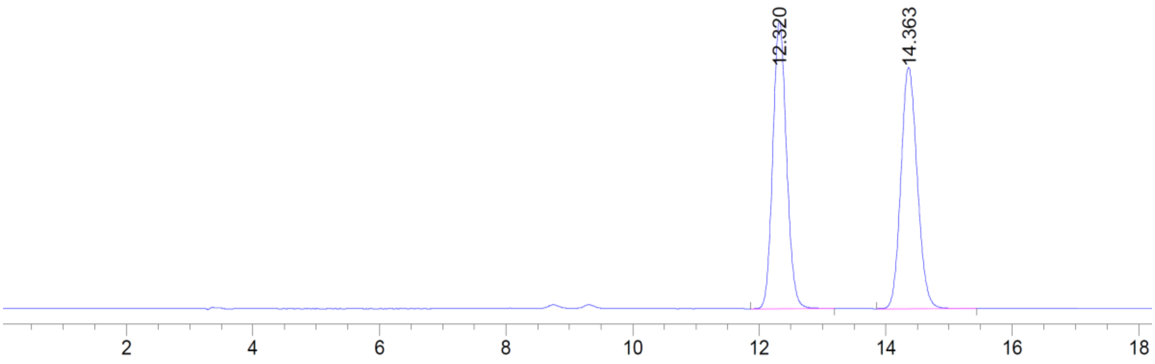

Peak #	RetTime [min]	Type	Width [min]	Area [mAU*s]	Height [mAU]	Area %
1	12.320	BB	0.2339	1008.37000	66.89353	49.9834
2	14.363	BB	0.2777	1009.04083	56.13845	50.0166

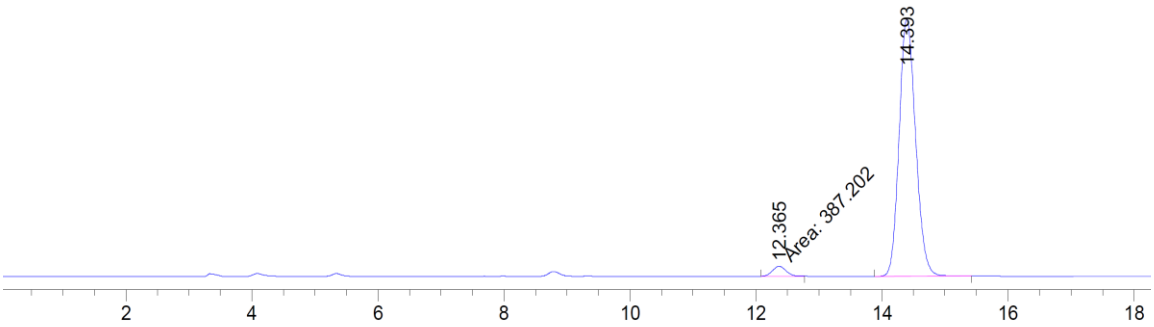

Peak #	RetTime [min]	Type	Width [min]	Area [mAU*s]	Height [mAU]	Area %
1	12.365	FM	0.2496	387.20151	25.85750	3.1477
2	14.393	BB	0.2815	1.19140e4	657.12177	96.8523

(1a*R*,7b*R*)-3-methyl-1a-phenyl-3,7b-dihydro-1*H*-cyclopropa[c]quinolin-2(1a*H*)-one (**2c**):

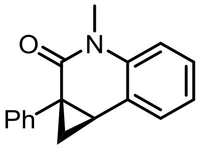

using [(η^3-cinnamyl)Pd(Cp)] as the palladium source, 90% isol. yield. **^{1}H NMR** (400 MHz, $CDCl_3$) δ = 7.46–7.26 (m, 7H), 7.09 (td, *J* = 7.4, 1.0 Hz, 1H), 7.02 (d, *J* = 8.2 Hz, 1H), 3.39 (s, 3H), 2.77 (dd, *J* = 9.0, 5.5 Hz, 1H), 2.00 (dd, *J* = 9.0, 4.5 Hz, 1H), 1.15 (dd, *J* = 5.4, 4.6 Hz, 1H) ppm; **^{13}C{^{1}H} NMR** (101 MHz, $CDCl_3$) δ = 169.6, 139.7, 137.4, 130.2, 128.4, 127.9, 127.5, 127.1, 124.1, 122.5, 114.3, 33.7, 29.9, 28.0, 18.0 ppm; **IR (ATR):** $\tilde{\nu}$ = 3057, 3027, 1970, 1940, 2888, 1656, 1600, 1501, 1473, 1446, 1416, 1391, 1350, 1309, 1229, 1134, 1102, 1044, 1006, 940, 776, 751, 698, 680, 645, 604, 580, 548, 517, 482 cm^{-1}; **HRMS (ESI)** calculated for $[C_{17}H_{16}NO]^+$ 250.1226, found 250.1227; ***R*$_f$**: 0.25 (pentane/EtOAc 5/1); **$[\alpha]_D^{20}$** = +44.5 (*c* = 1.0, $CHCl_3$). Chiral HPLC: (Chiralpak ID, 4.6 x 250 mm; 5% *i*-PrOH / hexane, 1.0 mL/min, 254 nm; t_r (minor) = 26.1 min, t_r (major) = 30.8 min), 95.3/4.7 *er.*

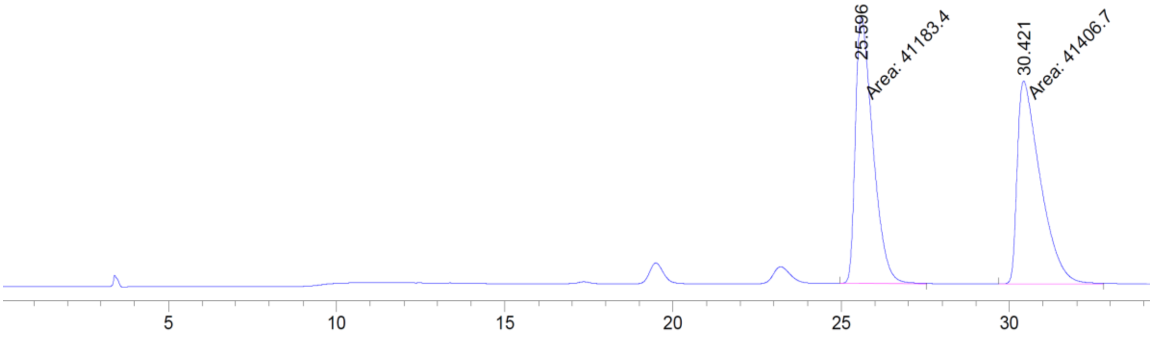

Peak #	RetTime [min]	Type	Width [min]	Area [mAU*s]	Height [mAU]	Area %
1	25.596	MF	0.6023	4.11834e4	1139.64990	49.8648
2	30.421	MF	0.7947	4.14067e4	868.41400	50.1352

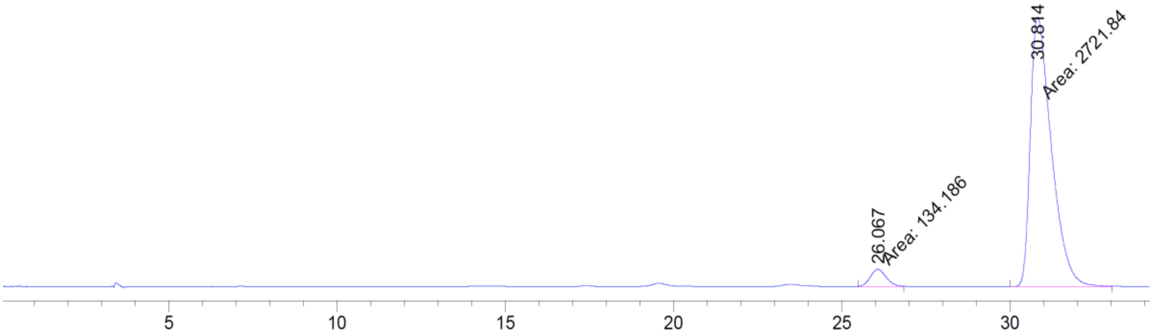

Peak #	RetTime [min]	Type	Width [min]	Area [mAU*s]	Height [mAU]	Area %
1	26.067	MF	0.5546	134.18636	4.03235	4.6984
2	30.814	MF	0.7241	2721.84180	62.64500	95.3016

(1a*R*,7b*R*)-1a,3-dimethyl-3,7b-dihydro-1*H*-cyclopropa[c]quinolin-2(1a*H*)-one (**2d**):

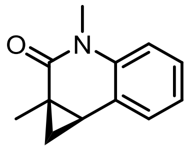

90% isol. yield. **^{1}H NMR** (400 MHz, $CDCl_3$) δ = 7.33 (dd, *J* = 7.4, 1.6 Hz, 1H), 7.22 (ddd, *J* = 8.2, 7.5, 1.6 Hz, 1H), 7.03 (td, *J* = 7.4, 1.0 Hz, 1H), 6.93 (d, *J* = 8.2 Hz, 1H), 3.34 (s, 3H), 2.38 (dd, *J* = 8.6, 5.2 Hz, 1H), 1.48 (s, 3H), 1.38 (dd, *J* = 8.6, 4.4 Hz, 1H), 0.76 (t, *J* = 4.8 Hz, 1H) ppm; **^{13}C{^{1}H} NMR** (101 MHz, $CDCl_3$) δ = 171.3, 137.4, 127.6, 126.7, 124.7, 122.3, 114.1, 29.7, 28.0, 24.3, 20.5, 19.3 ppm; **IR (ATR):** $\tilde{\nu}$ = 2968, 2931, 2899, 1655, 1601, 1504, n1474, 1417, 1370, 1350, 1303, 1261, 1227, 1132, 1110, 1040, 1006, 910, 750, 680, 487, 449 cm^{-1}; **HRMS (ESI)** calculated for $[C_{12}H_{14}NO]^+$ 188.1070, found 188.1069; ***R*$_f$**: 0.30 (pentane/EtOAc 5/1); **$[\alpha]_D^{20}$** = +88.8 (*c* = 1.0, $CHCl_3$). Chiral HPLC: (Chiralpak ID, 4.6 x 250 mm; 2% *i*-PrOH / hexane, 1.0 mL/min, 254 nm; t_r (minor) = 23.3 min, t_r (major) = 19.9 min), 97.6/2.4 *er.*

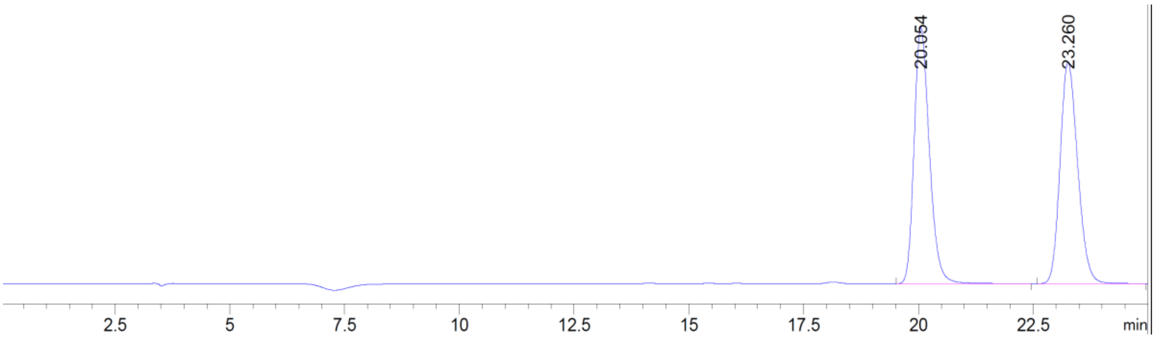

Peak #	RetTime [min]	Type	Width [min]	Area [mAU*s]	Height [mAU]	Area %
1	20.054	BB	0.3567	1554.40320	67.00037	50.1697
2	23.260	BBA	0.4136	1543.88696	57.80753	49.8303

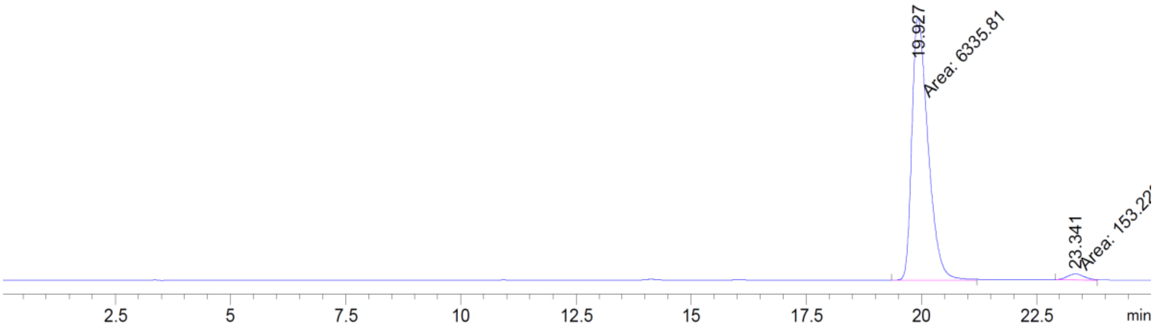

Peak #	RetTime [min]	Type	Width [min]	Area [mAU*s]	Height [mAU]	Area %
1	19.927	MF	0.4054	6335.81104	260.50760	97.6388
2	23.341	MF	0.4321	153.22151	5.91039	2.3612

<u>(1a*R*,7b*R*)-1a-methyl-3-propyl-3,7b-dihydro-1*H*-cyclopropa[c]quinolin-2(1a*H*)-one (**2e**):</u>

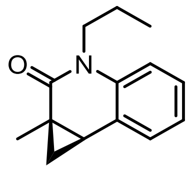

92% isol. yield. **^{1}H NMR** (400 MHz, $CDCl_3$) δ = 7.34 (d, *J* = 7.4 Hz, 1H), 7.22 (t, *J* = 7.9 Hz, 1H), 7.02 (t, *J* = 7.4 Hz, 1H), 6.92 (d, *J* = 8.3 Hz, 1H), 4.03–3.90 (m, 1H), 3.88–3.79 (m, 1H), 2.36 (dd, *J* = 8.4, 5.1 Hz, 1H), 1.78–1.59 (m, 2H), 1.49 (s, 3H), 1.38 (dd, *J* = 8.5, 4.3 Hz, 1H), 0.99 (t, *J* = 7.4 Hz, 3H), 0.71 (t, *J* = 4.7 Hz, 1H) ppm; **^{13}C{^{1}H} NMR** (101 MHz, $CDCl_3$) δ = 171.0, 136.3, 127.9, 126.6, 125.0, 122.0, 114.2, 43.6, 27.8, 24.2, 20.5, 20.2, 19.1, 11.3 ppm; **IR (ATR):** $\tilde{\nu}$ = 2963, 2932, 2875, 1657, 1602, 1500, 1468, 1395, 1369, 1338, 1302, 1258, 1241, 1201, 1138, 1120, 941, 749, 680 cm^{-1}; **HRMS (ESI)** calculated for $[C_{14}H_{18}NO]^+$: 216.1383, found: 216.1365; ***R*$_f$**: 0.75 (pentane/EtOAc 5/1); **$[\alpha]_D^{20}$** = +59.5 (*c* = 1.0, $CHCl_3$). Chiral HPLC: (Chiralpak AYH, 4.6 x 250 mm; 2% *i*-PrOH / hexane, 1.0 mL/min, 254 nm; t_r (minor) = 9.6 min, t_r (major) = 11.8 min), 97.9/2.1 *er.*

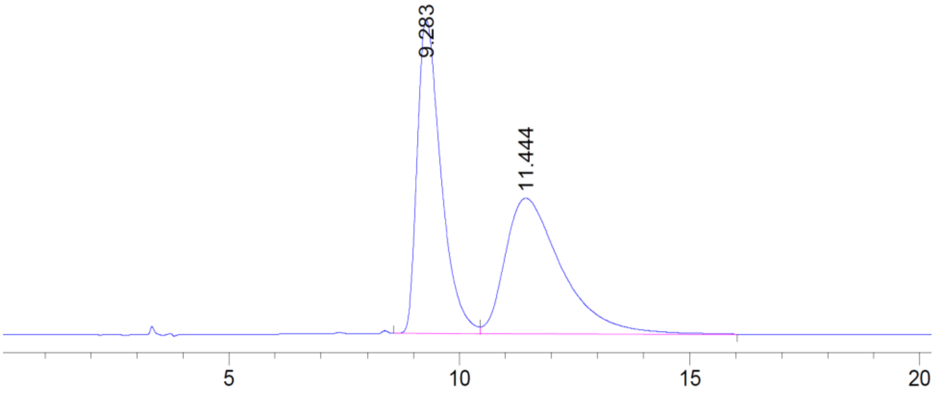

Peak #	RetTime [min]	Type	Width [min]	Area [mAU*s]	Height [mAU]	Area %
1	9.283	BV	0.5488	1.38889e4	385.04712	49.5869
2	11.444	VB	1.2855	1.41204e4	166.14925	50.4131

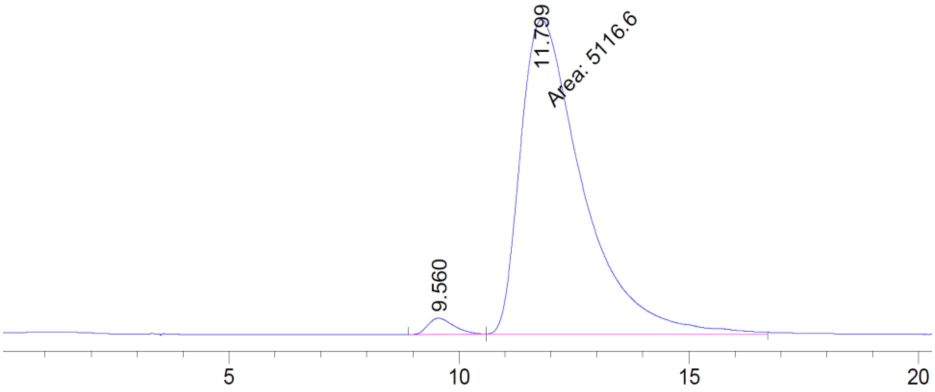

Peak #	RetTime [min]	Type	Width [min]	Area [mAU*s]	Height [mAU]	Area %
1	9.560	BB	0.5542	107.63626	2.78949	2.0603
2	11.799	MF	1.5662	5116.60059	54.44907	97.9397

(1a*R*,7b*R*)-3-(4-methoxybenzyl)-1a-methyl-3,7b-dihydro-1*H*-cyclopropa[c]quinolin-2(1a*H*)-one (**2f**): using [(η^3-cinnamyl)Pd(Cp)] as the palladium source, 99% isol. yield. **^{1}H NMR** (400 MHz, $CDCl_3$) δ = 7.31 (d, *J* = 7.4 Hz, 1H), 7.16–7.11 (m, 2H), 7.05 (t, *J* = 7.8 Hz, 1H), 6.96 (t, *J* = 7.4 Hz, 1H), 6.87–6.81 (m, 3H), 5.27 (d, *J* = 14.2 Hz, 1H), 4.99 (d, *J* = 15.4 Hz, 1H), 3.77 (s, 3H), 2.42 (dd, *J* = 8.5, 5.1 Hz, 1H), 1.55 (s, 3H), 1.44 (dd, *J* = 8.5, 4.4 Hz, 1H), 0.82 (t, J = 4.7 Hz, 1H) ppm; **^{13}C{^{1}H} NMR** (101 MHz, $CDCl_3$) δ = 171.5, 158.6, 136.4, 129.1, 127.7, 127.7, 126.6, 124.9, 122.3, 115.2, 114.1, 55.3, 45.2, 27.9, 24.3, 20.7, 19.4 ppm; **IR (ATR):** $\tilde{\nu}$ = 2997, 2966, 2932, 2904, 2835, 1655, 1603, 1585, 1512, 1469, 1395, 1368, 1333, 1302, 1248, 1182, 1125, 1111, 1033, 997, 889, 829, 808, 751, 680, 654, 630, 595, 570, 533, 509, 448 cm^{-1}; **HRMS (ESI)** calculated for $[C_{19}H_{20}NO_2]^+$ 294.1489, found 294.1492; ***R*$_f$**: 0.65 (pentane/EtOAc 3/1); **$[\alpha]_D^{20}$** = +14.3 (*c* = 1.0, $CHCl_3$). Chiral HPLC: (Chiralpak IB, 4.6 x 250 mm; 10% *i*-PrOH / hexane, 1.0 mL/min, 254 nm; t_r (minor) = 10.4 min, t_r (major) = 9.6 min), 97.7/2.3 *er.*

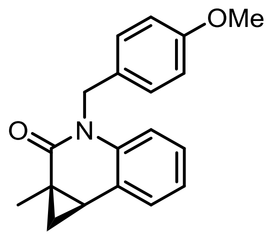

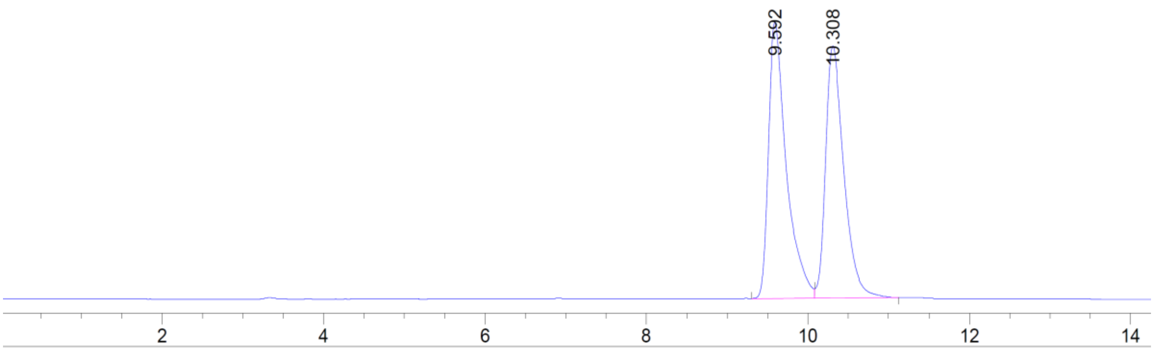

Peak #	RetTime [min]	Type	Width [min]	Area [mAU*s]	Height [mAU]	Area %
1	9.592	BV	0.2306	4813.09619	310.95715	51.8789
2	10.308	VB	0.2373	4464.45996	284.10776	48.1211

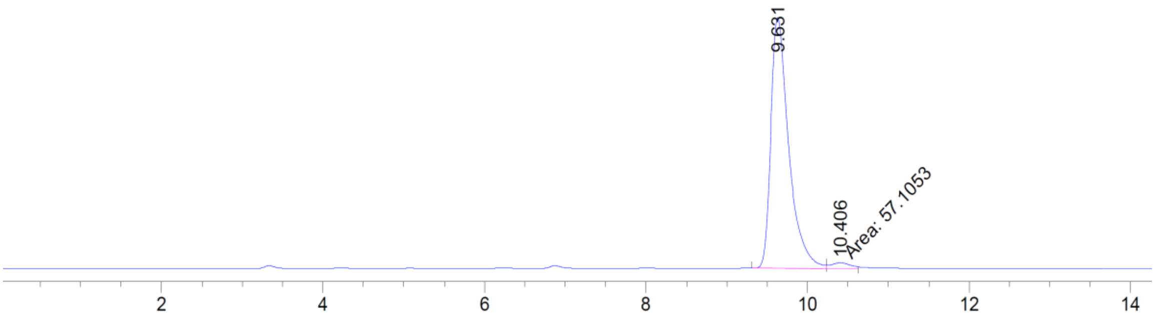

Peak #	RetTime [min]	Type	Width [min]	Area [mAU*s]	Height [mAU]	Area %
1	9.631	BV	0.2288	2412.10107	157.37639	97.6873
2	10.406	MF	0.2755	57.10527	3.45484	2.3127

(1a*R*,7b*R*)-3-(2-methoxybenzyl)-1a-methyl-3,7b-dihydro-1*H*-cyclopropa[c]quinolin-2(1a*H*)-one (**2g**): using [(η³-cinnamyl)Pd(Cp)] as the palladium source, 97% isol. yield. **^{1}H NMR** (400 MHz, $CDCl_3$) δ = 7.32 (dd, *J* = 7.4, 1.6 Hz, 1H), 7.20 (ddd, *J* = 8.8, 7.1, 2.1 Hz, 1H), 7.05 (ddd, *J* = 8.2, 7.5, 1.7 Hz, 1H), 6.96 (td, *J* = 7.4, 1.1 Hz, 1H), 6.90 (d, *J* = 8.0 Hz, 1H), 6.88–6.79 (m, 2H), 6.75 (d, *J* = 8.2 Hz, 1H), 5.32 (d, *J* = 17.0 Hz, 1H), 5.05 (d, *J* = 17.1 Hz, 1H), 3.93 (s, 3H), 2.44 (dd, *J* = 8.5, 5.1 Hz, 1H), 1.56 (s, 3H), 1.46 (dd, *J* = 8.5, 4.4 Hz, 1H), 0.86 (t, *J* = 4.7 Hz, 1H) ppm; **^{13}C{^{1}H} NMR** (101 MHz, $CDCl_3$) δ = 171.5, 156.7, 136.4, 127.9, 127.6, 126.7, 126.6, 124.8, 124.7, 122.2, 120.7, 115.3, 110.2, 55.4, 40.7, 27.9, 24.4, 20.7, 19.5 ppm; **IR (ATR):** $\tilde{\nu}$ = 3110, 3076, 3036, 2997, 2963, 2934, 2837, 1658, 1602, 1588, 1492, 1462, 1439, 1395, 1366, 1334, 1302, 1285, 1242, 1188, 1164, 1125, 1111, 1050, 1028, 999, 749, 679, 498, 452 cm^{-1}; **HRMS (ESI)** calculated for $[C_{19}H_{20}NO_2]^+$ 294.1489, found 294.1485; ***R*$_f$**: 0.25 (pentane/EtOAc 5/1); **$[\alpha]_D^{20}$** = +19.8 (*c* = 1.0, $CHCl_3$). Chiral HPLC: (Chiralpak IB, 4.6 x 250 mm; 10% *i*-PrOH / hexane, 1.0 mL/min, 254 nm; t_r (minor) = 11.7 min, t_r (major) = 9.0 min), 98.2/1.8 *er.*

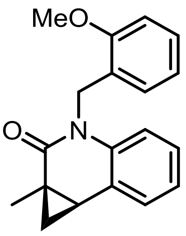

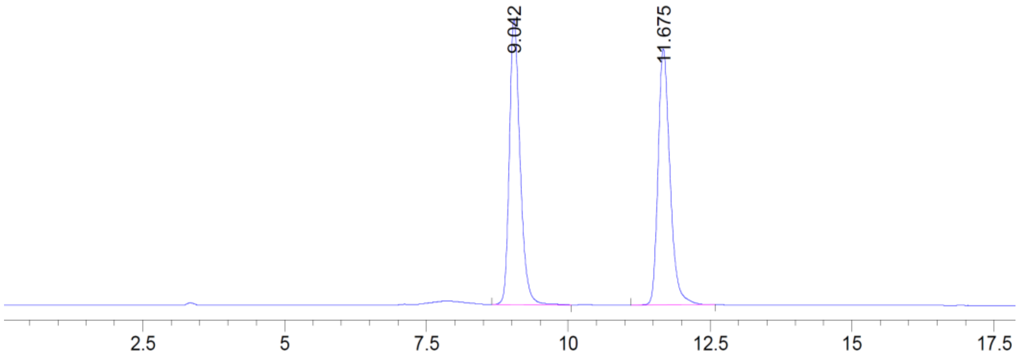

Peak #	RetTime [min]	Type	Width [min]	Area [mAU*s]	Height [mAU]	Area %
1	9.042	BB	0.2013	1618.10132	123.18160	50.0236
2	11.675	BB	0.2228	1616.57471	110.42328	49.9764

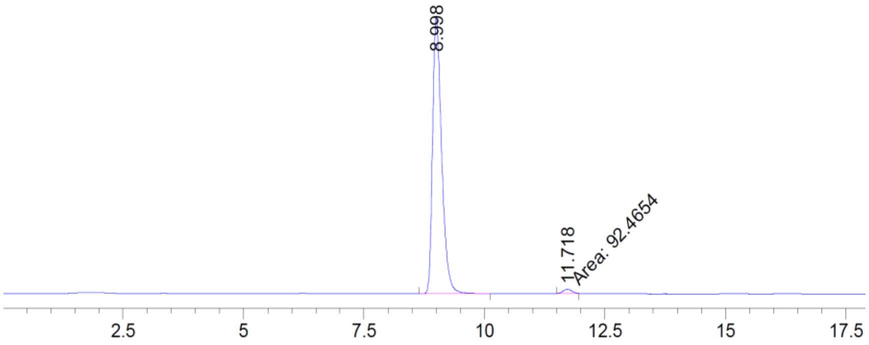

Peak #	RetTime [min]	Type	Width [min]	Area [mAU*s]	Height [mAU]	Area %
1	8.998	BB	0.2000	5145.54395	389.76895	98.2347
2	11.718	MF	0.2372	92.46542	6.49593	1.7653

(1a*R*,7b*R*)-1a,3-dimethyl-6-(trifluoromethoxy)-3,7b-dihydro-1*H*-cyclopropa[c]quinolin-2(1a*H*)-one (**2i**): using [(η^3-cinnamyl)Pd(Cp)] as the palladium source, 86% isol. yield. **^{1}H NMR** (400 MHz, $CDCl_3$) δ = 7.20 (d, *J* = 2.2 Hz, 1H), 7.07 (ddd, *J* = 9.0, 2.7, 0.8 Hz, 1H), 6.90 (d, *J* = 9.0 Hz, 1H), 3.33 (s, 3H), 2.36 (dd, *J* = 8.6, 5.1 Hz, 1H), 1.48 (s, 3H), 1.43 (dd, *J* = 8.6, 4.6 Hz, 1H), 0.80 (t, *J* = 4.8 Hz, 1H) ppm; **^{13}C{^{1}H} NMR** (101 MHz, $CDCl_3$) δ = 170.9, 144.0 (q, $J_{C\text{-}F}$ = 1.9 Hz), 136.2, 126.3, 120.5 (q, $J_{C\text{-}F}$ = 256.3 Hz), 120.5, 119.2, 114.9, 29.9, 27.7, 24.5, 20.3, 19.4 ppm; **IR (ATR):** $\tilde{\nu}$ = 2970, 2933, 2902, 1662, 1611, 1507, 1472, 1419, 1370, 1356, 1251, 1158, 1109, 981, 924, 883, 861, 809, 761, 703, 666, 564, 538, 489, 476, 460 cm^{-1}; **HRMS (ESI)** calculated for $[C_{13}H_{13}F_3NO_2]^+$ 272.0893, found 272.0898; ***R*$_f$**: 0.20 (pentane/EtOAc 5/1); **$[\alpha]_D^{20}$** = +65.2 (*c* = 1.0, $CHCl_3$). Chiral HPLC: (Chiralpak IB, 4.6 x 250 mm; 5% *i*-PrOH / hexane, 1.0 mL/min, 254 nm; t_r (minor) = 8.2 min, t_r (major) = 8.7 min), 95.1/4.9 *er.*

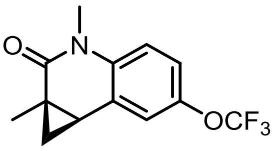

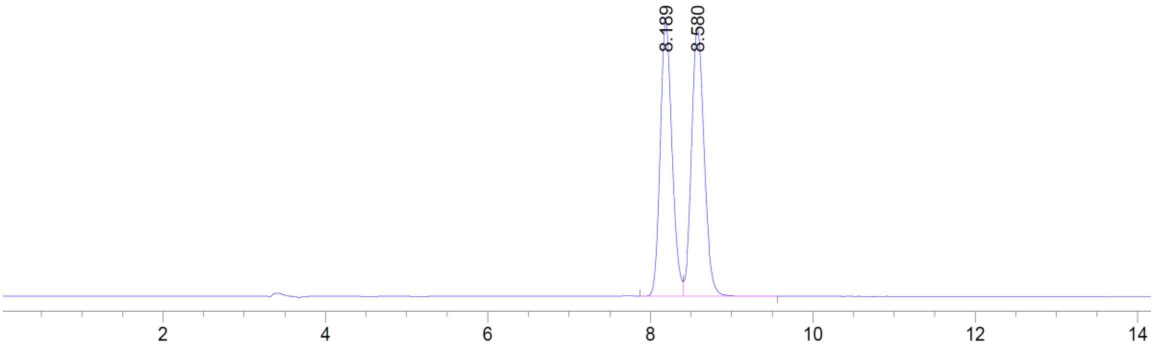

Peak #	RetTime [min]	Type	Width [min]	Area [mAU*s]	Height [mAU]	Area %
1	8.189	BV	0.1565	875.91199	86.26186	49.4428
2	8.580	VB	0.1651	895.65448	83.50131	50.5572

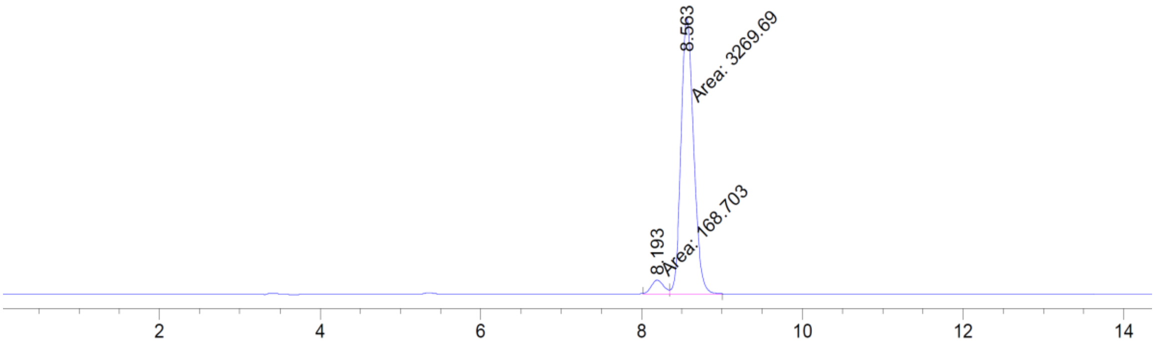

Peak #	RetTime [min]	Type	Width [min]	Area [mAU*s]	Height [mAU]	Area %
1	8.193	FM	0.1835	168.70302	15.32128	4.9064
2	8.563	MF	0.1808	3269.69360	301.38364	95.0936

(1a*R*,7b*R*)-1a,3,6-trimethyl-3,7b-dihydro-1*H*-cyclopropa[c]quinolin-2(1a*H*)-one (**2j**):

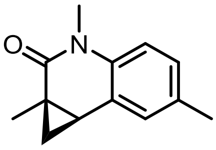

using [(η^3-cinnamyl)Pd(Cp)] as the palladium source, 94% isol. yield. **^{1}H NMR** (400 MHz, $CDCl_3$) δ = 7.14 (d, *J* = 1.8 Hz, 1H), 7.01 (dd, *J* = 8.6, 1.7 Hz, 1H), 6.82 (d, *J* = 8.3 Hz, 1H), 3.32 (s, 3H), 2.32 (s, 3H), 2.32 (dd, *J* = 8.2, 5.2 Hz, 1H), 1.47 (s, 3H), 1.35 (dd, *J* = 8.6, 4.3 Hz, 1H), 0.73 (t, *J* = 4.7 Hz, 1H) ppm; **^{13}C{^{1}H} NMR** (101 MHz, $CDCl_3$) δ = 171.2, 135.1, 131.8, 128.3, 127.1, 124.6, 114.0, 29.7, 27.9, 24.2, 20.5, 20.4, 19.4 ppm; **IR (ATR):** $\tilde{\nu}$ = 2967, 2929, 2902, 1655, 1617, 1590, 1509, 1459, 1416, 1360, 1319, 1299, 1269, 1229, 1108, 1011, 910, 889, 807, 760, 633, 533, 483, 442, 429, 405 cm^{-1}; **HRMS (ESI)** calculated for $[C_{13}H_{16}NO]^+$: 202.1226, found: 202.1232; ***R*$_f$**: 0.30 (pentane/EtOAc 5/1); **$[\alpha]_D^{20}$** = +92.8 (*c* = 1.0, $CHCl_3$). Chiral HPLC: (Chiralpak IB, 4.6 x 250 mm; 10% *i*-PrOH / hexane, 1.0 mL/min, 254 nm; t_r (minor) = 15.3 min, t_r (major) = 14.5 min), 96.2/3.8 *er*.

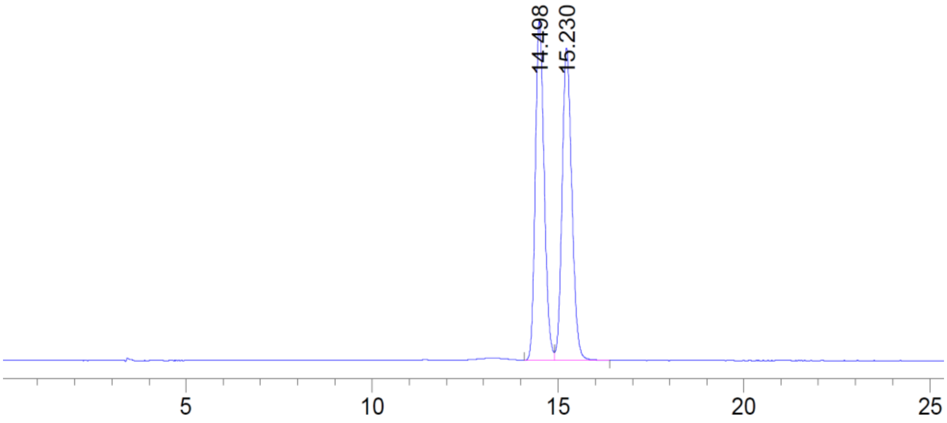

Peak #	RetTime [min]	Type	Width [min]	Area [mAU*s]	Height [mAU]	Area %
1	14.498	BV	0.2551	1705.64050	103.03926	49.6396
2	15.230	VB	0.2826	1730.40906	94.97816	50.3604

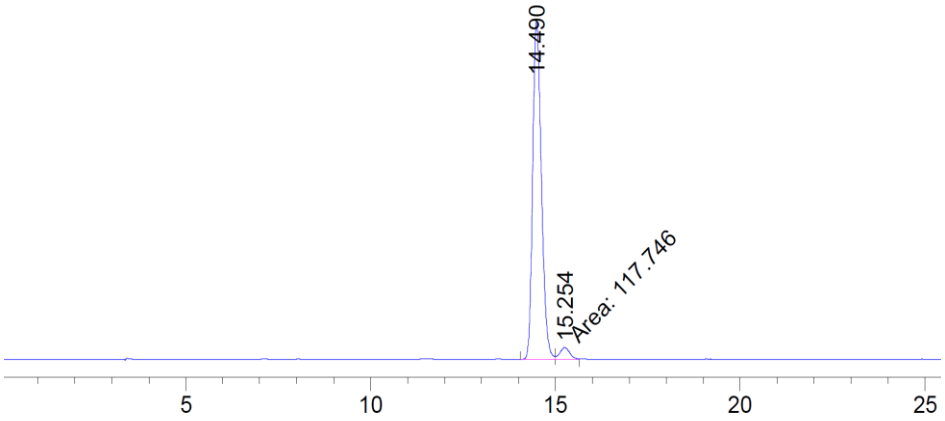

Peak #	RetTime [min]	Type	Width [min]	Area [mAU*s]	Height [mAU]	Area %
1	14.490	BV	0.2531	3009.95142	183.76501	96.2354
2	15.254	MF	0.3090	117.74577	6.34993	3.7646

(1a*R*,7b*R*)-1a,3-dimethyl-5-(trifluoromethyl)-3,7b-dihydro-1*H*-cyclopropa[c]quinolin-2(1a*H*)-one (**2k**):

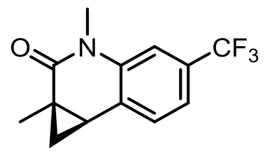

using [(η^3-cinnamyl)Pd(Cp)] as the palladium source, 85% isol. yield. **^{1}H NMR** (400 MHz, $CDCl_3$) δ = 7.43 (d, *J* = 7.8 Hz, 1H), 7.31–7.26 (m, 1H), 7.12 (s, 1H), 3.37 (s, 3H), 2.43 (dd, *J* = 8.7, 5.1 Hz, 1H), 1.50 (s, 3H), 1.47 (dd, *J* = 8.7, 4.5 Hz, 1H), 0.81 (t, *J* = 4.8 Hz, 1H) ppm; **^{13}C{^{1}H} NMR** (101 MHz, $CDCl_3$) δ = 171.0, 137.9, 129.1 (q, $J_{C\text{-}F}$ = 32.5 Hz), 128.6 (q, $J_{C\text{-}F}$ = 1.4 Hz), 128.0, 124.1 (q, $J_{C\text{-}F}$ = 272.0 Hz), 118.9 (q, $J_{C\text{-}F}$ = 3.9 Hz), 110.8 (q, $J_{C\text{-}F}$ = 3.9 Hz), 29.9, 27.8, 24.8, 20.3, 19.5 ppm; **IR (ATR):** $\tilde{\nu}$ = 2972, 2936, 2901, 1716, 1667, 1617, 1591, 1558, 1540, 1520, 1507, 1472, 1456, 1436, 1338, 1354, 1337, 1313, 1295, 1258, 1228, 1167, 1114, 1084, 1049, 1006, 928, 867, 823, 788, 700, 662, 649, 568, 493, 459, 418 cm^{-1}; **HRMS (ESI)** calculated for $[C_{13}H_{13}F_3NO]^+$ 256.0944, found 256.0951; ***R*$_f$**: 0.25 (pentane/EtOAc 5/1); **m.p.**: 73-74 °C; **$[\alpha]_D^{20}$** = +47.6 (*c* = 1.0, $CHCl_3$). Chiral HPLC: (Chiralpak IB, 4.6 x 250 mm; 5% *i*-PrOH / hexane, 1.0 mL/min, 294 nm; t_r (minor) = 7.3 min, t_r (major) = 7.6 min), 96.5/3.5 *er.*

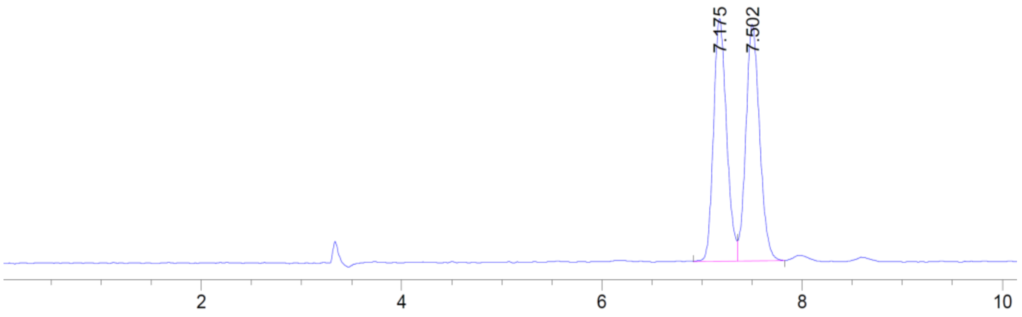

Peak #	RetTime [min]	Type	Width [min]	Area [mAU*s]	Height [mAU]	Area %
1	7.175	BV	0.1398	109.83360	12.11559	49.2659
2	7.502	VB	0.1477	113.10685	11.81476	50.7341

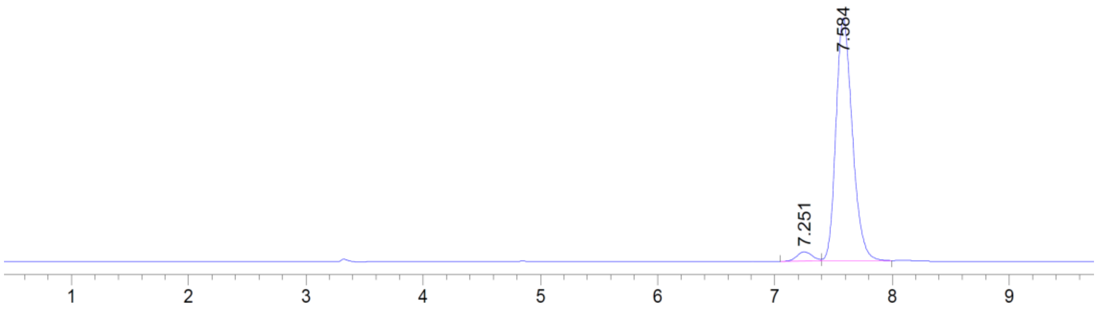

Peak #	RetTime [min]	Type	Width [min]	Area [mAU*s]	Height [mAU]	Area %
1	7.251	BV	0.1433	34.35759	3.73561	3.5093
2	7.584	VB	0.1495	944.69623	97.11185	96.4907

(1a*R*,7b*R*)-1a,3,5-trimethyl-3,7b-dihydro-1*H*-cyclopropa[c]quinolin-2(1a*H*)-one (**2l**):

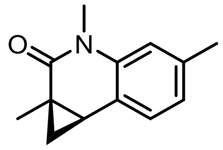

using [(η^3-cinnamyl)Pd(Cp)] as the palladium source, 94% isol. yield. **^{1}H NMR** (400 MHz, $CDCl_3$) δ = 7.20 (d, *J* = 7.6 Hz, 1H), 6.86–6.82 (m, 1H), 6.75 (s, 1H), 3.33 (s, 3H), 2.36 (dd, *J* = 8.5, 5.1 Hz, 1H), 2.34 (m, 1H), 1.47 (s, 3H), 1.34 (dd, *J* = 8.5, 4.3 Hz, 1H), 0.72 (t, *J* = 4.7 Hz, 1H) ppm; **^{13}C{^{1}H} NMR** (101 MHz, $CDCl_3$) δ = 171.6, 137.4, 136.4, 127.5, 122.9, 121.8, 114.9, 29.7, 27.7, 24.1, 21.5, 20.5, 19.4 ppm; **IR (ATR):** $\tilde{\nu}$ = 3018, 2966, 2930, 1655, 1613, 1584, 1518, 1469, 1418, 1356, 1314, 1300, 1267, 1230, 1110, 1014, 932, 853, 815, 791, 763, 743, 685, 601, 584, 485, 601, 584, 485, 456, 408 cm^{-1}; **HRMS (ESI)** calculated for $[C_{13}H_{16}NO]^+$ 202.1226, found 202.1233; ***R*$_f$**: 0.15 (pentane/EtOAc 5/1); **m.p.**: 92-93 °C; **$[\alpha]_D^{20}$** = +115.2 (*c* = 1.0, $CHCl_3$). Chiral HPLC: (Chiralpak ID, 4.6 x 250 mm; 10% *i*-PrOH / hexane, 1.0 mL/min, 254 nm; t_r (minor) = 10.1 min, t_r (major) = 9.0 min), 96.5/3.5 *er.*

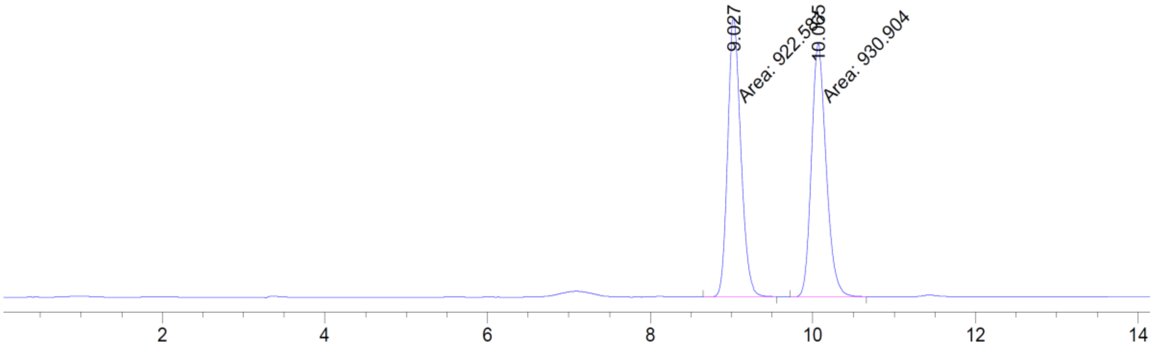

Peak #	RetTime [min]	Type	Width [min]	Area [mAU*s]	Height [mAU]	Area %
1	9.027	MF	0.1892	922.58112	81.25450	49.7755
2	10.065	MF	0.2105	930.90381	73.69472	50.2245

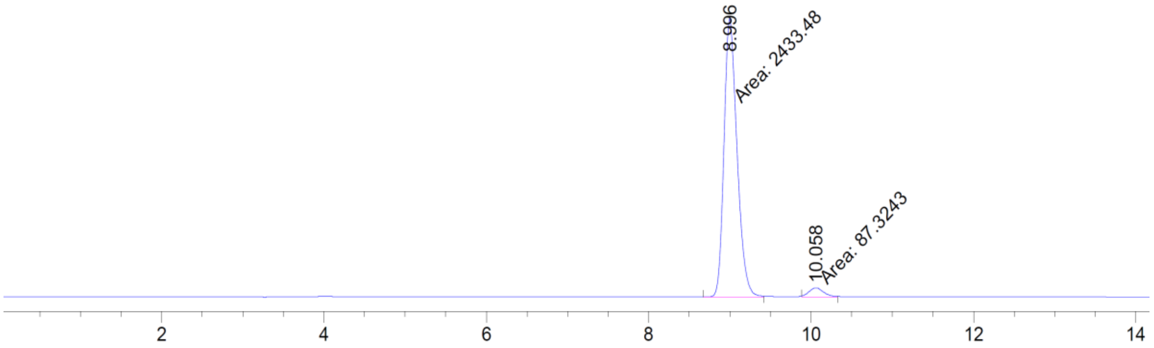

Peak #	RetTime [min]	Type	Width [min]	Area [mAU*s]	Height [mAU]	Area %
1	8.996	FM	0.1873	2433.48218	216.54494	96.5359
2	10.058	FM	0.2085	87.32432	6.98117	3.4641

(1a*R*,7b*R*)-6-chloro-4-fluoro-1a,3-dimethyl-3,7b-dihydro-1*H*-cyclopropa[c]quinolin-2(1a*H*)-one

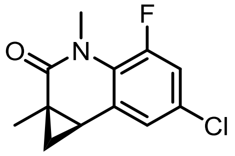

(2m): using [(η[3]-cinnamyl)Pd(Cp)] as the palladium source, 74% isol. yield. **^{1}H NMR** (400 MHz, $CDCl_3$) δ = 7.08 (dd, *J* = 2.3, 1.3 Hz, 1H), 6.96 (dd, *J* = 13.9, 2.4 Hz, 1H), 3.48 (d, *J* = 9.1 Hz, 3H), 2.32 (ddd, *J* = 8.7, 5.1, 1.1 Hz, 1H), 1.46 (s, 3H), 1.41 (dd, *J* = 8.7, 4.5 Hz, 1H), 0.83 (t, *J* = 4.8 Hz, 1H) ppm; **^{13}C{^{1}H} NMR** (101 MHz, $CDCl_3$) δ = 171.6, 150.3 (d, $J_{C\text{-}F}$ = 248.7 Hz), 129.1 (d, $J_{C\text{-}F}$ = 3.3 Hz), 127.4 (d, $J_{C\text{-}F}$ = 11.4 Hz), 124.7 (d, $J_{C\text{-}F}$ = 6.0 Hz), 123.4 (d, $J_{C\text{-}F}$ = 3.1 Hz), 115.8 (d, $J_{C\text{-}F}$ = 28.1 Hz), 34.0 (d, $J_{C\text{-}F}$ = 15.6 Hz), 28.3 (d, $J_{C\text{-}F}$ = 2.9 Hz), 24.7, 20.1, 19.5 ppm; **IR (ATR):** $\tilde{\nu}$ = 3083, 3000, 2967, 2933, 2904, 1666, 1606, 1583, 1485, 1460, 1387, 1374, 1354, 1247, 1194, 1119, 1107, 1081, 992, 925, 903, 869, 846, 800, 760, 733, 663, 603, 578, 526, 429 cm^{-1}; **HRMS (ESI)** calculated for $[C_{12}H_{12}ClFNO]^+$ 240.0586, found 240.0576; ***R*$_f$**: 0.75 (pentane/EtOAc 3/1); **$[\alpha]_D^{20}$** = +102.5 (*c* = 1.0, $CHCl_3$). Chiral HPLC: (Chiralpak AYH, 4.6 x 250 mm; 5% *i*-PrOH / hexane, 1.0 mL/min, 254 nm; t_r (minor) = 12.8 min, t_r (major) = 14.9 min), 94.5/5.5 *er.*

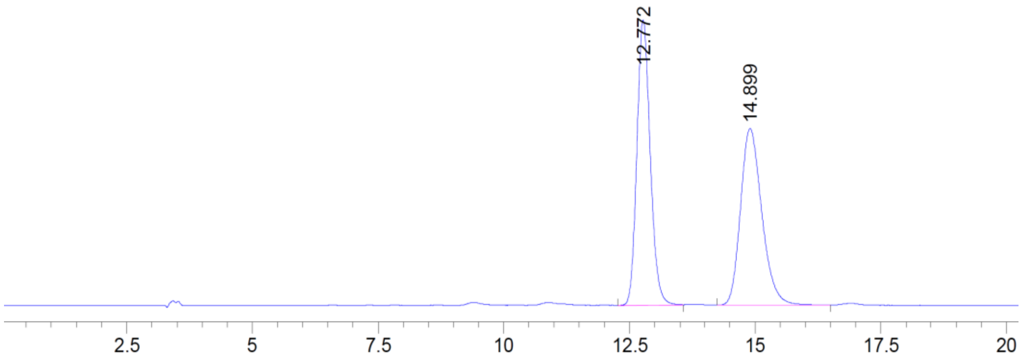

Peak #	RetTime [min]	Type	Width [min]	Area [mAU*s]	Height [mAU]	Area %
1	12.772	BB	0.2844	1147.47058	62.43169	50.1174
2	14.899	BB	0.4522	1142.09399	38.70955	49.8826

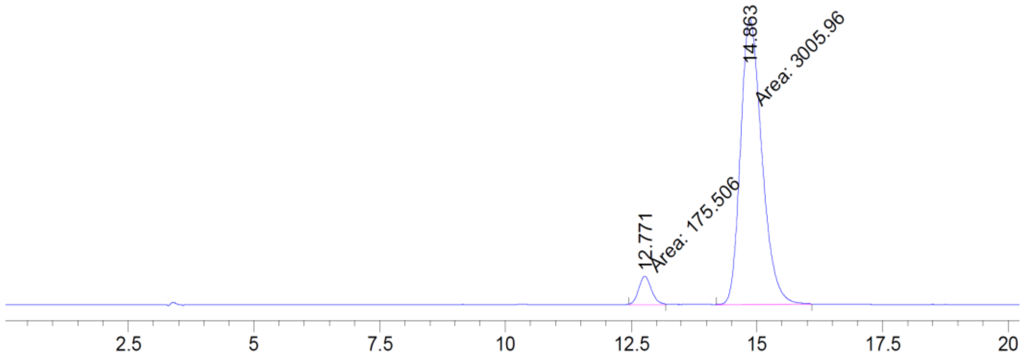

Peak #	RetTime [min]	Type	Width [min]	Area [mAU*s]	Height [mAU]	Area %
1	12.771	FM	0.2917	175.50618	10.02628	5.5165
2	14.863	FM	0.4934	3005.96387	101.54572	94.4835

(6a*R*,7a*S*)-5,6a-dimethyl-7,7a-dihydro-5*H*-cyclopropa[c][1,5]naphthyridin-6(6a*H*)-one (**2n**):

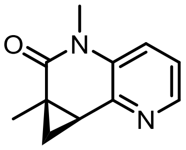

using 10 mol% Pd(dba)$_2$, 20 mol% **L2**, 0.50 equiv. PivOH and 2.0 equiv. Cs_2CO_3, 15% isol. yield. **^{1}H NMR** (400 MHz, $CDCl_3$) δ = 8.22 (dd, *J* = 3.8, 2.3 Hz, 1H), 7.27–7.22 (m, 2H), 3.32 (s, 3H), 2.83 (dd, *J* = 8.8, 5.2 Hz, 1H), 1.60 (dd, *J* = 8.9, 4.7 Hz, 1H), 1.53 (s, 3H), 0.92 (t, *J* = 4.9 Hz, 1H) ppm; **^{13}C{^{1}H} NMR** (101 MHz, $CDCl_3$) δ = 170.2, 144.6, 140.6, 134.1, 121.9, 121.5, 29.5, 29.3, 25.8, 20.1, 19.1 ppm; **IR (ATR):** $\tilde{\nu}$ = 3438, 3087, 2973, 2934, 1661, 1585, 1554, 1456, 1417, 1372, 1352, 1281, 1249, 1208, 1140, 1112, 1043, 1004, 946, 911, 798, 760, 685, 621, 583, 545, 492, 450 cm^{-1}; **HRMS (ESI)** calculated for $[C_{11}H_{13}N_2O]^+$ 189.1022, found 189.1025; ***R*$_f$**: 0.60 (EtOAc); **$[\alpha]_D^{20}$** = -15.4 (*c* = 0.2, $CHCl_3$). Chiral HPLC: (Chiralpak ID, 4.6 x 250 mm; 10% *i*-PrOH / hexane, 1.0 mL/min, 254 nm; t_r (minor) = 27.5 min, t_r (major) = 34.3 min), 54.7/45.3 *er.*

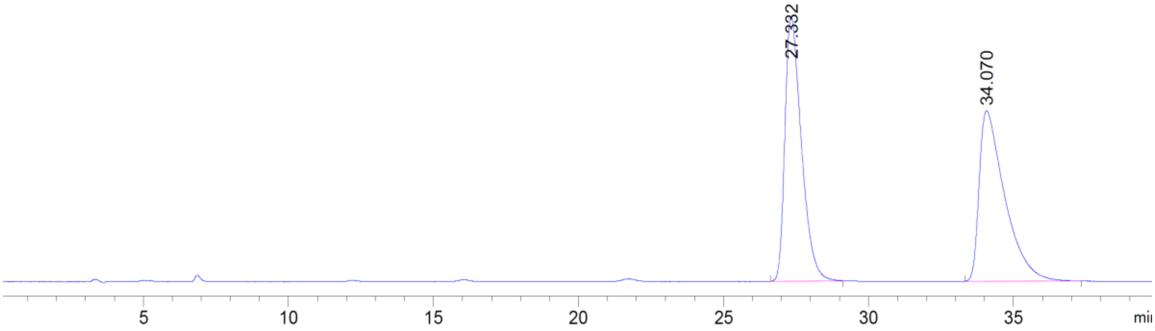

Peak #	RetTime [min]	Type	Width [min]	Area [mAU*s]	Height [mAU]	Area %
1	27.332	BB	0.5963	1044.96558	26.48480	50.0011
2	34.070	BB	0.8477	1044.91882	17.08186	49.9989

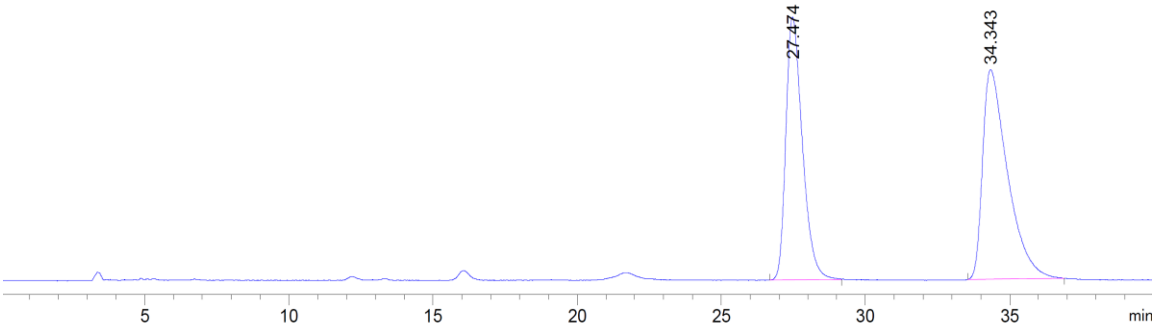

Peak #	RetTime [min]	Type	Width [min]	Area [mAU*s]	Height [mAU]	Area %
1	27.474	BB	0.5965	562.10657	14.11673	45.2798
2	34.343	BB	0.8128	679.29962	11.27627	54.7202

(1a*S*,7b*R*)-3-methyl-1a-(trimethylsilyl)-3,7b-dihydro-1*H*-cyclopropa[c]quinolin-2(1a*H*)-one

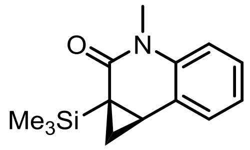

(**2o**): using [(η^3-cinnamyl)Pd(Cp)] as the palladium source, 13% isol. yield. **^{1}H NMR** (400 MHz, $CDCl_3$) δ = 7.34 (dd, *J* = 7.4, 1.6 Hz, 1H), 7.22 (ddd, *J* = 8.2, 7.5, 1.6 Hz, 1H), 7.02 (td, *J* = 7.4, 1.1 Hz, 1H), 6.92 (d, *J* = 8.2 Hz, 1H), 3.31 (s, 3H), 2.38 (dd, *J* = 8.0, 4.9 Hz, 1H), 1.44 (dd, *J* = 8.0, 4.0 Hz, 1H), 0.84 (dd, *J* = 4.9, 4.1 Hz, 1H), 0.14 (s, 9H) ppm; **^{13}C{^{1}H} NMR** (101 MHz, $CDCl_3$) δ = 172.0, 137.6, 128.1, 126.8, 124.4, 122.1, 114.0, 29.2, 23.9, 18.3, 14.5, -2.6 ppm; **IR (ATR):** $\tilde{\nu}$ = 2952, 2929, 2898, 1645, 1600, 1504, 1472, 1415, 1378, 1344, 1304, 1280, 1246, 1221, 1186, 1104, 1046, 1013, 939, 907, 841, 774, 749, 680, 629, 489 cm^{-1}; **HRMS (ESI)** calculated for $[C_{14}H_{20}NOSi]^+$ 246.1309, found 246.1310; $\boldsymbol{R_f}$: 0.70 (pentane/EtOAc 5/1); $[\alpha]_D^{20}$ = +162.5 (*c* = 0.2, $CHCl_3$). Chiral HPLC: Chiralpak ID, 4.6 x 250 mm; 10% *i*-PrOH / hexane, 1.0 mL/min, 254 nm; t_r (minor) = 5.5 min, t_r (major) = 6.0 min), 84.2/15.8 *er.*

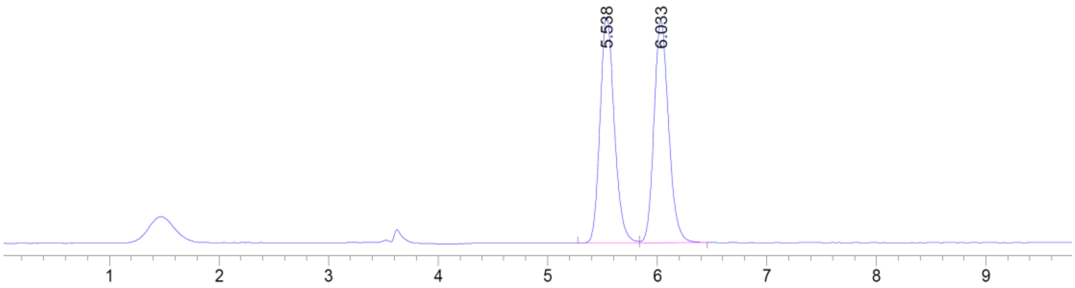

Peak #	RetTime [min]	Type	Width [min]	Area [mAU*s]	Height [mAU]	Area %
1	5.538	BV	0.1418	326.23959	35.96643	49.9404
2	6.033	VB	0.1442	327.01874	35.27081	50.0596

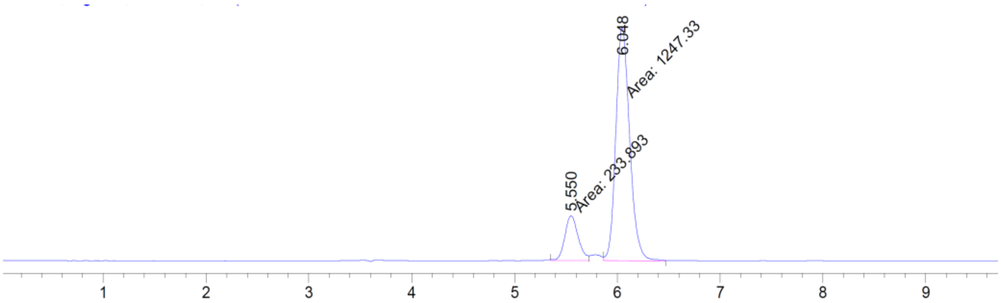

Signal 1: DAD1 A, Sig=254,4 Ref=360,100

Peak #	RetTime [min]	Type	Width [min]	Area [mAU*s]	Height [mAU]	Area %
1	5.550	FM	0.1526	233.89279	25.55264	15.7906
2	6.048	MF	0.1559	1247.32654	133.35989	84.2094

Spectroscopic data for compound **5** was in complete agreement with the reported values: C. L. Ladd, D. Sustac Roman, A. B. Charette, *Org. Lett.* 2013, **15**, 1350.

(3b*R*,4a*R*)-4a-methyl-4,4a-dihydro-3b*H*-cyclopropa[4,5]pyrido[3,2,1-de]phenanthridin-5(7*H*)-one (**2p**): using 4 mol% $Pd(dba)_2$, 8 mol% **L2** and 2.5 equiv. Cs_2CO_3, 95% isol. yield. **^{1}H NMR** (400 MHz, CD_2Cl_2) δ = 7.74–7.68 (m, 2H), 7.40–7.25 (m, 4H), 7.08 (t, *J* = 7.7 Hz, 1H), 5.15 (d, *J* = 16.5 Hz, 1H), 4.76 (d, *J* = 16.4 Hz, 1H), 2.43 (dd, *J* = 8.5, 5.1 Hz, 1H), 1.48 (s, 3H), 1.45 (dd, *J* = 8.5, 4.4 Hz, 1H), 0.71 (t, *J* = 4.7 Hz, 1H) ppm; **^{13}C{^{1}H} NMR** (101 MHz, CD_2Cl_2) δ = 170.4, 132.8, 130.6, 130.2, 127.9, 127.9, 127.2, 126.6, 125.2, 122.6, 122.5, 122.2, 121.0, 45.0, 27.7, 24.9, 20.2, 19.6 ppm; **IR (ATR):** $\tilde{\nu}$ = 3036, 2969, 2929, 2835, 1655, 1604, 1478, 1433, 1374, 1357, 1298, 1196, 1177, 1129, 755, 657, 601 cm^{-1}; **HRMS (ESI)** calculated for $[C_{18}H_{16}NO]^+$ 262.1226, found 262.1225; ***R*$_f$**: 0.50 (pentane/EtOAc 5/1); **$[\alpha]_D^{20}$** = +9.5 (*c* = 1.0, $CHCl_3$). Chiral HPLC: (Chiralpak IB, 4.6 x 250 mm; 10% *i*-PrOH / hexane, 1.0 mL/min, 296 nm; t_r (minor) = 10.6 min, t_r (major) = 16.9 min), 96.6/3.4 *er.*

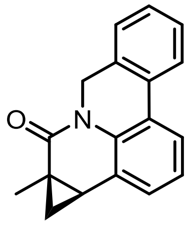

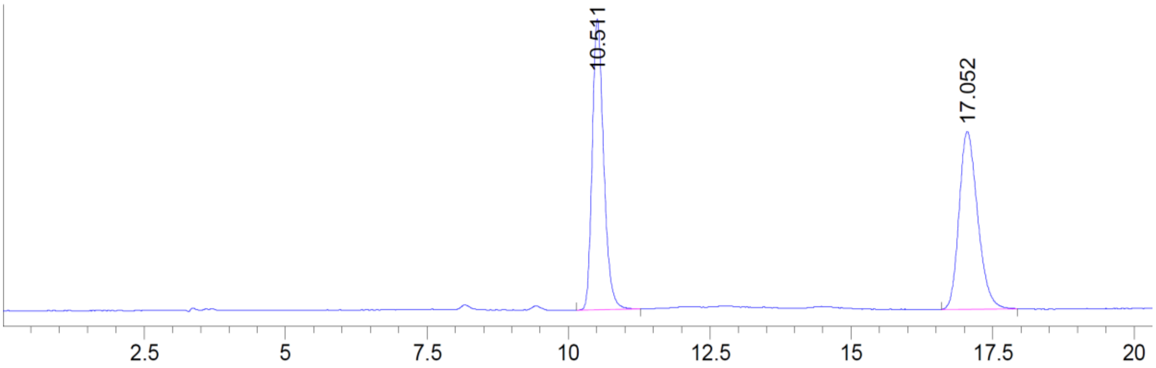

Peak #	RetTime [min]	Type	Width [min]	Area [mAU*s]	Height [mAU]	Area %
1	10.511	BB	0.2156	227.61594	16.23100	50.1093
2	17.052	BB	0.3485	226.62315	9.91949	49.8907

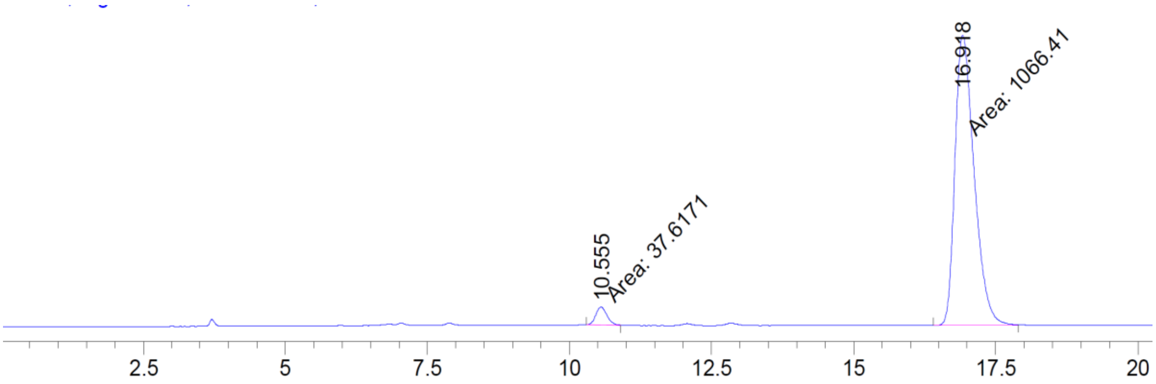

Peak #	RetTime [min]	Type	Width [min]	Area [mAU*s]	Height [mAU]	Area %
1	10.555	MF	0.2255	37.61708	2.77972	3.4073
2	16.918	MF	0.3918	1066.40833	45.36653	96.5927

Cleavage of PBM-protecting group

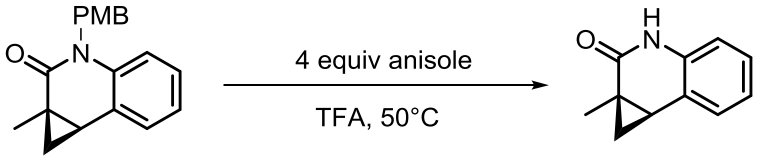

2f **2h**

Compound **2f** (21.3 mg, 73.0 µmol) was weighed into a vial equipped with a magnetic stirring bar and sealed with a rubber septum. Anisole (32.0 µl, 4 equiv.) and TFA (1.45 mL) were added. The mixture was stirred at 50°C for 14 h and the volatiles removed under reduced pressure. Purification by column chromatography on silica gel afforded the free amide **2h** (11.3 mg, 65.0 µmol, 90%) as a colourless oil.

(1a*R*,7b*R*)-1a-methyl-3,7b-dihydro-1*H*-cyclopropa[c]quinolin-2(1a*H*)-one (**2h**):

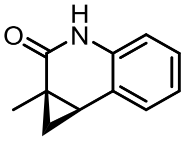

^{1}H NMR (400 MHz, $CDCl_3$) δ = 8.50 (s, 1H), 7.31 (dd, *J* = 7.5, 1.1 Hz, 1H), 7.13 (dd, *J* = 7.7, 1.5 Hz, 1H), 6.99 (td, *J* = 7.5, 1.2 Hz, 1H), 6.80–6.75 (m, 1H), 2.36 (dd, *J* = 8.6, 5.4 Hz, 1H), 1.47 (s, 3H), 1.45 (dd, *J* = 8.6, 4.6 Hz, 1H), 0.85 (t, *J* = 4.9 Hz, 1H) ppm; **^{13}C{^{1}H} NMR** (101 MHz, $CDCl_3$) δ = 172.6, 134.9, 127.4, 126.7, 123.6, 122.6, 115.1, 28.2, 24.1, 19.6, 19.1 ppm; **IR (ATR):** $\tilde{\nu}$ = 3200, 3121, 3060, 2966, 2928, 2861, 1661, 1593, 1558, 1505, 1491, 1458, 1435, 1405, 1379, 1363, 1308, 1363, 1203, 1024, 942, 910, 869, 829, 749, 673, 570, 528, 487, 446, 430 cm^{-1}; **HRMS (ESI)** calculated for $[C_{11}H_{12}NO]^+$ 174.0913, found 174.0913; **$[\alpha]_D^{20}$** = +67.5 (*c* = 1.0, $CHCl_3$); ***R*$_f$**: 0.15 (pentane/EtOAc 3/1).

Determination of the absolute configuration of the dihydroquinolones (2):

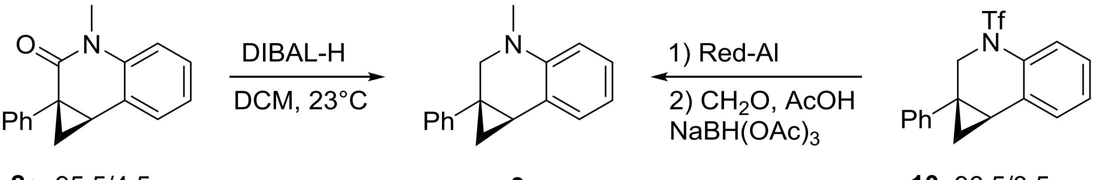

2c, 95.5/4.5 *er* **9** **10**, 96.5/3.5 *er*

from **2c**: $[\alpha]_D$ = 303 ($CHCl_3$, 20°C, c = 1.0)
from **10**: $[\alpha]_D$ = 307 ($CHCl_3$, 20°C, c = 1.0)

Amide **2c** (22.0 mg, 88.0 µmol) was dissolved in DCM (880 µL) and DIBAL-H (1.2 M solution in toluene, 290 µL, 353 µmol) was added. The reaction was stirred at 23°C for 14 h,

quenched with a saturated solution of Rochelle' salt (10 mL) and diluted with ether (25 mL). The biphasic mixture was vigorously stirred for 30 min. The organic layer was washed with aq. NaOH (0.5 M, 25 mL), brine (25 mL) and then dried over $MgSO_4$. The solvent was removed *in vacuo* and the residue was purified by chromatography on silica gel to afford **9** (15.2 mg, 65.0 μmol, 73%).

Compound **10** (68.5 mg, 194 μmol) was weighed into a vial equipped with a magnetic stirring bar, sealed with a rubber septum. The vial was evacuated and then backfilled with nitrogen. Dry toluene (1.45 mL) and Red-Al (3.5 M solution in toluene, 550 μL, 10.0 equiv.) were added. The reaction was stirred for 5 min at 23 °C and heated at 50 °C for 10 h. The reaction mixture was diluted with ether (50 mL) and poured into a saturated Rochelle' salt solution (25 mL). After stirring for 20 min, the layers were separated and the organic layer was washed with aq. NaOH (2 M, 50 mL) and brine (50 mL). The organic layer was dried over $MgSO_4$ and evaporated *in vacuo*. Purification by column chromatography on silica gel afforded the free aniline in 99% yield. The thus obtained aniline (30.0 mg, 136 μmol) and $NaBH(OAc)_3$ (144 mg, 678 μmol, 4.9 eq.) were weighed into a vial equipped with a magnetic stirring bar, sealed with a rubber septum and dissolved in THF (2.7 mL). Aqueous CH_2O (37%, 600 μl, 8.13 mmol, 60 eq.) and AcOH (8.00 μL, 136 μmol, 1.0 eq.) were added and the mixture stirred at 23 °C for 14 h, then diluted with Et_2O (25 mL). The organic layer was washed with aq. NaOH (2 M, 25 mL), brine (25 mL) and then dried over $MgSO_4$. The solvent was removed *in vacuo* and the residue was purified by chromatography on silica gel to afford **9** (22.6 mg, 96.0 μmol, 71%).

<u>(1a*R*,7b*S*)-3-methyl-1a-phenyl-1a,2,3,7b-tetrahydro-1*H*-cyclopropa[c]quino- line (**9**)</u>: **^{1}H-NMR**

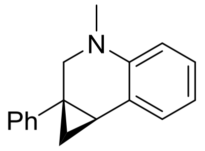

(400 MHz, $CDCl_3$) δ = 7.45–7.39 (m, 2H), 7.39–7.33 (m, 2H), 7.31–7.24 (m, 2H), 7.18–7.12 (m, 1H), 6.80 (td, *J* = 7.4, 1.1 Hz, 1H), 6.68 (d, *J* = 8.0 Hz, 1H), 3.44 (d, *J* = 10.5 Hz, 1H), 3.18 (d, *J* = 10.5 Hz, 1H), 2.85 (s, 3H), 2.24 (dd, *J* = 8.9, 4.8 Hz, 1H), 2.02 (t, *J* = 4.5 Hz, 1H), 1.21 (ddd, *J* = 8.9, 4.1, 0.7 Hz, 1H) ppm; **^{13}C{^{1}H} NMR** (101 MHz, $CDCl_3$) δ = 144.5, 143.0, 128.5, 128.2, 127.8, 127.1, 126.7, 126.3, 118.3, 111.5, 54.9, 38.7, 34.2, 23.8, 14.7 ppm; **IR (ATR):** $\tilde{\nu}$ = 3057, 3023, 2950, 2852, 2800, 1602, 1579, 1498, 1477, 1452, 1422, 1390, 1365, 1327, 1285, 1239, 1202, 1136, 1121, 1096, 1077, 1043, 1030, 968, 938, 895, 861, 746, 720, 698, 639, 588, 555, 543, 514, 480 cm^{-1}; **HRMS (ESI)** calculated for $[C_{17}H_{18}N]^+$ 236.1434, found 236.1429; **$[\alpha]_D^{20}$** = +303 (*c* = 1.0, $CHCl_3$) from **2c**; **$[\alpha]_D^{20}$** = +307 (*c* = 1.0, $CHCl_3$) from **10**; ***R*$_f$**: 0.35 (pentane/EtOAc 5/1).

General procedures for the synthesis of 2-bromo benzamides (3)

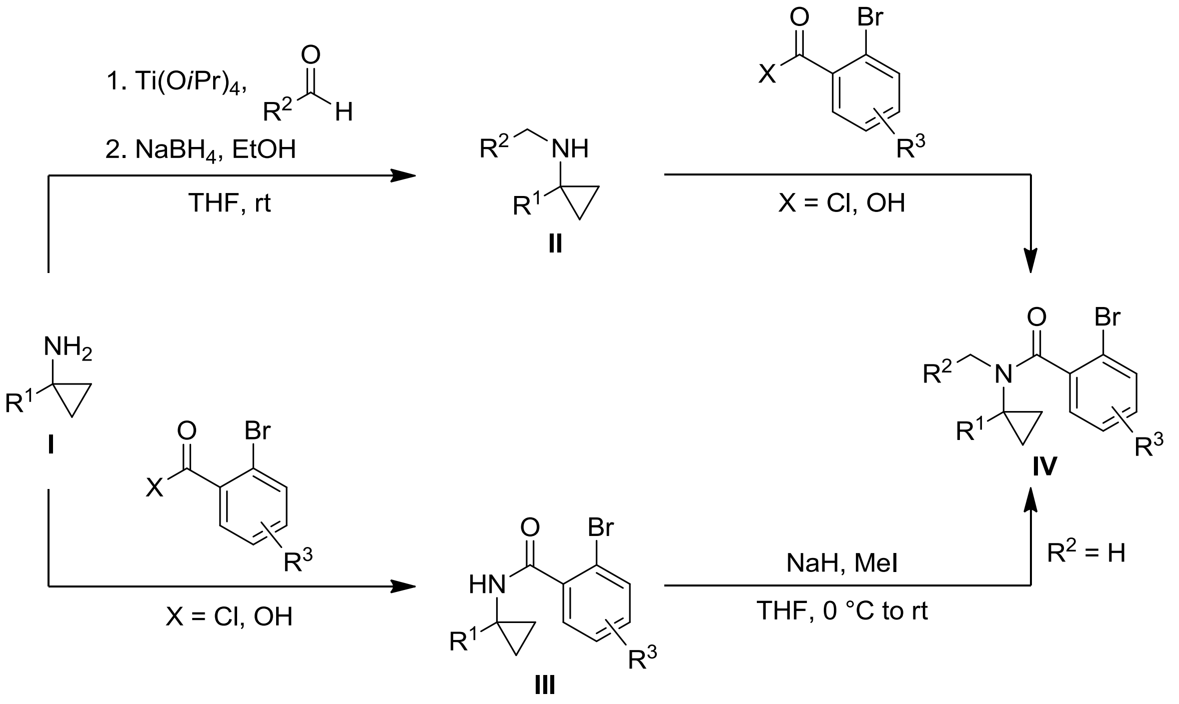

Synthesis of **I** – Substituted cyclopropylamines were obtained from nitriles following procedures described in literature (P. Bertus, J. Szymoniak, *Chem. Commun.* 2001, **18**, 1792 and P. Bertus, J. Szymoniak, *J. Org. Chem.* 2003, **68**, 7133).

Synthesis of **II** – To a solution of **I** in THF (0.25 M) were added aldehyde (1.0 eq.) and $Ti(OiPr)_4$ (1.1 eq.) at rt and the mixture stirred overnight (12-16 h) under N_2. After addition of $NaBH_4$ (2.0 eq.) and EtOH (20 eq.) the mixture was stirred at rt for further 12-16 h, then cooled down to 0 °C, 6 M aq. HCl was added and the cooling bath removed after 30 min. After stirring for 1 h at rt the reaction mixture was basified with 10% aq. NaOH and extracted with DCM, the combined organic phases washed with brine and dried over Na_2SO_4, the solvent removed under reduced pressure and the crude product analysed by ^1H NMR. If necessary, the crude product was purified by column chromatography on silica gel (eluent: DCM/MeOH/NH_3 600/15/1 to 150/15/1).

Synthesis of **III** and **IV** ($R^2 \neq H$)

a) X = Cl: to a solution of **I** or **II** in DCM (0.4 M) were added DIPEA (1.2 eq.) and acid chloride (1.0 eq.) at – 20 °C and the reaction mixture allowed to warm up to rt within 3-4 h. After stirring for further 2-10 h at rt, the reaction mixture was diluted with Et_2O and washed successively with 2 M aq. HCl, aq. sat. $NaHCO_3$ and brine, the organic phase dried over $MgSO_4$ and the solvent removed under reduced pressure. The crude product was purified by column chromatography on silica gel (eluent: pentane/EtOAc 10/1 to 1/1).b) X = OH: to a solution of primary or secondary amine in DCM (0.2 M) was added DIPEA (2.5 eq.), carboxylic acid (1.0 eq.), EDC·HCl (1.1 eq.) and HOBT (0.2 eq.) at 0 °C and the reaction mixture allowed to warm up to rt overnight. After stirring for further 5-7 h at rt, the reaction mixture was diluted with Et_2O and washed successively with 2 M aq. HCl, aq. sat. $NaHCO_3$ and brine, the organic phase dried over $MgSO_4$ and the solvent removed under reduced pressure. The crude product was purified by column chromatography on silica gel (eluent: pentane/EtOAc 10/1 to 1/1).

Synthesis of **IV** ($R^2 = H$)

A solution of **III** in THF (0.2 M) was added dropwise to a suspension of NaH (1.1 eq.) in THF (0.2 M) at 0 °C. After 30 min, MeI (1.2 eq) was added and the reaction mixture allowed to warm up to rt within 2-3 h and stirred for further 1-2 h. The reaction mixture was cooled to 0 °C and quenched with H_2O, the aq. phase extracted with Et_2O, the combined organic phases washed with brine and dried over $MgSO_4$ and the solvent removed under reduced pressure. The crude product was purified by column chromatography on silica gel (eluent: pentane/EtOAc 10/1 to 1/1).

2-bromo-*N*-cyclopropyl-*N*-methylbenzamide (**3a**):

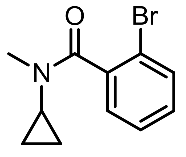

Obtained as a yellow oil. **^{1}H NMR** (400 MHz, $CDCl_3$) (mixture of rotamers in ratio 3.8/1) δ 7.59 – 7.50 (m, 1H), 7.36 – 7.30 (m, 1H), 7.26 – 7.18 (m, 2H), 3.11 (s, 2.4H), 2.92 – 2.85 (m, 0.2H), 2.77 (s, 0.6H), 2.71 (dt, *J* = 11.4, 5.2 Hz, 0.8H), 1.00 - 0.73 (br m, 1H), 0.71 – 0.31 (m, 3H) ppm; **^{13}C{^{1}H} NMR** (101 MHz, $CDCl_3$) δ = 171.4, 170.9, 140.1, 139.1, 132.8, 132.7, 130.3, 130.0, 128.1, 127.9, 127.8, 127.3, 119.2, 36.5, 34.2, 32.1, 30.1 ppm; **IR (ATR):** $\tilde{\nu}$ 3012, 1642, 1590, 1454, 1424, 1384, 1364, 1289, 1106, 1024, 931, 859, 829, 768, 749, 702, 652, 575, 448, 411 cm^{-1}; **HRMS (ESI)** calculated for $[C_{11}H_{13}{}^{79}BrNO]^+$ 254.0175, found 254.0170; **R_f:** 0.39 (pentane/EtOAc 1/1).

2-bromo-*N*-methyl-*N*-(1-(3-phenylpropyl)cyclopropyl)benzamide (**3b**):

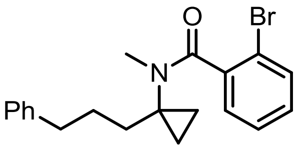

Obtained as a colourless oil. **^{1}H NMR** (400 MHz, $CDCl_3$) (mixture of rotamers in ratio 4/1) δ 7.57 (d, *J* = 9.1 Hz, 0.2H), 7.53 (d, *J* = 7.9 Hz, 0.8H), 7.37 – 7.03 (m, 8H), 3.12 (s, 0.6H), 2.76 (s, 2.4H), 2.67 (t, *J* = 7.1 Hz, 1.6H), 2.59 (d, *J* = 4.5 Hz, 0.4H), 2.15 – 1.47 (m, 4H), 1.17 – 0.35 (m, 4H) ppm; **^{13}C{^{1}H} NMR** (101 MHz, $CDCl_3$) δ = 169.8, 142.5, 141.9, 139.9, 139.6, 133.0, 132.8, 130.0, 129.8, 128.6, 128.4, 127.9, 127.5, 126.8, 126.1, 125.8, 120.2, 119.0, 41.5, 40.2, 38.1, 36.7, 36.2, 36.0, 35.3, 28.7 ppm; **IR (ATR):** $\tilde{\nu}$ 2937, 1646, 1591, 1495, 1475, 1452, 1437, 1381, 1210, 1076, 1025, 768, 748, 699, 683, 635, 449 cm^{-1}; **HRMS (ESI)** calculated for $[C_{20}H_{23}{}^{79}BrNO]^+$ 372.0958, found 372.0955; **R_f:** 0.39 (pentane/EtOAc 3/1).

2-bromo-*N*-cyclopropyl-*N*-(4-methoxybenzyl)benzamide (**3c**):

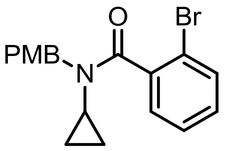

Obtained as a white solid. **^{1}H NMR** (400 MHz, $CDCl_3$) (mixture of rotamers in ratio 5/1) δ 7.57 – 7.52 (m, 1H), 7.39 (d, *J* = 8.6 Hz, 1.7H), 7.35 – 7.29 (m, 1.2H), 7.24 – 7.18 (m, 1.8H), 7.08 (d, *J* = 8.6 Hz, 0.3H), 6.88 (d, *J* = 8.7 Hz, 1.7H), 6.84 (d, *J* = 8.6 Hz, 0.3H), 4.71 (d, *J* = 71.2 Hz, 1.7H), 4.30 – 4.16 (m, 0.3H), 3.82 (s, 2.5H), 3.79 (s, 0.5H), 2.64 - 2.58 (m, 0.3H), 2.50 (ddd, *J* = 11.5, 7.0, 4.6 Hz, 0.7H), 1.03 – 0.39 (m, 4H) ppm; **^{13}C{^{1}H} NMR** (101 MHz, $CDCl_3$) δ = 171.3, 159.1, 140.0, 132.9, 130.1, 129.9, 129.8, 128.7, 128.1, 127.3, 119.3, 114.1, 113.9, 55.4, 52.7, 49.6, 30.5, 28.4 ppm; **IR (ATR):** $\tilde{\nu}$ 3007, 2937, 1643, 1612, 1588, 1512, 1432, 1402, 1302, 1287, 1247, 1176, 1109, 1029, 983, 833, 759, 605, 556 cm^{-1}; **HRMS (ESI)** calculated for $[C_{18}H_{19}{}^{79}BrNO_2]^+$ 360.0594, found 360.0599; **R_f:** 0.28 (pentane/EtOAc 3/1); **m.p.:** 67.6-71.2 °C.

2-bromo-*N*-cyclopropyl-*N*-(2,4,6-trimethylbenzyl)benzamide (**3d**):

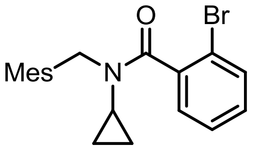

Obtained as a colourless wax. **^{1}H NMR** (400 MHz, $CDCl_3$) (mixture of rotamers in ratio 7.3/1) δ 7.58 (d, *J* = 8.0 Hz, 1H), 7.38 – 7.26 (m, 2H), 7.24 – 7.17 (m, 1H), 6.88 (s, 1.75H), 6.81 (s, 0.25H), 5.38 – 4.09 (m, 2H), 2.45 – 2.23 (m, 10H), 0.86 – 0.79 (m, 1H), 0.39 – 0.14 (m, 3H) ppm; **^{13}C{^{1}H} NMR** (101 MHz, $CDCl_3$) δ = 170.5, 140.3, 138.2, 137.2, 133.2, 130.3, 130.0, 129.4, 127.9, 127.1, 119.9, 44.2, 30.0, 21.0, 20.5 ppm; **IR (ATR):** $\tilde{\nu}$ 2951, 2923, 1645, 1468, 1432, 1403, 1373, 1300, 1253, 1027, 769, 746, 400 cm^{-1}; **HRMS (ESI)** calculated for $[C_{20}H_{23}{}^{79}BrNO]^+$ 372.0958, found 372.0953; **R_f:** 0.28 (pentane/EtOAc 2/1).

2-bromo-*N*-cyclopropyl-*N*-methylbenzamide (**3e**):

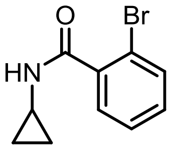

Obtained as a white solid. **^{1}H NMR** (400 MHz, $CDCl_3$) 7.56 (dd, *J* = 8.0, 0.9 Hz, 1H), 7.52 (dd, *J* = 7.6, 1.7 Hz, 1H), 7.34 (td, *J* = 7.5, 1.1 Hz, 1H), 7.29 – 7.22 (m, 1H), 6.06 (s, 1H), 2.92 (tq, *J* = 7.1, 3.7 Hz, 1H), 0.88 (dt, *J* = 6.9, 3.3 Hz, 2H), 0.69 – 0.64 (m, 2H) ppm; **^{13}C{^{1}H} NMR** (101 MHz, $CDCl_3$) δ = 169.1, 137.8, 133.4, 131.4, 129.7, 127.7, 119.4, 23.2, 6.9 ppm; **IR (ATR):** $\tilde{\nu}$ 3249, 1641, 1590, 1539, 1452, 1430, 1361, 1322, 1309, 1260, 859, 773, 756, 687 cm^{-1}; **HRMS (ESI)** calculated for $[C_{10}H_{11}{}^{79}BrNO]^+$ 240.0019, found 240.0013; **R_f:** 0.37 (pentane/EtOAc 1/1); **m.p.:** 121.3-123.2 °C.

2-bromo-*N*-(4-methoxybenzyl)-*N*-(1-(3-phenylpropyl)cyclopropyl)benzamide (**3f**):

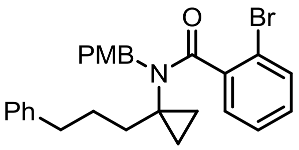

Obtained as a yellow oil. **^{1}H NMR** (400 MHz, $CDCl_3$) (mixture of rotamers in ratio 2.5/1) δ 7.61 – 7.56 (m, 0.25H), 7.50 – 7.45 (m, 0.75H), 7.35 – 6.96 (m, 9.5H), 6.86 (dd, *J* = 13.6, 8.6 Hz, 2H), 6.73 (d, *J* = 8.7 Hz, 1.5H), 5.11 (br s, 0.25H), 4.52 – 4.16 (m, 1.75H), 3.80 (s, 0.85H), 3.77 (s, 2.15H), 2.55 (t, *J* = 7.5 Hz, 2H), 2.07 – 0.69 (m, 8H) ppm; **^{13}C{^{1}H} NMR** (101 MHz, $CDCl_3$) δ = 170.5, 159.0, 158.7, 142.6, 139.3, 133.1, 132.6, 131.2, 130.4, 129.9, 129.8, 128.6, 128.4, 128.2, 127.4, 126.7, 126.1, 125.8, 119.0, 113.9, 55.4, 53.5, 40.7, 36.3, 36.2, 36.0, 28.6 ppm; **IR (ATR):** $\tilde{\nu}$ 2936, 1644, 1612, 1512, 1453, 1440, 1427, 1398, 1357, 1302, 1248, 1175, 1029, 771, 749, 700 cm^{-1}; **HRMS (ESI)** calculated for $[C_{27}H_{29}{}^{79}BrNO_2]^+$ 478.1376, found 478.1378; **R_f:** 0.32 (pentane/EtOAc 3/1).

2-bromo-*N*-(1-isopropylcyclopropyl)-*N*-methylbenzamide (**3g**):

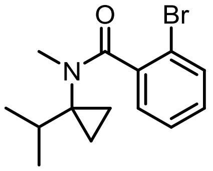

Obtained as a white solid. **^{1}H NMR** (400 MHz, $CDCl_3$) (mixture of rotamers in ratio 9/1) δ 7.60 – 7.49 (m, 8.1 Hz, 1H), 7.37 – 7.30 (m, 1H), 7.23 – 7.15 (m, 2H), 3.14 (s, 0.3H), 2.80 (s, 2.7H), 1.92 (dt, *J* = 13.8, 6.9 Hz, 1H), 1.09 – 0.81 (m, 10H) ppm; **^{13}C{^{1}H} NMR** (101 MHz, $CDCl_3$) δ = 170.1, 132.9, 130.5, 129.9, 128.5, 127.9, 127.5, 126.1, 118.8, 44.8, 38.1, 34.3, 34.0, 20.1, 20.0 ppm; **IR (ATR):** $\tilde{\nu}$ 3013, 2963, 2872, 1652, 1591, 1468, 1439, 1378, 1209, 1077, 1026, 769, 749, 673, 636 cm^{-1}; **HRMS (ESI)** calculated for $[C_{14}H_{19}{}^{79}BrNO]^+$ 296.0645, found 296.0646; **R_f:** 0.53 (pentane/EtOAc 3/1); **m.p.:** 62.1-63.2 °C.

2-bromo-*N*-methyl-*N*-(1-phenylcyclopropyl)benzamide (**3h**):

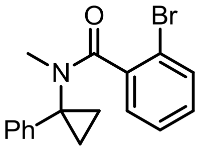

Obtained as a white solid. ^{1}**H NMR** (400 MHz, $CDCl_3$) (mixture of rotamers in ratio 2.0/1) δ 7.59 – 7.52 (m, 1H), 7.39 – 7.29 (m, 4.7H), 7.23 (ddd, *J* = 7.4, 4.9, 1.7 Hz, 1.7H), 7.12 (td, *J* = 7.8, 1.7 Hz, 0.3H), 7.00 – 6.92 (m, 1H), 6.86 (dd, *J* = 7.7, 1.6 Hz, 0.3H), 3.31 (s, 1H), 2.86 (s, 2H), 1.71 – 1.31 (m, 4.3H), 1.03 (s, 0.7H) ppm; ^{13}**C{^{1}H} NMR** (101 MHz, $CDCl_3$) δ = 171.2, 170.0, 143.2, 140.9, 139.5, 139.1, 133.0, 132.9, 130.4, 130.0, 128.9, 128.7, 128.0, 128.0, 126.9, 126.8, 126.7, 126.4, 126.1, 123.8, 120.3, 119.2, 43.9, 42.3, 36.4, 35.2, 19.4, 17.6 ppm; **IR (ATR):** $\tilde{\nu}$ 3059, 1650, 1602, 1591, 1497, 1479, 1459, 1437, 1382, 1330, 1211, 1080, 1030, 751, 699, 680, 665, 636, 556 cm^{-1}; **HRMS (ESI)** calculated for $[C_{17}H_{17}{}^{79}BrNO]^+$ 330.0488, found 330.0490; **R$_f$:** 0.36 (pentane/EtOAc 3/1); **m.p.:** 72.6-73.7 °C.

2-bromo-*N*-(1-(4-chlorophenyl)cyclopropyl)-*N*-(4-methoxybenzyl)benzamide (**3i**):

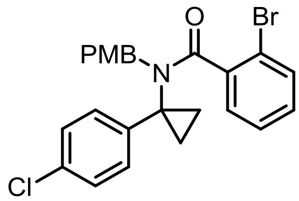

Obtained as a colourless wax. ^{1}**H NMR** (400 MHz, $CDCl_3$) (mixture of rotamers in ratio 1/1) 7.59 – 7.55 (m, 0.5H), 7.51 (dd, *J* = 7.8, 1.1 Hz, 0.5H), 7.39 (d, *J* = 8.7 Hz, 1H), 7.33 – 7.29 (m, 2H), 7.25 – 7.12 (m, 3H), 7.00 (t, *J* = 7.5 Hz, 0.5H), 6.90 – 6.84 (m, 2H), 6.81 (d, *J* = 6.9 Hz, 0.5H), 6.72 (d, *J* = 8.7 Hz, 1H), 6.65 – 6.56 (m, 1H), 5.98 – 5.38 (br s, 0.5H), 4.49 – 4.02 (br m, 1.5H), 3.81 (s, 1.5H), 3.73 (s, 1.5H), 1.48 – 1.22 (m, 3H), 0.84 (br s, 1H) ppm; ^{13}**C{^{1}H} NMR** (101 MHz, $CDCl_3$) δ = 171.9, 170.7, 159.1, 159.0, 142.4, 140.5, 139.3, 138.8, 133.1, 132.8, 132.6, 132.2, 130.5, 130.3, 130.1, 129.9, 129.2, 129.0, 128.5, 128.1, 127.7, 126.7, 126.7, 125.3, 120.2, 119.2, 114.0, 113.8, 55.4, 55.4, 53.2, 51.7, 43.6, 42.0 ppm; **IR (ATR):** $\tilde{\nu}$ 2932, 1644, 1612, 1588, 1512, 1492, 1463, 1428, 1396, 1359, 1329, 1303, 1248, 1176, 1109, 1096, 1031, 1011, 983, 946, 910, 821, 772, 752, 732, 698, 676, 646, 566, 527 cm^{-1}; **HRMS (ESI)** calculated for $[C_{24}H_{22}{}^{79}BrClNO_2]^+$ 470.0517, found 470.0519; **R$_f$:** 0.38 (pentane/EtOAc 3/1).

N-(1-benzylcyclopropyl)-2-bromo-*N*-methylbenzamide (**3j**):

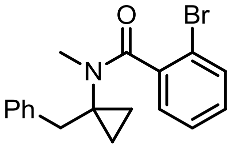

Obtained as a yellow oil. ^{1}**H NMR** (400 MHz, $CDCl_3$) (mixture of rotamers in ratio 4/1) δ = 7.58 (d, *J* = 7.8 Hz, 0.2H), 7.55 – 7.47 (m, 0.8H), 7.37 – 7.27 (m, 5H), 7.25 – 7.14 (m, 2H), 7.08 (m, 0.8H), 6.95 (br s, 0.2H), 3.35 (br s, 1H), 2.95 (s, 0.6H), 2.89 (br s, 1H), 2.37 (s, 2.4H), 1.17 – 0.24 (m, 4H) ppm; ^{13}**C{^{1}H} NMR** (101 MHz, $CDCl_3$) δ = 170.7, 170.0, 139.4, 138.8, 137.4, 133.1, 132.9, 130.1, 130.1, 130.0, 128.7, 128.4, 127.9, 127.6, 127.1, 126.8, 126.7, 119.0, 42.7, 41.7, 40.6, 36.8, 34.8 ppm; **IR (ATR):** $\tilde{\nu}$ 3026, 1648, 1591, 1494, 1478, 1454, 1440, 1383, 1075, 1027, 769, 747, 703, 637 cm^{-1}; **HRMS (ESI)** calculated for $[C_{18}H_{19}{}^{79}BrNO]^+$ 344.0645, found 344.0649; **R$_f$:** 0.33 (pentane/Et_2O 1/1).

Ethyl 1-(2-bromo-*N*-methylbenzamido)cyclopropanecarboxylate (**3k**):

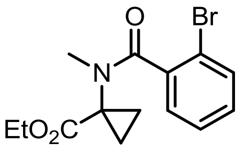

Obtained as a colourless oil. **^{1}H NMR** (400 MHz, $CDCl_3$) (mixture of rotamers in ratio 1.5/1) δ 7.58 (d, *J* = 7.6 Hz, 0.4H), 7.54 (d, *J* = 8.0 Hz, 0.6H), 7.37 (td, *J* = 7.5, 0.9 Hz, 0.6H), 7.30 (d, *J* = 6.7 Hz, 0.4H), 7.26 – 7.18 (m, 2H), 4.29 – 4.12 (m, 2H), 3.18 (s, 1.2H), 2.86 (s, 1.8H), 1.91 – 1.11 (m, 7H) ppm; **^{13}C{^{1}H} NMR** (101 MHz, $CDCl_3$) δ = 172.4, 171.9, 170.9, 170.6, 139.5, 138.7, 133.3, 132.9, 130.5, 130.3, 128.1, 127.9, 127.2, 126.7, 119.8, 119.0, 61.9, 61.6, 43.3, 40.7, 37.1, 34.9, 20.8, 18.7, 14.4 ppm; **IR (ATR):** $\tilde{\nu}$ 2981, 1728, 1657, 1591, 1476, 1437, 1385, 1369, 1328, 1298, 1214, 1189, 1136, 1080, 1024, 771, 749, 448 cm^{-1}; **HRMS (ESI)** calculated for $[C_{14}H_{17}{}^{79}BrNO_3]^+$ 326.0386, found 326.0390; **R_f:** 0.19 (pentane/EtOAc 3/1).

2-bromo-*N*-(1-cyanocyclopropyl)-*N*-methylbenzamide (**3l**):

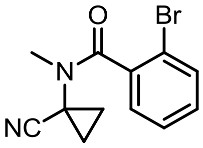

Obtained as a pale yellow oil. **^{1}H NMR** (400 MHz, $CDCl_3$) (mixture of rotamers in ratio 1.5/1) δ 7.63 (d, *J* = 8.0 Hz, 0.4H), 7.58 – 7.49 (m, 1H), 7.48 – 7.36 (m, 1H), 7.31 (dd, *J* = 13.6, 7.5 Hz, 1.6H), 3.22 (s, 1.2H), 2.90 (s, 1.8H), 1.81 – 1.09 (m, 4H) ppm; **^{13}C{^{1}H} NMR** (101 MHz, $CDCl_3$) δ = 170.5, 138.1, 137.2, 133.4, 133.0, 131.1, 131.0, 128.1, 127.7, 127.5, 120.0, 119.5, 119.3, 119.1, 36.4, 34.1, 30.7, 27.9, 17.5 ppm; **IR (ATR):** $\tilde{\nu}$ 2237, 1659, 1590, 1476, 1431, 1371, 1323, 1214, 1158, 1077, 1048, 1029, 771, 750, 673, 636, 566 cm^{-1}; **HRMS (ESI)** calculated for $[C_{12}H_{12}{}^{79}BrN_2O]^+$ 279.0128, found 279.0132; **R_f:** 0.16 (pentane/EtOAc 3/1).

2-bromo-4-chloro-*N*-(4-methoxybenzyl)-*N*-(1-(3-phenylpropyl)cyclopropyl)benzamide (**3m**):

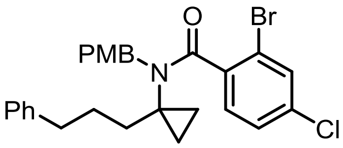

Obtained as a colourless oil. **^{1}H NMR** (400 MHz, $CDCl_3$) (mixture of rotamers in ratio 2.75/1) δ 7.54 – 7.50 (m, 0.25H), 7.40 – 7.36 (m, 0.75H), 7.32 – 7.26 (m, 2.5H), 7.24 – 7.10 (m, 3.75H), 7.06 (dd, *J* = 8.6, 2.5 Hz, 0.75H), 6.88 – 6.77 (m, 2.5H), 6.77 – 6.71 (m, 1.5H), 5.05 (br s, 0.25H), 4.55 – 4.21 (m, 1.75H), 3.80 (s, 0.75H), 3.78 (s, 2.25H), 2.67 – 2.46 (m, 2H), 2.06 – 0.39 (m, 8H) ppm; **^{13}C{^{1}H} NMR** (101 MHz, $CDCl_3$) δ = 169.1, 159.1, 158.8, 142.5, 140.6, 134.4, 133.7, 133.0, 130.8, 130.1, 130.0, 128.7, 128.6, 128.4, 128.2, 126.2, 125.9, 116.7, 114.1, 114.0, 55.5, 55.4, 53.8, 41.1, 36.2, 36.2, 36.0, 28.6 ppm; **IR (ATR):** $\tilde{\nu}$ 2935, 1646, 1612, 1512, 1496, 1454, 1425, 1405, 1372, 1356, 1322, 1302, 1248, 1175, 1095, 1032, 815, 749, 700, 504 cm^{-1}; **HRMS (ESI)** calculated for $[C_{27}H_{28}{}^{79}BrClNO_2]^+$ 512.0986, found 512.0967; **R_f:** 0.55 (pentane/EtOAc 3/1).

2-bromo-5-methoxy-*N*-(4-methoxybenzyl)-*N*-(1-(3-phenylpropyl)cyclopropyl)benzamide (**3n**):

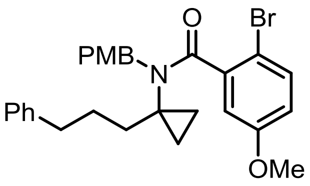

Obtained as a colourless oil. **^{1}H NMR** (400 MHz, $CDCl_3$) (mixture of rotamers in ratio 2.8/1) δ 7.46 (d, *J* = 8.6 Hz, 0.2H), 7.37 – 7.23 (m, 3.8H), 7.22 – 7.09 (m, 3H), 6.89 – 6.80 (m, 2H), 6.76 – 6.70 (m, 1.5H), 6.66 (dd, *J* = 8.8, 3.0 Hz, 0.75H), 6.43 (br s, 0.75H), 5.09 (s, 0.3H), 4.33 (d, *J* = 4.9 Hz, 1.7H), 3.79 (s, 0.8H), 3.76 (s, 2.2H), 3.66 (s, 0.8H), 3.56 (s, 2.2H), 2.57 (t, *J* = 7.6 Hz, 2H), 2.23 – 1.60 (m, 3H), 1.24 – 0.37 (m, 5H) ppm; **^{13}C{^{1}H} NMR** (101 MHz, $CDCl_3$) δ = 170.4, 159.0, 158.8, 158.3, 142.6, 139.9, 133.9, 133.3, 130.7, 128.6, 128.6, 128.4, 128.2, 126.1, 125.8, 117.0, 113.9, 113.1, 109.1, 55.6, 55.4, 53.7, 41.0, 38.4, 36.3, 36.1, 28.7 ppm; **IR (ATR):** $\tilde{\nu}$ 2937, 2836, 1649, 1612, 1591, 1571, 1513, 1496, 1462, 1412, 1386, 1356, 1290, 1248, 1175, 1112, 1021, 817, 749, 701, 600 cm^{-1}; **HRMS (ESI)** calculated for $[C_{28}H_{31}{}^{79}BrNO_3]^+$ 508.1482, found 508.1488; **R_f:** 0.22 (pentane/EtOAc 3/1).

3-bromo-*N*-(4-methoxybenzyl)-*N*-(1-(3-phenylpropyl)cyclopropyl)isonicotinamide (**3o**):

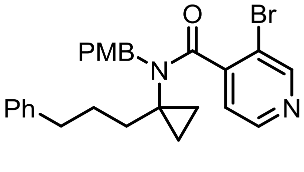

Obtained as a colourless oil. **^{1}H NMR** (400 MHz, $CDCl_3$) (mixture of rotamers in ratio 2/1) δ 8.82 – 8.59 (m, 1H), 8.49 – 8.22 (m, 1H), 7.35 – 7.09 (m, 6H), 6.87 – 6.71 (m, 4H), 5.08 (br s, 0.25H), 4.46 – 4.18 (m, 1.75H), 3.80 (s, 1H), 3.77 (s, 2H), 2.69 – 2.48 (m, 2H), 2.19 – 0.10 (m, 8H) ppm; **^{13}C{^{1}H} NMR** (101 MHz, $CDCl_3$) δ = 169.1, 168.2, 159.2, 158.9, 152.4, 151.9, 148.1, 147.8, 147.1, 146.3, 142.4, 141.6, 130.5, 129.8, 128.8, 128.6, 128.6, 128.5, 127.9, 126.3, 125.9, 122.4, 117.1, 114.1, 114.0, 55.4, 55.4, 53.5, 51.9, 41.2, 37.9, 36.2, 36.1, 35.8, 28.7, 28.1 ppm; **IR (ATR):** $\tilde{\nu}$ 2937, 1649, 1612, 1513, 1453, 1425, 1404, 1303, 1249, 1176, 1030, 839, 750, 701 cm^{-1}; **HRMS (ESI)** calculated for $[C_{26}H_{28}{}^{79}BrN_2O_2]^+$ 479.1329, found 479.1335; **R_f:** 0.29 (pentane/EtOAc 1/1).

4-bromo-*N*-(4-methoxybenzyl)-*N*-(1-(3-phenylpropyl)cyclopropyl)thiophene-3-carboxamide (**3p**) :

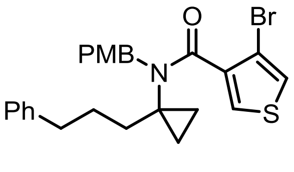

Obtained as a colourless oil. **^{1}H NMR** (400 MHz, $CDCl_3$) δ 7.36 – 7.07 (m, 7H), 7.02 – 6.75 (m, 4H), 5.04 (br s, 0.5H), 4.37 (br s, 1.5H), 3.78 (s, 3H), 2.55 (t, *J* = 7.2 Hz, 2H), 1.98 – 1.44 (m, 3H), 1.18 – 0.38 (m, 5H) ppm; **^{13}C{^{1}H} NMR** (101 MHz, $CDCl_3$) δ = 167.2, 159.0, 142.6, 141.9, 138.7, 130.8, 128.6, 128.4, 127.6, 126.2, 125.8, 124.7, 123.7, 123.3, 114.0, 55.4, 53.8, 51.8, 41.2, 38.2, 36.3, 35.9, 28.6, 28.2 ppm; **IR (ATR):** $\tilde{\nu}$ 3084, 3025, 2937, 1643, 1613, 1585, 1512, 1453, 1427, 1387, 1357, 1301, 1248, 1175, 1031, 852, 803, 749, 701 cm^{-1}; **HRMS (ESI)** calculated for $[C_{25}H_{27}{}^{79}BrNO_2S]^+$ 484.0940, found 484.0936; **R_f:** 0.35 (pentane/EtOAc 3/1).

General procedure for the enantioselective synthesis of cyclopropane containing dihydroisoquinolones (4):

$Pd(dba)_2$ (1.44 mg, 2.50 μmol, 2.5 mol%), **L2** (3.26 mg, 5.0 μmol, 10 mol%), benzamide (**3**, 0.10 mmol), $AdCO_2H$ (3.60 mg, 0.02 mmol, 20 mol%), and Cs_2CO_3 (49.0 mg, 0.15 mmol, 1.5 eq) were weighed in a tube containing a magnetic stirring bar. The tube was sealed with a rubber septum, evacuated and refilled with nitrogen. After addition of toluene (0.4 ml, 0.25 M) the reaction mixture was degassed by three freeze-pump-thaw cycles and stirred for 12 h at 110 °C. After cooling to rt the mixture was filtered over a pad of silica gel (eluted with EtOAc) and the crude analysed by ^1H NMR (2.5 – 5.0 μmol 1,3,5-trimethoxybenzene added as internal standard). The crude mixture was chromatographed on silica gel (eluted with pentane/EtOAc 10/1 to 1/1) to afford the corresponding dihydroisoquinolone **4**.

(1a*R*,7b*R*)-2-methyl-1a,2-dihydro-1*H*-cyclopropa[c]isoquinolin-3(7b*H*)-one (**4a**):

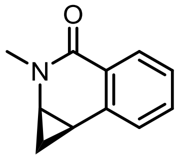

Obtained as a colourless oil in 64% yield (11.0 mg). **^{1}H NMR** (400 MHz, $CDCl_3$) δ = 8.16 (d, *J* = 7.8 Hz, 1H), 7.42 (ddd, *J* = 16.7, 11.5, 3.8 Hz, 2H), 7.33 – 7.27 (m, 1H), 3.25 (s, 3H), 3.15 (ddd, *J* = 8.2, 6.5, 3.9 Hz, 1H), 2.31 (ddd, *J* = 9.5, 8.3, 5.8 Hz, 1H), 1.35 (ddd, *J* = 9.8, 6.4, 5.2 Hz, 1H), 0.27 (td, *J* = 5.4, 4.0 Hz, 1H) ppm; **^{13}C{^{1}H} NMR** (101 MHz, $CDCl_3$) δ = 161.5, 139.3, 131.9, 129.1, 128.2, 126.6, 125.6, 36.3, 34.9, 15.0, 13.8 ppm; **IR (ATR):** $\tilde{\nu}$ 2917, 1644, 1604, 1577, 1483, 1441, 1428, 1395, 1377, 1359, 1275, 1173, 1103, 1033, 822, 761, 710, 693, 553 cm^{-1}; **HRMS (ESI)** calculated for $[C_{11}H_{12}NO]^+$ 174.0913, found 174.0913; **R_f:** 0.30 (pentane/EtOAc 1/1); **$[\alpha]_D^{20}$:** -116.7° (*c* = 0.1, $CHCl_3$). Chiral HPLC: (Chiralpak IA, 4.6 x 250 mm; 20% *i*-PrOH / hexane, 1.0 mL/min, 254 nm; t_r (minor) = 10.2 min, t_r (major) = 7.0 min), 93.7/6.3 *er.*

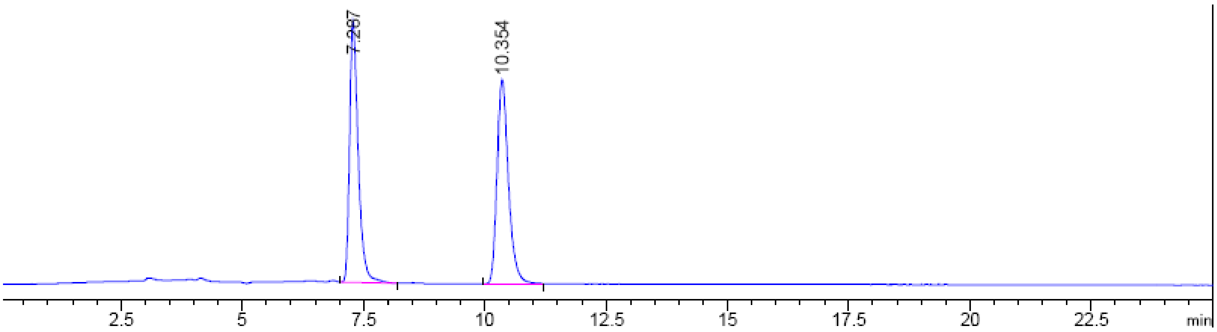

Peak #	RetTime [min]	Type	Width [min]	Area [mAU*s]	Height [mAU]	Area %
1	7.287	BB	0.1856	626.10437	50.15298	49.9592
2	10.354	BB	0.2425	627.12732	39.22387	50.0408

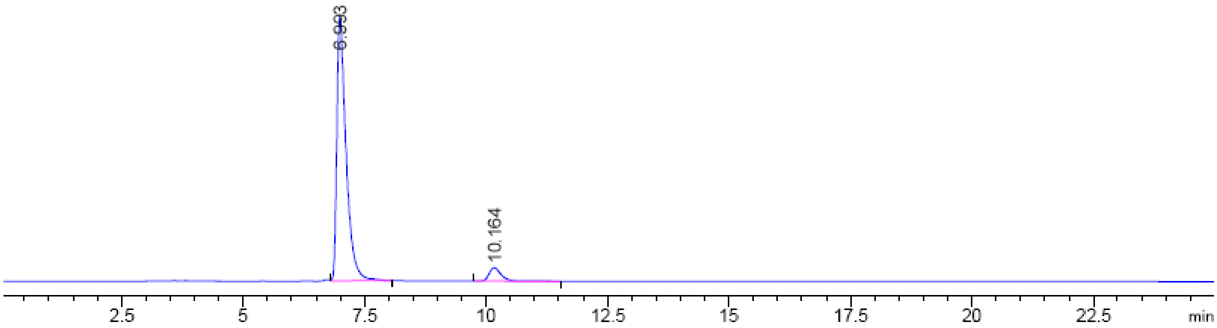

Peak #	RetTime [min]	Type	Width [min]	Area [mAU*s]	Height [mAU]	Area %
1	6.993	VB	0.1965	4336.31299	323.33264	93.6552
2	10.164	BB	0.2632	293.76965	16.86568	6.3448

Spectroscopic data for compounds **14a**, **14b** and **15** were in complete agreement with the reported values: C. L. Ladd, D. Sustac Roman, A. B. Charette, *Tetrahedron* 2013, **69**, 4479.

(1a*R*,7b*R*)-2-methyl-1a-(3-phenylpropyl)-1a,2-dihydro-1*H*-cyclopropa[c]isoquinolin-3(7b*H*)-one (**4b**): obtained as a colourless oil in 93% yield (27.0 mg). **^{1}H NMR** (400 MHz, $CDCl_3$) δ = 8.12 (d, *J* = 7.8 Hz, 1H), 7.42 (td, *J* = 7.5, 1.3 Hz, 1H), 7.32 (d, *J* = 7.5 Hz, 1H), 7.30 – 7.22 (m, 3H), 7.21 – 7.08 (m, 3H), 3.14 (s, 3H), 2.69 – 2.55 (m, 2H), 2.45 (ddd, *J* = 14.8, 10.6, 4.3 Hz, 1H), 2.09 – 2.03 (m, 1H), 1.85 – 1.67 (m, 2H), 1.19 (dd, *J* = 9.9, 4.8 Hz, 1H), 0.92 (ddd, *J* = 14.8, 11.0, 6.1 Hz, 1H), 0.49 (dd, *J* = 5.8, 5.0 Hz, 1H) ppm; **^{13}C{^{1}H} NMR** (101 MHz, $CDCl_3$) δ = 162.9, 141.8, 138.7, 131.9, 129.1, 128.5, 128.4, 128.0, 126.7, 126.1, 125.2, 43.6, 35.8, 35.4, 30.8, 27.6, 22.7, 19.3 ppm; **IR (ATR):** $\tilde{\nu}$ 2935, 1641, 1603, 1579, 1481, 1453, 1413, 1396, 1369, 1280, 1161, 1030, 750, 697, 562 cm^{-1}; **HRMS (ESI)** calculated for $[C_{20}H_{22}NO]^+$ 292.1696, found 292.1699; **R_f:** 0.28 (pentane/EtOAc 3/1); **$[\alpha]_D^{20}$:** -68.3° (*c* = 0.1, $CHCl_3$). Chiral HPLC: (Chiralpak IA, 4.6 x 250 mm; 20% *i*-PrOH / hexane, 1.0 mL/min, 254 nm; t_r (minor) = 7.9 min, t_r (major) = 7.0 min), 93.6/6.4 *er.*

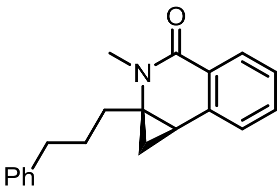

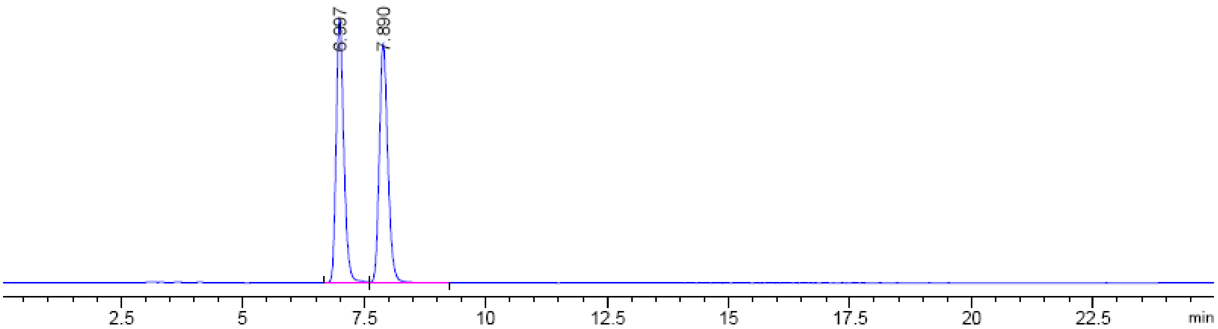

Peak #	RetTime [min]	Type	Width [min]	Area [mAU*s]	Height [mAU]	Area %
1	6.997	BV	0.1697	1759.38123	158.23108	49.8586
2	7.890	VB	0.1896	1769.36279	143.72514	50.1414

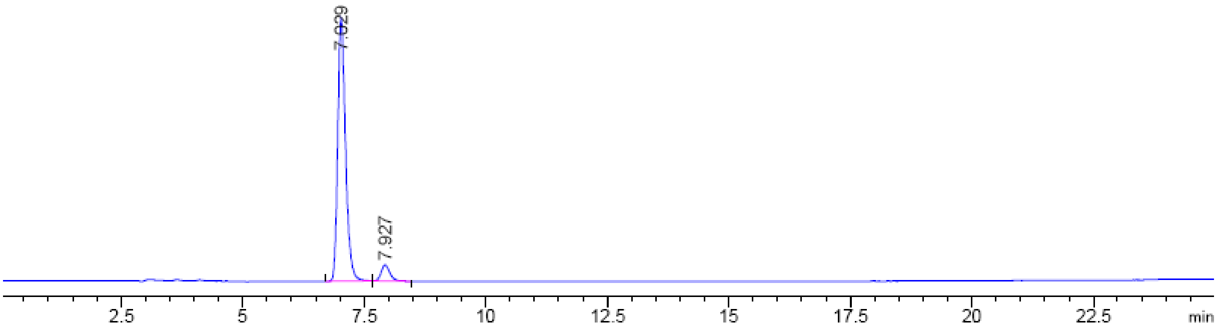

Peak #	RetTime [min]	Type	Width [min]	Area [mAU*s]	Height [mAU]	Area %
1	7.029	BB	0.1686	845.11700	75.48396	93.6246
2	7.927	BB	0.1876	57.54890	4.67470	6.3754

<u>(1a*R*,7b*R*)-2-(4-methoxybenzyl)-1a,2-dihydro-1*H*-cyclopropa[c]isoquinolin-3(7b*H*)-one (**4c**):</u>

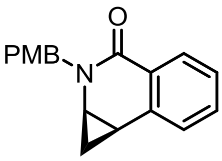

Obtained as a colourless oil in 70% yield (19.6 mg). **^{1}H NMR** (400 MHz, $CDCl_3$) δ = 8.22 (d, *J* = 7.8 Hz, 1H), 7.43 (dd, *J* = 10.5, 4.3 Hz, 1H), 7.38 – 7.29 (m, 4H), 6.87 (d, *J* = 8.6 Hz, 2H), 5.00 (d, *J* = 14.4 Hz, 1H), 4.70 (d, *J* = 14.4 Hz, 1H), 3.80 (s, 3H), 3.11 (ddd, *J* = 8.2, 6.6, 4.0 Hz, 1H), 2.29 – 2.21 (m, 1H), 1.31 – 1.23 (m, 1H), 0.18 (dd, *J* = 9.5, 5.4 Hz, 1H) ppm; **^{13}C{^{1}H} NMR** (101 MHz, $CDCl_3$) δ = 161.3, 159.2, 139.3, 132.0, 130.0, 129.4, 129.2, 128.3, 126.6, 125.7, 114.1, 55.4, 50.2, 34.2, 14.8, 14.4 ppm; **IR (ATR):** $\tilde{\nu}$ 2931, 1639, 1604, 1577, 1511, 1477, 1443, 1415, 1373, 1350, 1301, 1276, 1245, 1176, 1158, 1111, 1033, 986, 919, 846, 820, 761, 708, 692, 604, 586, 570, 551, 522 cm^{-1}; **HRMS (ESI)** calculated for $[C_{18}H_{18}NO_2]^+$ 280.1332, found 280.1338; **R_f:** 0.27 (pentane/EtOAc 3/1); **$[\alpha]_D^{20}$:** -63.3° (*c* = 0.1, $CHCl_3$). Chiral HPLC: (Chiralpak IA, 4.6 x 250 mm; 20% *i*-PrOH / hexane, 1.0 mL/min, 254 nm; t_r (minor) = 14.7 min, t_r (major) = 10.6 min), 92.3/7.7 *er.*

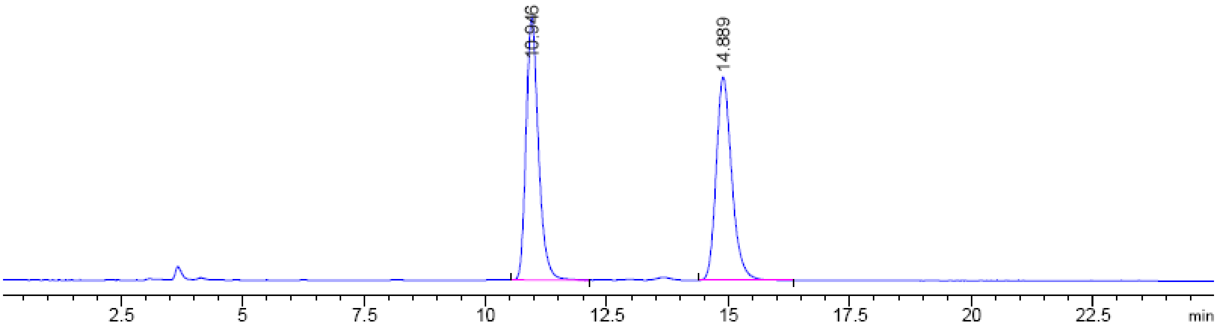

Peak #	RetTime [min]	Type	Width [min]	Area [mAU*s]	Height [mAU]	Area %
1	10.946	BB	0.2647	1472.66382	84.75246	49.8693
2	14.889	BB	0.3432	1480.38391	65.61608	50.1307

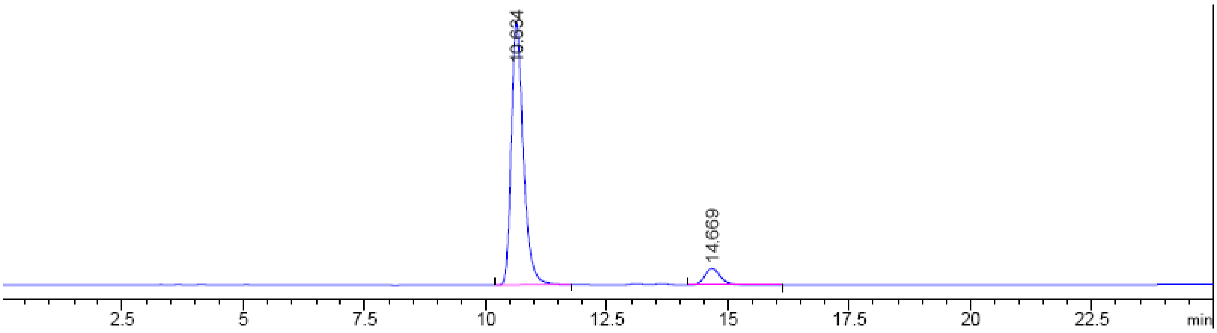

Peak #	RetTime [min]	Type	Width [min]	Area [mAU*s]	Height [mAU]	Area %
1	10.634	BB	0.2619	2099.87720	121.34597	92.3381
2	14.669	BB	0.3656	174.24155	7.43067	7.6619

(1a*R*,7b*R*)-2-(2,4,6-trimethylbenzyl)-1a,2-dihydro-1*H*-cyclopropa[c]isoquinolin-3(7b*H*)-one

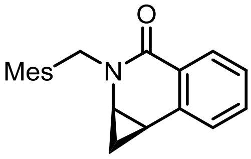

(4d): obtained as a colourless oil in 82% yield (24.0 mg) employing 5.0 mol% $Pd(dba)_2$ and 10 mol% **L2**. **^{1}H NMR** (400 MHz, $CDCl_3$) δ = 8.28 – 8.18 (m, 1H), 7.43 (td, *J* = 7.5, 1.4 Hz, 1H), 7.37 – 7.29 (m, 2H), 6.89 (s, 2H), 5.50 (d, *J* = 14.4 Hz, 1H), 4.50 (d, *J* = 14.4 Hz, 1H), 2.80 (ddd, *J* = 8.3, 6.6, 4.1 Hz, 1H), 2.35 (s, 6H), 2.29 (s, 3H), 2.20 – 2.14 (m, 1H), 1.18 (ddd, *J* = 9.7, 6.5, 5.1 Hz, 1H), 0.22 (dd, *J* = 9.6, 5.2 Hz, 1H) ppm; **^{13}C{^{1}H} NMR** (101 MHz, $CDCl_3$) δ = 161.1, 139.2, 138.7, 137.6, 132.0, 129.4, 129.3, 129.0, 128.2, 126.6, 125.6, 43.3, 31.7, 21.1, 20.2, 15.0, 14.2 ppm; **IR (ATR):** $\tilde{\nu}$ 2954, 2921, 1642, 1603, 1578, 1476, 1415, 1377, 1293, 1266, 1245, 1155, 761, 694 cm^{-1}; **HRMS (ESI)** calculated for $[C_{20}H_{22}NO]^+$ 292.1696, found 292.1697; **R_f:** 0.35 (pentane/EtOAc 5/1); **$[\alpha]_D^{20}$:** -70.0° (*c* = 0.1, $CHCl_3$, sample of 97.5/2.5 er). Chiral HPLC: (Chiralpak IC, 4.6 x 250 mm; 20% *i*-PrOH / hexane, 1.0 mL/min, 254 nm; t_r (minor) = 10.4 min, t_r (major) = 11.3 min), 91.6/8.4 *er.*

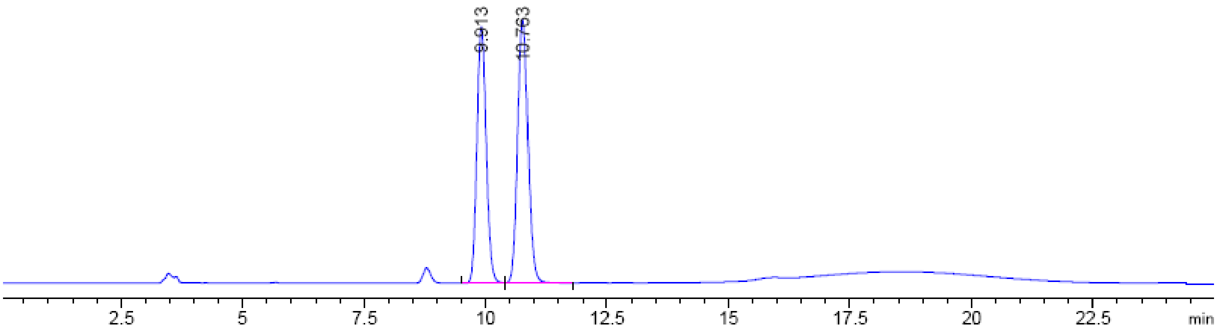

Peak #	RetTime [min]	Type	Width [min]	Area [mAU*s]	Height [mAU]	Area %
1	9.913	BB	0.2056	869.29059	66.01301	46.9465
2	10.763	BB	0.2231	982.36963	67.78742	53.0535

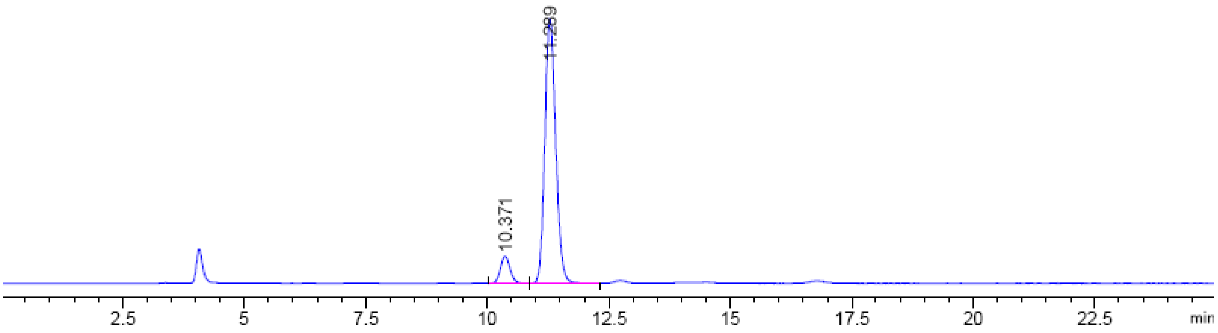

Peak #	RetTime [min]	Type	Width [min]	Area [mAU*s]	Height [mAU]	Area %
1	10.371	BB	0.2168	343.31189	24.59543	8.3685
2	11.289	BB	0.2428	3759.09595	239.95052	91.6315

(1a*R*,7b*R*)-2-(4-methoxybenzyl)-1a-(3-phenylpropyl)-1a,2-dihydro-1*H*-cyclopropa[c]isoquinolin-3(7b*H*)-one (**4f**): obtained as a colourless oil in 98% yield (39.1 mg). **^{1}H NMR** (400 MHz, $CDCl_3$) δ = 8.24 – 8.16 (m, 1H), 7.45 – 7.39 (m, 1H), 7.33 – 7.24 (m, 6H), 7.17 (dd, *J* = 8.4, 6.3 Hz, 1H), 7.05 (d, *J* = 7.0 Hz, 2H), 6.86 – 6.79 (m, 2H), 5.29 (d, *J* = 15.5 Hz, 1H), 4.38 (d, *J* = 14.8 Hz, 1H), 3.79 (s, 3H), 2.56 – 2.45 (m, 2H), 2.43 – 2.35 (m, 1H), 1.94 (dd, *J* = 9.8, 6.1 Hz, 1H), 1.69 – 1.61 (m, 2H), 1.09 (dd, *J* = 9.9, 5.0 Hz, 1H), 0.83 (ddd, *J* = 14.9, 10.9, 6.6 Hz, 1H), 0.29 (dd, *J* = 5.9, 5.2 Hz, 1H) ppm; **^{13}C{^{1}H} NMR** (101 MHz, $CDCl_3$) δ = 163.2, 158.9, 141.9, 139.0, 132.1, 130.1, 130.0, 129.4, 128.5, 128.0, 126.6, 126.0, 125.3, 113.8, 55.4, 46.7, 42.6, 35.9, 35.4, 27.8, 21.4, 21.3 ppm; **IR (ATR):** $\tilde{\nu}$ 2936, 1640, 1605, 1581, 1512, 1495, 1474, 1403, 1381, 1337, 1302, 1248, 1176, 1035, 752, 699 cm^{-1}; **HRMS (ESI)** calculated for $[C_{27}H_{28}NO_2]^+$ 398.2115, found 398.2106; **R_f:** 0.42 (pentane/EtOAc 3/1); **$[\alpha]_D^{20}$:** -85.0° (*c* = 0.1, $CHCl_3$). Chiral HPLC: (Chiralpak IB, 4.6 x 250 mm; 20% *i*-PrOH / hexane, 1.0 mL/min, 254 nm; t_r (minor) = 8.2 min, t_r (major) = 9.3 min), 94.4/5.6 *er.*

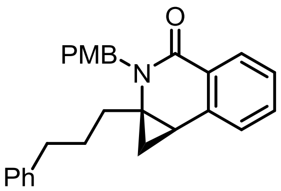

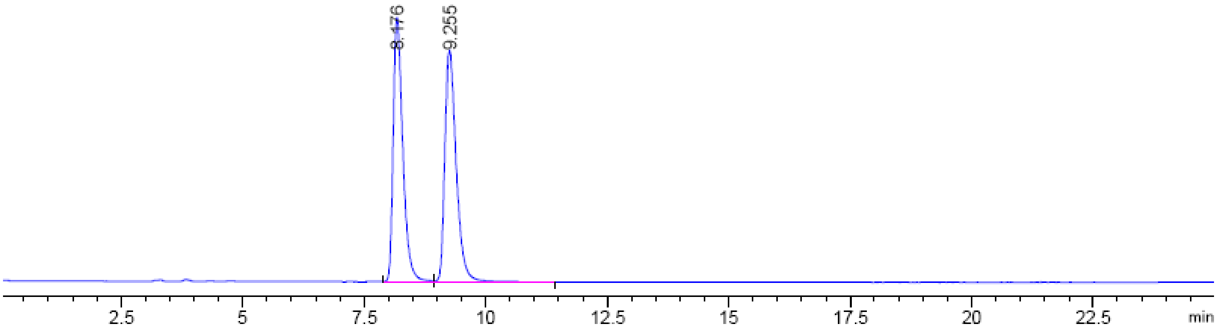

Peak #	RetTime [min]	Type	Width [min]	Area [mAU*s]	Height [mAU]	Area %
1	8.176	VV	0.2141	2483.37158	178.71544	49.9088
2	9.255	VB	0.2412	2492.44287	156.99364	50.0912

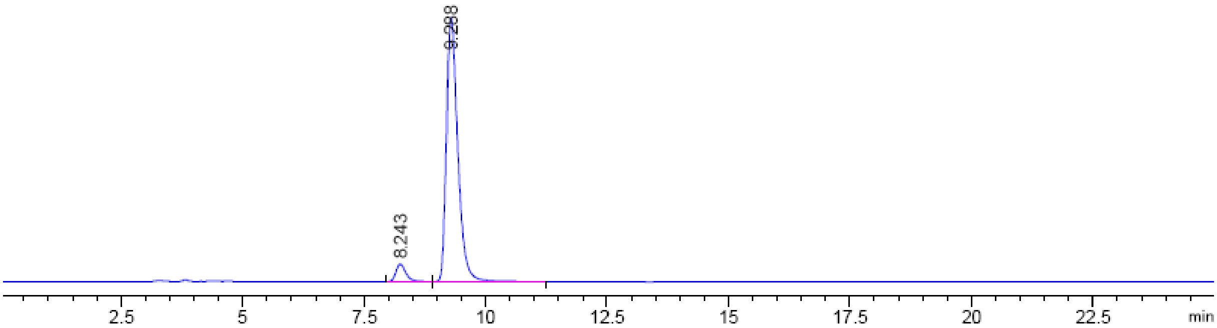

Peak #	RetTime [min]	Type	Width [min]	Area [mAU*s]	Height [mAU]	Area %
1	8.243	BB	0.2157	118.69316	8.25506	5.6323
2	9.288	BB	0.2423	1988.66467	124.52590	94.3677

(1a*S*,7b*R*)-1a-isopropyl-2-methyl-1a,2-dihydro-1*H*-cyclopropa[c]isoquinolin-3(7b*H*)-one (**4g**):

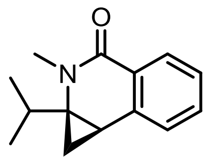

Obtained as a colourless oil in 85% yield (18.2 mg) employing 5.0 mol% $Pd(dba)_2$ and 10 mol% **L2**. **^{1}H NMR** (400 MHz, $CDCl_3$) δ = 8.15 – 8.09 (m, 1H), 7.42 (td, *J* = 7.5, 1.4 Hz, 1H), 7.35 (d, *J* = 6.6 Hz, 1H), 7.29 (td, *J* = 7.8, 1.3 Hz, 1H), 3.20 (s, 3H), 2.52 – 2.44 (m, 1H), 2.16 (dd, *J* = 10.2, 6.4 Hz, 1H), 1.42 (dd, *J* = 10.2, 5.0 Hz, 1H), 0.99 (d, *J* = 6.6 Hz, 3H), 0.77 (d, *J* = 6.9 Hz, 3H), 0.48 (dd, *J* = 6.3, 5.1 Hz, 1H) ppm; **^{13}C{^{1}H} NMR** (101 MHz, $CDCl_3$) δ = 163.1, 138.8, 131.9, 128.9, 128.2, 126.6, 125.4, 48.9, 31.7, 28.6, 19.6, 17.1, 17.0, 16.9 ppm; **IR (ATR):** $\tilde{\nu}$ 2964, 2929, 1642, 1603, 1581, 1483, 1415, 1374, 1275, 1141, 1110, 1031, 748, 695 cm^{-1}; **HRMS (ESI)** calculated for $[C_{14}H_{18}NO]^+$ 216.1383, found 216.1379; **R_f:** 0.31 (pentane/EtOAc 3/1); **$[\alpha]_D^{20}$:** -61.7° (*c* = 0.1, $CHCl_3$). Chiral HPLC: (Chiralpak IA, 4.6 x 250 mm; 20% *i*-PrOH / hexane, 1.0 mL/min, 254 nm; t_r (minor) = 6.7 min, t_r (major) = 7.3 min), 93.5/6.5 *er.*

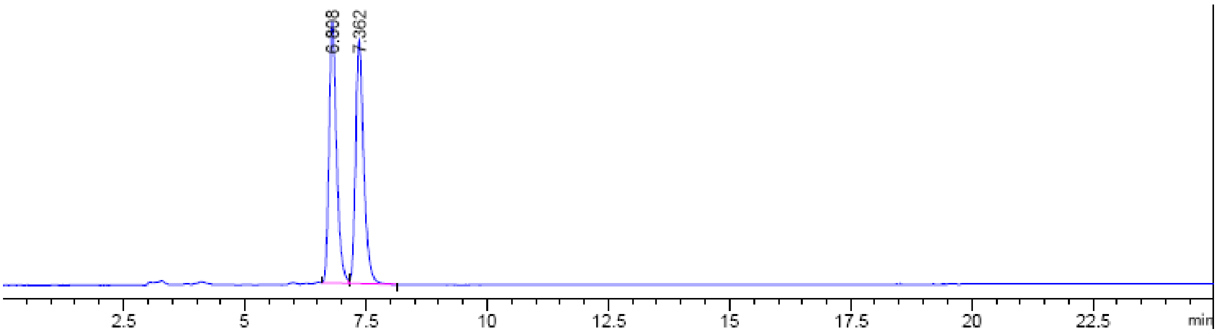

Peak #	RetTime [min]	Type	Width [min]	Area [mAU*s]	Height [mAU]	Area %
1	6.808	BV	0.1642	267.56415	24.72706	49.7582
2	7.362	VB	0.1767	270.16415	23.05650	50.2418

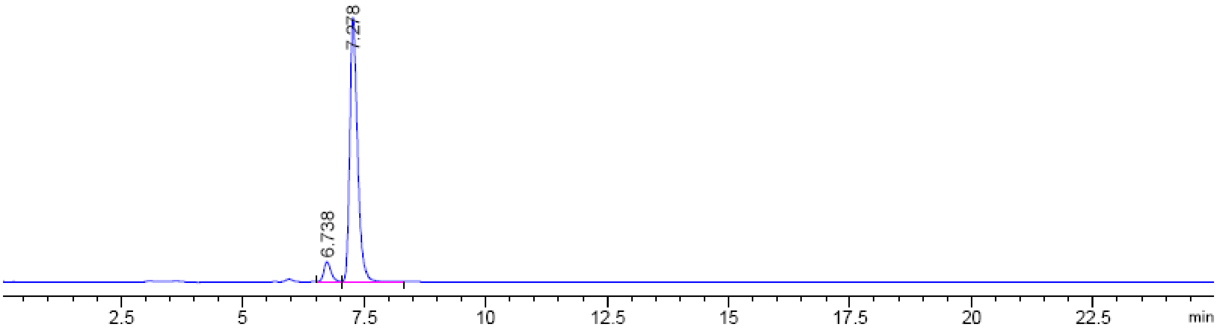

Peak #	RetTime [min]	Type	Width [min]	Area [mAU*s]	Height [mAU]	Area %
1	6.738	BV	0.1565	145.12923	14.28806	6.4681
2	7.278	VB	0.1680	2098.63940	188.32515	93.5319

(1a*R*,7b*R*)-2-methyl-1a-phenyl-1a,2-dihydro-1*H*-cyclopropa[c]isoquinolin-3(7b*H*)-one (**4h**):

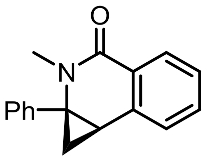

Obtained as a colourless oil in 99% yield (24.8 mg) after 24 h reaction time. **^{1}H NMR** (400 MHz, $CDCl_3$) δ = 8.27 – 8.16 (m, 1H), 7.46 (td, *J* = 7.5, 1.4 Hz, 1H), 7.40 – 7.27 (m, 7H), 2.96 (s, 3H), 2.30 (dd, *J* = 10.2, 6.5 Hz, 1H), 2.12 (dd, *J* = 10.2, 5.2 Hz, 1H), 0.80 (dd, *J* = 6.4, 5.3 Hz, 1H) ppm; **^{13}C{^{1}H} NMR** (101 MHz, $CDCl_3$) δ = 162.7, 139.8, 138.2, 132.1, 129.1, 129.0, 128.1, 128.0, 126.9, 125.1, 47.4, 32.9, 25.9, 17.7 ppm; **IR (ATR):** $\tilde{\nu}$ 3058, 3029, 1640, 1602, 1579, 1475, 1447, 1416, 1393, 1367, 1307, 1195, 1156, 1100, 1082, 1047, 1030, 968, 937, 921, 906, 886, 797, 761, 747, 695, 679, 606, 580, 542, 514, 496, 467, 412 cm^{-1}; **HRMS (ESI)** calculated for $[C_{17}H_{16}NO]^+$ 250.1226, found 250.1228; **R_f:** 0.35 (pentane/EtOAc 3/1); **$[\alpha]_D^{20}$:** +36.7° (*c* = 0.1, $CHCl_3$). Chiral HPLC: (Chiralpak IB, 4.6 x 250 mm; 20% *i*-PrOH / hexane, 1.0 mL/min, 254 nm; t_r (minor) = 6.7 min, t_r (major) = 6.2 min), 91.3/8.7 *er.*

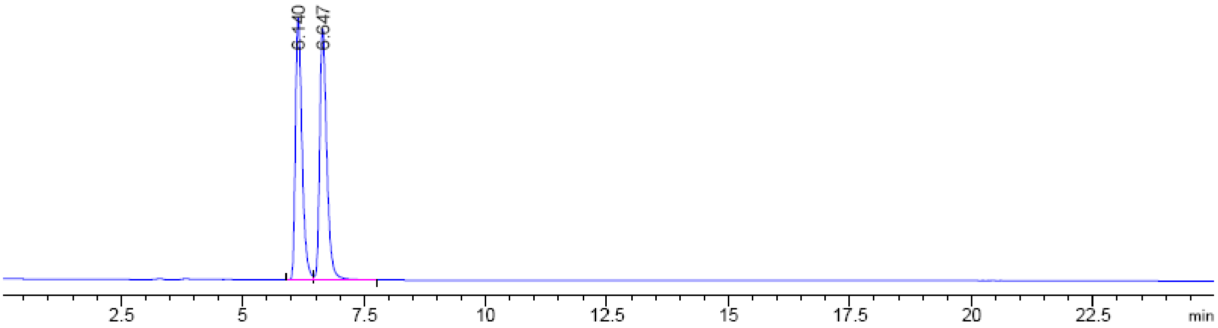

Peak #	RetTime [min]	Type	Width [min]	Area [mAU*s]	Height [mAU]	Area %
1	6.140	BV	0.1422	3590.85229	380.20032	49.3084
2	6.647	VB	0.1547	3691.59009	362.79193	50.6916

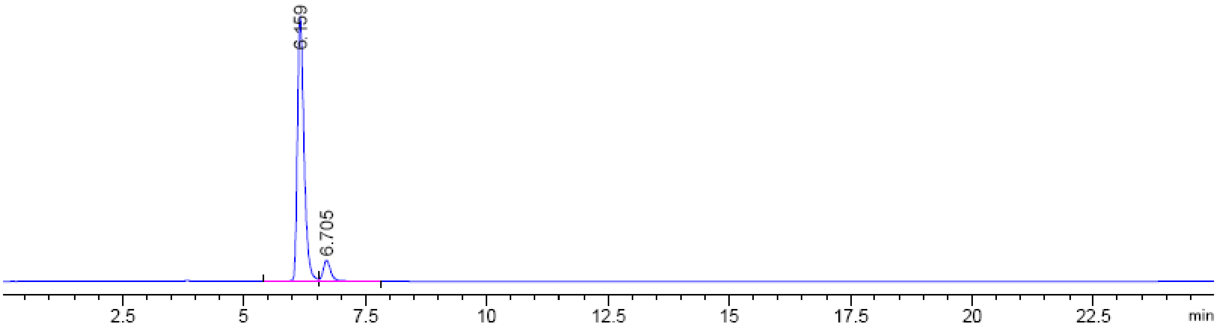

Peak #	RetTime [min]	Type	Width [min]	Area [mAU*s]	Height [mAU]	Area %
1	6.159	BV	0.1435	3461.68311	362.27420	91.3433
2	6.705	VV	0.1741	328.06680	28.53560	8.6567

(1a*R*,7b*R*)-1a-(4-chlorophenyl)-2-(4-methoxybenzyl)-1a,2-dihydro-1*H*-cyclopropa[c]isoquinolin-3(7b*H*)-one (**4i**): obtained as a yellow oil in 99% yield (38.5 mg) after 24 h reaction time employing 5.0 mol% Pd(dba)$_2$ and 10 mol% **L2**. **^{1}H NMR** (400 MHz, CDCl$_3$) δ = 8.29 (d, *J* = 7.7 Hz, 1H), 7.46 (td, *J* = 7.4, 1.1 Hz, 1H), 7.38 (dd, *J* = 11.0, 4.0 Hz, 1H), 7.28 (dt, *J* = 11.6, 8.6 Hz, 5H), 7.05 (d, *J* = 8.6 Hz, 2H), 6.75 (d, *J* = 8.6 Hz, 2H), 5.43 (d, *J* = 14.5 Hz, 1H), 3.88 (d, *J* = 14.5 Hz, 1H), 3.77 (s, 3H), 2.13 (dd, *J* = 10.2, 6.7 Hz, 1H), 1.93 (dd, *J* = 10.3, 5.6 Hz, 1H), 0.51 (t, *J* = 6.1 Hz, 1H) ppm; **^{13}C{^{1}H} NMR** (101 MHz, CDCl$_3$) δ = 162.4, 158.9, 138.3, 138.0, 133.9, 132.3, 130.4, 130.0, 129.5, 129.3, 129.0, 128.1, 127.0, 125.2, 113.6, 55.3, 47.3, 45.2, 24.4, 19.2 ppm; **IR (ATR):** $\tilde{\nu}$ 2954, 2929, 1643, 1605, 1582, 1511, 1489, 1469, 1439, 1397, 1378, 1356, 1323, 1303, 1273, 1247, 1176, 1163, 1110, 1093, 1034, 1013, 979, 830, 807, 751, 692, 570, 523 cm^{-1}; **HRMS (ESI)** calculated for $[C_{24}H_{21}ClNO_2]^+$ 390.1255, found 390.1255; **R$_f$:** 0.33 (pentane/EtOAc 5/1); **$[\alpha]_D^{20}$:** +18.3° (*c* = 0.1, CHCl$_3$). Chiral HPLC: (Chiralpak IB, 4.6 x 250 mm; 20% *i*-PrOH / hexane, 1.0 mL/min, 254 nm; t_r (minor) = 10.3 min, t_r (major) = 11.3 min), 95.2/4.8 *er.*

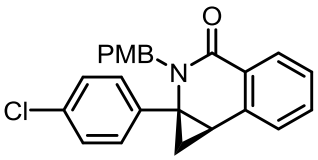

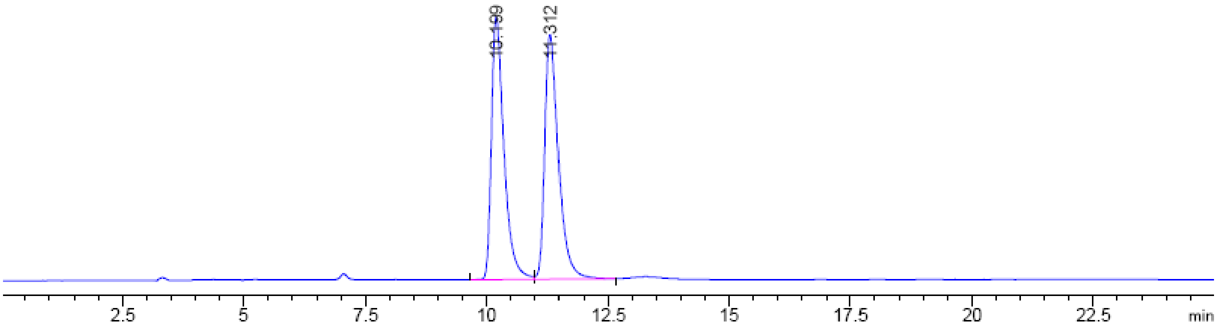

Peak #	RetTime [min]	Type	Width [min]	Area [mAU*s]	Height [mAU]	Area %
1	10.199	BV	0.2734	1899.98975	104.83179	49.9476
2	11.312	VB	0.2935	1903.97534	97.62679	50.0524

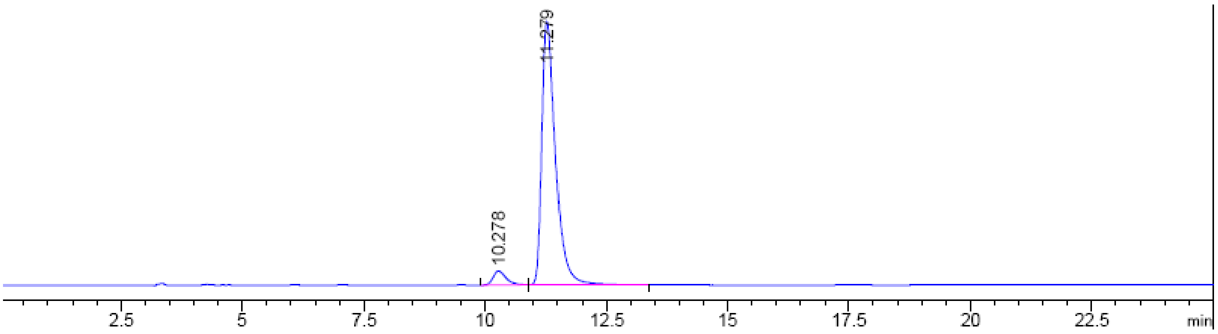

Peak #	RetTime [min]	Type	Width [min]	Area [mAU*s]	Height [mAU]	Area %
1	10.278	BB	0.2781	144.21025	7.85796	4.7815
2	11.279	BB	0.2944	2871.77026	146.67047	95.2185

(1a*S*,7b*R*)-1a-benzyl-2-methyl-1a,2-dihydro-1*H*-cyclopropa[c]isoquinolin-3(7b*H*)-one (**4j**):

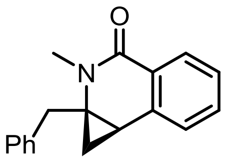

Obtained as a pale yellow oil in 89% yield (23.5 mg). **^{1}H NMR** (400 MHz, $CDCl_3$) δ = 8.12 (d, *J* = 7.7 Hz, 1H), 7.44 (td, *J* = 7.5, 1.0 Hz, 1H), 7.38 – 7.15 (m, 7H), 3.67 (d, *J* = 15.8 Hz, 1H), 3.20 (s, 3H), 2.60 (d, *J* = 15.8 Hz, 1H), 2.33 (dd, *J* = 9.9, 6.1 Hz, 1H), 1.32 (dd, *J* = 9.9, 4.9 Hz, 1H), 0.59 – 0.52 (m, 1H) ppm; **^{13}C{^{1}H} NMR** (101 MHz, $CDCl_3$) δ = 162.7, 138.3, 136.8, 132.0, 129.2, 128.8, 128.7, 128.1, 126.9, 126.8, 125.2, 43.2, 41.7, 31.2, 22.8, 17.9 ppm; **IR (ATR):** $\tilde{\nu}$ 3028, 2922, 1639, 1604, 1579, 1479, 1454, 1438, 1413, 1394, 1370, 1279, 1141, 1049, 1030, 757, 743, 696, 615, 574, 559 cm^{-1}; **HRMS (ESI)** calculated for $[C_{18}H_{18}NO]^+$ 264.1383, found 264.1379; **R$_f$:** 0.19 (pentane/EtOAc 3/1); **$[\alpha]_D^{20}$:** -218.3° (*c* = 0.1, $CHCl_3$). Chiral HPLC: (Chiralpak IA, 4.6 x 250 mm; 20% *i*-PrOH / hexane, 1.0 mL/min, 254 nm; t_r (minor) = 8.1 min, t_r (major) = 7.4 min), 92.2/7.8 *er*.

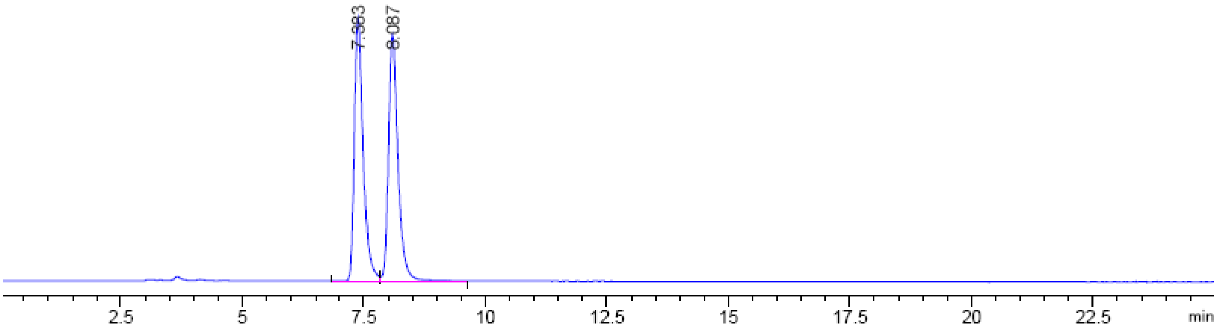

Peak #	RetTime [min]	Type	Width [min]	Area [mAU*s]	Height [mAU]	Area %
1	7.383	BV	0.1905	806.64752	64.22442	49.4591
2	8.087	VB	0.2057	824.28973	60.23296	50.5409

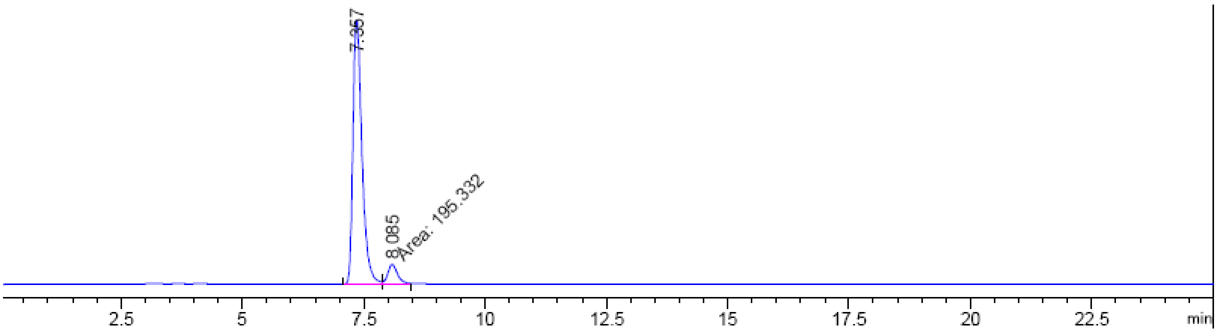

Peak #	RetTime [min]	Type	Width [min]	Area [mAU*s]	Height [mAU]	Area %
1	7.357	VV	0.1865	2321.47534	187.36879	92.2389
2	8.085	MF	0.2385	195.33212	13.65265	7.7611

(1a*R*,7b*R*)-ethyl 2-methyl-3-oxo-1a,2,3,7b-tetrahydro-1*H*-cyclopropa[c]isoquinoline-1a-carboxylate (**4k**): obtained as a colourless oil in 99% yield (25.0 mg).

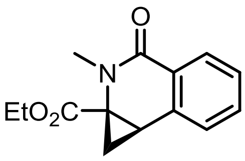

^{1}H NMR (400 MHz, $CDCl_3$) δ = 8.15 (d, *J* = 7.9 Hz, 1H), 7.50 – 7.43 (m, 1H), 7.39 – 7.32 (m, 2H), 4.34 – 4.16 (m, 2H), 3.25 (s, 3H), 2.74 (dd, *J* = 10.4, 7.2 Hz, 1H), 2.17 (dd, *J* = 10.4, 4.6 Hz, 1H), 1.31 (t, *J* = 7.1 Hz, 3H), 0.88 (dd, *J* = 7.2, 4.6 Hz, 1H) ppm; **^{13}C{^{1}H} NMR** (101 MHz, $CDCl_3$) δ = 170.1, 162.3, 136.4, 132.2, 129.2, 128.2, 127.6, 125.2, 62.1, 45.0, 34.8, 26.5, 20.1, 14.3 ppm; **IR (ATR):** $\tilde{\nu}$ 2981, 1729, 1652, 1605, 1583, 1471, 1445, 1421, 1367, 1336, 1288, 1249, 1202, 1176, 1135, 1107, 1077, 1053, 1021, 858, 750, 725, 694, 676, 565 cm^{-1}; **HRMS (ESI)** calculated for $[C_{14}H_{16}NO_3]^+$ 246.1125, found 246.1126; **R_f:** 0.23 (pentane/EtOAc 3/1); **$[\alpha]_D^{20}$:** +115.0° (*c* = 0.1, $CHCl_3$). Chiral HPLC: (Chiralpak IA, 4.6 x 250 mm; 20% *i*-PrOH / hexane, 1.0 mL/min, 254 nm; t_r (minor) = 19.0 min, t_r (major) = 12.7 min), 91.5/8.5 *er.*

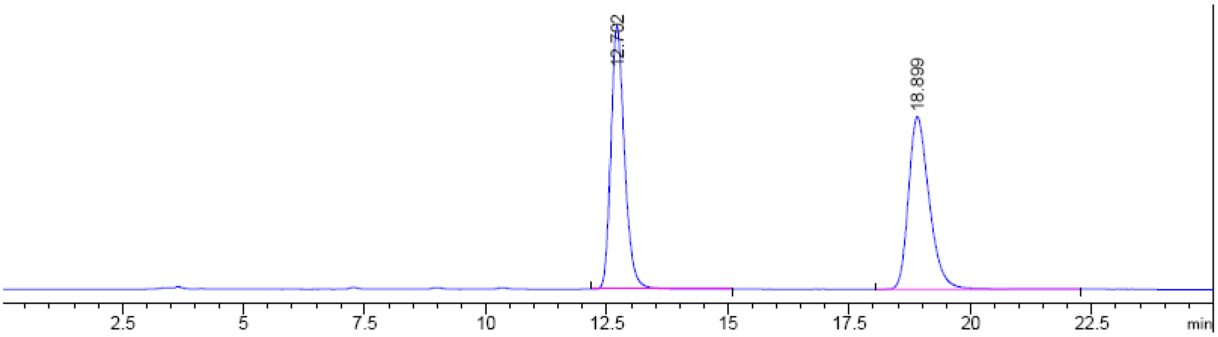

Peak #	RetTime [min]	Type	Width [min]	Area [mAU*s]	Height [mAU]	Area %
1	12.702	BB	0.2953	3067.13892	157.41638	49.7540
2	18.899	BB	0.4578	3097.47339	103.30487	50.2460

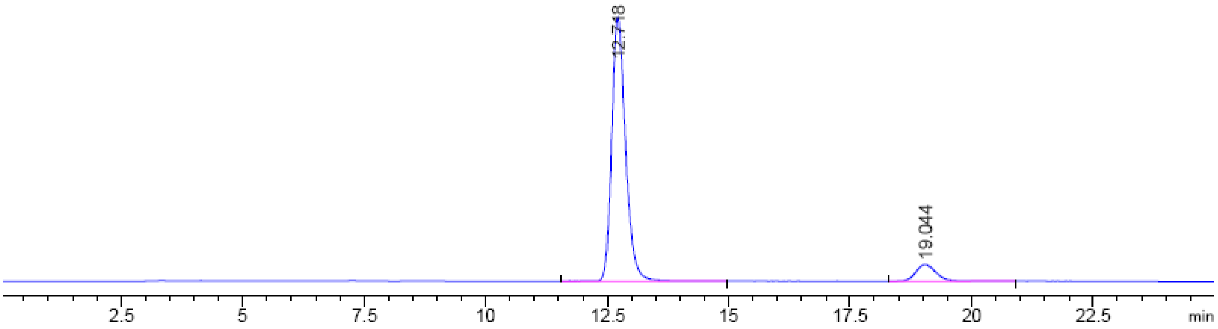

Peak #	RetTime [min]	Type	Width [min]	Area [mAU*s]	Height [mAU]	Area %
1	12.718	BB	0.3038	3425.18945	172.36316	91.5262
2	19.044	BB	0.4444	317.11465	10.99726	8.4738

(1a*R*,7b*R*)-2-methyl-3-oxo-1a,2,3,7b-tetrahydro-1*H*-cyclopropa[c]isoquinoline-1a-carbonitrile

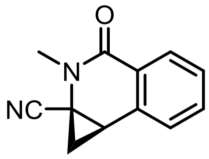

(4l): obtained as a white solid in 89% yield (18.9 mg) employing 5.0 mol% $Pd(dba)_2$ and 10 mol% **L2**. **^{1}H NMR** (400 MHz, $CDCl_3$) δ = 8.22 – 8.08 (m, 1H), 7.56 – 7.50 (m, 1H), 7.45 – 7.37 (m, 2H), 3.38 (s, 3H), 3.00 (dd, *J* = 10.5, 7.4 Hz, 1H), 1.99 (dd, *J* = 10.5, 5.6 Hz, 1H), 0.98 (dd, *J* = 7.4, 5.6 Hz, 1H) ppm; **^{13}C{^{1}H} NMR** (101 MHz, $CDCl_3$) δ = 160.8, 134.6, 132.9, 129.6, 128.4, 128.3, 124.4, 117.7, 33.3, 23.7, 21.6 ppm; **IR (ATR):** $\tilde{\nu}$ 2920, 2243, 1658, 1605, 1582, 1473, 1420, 1392, 1369, 1324, 1150, 1052, 1033, 753, 692 cm^{-1}; **HRMS (ESI)** calculated for $[C_{12}H_{11}N_2O]^+$ 199.0866, found 199.0865; **R_f:** 0.23 (pentane/EtOAc 3/1); **m.p.:** 163.0-165.7 °C; **$[\alpha]_D^{20}$:** +55.0° (*c* = 0.1, $CHCl_3$). Chiral HPLC: (Chiralpak IA, 4.6 x 250 mm; 20% *i*-PrOH / hexane, 1.0 mL/min, 254 nm; t_r (minor) = 18.3 min, t_r (major) = 9.6 min), 93.5/6.5 *er.*

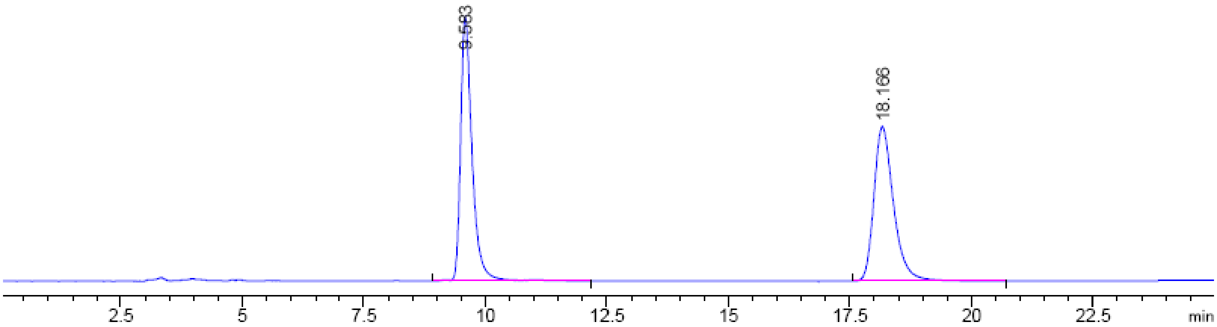

Peak #	RetTime [min]	Type	Width [min]	Area [mAU*s]	Height [mAU]	Area %
1	9.583	BB	0.2559	803.12640	47.82684	50.1942
2	18.166	BB	0.4317	796.91278	28.03281	49.8058

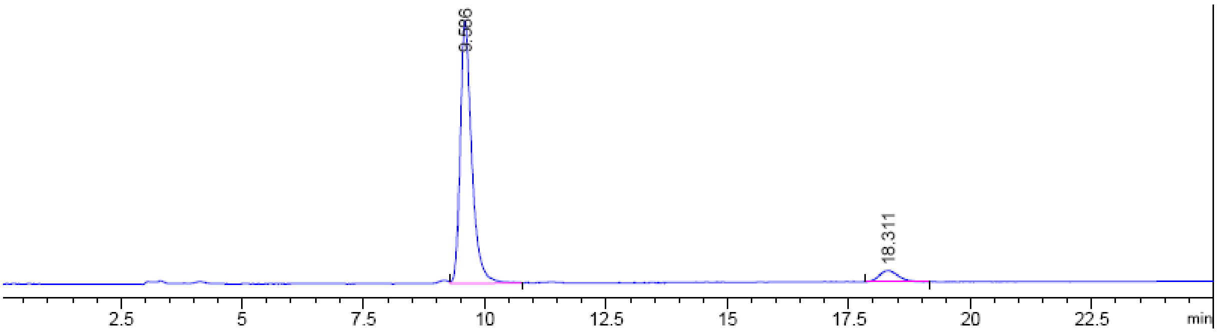

Peak #	RetTime [min]	Type	Width [min]	Area [mAU*s]	Height [mAU]	Area %
1	9.586	VB	0.2451	463.49368	28.29638	93.5375
2	18.311	BB	0.3894	32.02285	1.15533	6.4625

(1a*R*,7b*R*)-6-chloro-2-(4-methoxybenzyl)-1a-(3-phenylpropyl)-1a,2-dihydro-1*H*-cyclopropa[c]-isoquinolin-3(7b*H*)-one (**4m**): obtained as a yellow oil in 98% yield (42.5 mg). **^{1}H NMR** (400 MHz, $CDCl_3$) δ = 8.17 (m, 1H), 7.37 (dd, *J* = 8.1, 2.2 Hz, 1H), 7.29 – 7.15 (m, 6H), 7.05 (d, *J* = 7.9 Hz, 2H), 6.82 (d, *J* = 8.6 Hz, 2H), 5.27 (d, *J* = 14.7 Hz, 1H), 4.33 (d, *J* = 14.7 Hz, 1H), 3.79 (s, 3H), 2.56 – 2.35 (m, 3H), 1.90 (dd, *J* = 9.8, 6.1 Hz, 1H), 1.74 – 1.60 (m, 2H), 1.10 (dd, *J* = 9.8, 5.1 Hz, 1H), 0.87 – 0.78 (m, 1H), 0.26 (t, *J* = 5.6 Hz, 1H) ppm; **^{13}C{^{1}H} NMR** (101 MHz, $CDCl_3$) δ = 162.0, 159.0, 141.7, 137.3, 132.7, 132.1, 130.1, 129.7, 129.5, 129.4, 128.5, 128.5, 126.9, 126.1, 113.9, 55.4, 46.7, 42.7, 35.8, 35.4, 27.8, 21.4, 20.9 ppm; **IR (ATR):** $\tilde{\nu}$ 2935, 1643, 1611, 1599, 1512, 1487, 1459, 1425, 1377, 1334, 1302, 1248, 1176, 1033, 819, 750, 700 cm^{-1}; **HRMS (ESI)** calculated for $[C_{27}H_{27}ClNO_2]^+$ 432.1725, found 432.1731; **R_f:** 0.38 (pentane/EtOAc 5/1); **$[\alpha]_D^{20}$:** -57.8° (*c* = 0.1, $CHCl_3$). Chiral HPLC: (Chiralpak IB, 4.6 x 250 mm; 20% *i*-PrOH / hexane, 1.0 mL/min, 290 nm; t_r (minor) = 8.3 min, t_r (major) = 9.6 min), 94.2/5.8 *er.*

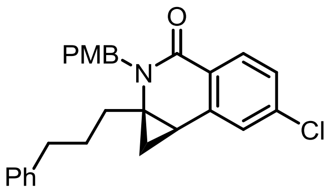

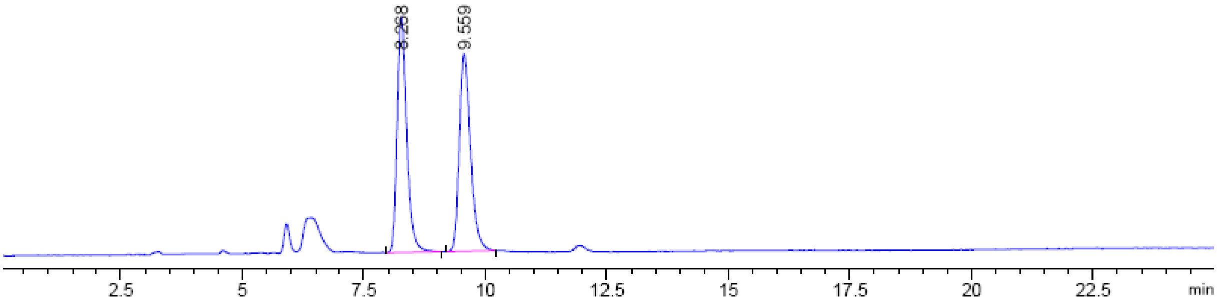

Peak #	RetTime [min]	Type	Width [min]	Area [mAU*s]	Height [mAU]	Area %
1	8.268	BB	0.2118	195.69644	14.11291	50.3414
2	9.559	BB	0.2500	193.04208	11.85233	49.6586

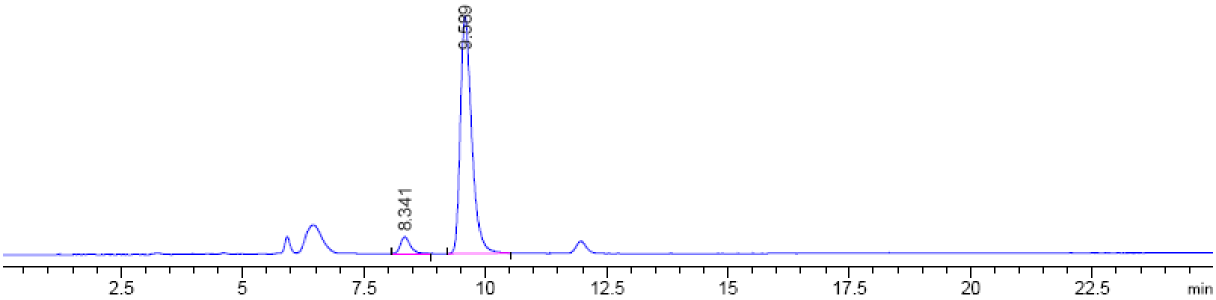

Peak #	RetTime [min]	Type	Width [min]	Area [mAU*s]	Height [mAU]	Area %
1	8.341	BB	0.2108	31.11010	2.20303	5.7618
2	9.589	BB	0.2494	508.82834	31.01768	94.2382

(1a*R*,7b*R*)-5-methoxy-2-(4-methoxybenzyl)-1a-(3-phenylpropyl)-1a,2-dihydro-1*H*-cyclopropa-[c]isoquinolin-3(7b*H*)-one (**4n**): obtained as a colourless oil in 99% yield (42.5 mg). **^{1}H NMR** (400 MHz, $CDCl_3$) δ = 7.80 (d, *J* = 2.7 Hz, 1H), 7.37 – 7.21 (m, 6H), 7.11 (d, *J* = 7.1 Hz, 2H), 7.07 (dd, *J* = 8.3, 2.8 Hz, 1H), 6.89 (d, *J* = 8.6 Hz, 2H), 5.32 (d, *J* = 14.7 Hz, 1H), 4.46 (d, *J* = 14.7 Hz, 1H), 3.92 (s, 3H), 3.86 (s, 3H), 2.62 – 2.40 (m, 3H), 1.97 (dd, *J* = 9.6, 6.1 Hz, 1H), 1.76 – 1.66 (m, 2H), 1.10 (dd, *J* = 9.7, 5.0 Hz, 1H), 0.93 – 0.85 (m, 1H), 0.29 (t, *J* = 5.5 Hz, 1H) ppm; **^{13}C{^{1}H} NMR** (101 MHz, $CDCl_3$) δ = 163.2, 158.9, 158.5, 141.9, 131.2, 130.0, 129.1, 128.5, 128.5, 126.3, 126.0, 120.2, 113.8, 112.0, 55.7, 55.4, 46.8, 42.4, 36.0, 35.4, 27.8, 20.9, 20.7 ppm; **IR (ATR):** $\tilde{\nu}$ 2933, 1640, 1608, 1581, 1504, 1461, 1441, 1428, 1380, 1332, 1300, 1276, 1244, 1176, 1150, 1102, 1034, 829, 780, 752, 701 cm^{-1}; **HRMS (ESI)** calculated for $[C_{28}H_{30}NO_3]^+$ 428.2220, found 428.2215; **R_f:** 0.30 (pentane/EtOAc 3/1); **$[\alpha]_D^{20}$:** -88.3° (*c* = 0.1, $CHCl_3$). Chiral HPLC: (Chiralpak IB, 4.6 x 250 mm; 20% *i*-PrOH / hexane, 1.0 mL/min, 254 nm; t_r (minor) = 8.5 min, t_r (major) = 9.5 min), 93.9/6.1 *er.*

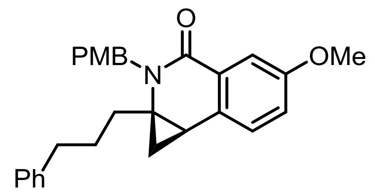

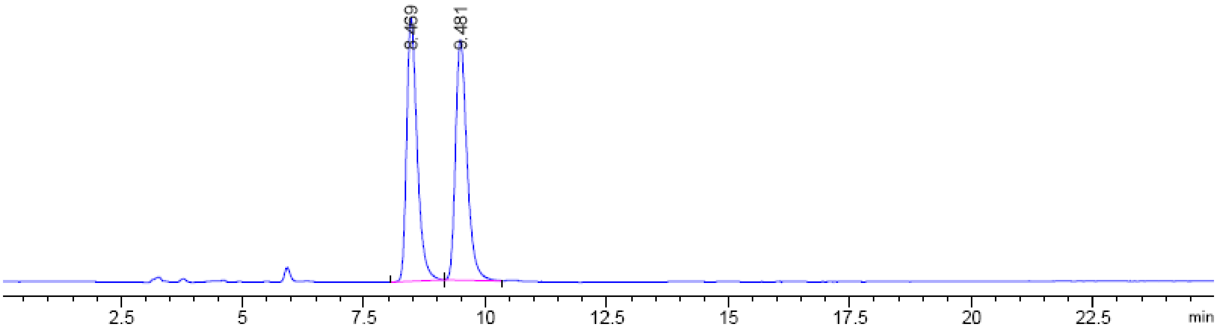

Peak #	RetTime [min]	Type	Width [min]	Area [mAU*s]	Height [mAU]	Area %
1	8.469	BB	0.2284	446.83670	29.54929	50.0708
2	9.481	BB	0.2525	445.57285	27.00421	49.9292

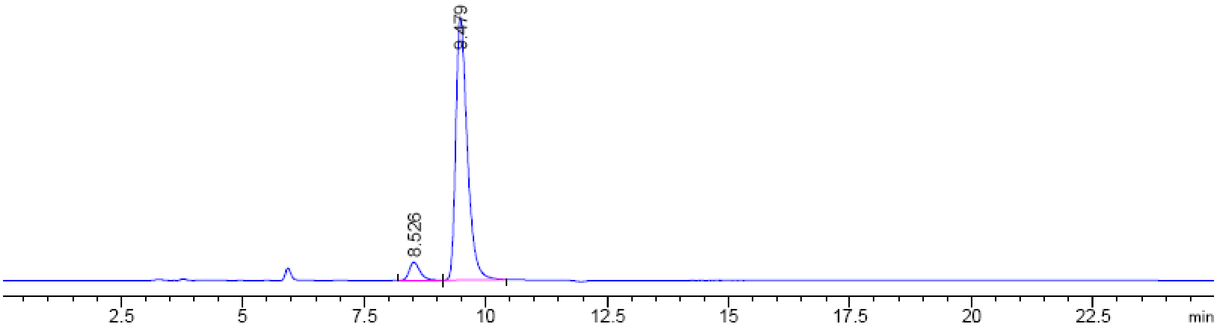

Peak #	RetTime [min]	Type	Width [min]	Area [mAU*s]	Height [mAU]	Area %
1	8.526	BB	0.2342	102.56538	6.63861	6.0764
2	9.479	BB	0.2510	1585.36414	95.84155	93.9236

(1a*R*,7b*R*)-2-(4-methoxybenzyl)-1a-(3-phenylpropyl)-1a,2-dihydro-1*H*-cyclopropa[c][2,6]-naphthyridin-3(7b*H*)-one (**4o**): obtained as a yellow oil in 99% yield (39.6 mg). **^{1}H NMR** (400 MHz, $CDCl_3$) δ = 8.65 (s, 1H), 8.60 (d, *J* = 5.1 Hz, 1H), 7.95 (d, *J* = 5.1 Hz, 1H), 7.30 – 7.16 (m, 6H), 7.08 – 7.03 (m, 2H), 6.85 – 6.80 (m, 2H), 5.29 (d, *J* = 14.7 Hz, 1H), 4.32 (d, *J* = 14.7 Hz, 1H), 3.79 (s, 3H), 2.52 (tt, *J* = 10.1, 6.3 Hz, 2H), 2.45 – 2.37 (m, 1H), 1.96 (dd, *J* = 9.9, 6.1 Hz, 1H), 1.69 – 1.59 (m, 2H), 1.18 (dd, *J* = 9.9, 5.3 Hz, 1H), 0.87 (ddd, *J* = 15.0, 10.7, 6.7 Hz, 1H), 0.30 (t, *J* = 5.7 Hz, 1H) ppm; **^{13}C{^{1}H} NMR** (101 MHz, $CDCl_3$) δ = 161.4, 159.1, 150.0, 148.5, 141.6, 133.3, 131.8, 130.1, 129.2, 128.6, 128.5, 126.2, 121.8, 113.9, 55.4, 46.7, 43.0, 35.6, 35.3, 27.8, 21.5, 18.6 ppm; **IR (ATR):** $\tilde{\nu}$ 2935, 1645, 1612, 1571, 1512, 1497, 1460, 1425, 1395, 1380, 1359, 1336, 1302, 1247, 1213, 1176, 1112, 1032, 848, 815, 751, 700 cm^{-1}; **HRMS (ESI)** calculated for $[C_{26}H_{27}N_2O_2]^+$ 399.2067, found 399.2067; **R_f:** 0.13 (pentane/EtOAc 1/1); **$[\alpha]_D^{20}$:** -153.3° (*c* = 0.1, $CHCl_3$). Chiral HPLC: (Chiralpak IA, 4.6 x 250 mm; 20% *i*-PrOH / hexane, 1.0 mL/min, 254 nm; t_r (minor) = 12.0 min, t_r (major) = 13.9 min), 96.4/3.6 *er.*

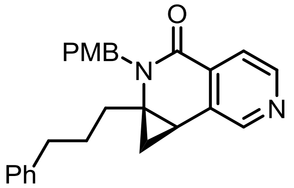

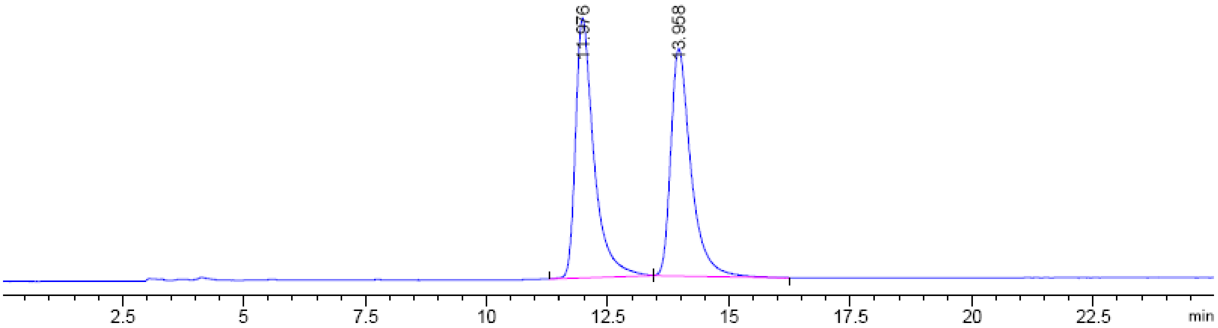

Peak #	RetTime [min]	Type	Width [min]	Area [mAU*s]	Height [mAU]	Area %
1	11.976	BB	0.3918	680.52460	25.62887	51.1499
2	13.958	BB	0.4320	649.92627	22.43330	48.8501

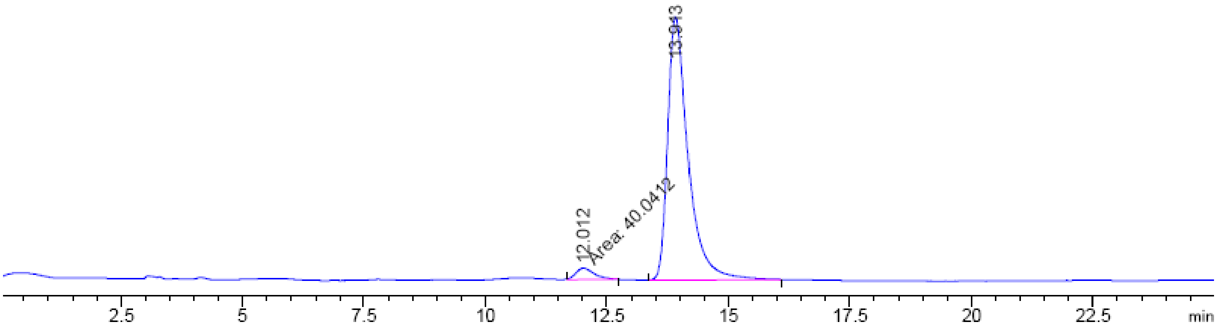

Peak #	RetTime [min]	Type	Width [min]	Area [mAU*s]	Height [mAU]	Area %
1	12.012	MM	0.4290	40.04124	1.55565	3.6434
2	13.913	BB	0.4351	1058.96948	36.21864	96.3566

(5a*R*,6a*R*)-5-(4-methoxybenzyl)-5a-(3-phenylpropyl)-5,5a,6,6a-tetrahydro-4*H*-cyclopropa[b]-thieno[3,4-d]pyridin-4-one (**4p**): obtained as a colourless wax 29% yield (11.1 mg) employing 5.0 mol% Pd(dba)$_2$ and 10 mol% **L2** in mesitylene (0.1 M) at 130 °C. **^{1}H NMR** (400 MHz, CDCl$_3$) δ = 8.08 (d, *J* = 3.1 Hz, 1H), 7.29 – 7.15 (m, 5H), 7.07 – 7.02 (m, 3H), 6.82 (d, *J* = 8.6 Hz, 2H), 5.24 (d, *J* = 14.8 Hz, 1H), 4.30 (d, *J* = 14.8 Hz, 1H), 3.79 (s, 3H), 2.54 – 2.33 (m, 3H), 1.99 (dd, *J* = 9.6, 6.3 Hz, 1H), 1.68 – 1.59 (m, 2H), 1.02 (dd, *J* = 9.6, 5.3 Hz, 1H), 0.80 (ddd, *J* = 14.9, 10.5, 6.9 Hz, 1H), 0.36 (t, *J* = 5.8 Hz, 1H) ppm; **^{13}C{^{1}H} NMR** (101 MHz, CDCl$_3$) δ = 160.8, 158.9, 141.9, 137.5, 131.2, 130.1, 130.0, 129.8, 128.5, 126.1, 120.3, 113.8, 55.4, 45.9, 44.0, 36.0, 35.4, 27.9, 21.7, 18.9 ppm; **IR (ATR):** $\tilde{\nu}$ 2931, 1635, 1556, 1512, 1466, 1454, 1396, 1322, 1301, 1248, 1176, 1033, 816, 772, 752, 700 cm^{-1}; **HRMS (ESI)** calculated for $[C_{25}H_{26}NO_2S]^+$ 404.1679, found 404.1673; **R$_f$:** 0.29 (pentane/EtOAc 3/1); **$[\alpha]_D^{20}$:** -60.0° (*c* = 0.1, CHCl$_3$). Chiral HPLC: (Chiralpak IB, 4.6 x 250 mm; 20% *i*-PrOH / hexane, 1.0 mL/min, 254 nm; tr (minor) = 8.8 min, tr (major) = 9.5 min), 86.0/14.0 *er.*

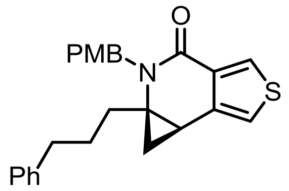

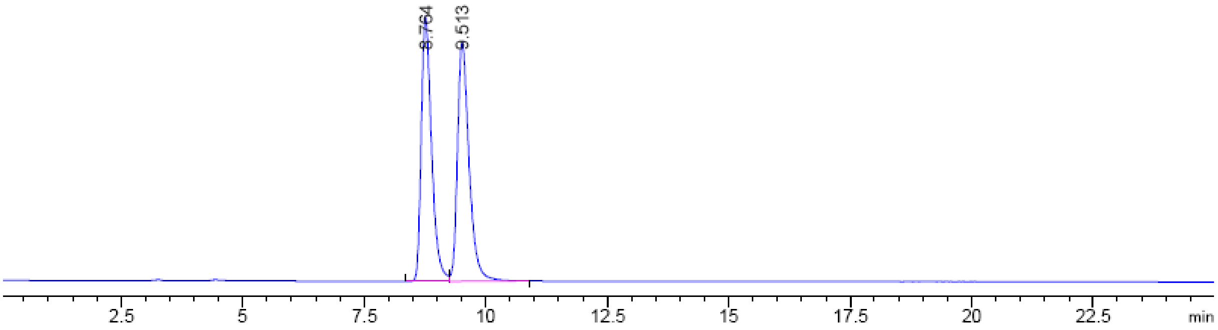

Peak #	RetTime [min]	Type	Width [min]	Area [mAU*s]	Height [mAU]	Area %
1	8.764	BV	0.2248	3581.27686	241.80182	49.4227
2	9.513	VB	0.2534	3664.93555	218.77483	50.5773

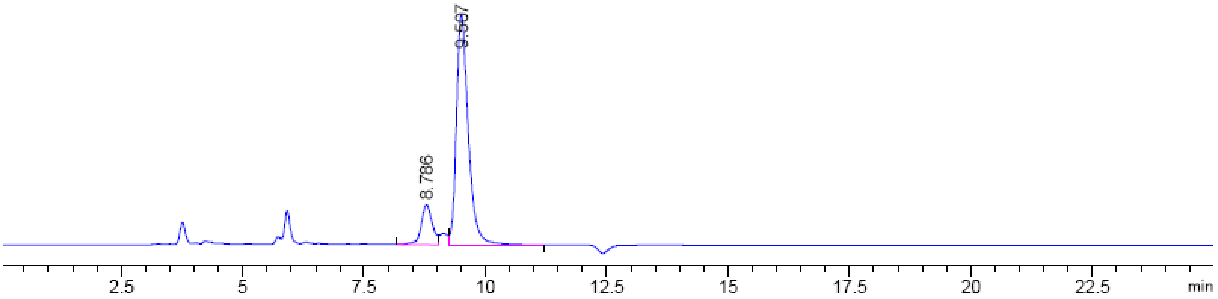

Peak #	RetTime [min]	Type	Width [min]	Area [mAU*s]	Height [mAU]	Area %
1	8.786	BV	0.2392	465.84222	29.34346	13.9592
2	9.507	VB	0.2596	2871.32007	167.83911	86.0408

Cleavage of PBM-protecting group

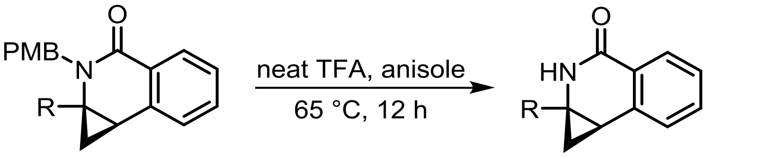

4c (R = H, 92.3/7.7 er))
4i (R = 4-Cl-C_6H_4)

4e (R = H, 93.5/6.5 er)
4q (R = 4-Cl-C_6H_4)

To a mixture of isoquinolinone **4** and anisole (5.0 eq.) was added TFA (43 eq.) and the resulting clear yellow solution stirred at 65 °C for 12 h. After cooling down to rt, TFA was removed under reduced pressure and the residue chromatographed on silica gel to afford the corresponding secondary amide.

<u>(1a*R*,7b*R*)-1a-(4-chlorophenyl)-1a,2-dihydro-1*H*-cyclopropa[c]isoquinolin-3(7b*H*)-one (**4q**):</u>

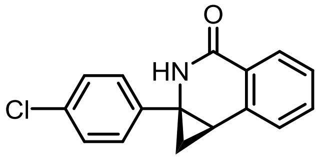

Obtained as a white solid in quantitative yield (29.0 mg, 0.11 mmol scale). **^{1}H NMR** (400 MHz, $CDCl_3$) δ = 8.11 (d, *J* = 7.7 Hz, 1H), 7.42 (td, *J* = 7.6, 1.3 Hz, 1H), 7.29 (dd, *J* = 14.4, 6.5 Hz, 2H), 7.16 (s, 4H), 6.18 (s, 1H), 2.36 – 2.26 (m, 1H), 1.99 (dd, *J* = 10.2, 5.5 Hz, 1H), 0.75 (dd, *J* = 5.9, 5.5 Hz, 1H) ppm; **^{13}C{^{1}H} NMR** (101 MHz, $CDCl_3$) δ = 163.1, 139.6, 138.8, 134.2, 133.0, 129.3, 129.0, 128.5, 128.3, 127.1, 124.3, 42.2, 25.5, 20.6 ppm; **IR (ATR):** $\tilde{\nu}$ 3188, 3074, 2964, 2922, 1660, 1604, 1575, 1478, 1384, 1261, 1096, 1014, 800, 751, 520 cm^{-1}; **HRMS (ESI)** calculated for $[C_{16}H_{13}ClNO]^+$ 270.0680, found 270.0678; **R_f:** 0.75 (EtOAc); **m.p.:** 140.7-142.1 °C; **$[\alpha]_D^{20}$:** +115.0° (*c* = 0.1, $CHCl_3$). This compound was crystallised from *i*-Pr_2O/Et_2O and its absolute configuration assigned by X-ray crystallography as *R*,*R*:

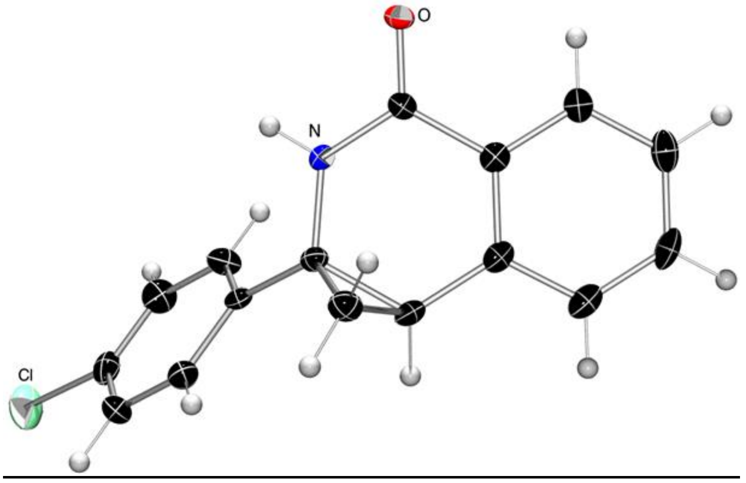

(1aR,7bR)-1a,2-dihydro-1H-cyclopropa[c]isoquinolin-3(7bH)-one (**4e**):

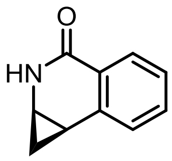

Obtained as a white wax in quantitative yield (0.25 g, 1.6 mmol scale). **^{1}H NMR** (400 MHz, $CDCl_3$) δ = 8.14 (d, J = 7.6 Hz, 1H), 7.44 (ddt, J = 18.7, 14.8, 3.9 Hz, 2H), 7.30 (dt, J = 13.0, 2.9 Hz, 1H), 3.18 (ddd, J = 10.5, 7.1, 3.6 Hz, 1H), 2.26 (ddd, J = 9.5, 8.3, 5.0 Hz, 1H), 1.38 – 1.27 (m, 1H), 0.26 (td, J = 5.4, 4.1 Hz, 1H) ppm; **^{13}C{^{1}H} NMR** (101 MHz, $CDCl_3$) δ = 163.6, 140.5, 132.7, 129.0, 128.4, 126.6, 124.8, 30.0, 14.8, 14.5 ppm; **IR (ATR):** $\tilde{\nu}$ 3197, 3040, 1658, 1604, 1574, 1479, 1409, 1352, 1203, 1182, 1153, 1136, 861, 802, 758, 731 cm^{-1}; **HRMS (ESI)** calculated for $[C_{10}H_{10}NO]^+$ 160.0757, found 160.0759; **R_f:** 0.32 (EtOAc); **$[\alpha]_D^{20}$:** -106.7° (c = 0.1, $CHCl_3$). Chiral HPLC: (Chiralpak AYH, 4.6 x 250 mm; 20% i-PrOH / hexane, 1.0 mL/min, 254 nm; t_r (minor) = 14.9 min, t_r (major) = 10.5 min), 93.5/6.5 *er.*

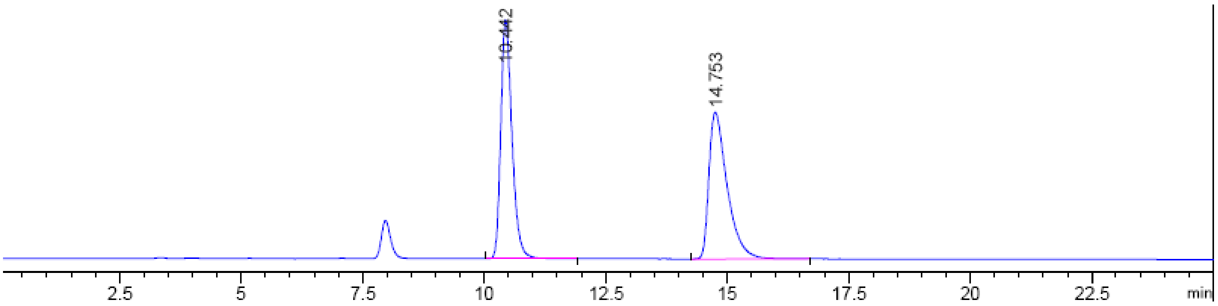

Peak #	RetTime [min]	Type	Width [min]	Area [mAU*s]	Height [mAU]	Area %
1	10.442	BB	0.2463	1188.08472	73.59267	49.8985
2	14.753	BB	0.3954	1192.91711	45.27348	50.1015

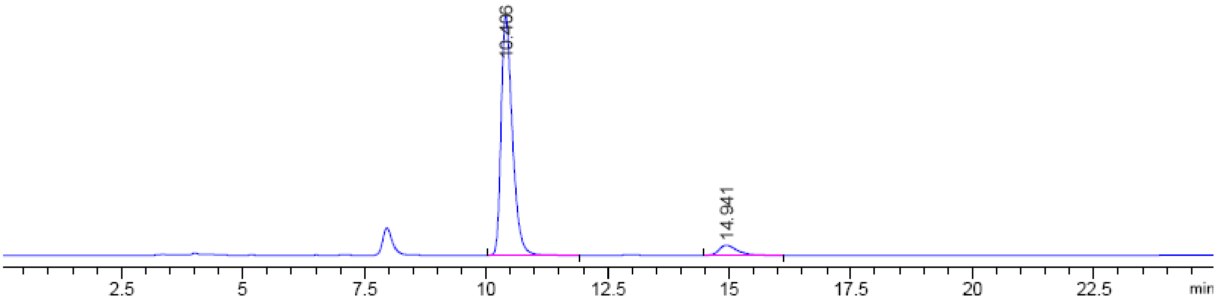

Peak #	RetTime [min]	Type	Width [min]	Area [mAU*s]	Height [mAU]	Area %
1	10.406	BB	0.2466	2435.42017	150.62355	93.5494
2	14.941	BB	0.3909	167.93280	6.38375	6.4506

Enantioselective C-H functionalization strategy towards the BMS-791325 ring system

Synthesis of ethyl 1-acetyl-2-(2-bromo-4-methoxyphenyl)-3-cyclohexyl-1*H*-indole-6-carboxylate (**19**):

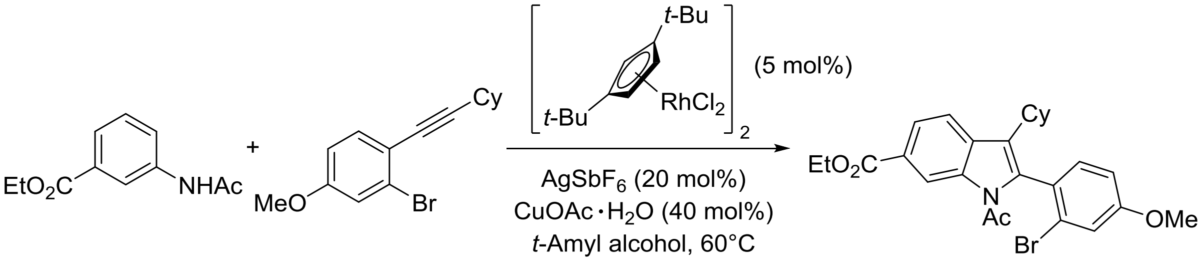

17 **18** **19**

A mixture of ethyl 3-acetamidobenzoate (**17**, 104 mg, 0.5 mmol), 2-bromo-1-(cyclohexylethynyl)-4-methoxybenzene (**18**, 161 mg, 1.1 eq.), $[Cp^tRhCl_2]_2$ (18 mg, 5 mol%), $AgSbF_6$ (34 mg, 20 mol%) and $Cu(OAc)_2{\cdot}H_2O$ (36 mg, 40 mol%) in *t*-amyl alcohol (2.5 mL) was stirred at 60 °C under O_2 atmosphere (1 atm - balloon) for 24 h. Upon completion of the reaction time the volatiles were removed *in vacuo*. The residue was suspended in DCM (5 mL) and filtered through celite. The filtrate was concentrated under reduced pressure and the residue subjected to column chromatography on silica gel to afford pure **19** as yellowish oil in 68% yield (170 mg).

^{1}**H-NMR** (400 MHz, $CDCl_3$): δ = 9.16 (d, *J* = 1.4 Hz, 1H), 7.98 (dd, J_1 = 8.4 Hz, J_2 = 1.5 Hz, 1H), 7.80 (d, *J* = 8.3 Hz, 1H), 7.25-7.28 (m, 2H), 6.99 (dd, J_1 = 8.5 Hz, J_2 = 2.6 Hz, 1H), 4.42 (qd, J_1 = 7.2 Hz, J_2 = 1.3 Hz, 2H), 3.90 (s, 3H), 2.27-2.40 (m, 1H), 2.02 (s, 3H), 1.63-1.88 (m, 7H), 1.42 (t, *J* = 7.1 Hz, 3H), 1.12-1.34 (m, 3H); ^{13}**C{^{1}H}-NMR** (100 MHz, $CDCl_3$): δ = 170.3, 167.3, 160.9, 136.5, 135.3, 133.0, 132.1, 127.4, 126.9, 126.7, 126.5, 124.3, 120.1, 118.8, 118.4, 113.9, 61.0, 55.8, 37.0, 32.0, 31.8, 26.9, 26.7, 26.2, 14.5; **IR (ATR)**: $\tilde{\nu}$ = 2977, 2929, 2852, 1710, 1604, 1582, 1555, 1491, 1475, 1425, 1368, 1306, 1287, 1230, 1160, 1128, 1095, 1031, 950, 912, 843, 777, 750; **HRMS (ESI)** calculated for $[C_{26}H_{29}{}^{79}BrNO_4]^+$ 498.1271, found 498.1274; **R*f***: 0.27 (pentane/EtOAc 5/1).

Synthesis of ethyl 2-(2-bromo-4-methoxyphenyl)-3-cyclohexyl-1*H*-indole-6-carboxylate:

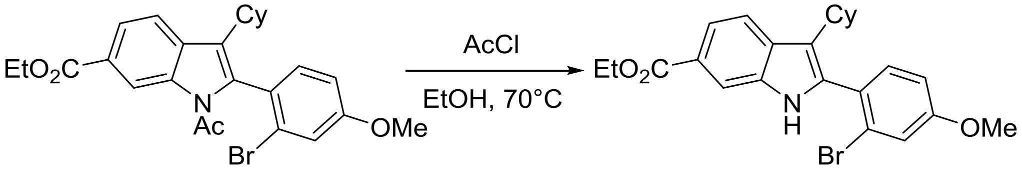

19

Acetyl chloride (0.345 ml, 10 eq.) was added dropwise to a solution of **19** (242 mg, 0.486 mmol) in EtOH (4 mL) at 0 °C. The obtained solution was heated at 70 °C for 4 h, whereupon all volatiles were removed *in vacuo*. The residue was dissolved in DCM (5 mL), stirred with K_2CO_3 (500 mg) for 20 min, filtered through celite and concentrated under reduced pressure. Purification of the residue by column chromatography on silica gel afforded ethyl 2-(2-bromo-4-methoxyphenyl)-3-cyclohexyl-1*H*-indole-6-carboxylate as yellowish oil (201 mg, 91%).

^{1}H-NMR (400 MHz, $CDCl_3$): δ = 8.10 (s, 1H), 7.77-7.84 (m, 2H), 7.29 (d, *J* = 8.5 Hz, 1H), 7.25 (d, *J* = 2.8 Hz, 1H), 6.94 (dd, J_1 = 8.4 Hz, J_2 = 2.6 Hz, 1H), 4.40 (q, *J* = 7.1 Hz, 2H), 3.88 (s, 3H), 2.60 (p, *J* = 7.9 Hz, 1H), 1.67-1.90 (m, 7H), 1.41 (t, *J* = 7.1 Hz, 3H), 1.21-1.35 (m, 3H); **^{13}C{^{1}H}-NMR** (100 MHz, $CDCl_3$): δ = 167.8, 160.5, 135.6, 135.2, 133.2, 130.5, 126.4, 125.0, 123.7, 120.5, 120.14, 120.13, 118.3, 113.4, 113.3, 60.7, 55.8, 36.7, 33.1, 27.2, 26.3, 14.6; **IR (ATR)**: $\tilde{\nu}$ = 3324, 2978, 2926, 2849, 1687, 1605, 1555, 1492, 1460, 1438, 1392, 1368, 1319, 1286, 1212, 1141, 1118, 1095, 1033, 987, 949, 888, 863, 844, 820, 775, 748; **HRMS (ESI)** calculated for $[C_{24}H_{27}{}^{79}BrNO_3]^+$ 456.1169, found 456.1148; **R*f***: 0.34 (pentane/EtOAc 5/1).

Synthesis of ethyl 2-(2-bromo-4-methoxyphenyl)-3-cyclohexyl-1-((1-(ethoxycarbonyl)-cyclopropyl)methyl)-1*H*-indole-6-carboxylate (**16**):

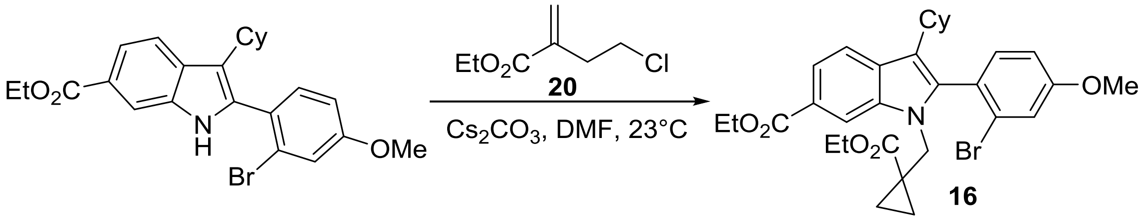

A mixture of ethyl 2-(2-bromo-4-methoxyphenyl)-3-cyclohexyl-1*H*-indole-6-carboxylate (163 mg, 0.358 mmol) and Cs_2CO_3 (233 mg, 2 eq.) in DMF (3.6 mL) was stirred at 23 °C for 30 min followed by addition of 4-chloro-2-methylenebutanoate (**20**, 87 mg, 1.5 eq.). The mixture was stirred at 23 °C for 17 h, whereupon all volatiles were removed *in vacuo*. The residue was suspended in EtOAc (5 mL), filtered through celite and concentrated under reduced pressure. Purification of the residue by column chromatography on silica gel afforded pure **16** (201 mg, 96%) as colourless oil.

^{1}H-NMR (500 MHz, $CDCl_3$): δ = 8.16 (s, 1H), 7.78 (s, 2H), 7.26 (d, J = 2.6 Hz, 1H), 7.19 (d, J = 8.5 Hz, 1H), 6.96 (dd, J_1 = 8.5 Hz, J_2 = 2.6 Hz, 1H), 4.64 (d, J = 15.6 Hz, 1H), 4.36-4.46 (m, 2H), 4.27 (d, J = 15.6 Hz, 1H), 4.01 (q, J = 7.1 Hz, 2H), 3.89 (s, 3H), 2.35-2.47 (m, 1H), 1.66-1.86 (m, 7H), 1.42 (t, J = 7.1 Hz, 3H), 1.17-1.28 (m, 3H), 1.10 (t, J = 7.1 Hz, 3H), 1.03-1.06 (m, 2H), 0.30-0.43 (m, 2H); **^{13}C{^{1}H}-NMR** (125 MHz, $CDCl_3$): δ = 173.6, 167.9, 160.6, 138.3, 136.8, 133.9, 129.8, 126.6, 125.5, 123.4, 120.7, 120.0, 119.8, 118.1, 113.5, 112.7, 61.0, 60.7, 55.7, 44.4, 37.0, 33.2, 33.1, 27.1, 26.3, 24.0, 15.1, 15.0, 14.6, 14.1; **IR (ATR)**: $\tilde{\nu}$ = 2978, 2926, 2851, 1707, 1605, 1556, 1490, 1459, 1376, 1342, 1286, 1262, 1234, 1217, 1194, 1173, 1147, 1104, 1031, 982, 910, 889, 862, 843, 827, 774, 748, 734; **HRMS (ESI)** calculated for $[C_{31}H_{37}{}^{79}BrNO_5]^+$ 582.1815, found 582.1827; **R$_f$**: 0.27 (pentane/EtOAc 5/1).

Synthesis of (4b*R*,5a*S*)-diethyl 12-cyclohexyl-3-methoxy-4b,5,5a,6-tetrahydrobenzo[3,4]-cyclopropa[5,6]azepino[1,2-*a*]indole-5a,9-dicarboxylate (***ent*-15**):

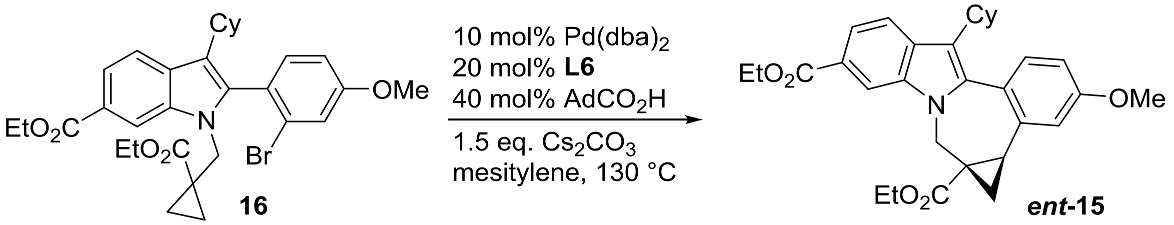

$Pd(dba)_2$ (7.24 mg, 13 μmol, 10 mol%), **L6** (17.2 mg, 25 μmol, 20 mol%), $AdCO_2H$ (9.1 mg, 50 μmol, 40 mol%) and Cs_2CO_3 (61.5 mg, 0.19 mmol, 1.5 eq.) were weighed in a microwave tube containing a magnetic stirring bar. The tube was closed with a cap, evacuated and backfilled with N_2. Ethyl 2-(2-bromo-4-methoxyphenyl)-3-cyclohexyl-1-((1-(ethoxycarbonyl)-cyclopropyl)methyl)-1*H*-indole-6-carboxylate (**16**, 73.3 mg, 0.126 mmol, 1.0 eq.) was added as a solution in mesitylene (2.5 ml, 0.05 M) and the reaction mixture degassed by three freeze-pump-thaw cycles. The tube was sealed and placed in a pre-heated oil bath. The reaction mixture was stirred at 130 C for 9 h. After cooling down to rt, the mixture was chromatographed on silica gel to afford pure ***ent*-15** as a yellow foam in 80% yield (50.5 mg) and 94.5/5.5 *er.*

^{1}H-NMR (500 MHz, $CDCl_3$) mixture of atropisomers in ratio 1.5/1 δ = 8.32 (d, J = 0.9 Hz, 0.6H), 8.14 (s, 0.4H), 7.87 – 7.69 (m, 2H), 7.27 (dd, J = 11.5, 8.5 Hz, 2H), 7.14 (d, J = 2.2 Hz, 0.6H), 7.02 (d, J = 2.6 Hz, 0.4H), 6.97 – 6.88 (m, 1H), 5.45 (d, J = 15.0 Hz, 0.6H), 5.20 (d, J = 15.1 Hz, 0.4H), 4.50 – 4.35 (m, 2H), 4.33 – 4.19 (m, 0.8H), 4.12 – 4.02 (m, 1.2H), 3.93 (dt, J = 7.2, 5.4 Hz, 0.6H), 3.89 (s, 3H), 3.44 (d, J = 15.0 Hz, 0.6H), 2.98 – 2.87 (m, 1.2H), 2.79 (tt, J = 12.0, 3.5 Hz, 0.4H), 2.64 (dd, J = 10.0, 6.9 Hz, 0.4H), 2.14 – 1.89 (m, 4H), 1.84 – 1.71 (m, 2.8H), 1.68 (dd, J = 9.5, 4.0 Hz, 0.6H), 1.46 – 1.32 (m, 6H), 1.21 (dd, J = 6.1, 4.1

Hz, 0.6H), 1.13 (t, J = 7.2 Hz, 2H), 0.44 – 0.38 (m, 0.4H); **^{13}C{^{1}H}-NMR** (101 MHz, $CDCl_3$): δ = 173.0, 172.3, 168.0, 167.9, 159.9, 159.7, 139.0, 137.3, 136.8, 136.5, 135.4, 135.3, 133.1, 133.0, 130.3, 130.1, 123.2, 123.1, 122.5, 120.6, 120.2, 119.9, 119.7, 119.6, 119.2, 118.7, 117.6, 117.5, 113.4, 113.0, 112.3, 111.5, 61.7, 61.6, 60.9, 60.7, 55.5, 44.5, 39.8, 37.0, 36.6, 35.3, 33.4, 33.3, 33.2, 33.1, 32.8, 32.3, 27.3, 27.3, 27.2, 26.5, 26.4, 22.2, 15.0, 14.7, 14.4, 14.1; **IR (ATR)**: $\tilde{\nu}$ = 2927, 2851, 1706, 1610, 1463, 1403, 1379, 1304, 1272, 1234, 1214, 1196, 1168, 1148, 1095, 1027, 774, 746; **HRMS (ESI)** calculated for $[C_{31}H_{36}NO_5]^+$ 502.2588, found 502.2576; **R$_f$**: 0.50 (pentane/EtOAc 5/1); **$[\alpha]_D^{20}$:** +230.0° (c = 0.1, $CHCl_3$). Chiral HPLC: (Chiralpak IC, 4.6 x 250 mm; 10% i-PrOH / hexane, 1.0 mL/min, 320 nm; tr (minor) = 17.1 min, tr (major) = 14.4 min), 94.5/5.5 *er*.

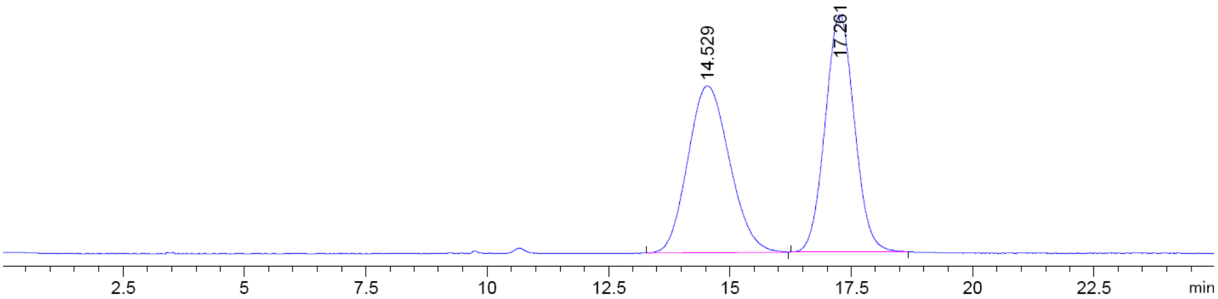

Peak #	RetTime [min]	Type	Width [min]	Area [mAU*s]	Height [mAU]	Area %
1	14.529	BB	0.7339	1478.34644	24.78570	50.0788
2	17.261	BB	0.6207	1473.69165	35.18001	49.9212

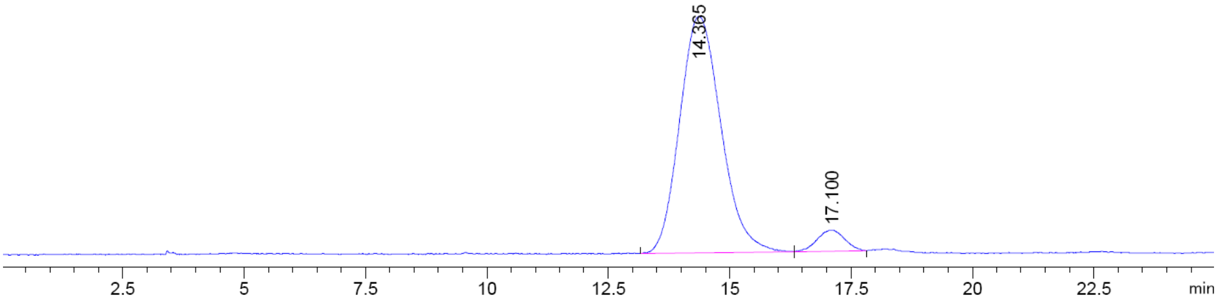

Peak #	RetTime [min]	Type	Width [min]	Area [mAU*s]	Height [mAU]	Area %
1	14.365	BB	0.8017	937.16504	15.85383	94.4350
2	17.100	BB	0.4691	55.22676	1.40954	5.5650

Synthesis of (4b*R*,5a*S*)-12-cyclohexyl-9-(ethoxycarbonyl)-3-methoxy-4b,5,5a,6-tetrahydro-benzo[3,4]cyclopropa[5,6]azepino[1,2-*a*]indole-5a-carboxylic acid (**21**):

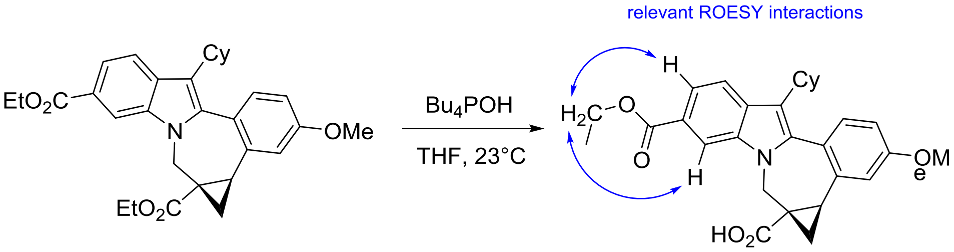

***ent*-15** **21**

Aqueous (40wt%) Bu_4POH (62 µL, 1.3 eq.) was added to a solution of ***ent*-15** (34 mg, 0.068 mmol) in THF (2.5 mL). After stirring for 1 h at 23 °C, 1M HCl (1 mL) was added and the mixture was extracted with EtOAc. Combined organic phases were dried (Na_2SO_4) and concentrated. Purification of the residue by column chromatography on silica gel (gradient elution from DCM to DCM/EtOAc 1/1) afforded **21** (26 mg, 81%) as yellowish film.

^{1}H-NMR (600 MHz, $CDCl_3$): mixture of atropisomers in ratio 1.4/1 - δ = 8.37 (d, J = 1.2 Hz, 0.42H), 8.13 (d, J = 1.2 Hz, 0.58H), 7.85 (d, J = 8.5 Hz, 0.58H), 7.82 (d, J = 8.5 Hz, 0.42H), 7.78 (dd, J_1 = 1.4 Hz, J_2 = 8.5 Hz, 0.58H), 7.73 (dd, J_1 = 1.4 Hz, J_2 = 8.5 Hz, 0.42H), 7.26-7.30 (m, 1H), 7.14 (dd, J_1 = 0.8 Hz, J_2 = 2.8 Hz, 0.42H), 7.02 (d, J = 2.8 Hz, 0.58H), 6.90-6.96 (m, 1H), 5.45 (d, J = 15.0 Hz, 0.42H), 5.20 (d, J = 15.0 Hz, 0.58H), 4.35-4.49 (m, 2H), 4.07 (d, J = 15.0 Hz, 0.58H), 3.89 (s, 3H), 3.43 (d, J = 15.0 Hz, 0.42H), 2.98 (dd, J_1 = 6.2 Hz, J_2 = 9.2 Hz, 0.42H), 2.93 (tt, J_1 = 3.6 Hz, J_2 = 12.2 Hz, 0.42H), 2.79 (tt, J_1 = 3.6 Hz, J_2 = 12.2 Hz, 0.58H), 2.74 (dd, J_1 = 6.9 Hz, J_2 = 10.1 Hz, 0.58H), 1.88-2.13 (m, 4H), 1.68-1.83 (m, 3H), 1.53-1.61 (m, 0.58H), 1.18-1.50 (m, 8H), 0.46-0.51 (m, 0.58H); **^{13}C{^{1}H}-NMR** (150 MHz, $CDCl_3$): δ = 178.5, 178.1, 168.2, 167.9, 159.9, 159.6, 138.5, 137.0, 136.6, 135.8, 135.5, 135.2, 133.1, 133.0, 130.2, 129.9, 123.3, 123.0, 122.8, 120.6, 120.1, 120.0, 119.7, 119.2, 118.8, 117.5, 117.4, 113.5, 113.1, 112.7, 111.5, 60.9, 60.6, 55.54, 55.53, 44.3, 39.4, 37.0, 36.6, 34.9, 33.5, 33.4, 33.3, 33.19, 33.14, 32.0, 27.38, 27.30, 27.27, 27.23, 27.1, 26.4, 26.3, 22.3, 15.5, 14.66, 14.61; **IR (ATR)**: $\tilde{\nu}$ = 2974, 2926, 2850, 1690, 1609, 1561, 1491, 1461, 1423, 1382, 1368, 1344, 1321, 1273, 1237, 1216, 1164, 1104, 1073, 1041, 980, 908, 878, 827, 773, 731; **HRMS (ESI)** calculated. for $[C_{29}H_{32}NO_5]^+$ 474.2275, found 474.2280; **R$_f$**: 0.28 (DCM/EtOAc 1/1); **$[\alpha]_D^{20}$** = +191.5° (c = 1.0, $CHCl_3$).

Determination of the absolute configuration

Synthesis of (4b*R*,5a*S*)-12-cyclohexyl-9-((N,N-dimethylsulfamoyl)carbamoyl)-3-methoxy-4b,5,5a,6-tetrahydrobenzo[3,4]cyclopropa[5,6]azepino[1,2-*a*]indole-5a-carboxylic acid:

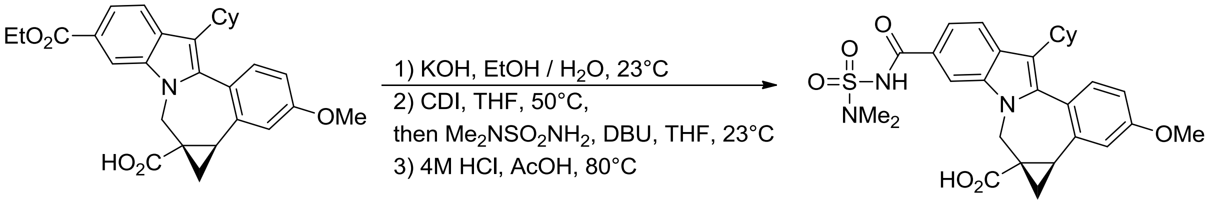

1) KOH (29.6 mg, 20 eq.) was added to a solution of **21** (13 mg, 0.027 mmol) in a mixture of EtOH (0.5 mL) and H_2O (0.5 mL) at 23 °C. After stirring for 2 h at 23 °C the mixture was evaporated under reduced pressure and the residue was partitioned between 4M HCl and EtOAc. The aqueous layer was extracted with EtOAc, combined organic phases dried (Na_2SO_4) and concentrated under reduced pressure affording the crude diacid (10 mg, 0.022 mmol, 85%), which was used in the next step without purification.

2) A mixture of the above diacid (10 mg, 0.022 mmol) and 1,1′-carbonyldiimidazole (10.9 mg, 3 eq.) in THF (0.1 mL) was stirred at 50 °C for 30 min. Upon cooling to 23 °C a solution of *N*,*N*-dimethylsulfamide (8.9 mg, 3.2 eq.) and DBU (13.7 mg, 4 eq.) in THF (0.2 mL) was added at once and the mixture was stirred at 23 °C for 16 h. Then the reaction mixture was partitioned between 4M HCl and EtOAc. The aqueous layer was extracted with EtOAc, combined organic phases dried (Na_2SO_4) and concentrated under reduced pressure affording the crude diamide, which was used in the next step without purification.

3) A solution of the above diamide in a mixture of 4M HCl (0.5 mL) and AcOH (0.5 mL) was stirred at 80 °C for 3 h. Upon cooling to 23 °C the reaction mixture was concentrated under reduced pressure and extracted with EtOAc. Combined organic phases were dried (Na_2SO_4) and concentrated. The title compound (3 mg, 25%) was isolated from the crude product mixture by preparative HPLC (Agilent 1260 Infinity Series) on reversed-phase C18 column (Phenomenex Kinetex EVO, 150 mm X 21.2 mm, 100 Å, 5 µm). Elution was performed using a linear gradient of 50 % B to 98 % B over 25 min at a flow rate of 25 mL/min with UV detection at 254 nm (Solvent A = H_2O + 0.1 % FA; Solvent B = 95 : 5 MeCN : H_2O + 0.1 % FA). Retention time 10.1 min.

Spectroscopic data for this compound was in complete agreement with the reported values for the opposite enantiomer: R. G. Gentles *et al. J. Med. Chem.* 2014, **57**, 1855:

^{1}H-NMR (600 MHz, CD_3OD): mixture of atropisomers in ratio 1.6/1 - δ = 8.28 (d, *J* = 1.6 Hz, 0.62H), 8.09 (d, *J* = 1.6 Hz, 0.38H), 7.89 (d, *J* = 8.5 Hz, 0.38H), 7.85 (d, *J* = 8.4 Hz, 0.62H), 7.61 (dd, J_1 = 8.5 Hz, J_2 = 1.6 Hz, 0.38H), 7.52 (dd, J_1 = 8.5 Hz, J_2 = 1.6 Hz, 0.62H), 7.31 (d, *J* = 8.5 Hz, 0.38H), 7.27 (d, *J* = 8.6 Hz, 0.62H), 7.21 (d, *J* = 2.6 Hz, 0.62H), 7.14 (d, *J* = 2.6 Hz, 0.38H), 6.97-7.01 (m, 1H), 5.46 (d, *J* = 15.0 Hz, 0.62H), 5.27 (d, *J* = 15.2 Hz, 0.38H), 4.03 (d, *J* = 15.2 Hz, 0.38H), 3.44 (d, *J* = 15.0 Hz, 0.62H), 3.02 (s, 3.72H), 3.01 (s, 2.28H), 2.96 (tt, J_1 = 12.3 Hz, J_2 = 3.6 Hz, 0.62H), 2.80-2.90 (m, 1H), 2.74 (dd, J_1 = 10.0 Hz, J_2 = 6.7 Hz, 0.38H), 1.98-2.18 (m, 2.76H), 1.91-1.97 (m, 1H), 1.74-1.85 (m, 2H), 1.63-1.71 (m, 1.24H), 1.21-1.55 (m, 5H), 0.23–0.27 (m, 0.38H); **HRMS (ESI)** calculated. for $[C_{29}H_{34}N_3O_6S]^+$ 552.2163, found 552.2172; $[\alpha]_D^{20}$ = +167.1° (*c* = 0.33, MeOH) – reported value for the opposite enantiomer: $[\alpha]_D^{25}$ = -166.99° (*c* = 1.0, MeOH).

^{1}H and ^{13}C-NMR spectra

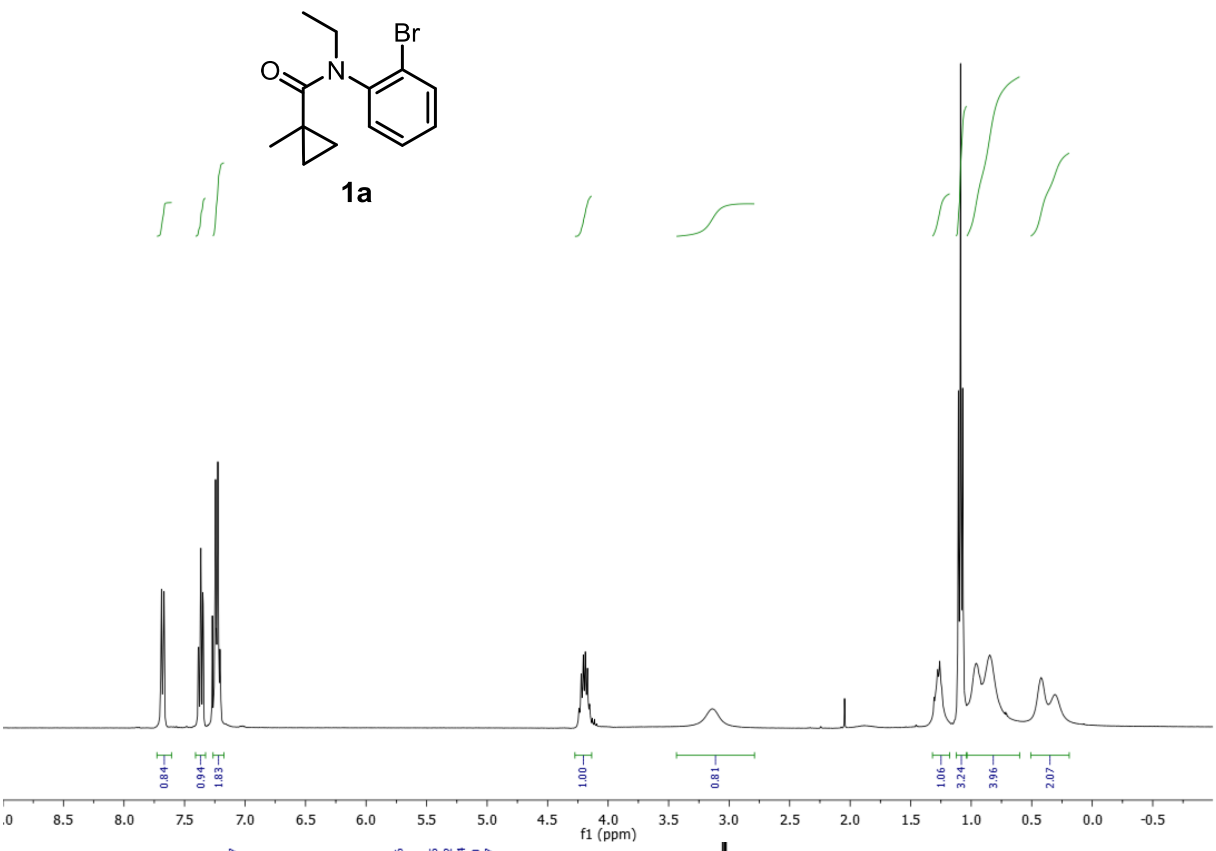

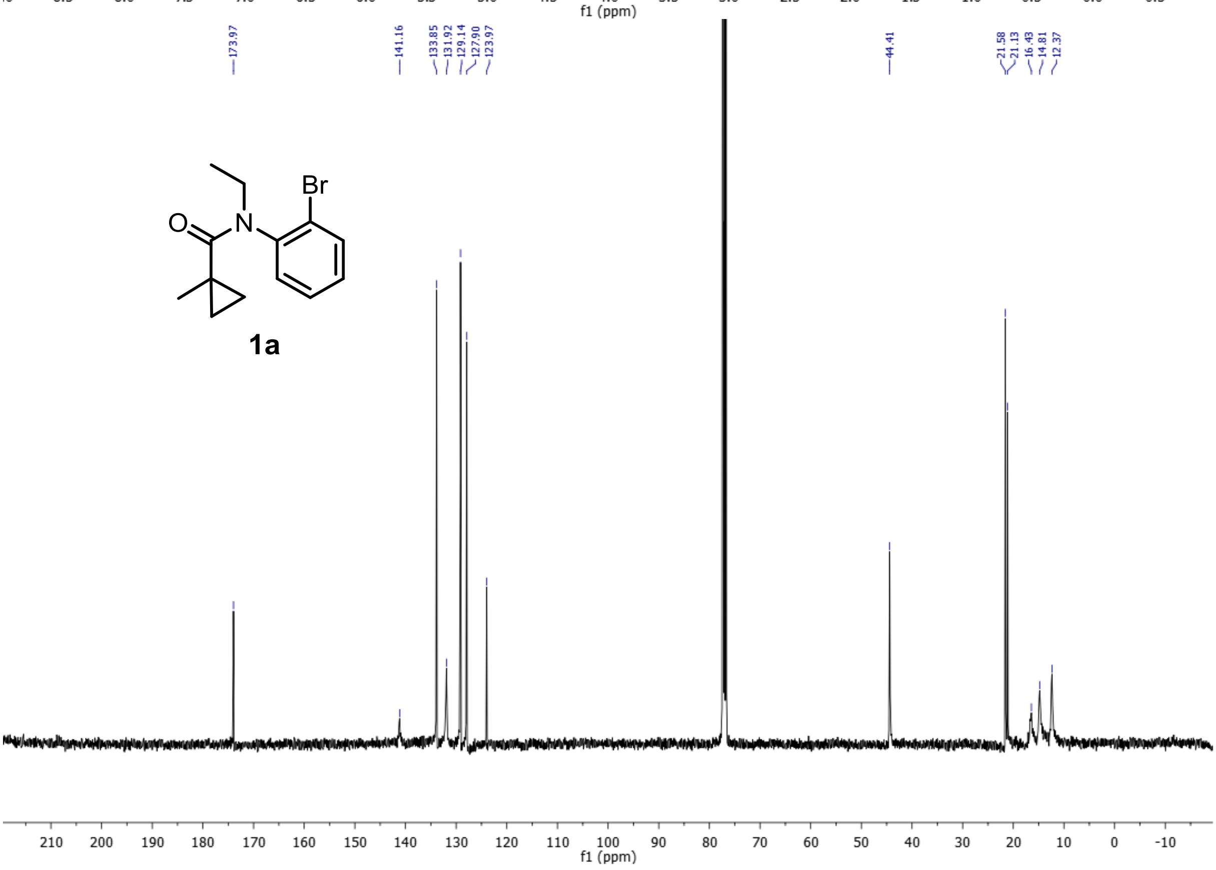

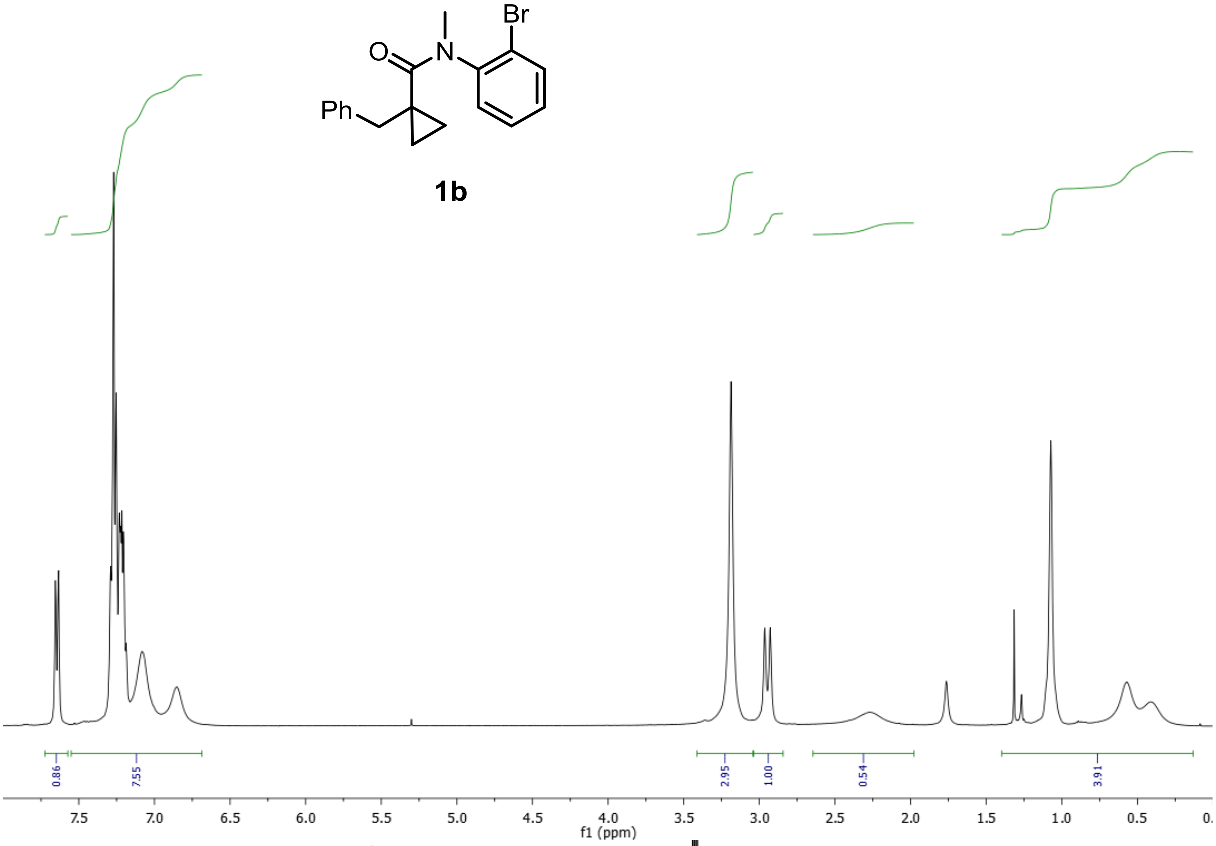
Br
O
N
Ph
1b
0.86
7.55
2.95
1.00
0.54
3.91
7.5 7.0 6.5 6.0 5.5 5.0 4.5 4.0 3.5 3.0 2.5 2.0 1.5 1.0 0.5
f1 (ppm)

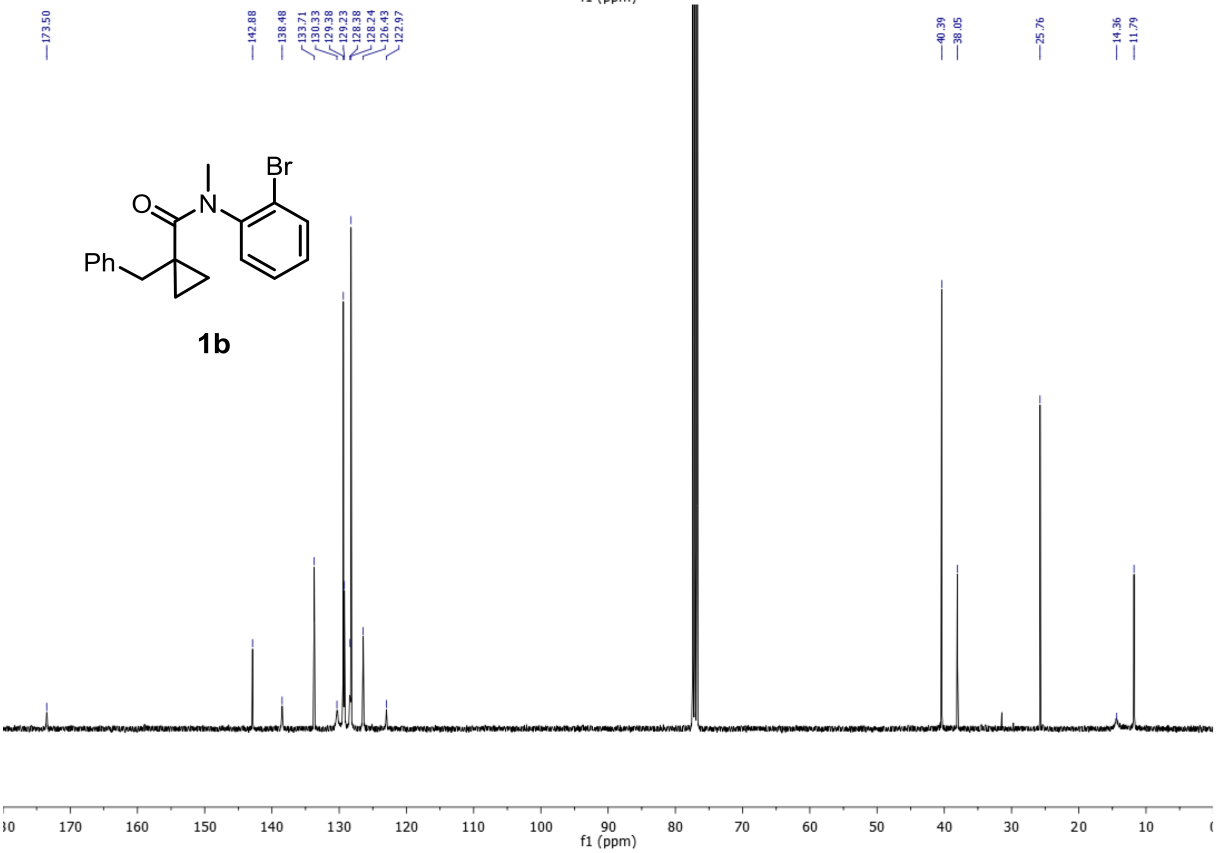
173.50
142.88
138.48
133.71
130.33
129.38
129.23
128.38
128.24
126.43
122.97
40.39
38.05
25.76
14.36
11.79
Br
O
N
Ph
1b
170 160 150 140 130 120 110 100 90 80 70 60 50 40 30 20 10
f1 (ppm)

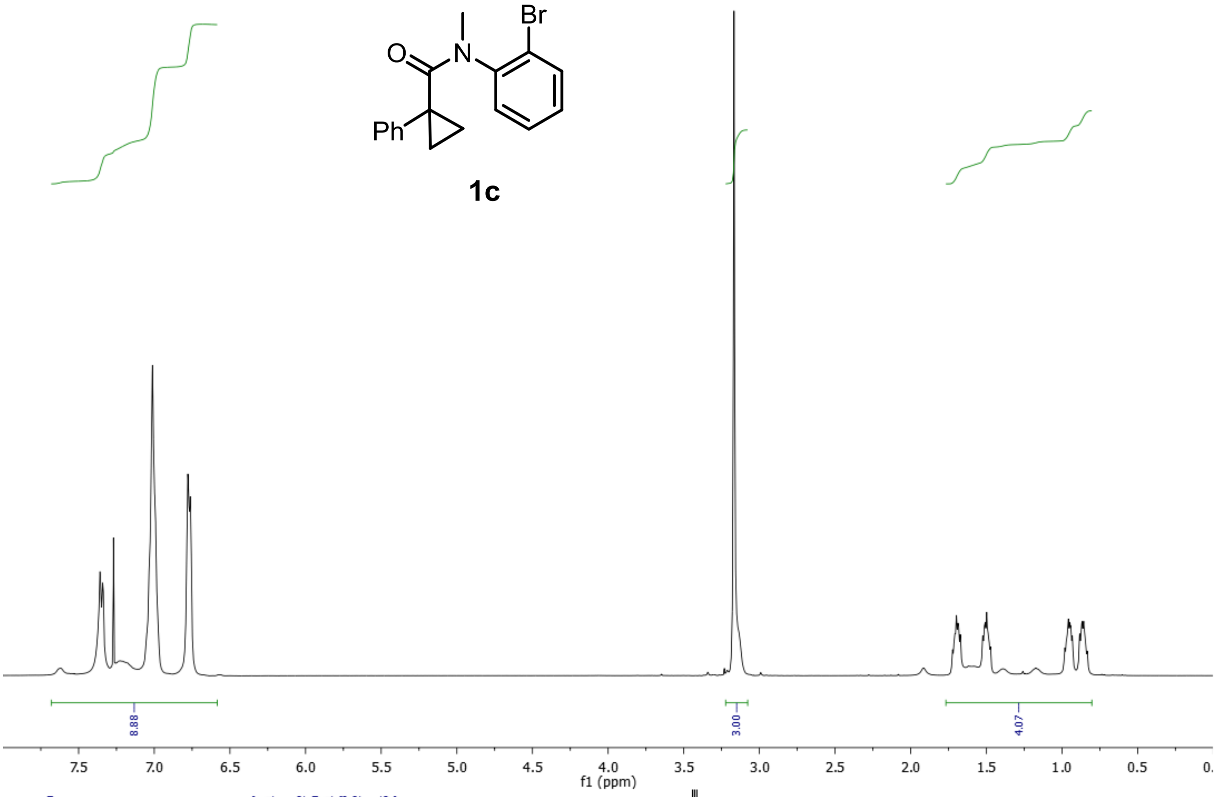
Br
O
N
Ph
1c
8.88
3.00
4.07
7.5 7.0 6.5 6.0 5.5 5.0 4.5 4.0 3.5 3.0 2.5 2.0 1.5 1.0 0.5
f1 (ppm)

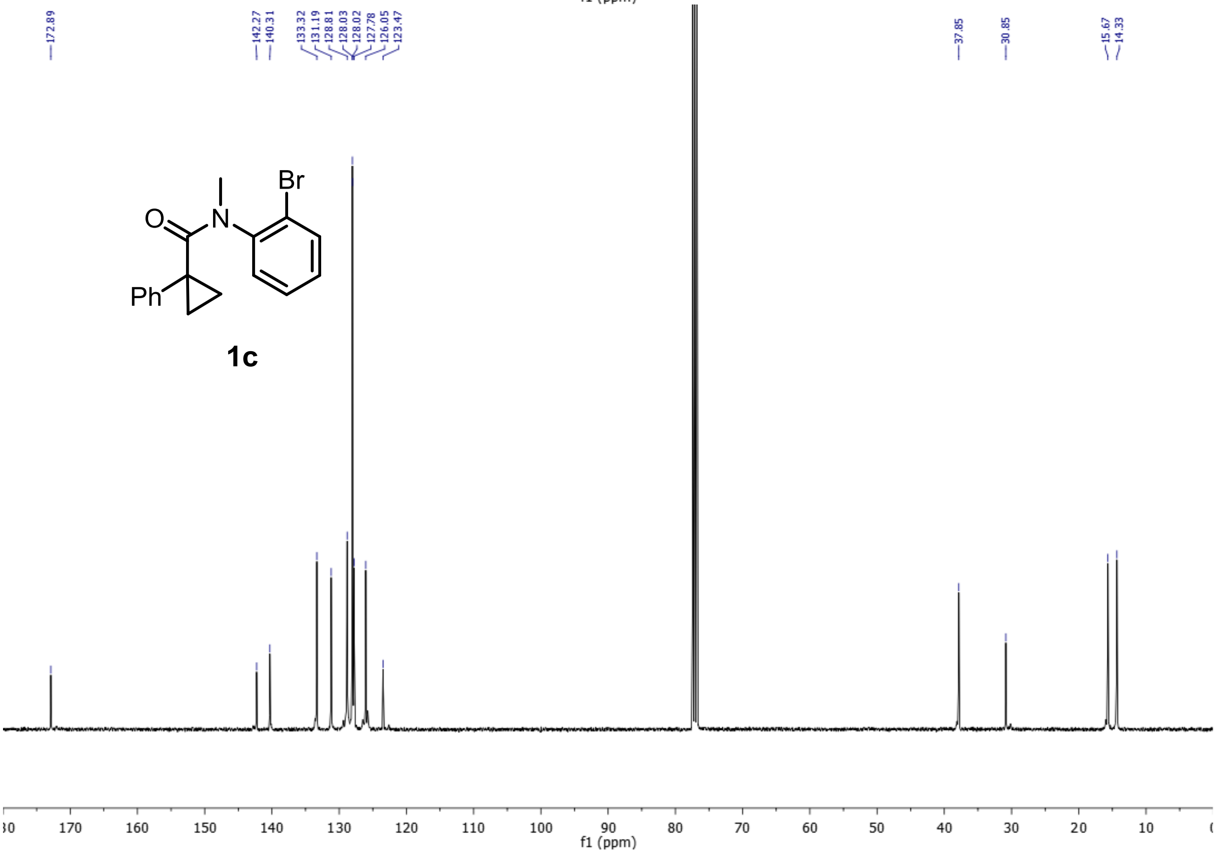
172.89
142.27
140.31
133.32
131.19
128.81
128.03
128.02
127.78
126.05
123.47
37.85
30.85
15.67
14.33
Br
O
N
Ph
1c
170 160 150 140 130 120 110 100 90 80 70 60 50 40 30 20 10
f1 (ppm)

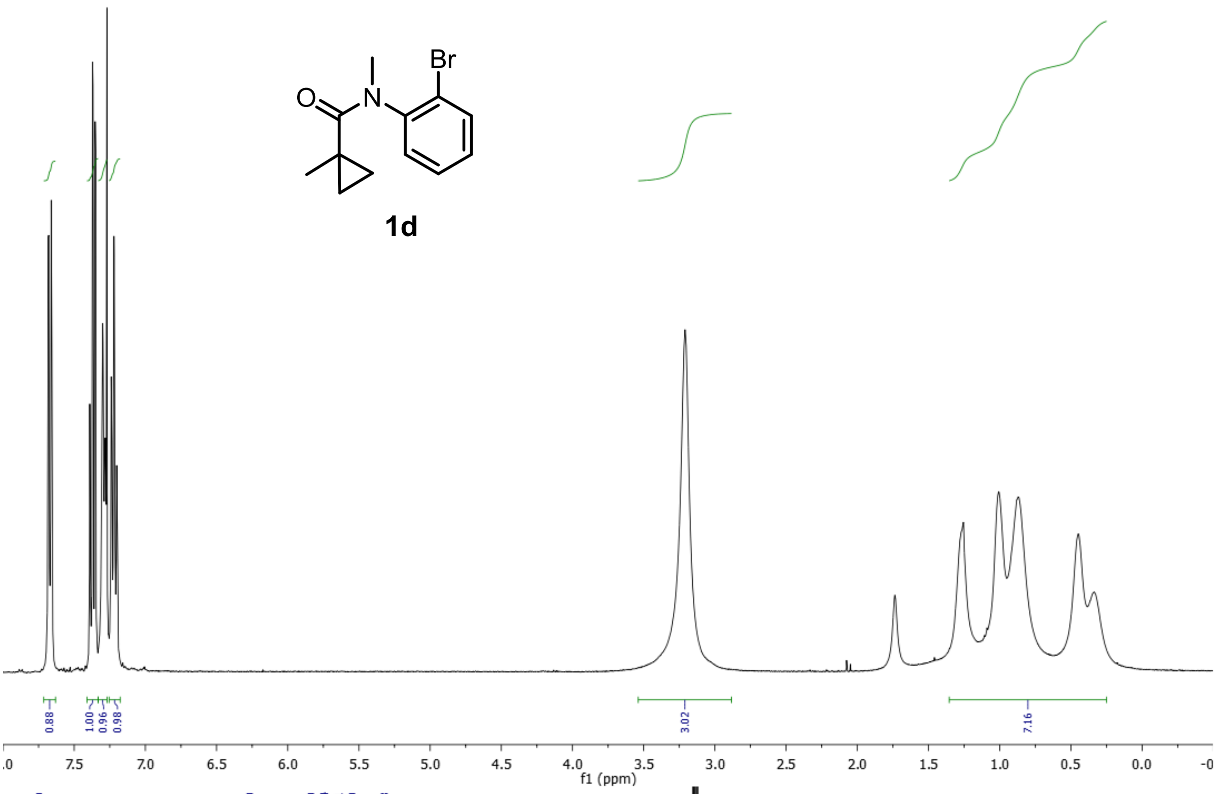
Br
O
N
1d
0.88
1.00
0.96
0.98
3.02
7.16
f1 (ppm)

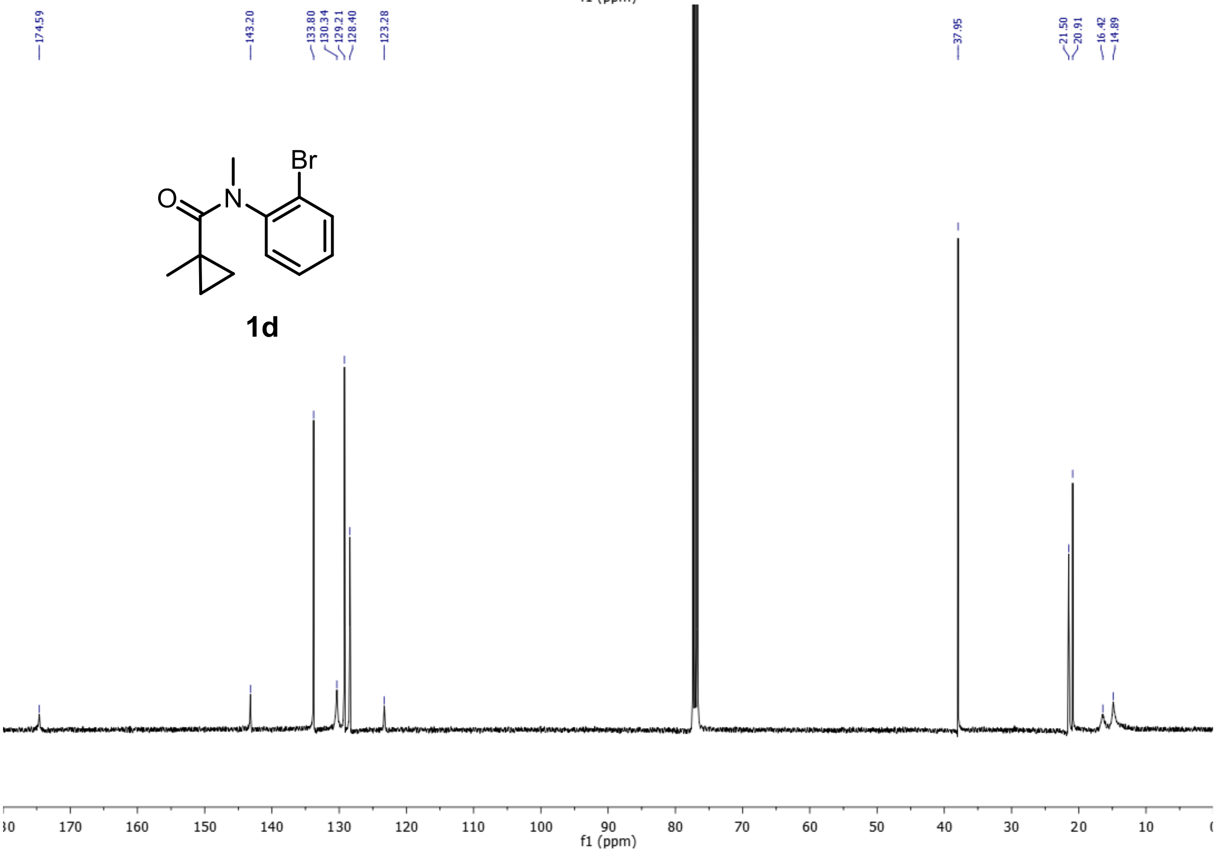
174.59
143.20
133.80
130.34
129.21
128.40
123.28
37.95
21.50
20.91
16.42
14.89
Br
O
N
1d
f1 (ppm)

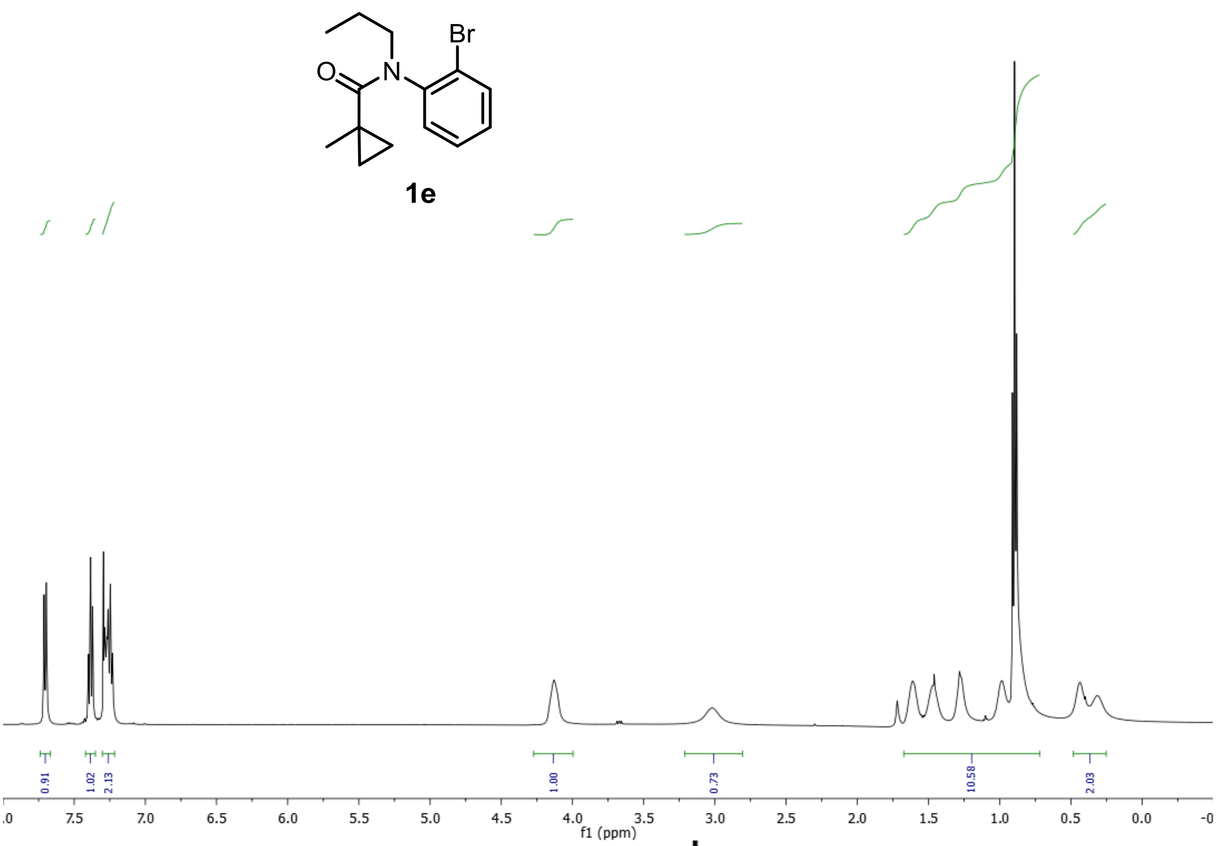

Br
O
N
1e
0.91
1.02
2.13
1.00
0.73
10.58
2.03
f1 (ppm)

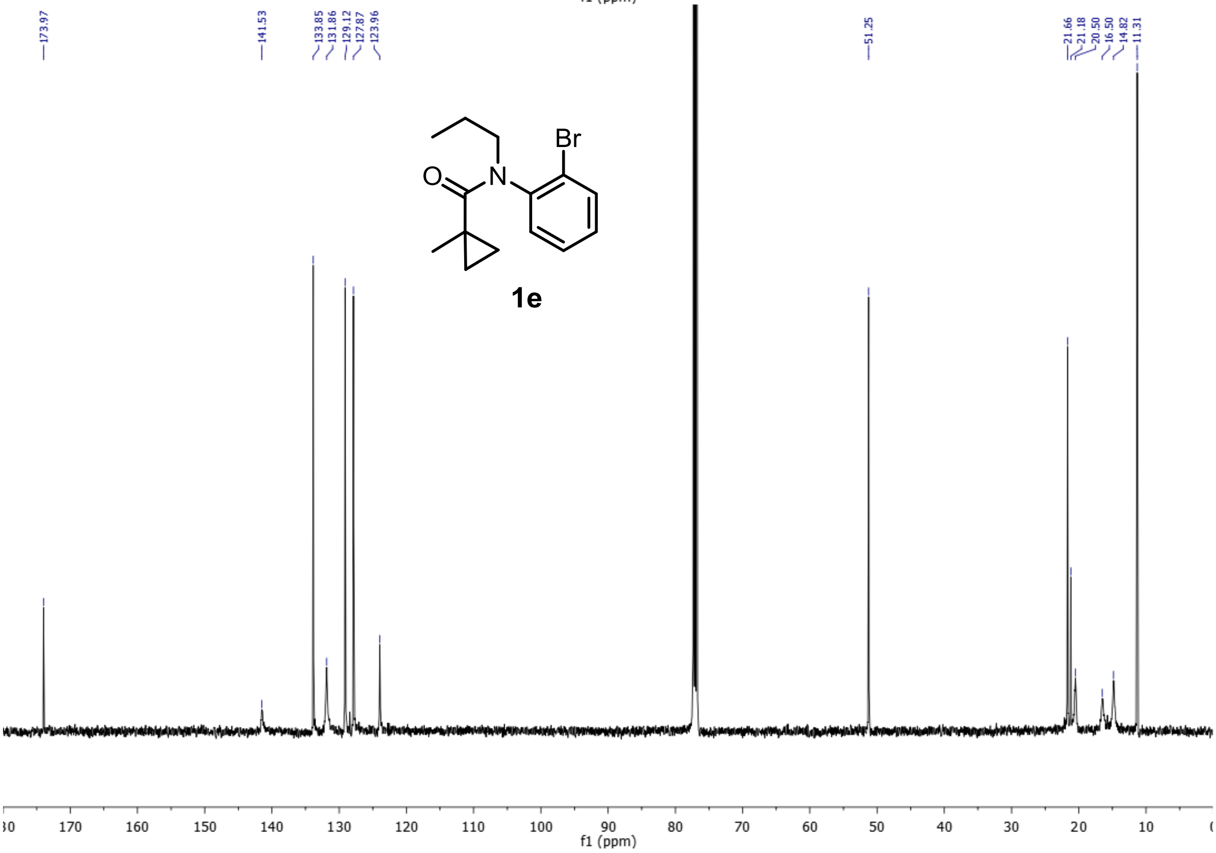

173.97
141.53
133.85
131.86
129.12
127.87
123.96
51.25
21.66
21.18
20.50
16.50
14.82
11.31
Br
O
N
1e
f1 (ppm)

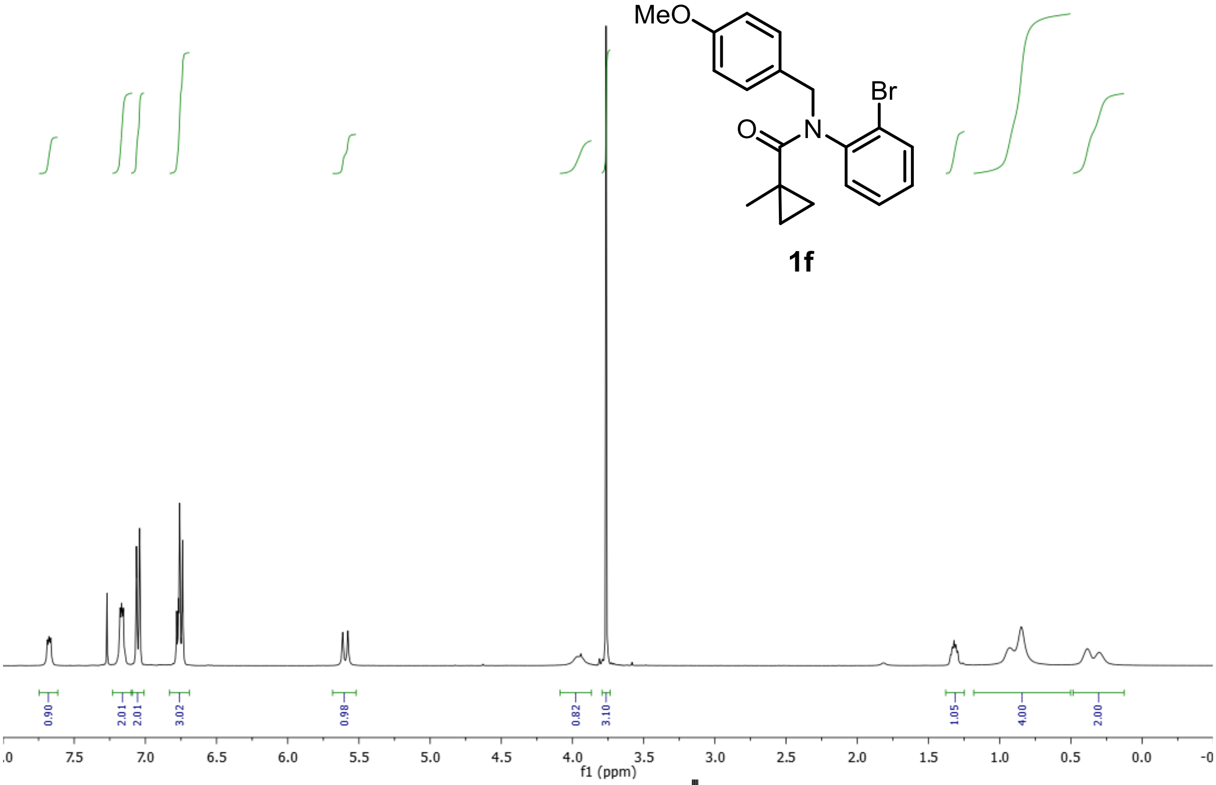
MeO
N
O
Br
1f
0.90
2.01
2.01
3.02
0.98
0.82
3.10
1.05
4.00
2.00
f1 (ppm)

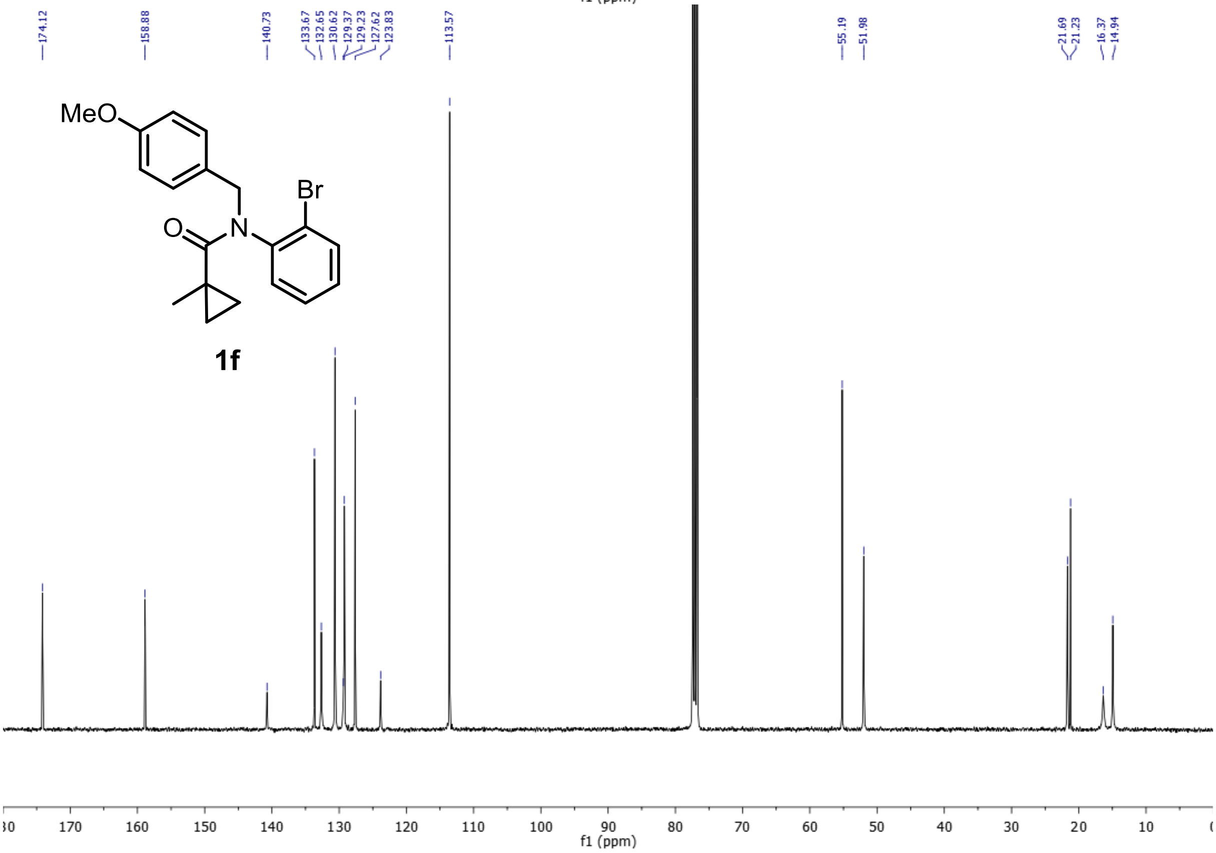
174.12
158.88
140.73
133.67
132.65
130.62
129.37
129.23
127.62
123.83
113.57
55.19
51.98
21.69
21.23
16.37
14.94
MeO
N
O
Br
1f
f1 (ppm)

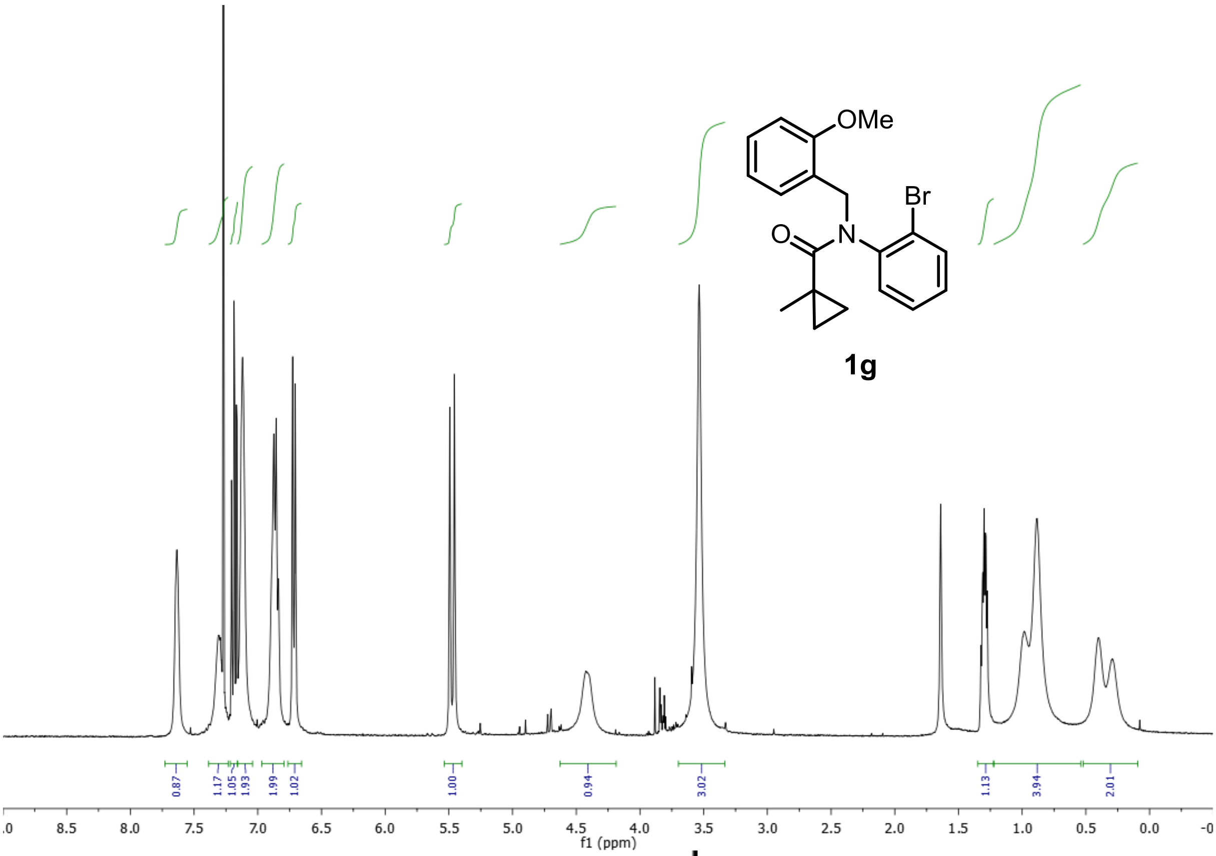
OMe
Br
O
N
1g
0.87
1.17
1.05
1.93
1.99
1.02
1.00
0.94
3.02
1.13
3.94
2.01
f1 (ppm)

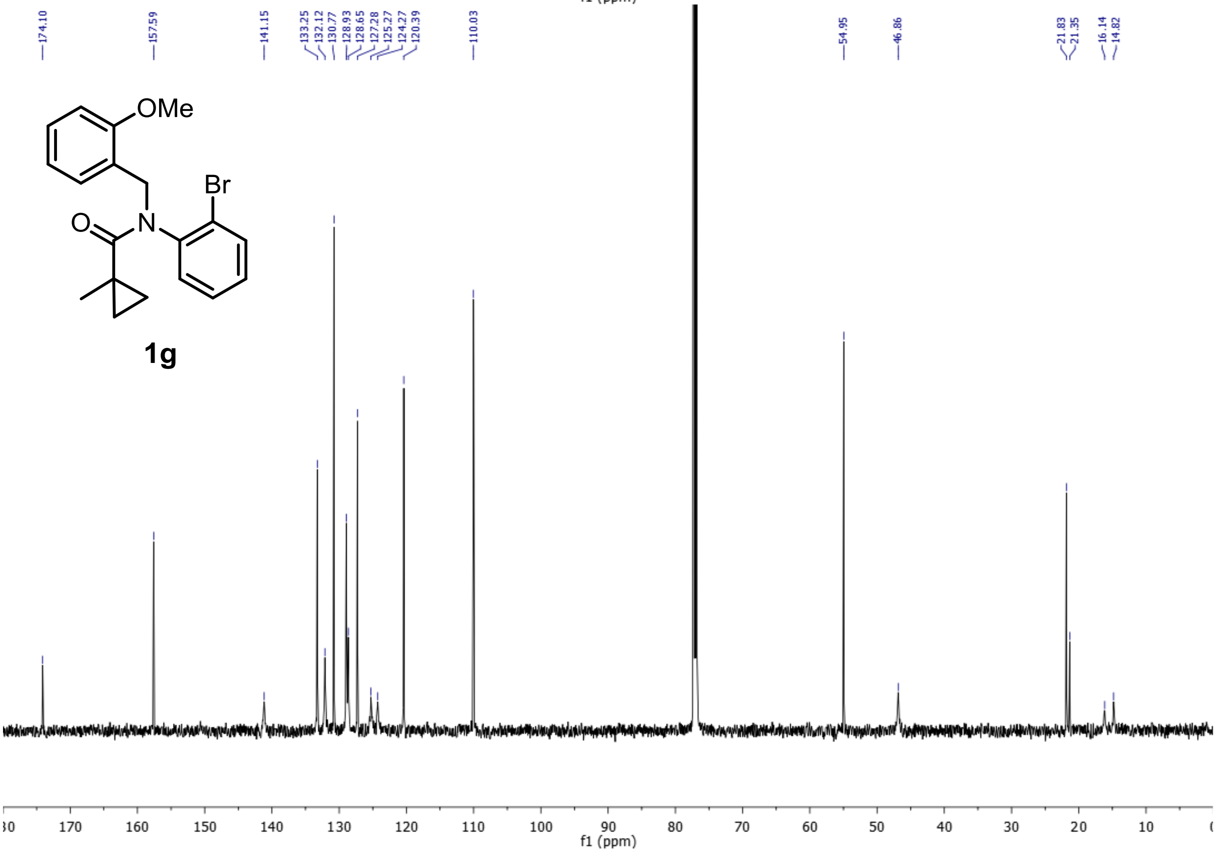
174.10
157.59
141.15
133.25
132.12
130.77
128.93
128.65
127.28
125.27
124.27
120.39
110.03
54.95
46.86
21.83
21.35
16.14
14.82
OMe
Br
O
N
1g
f1 (ppm)

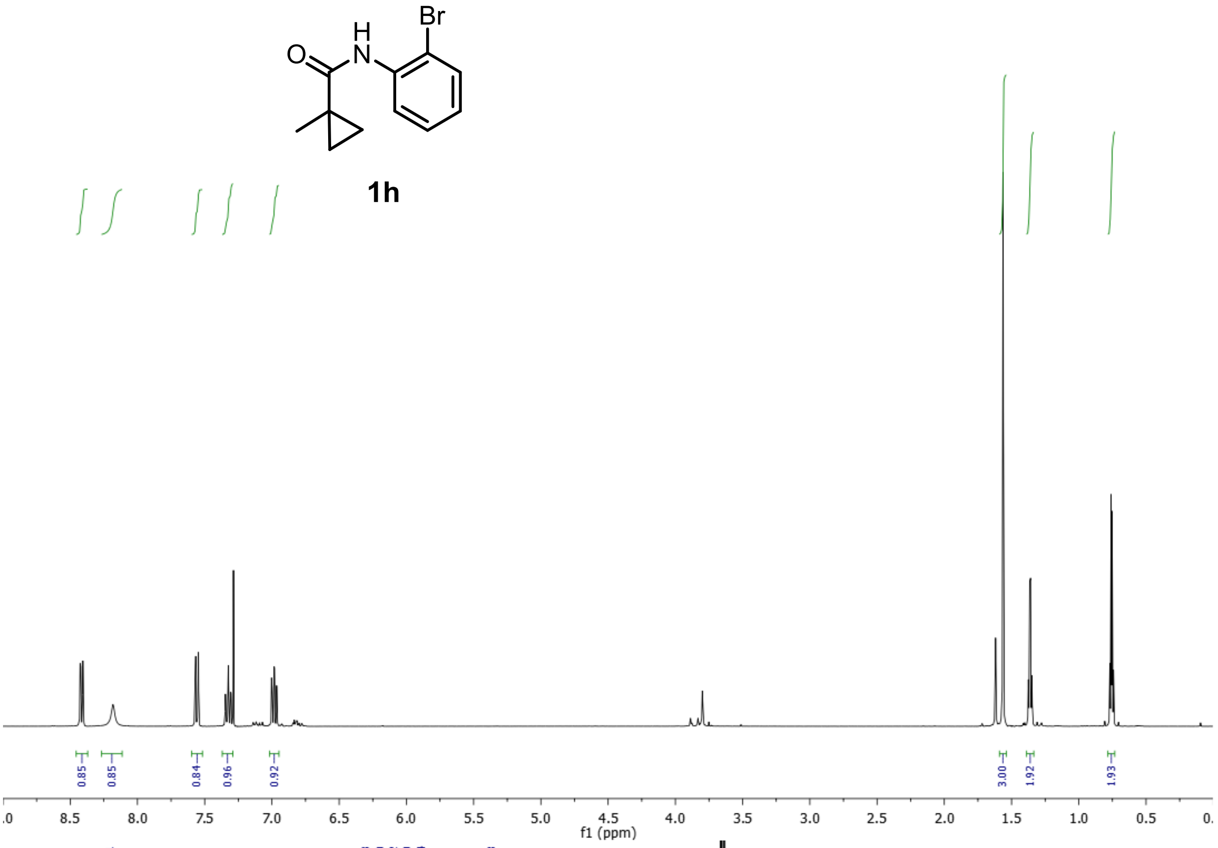
Br
H
N
O
1h
0.85
0.85
0.84
0.96
0.92
3.00
1.92
1.93
8.5 8.0 7.5 7.0 6.5 6.0 5.5 5.0 4.5 4.0 3.5 3.0 2.5 2.0 1.5 1.0 0.5
f1 (ppm)

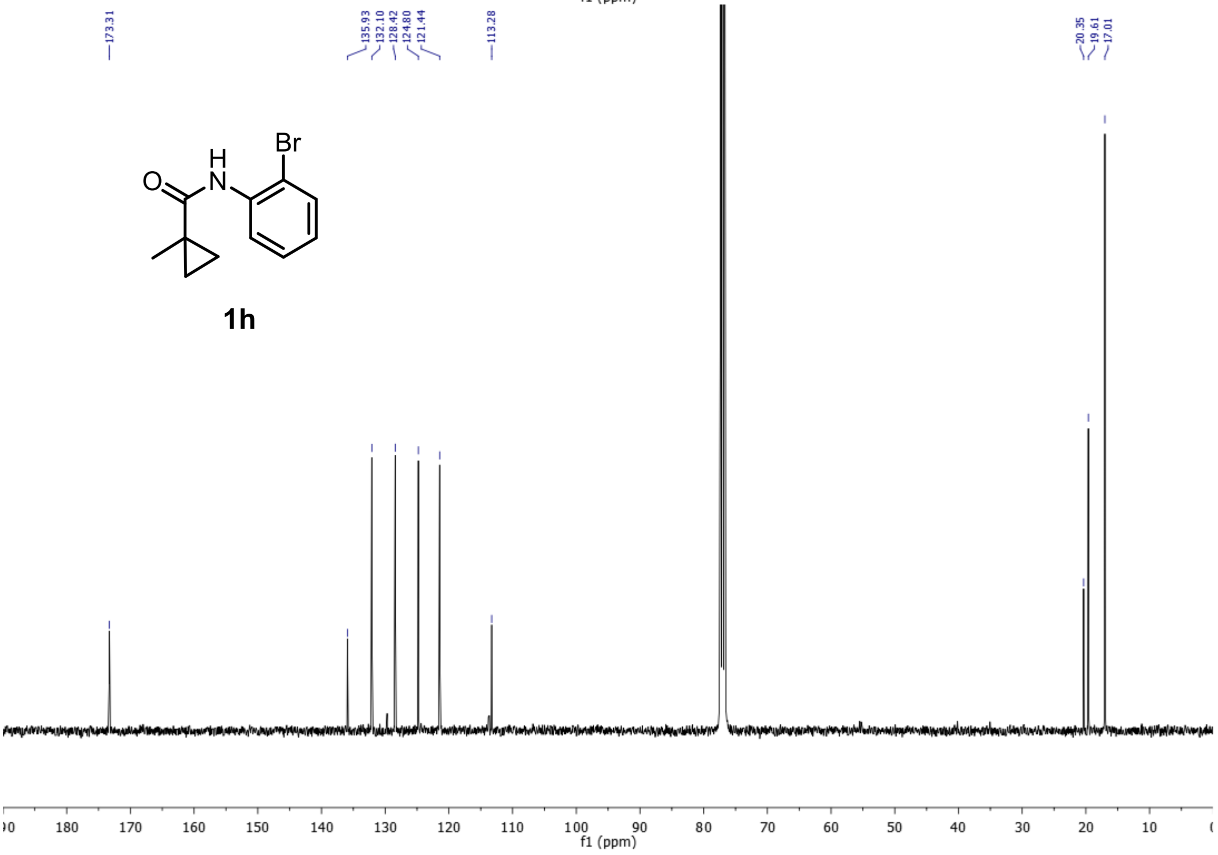
173.31
135.93
132.10
128.42
124.80
121.44
113.28
20.35
19.61
17.01
Br
H
N
O
1h
180 170 160 150 140 130 120 110 100 90 80 70 60 50 40 30 20 10
f1 (ppm)

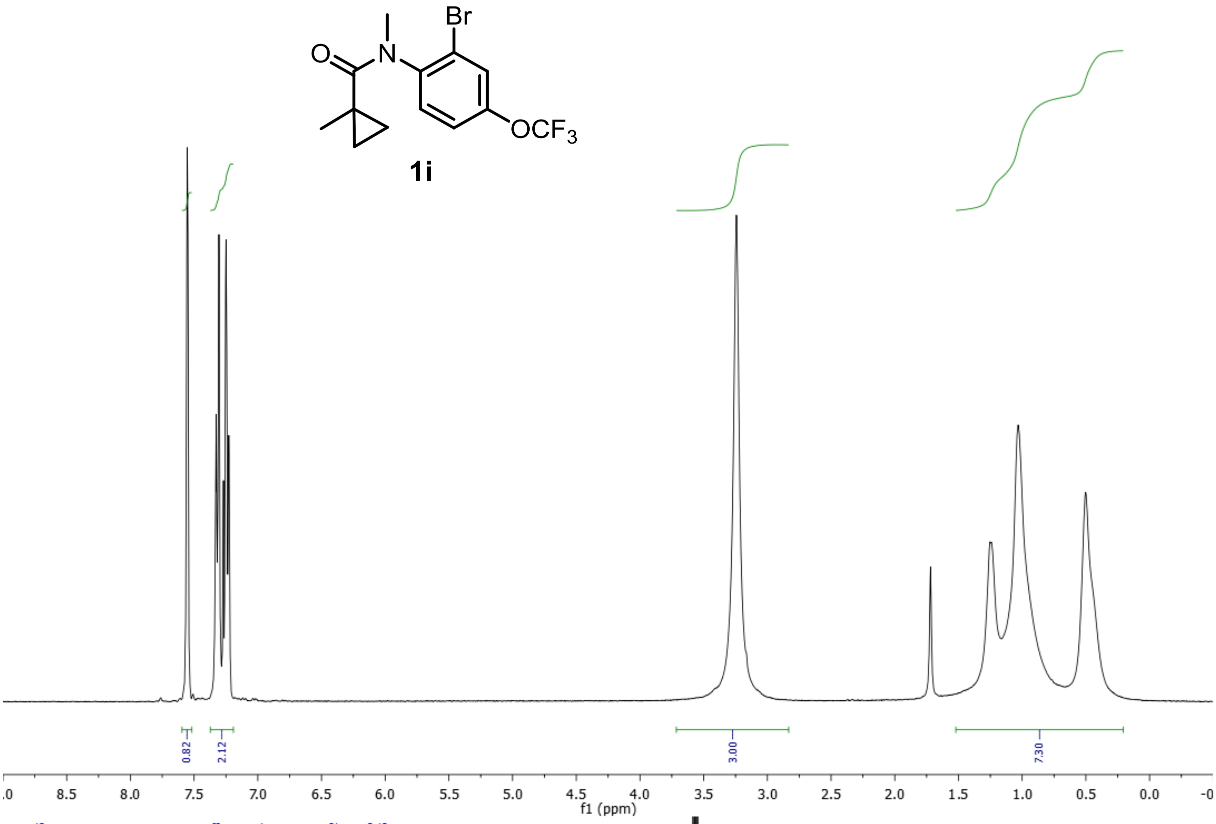
Br
O
N
OCF3
1i
0.82
2.12
3.00
7.30
8.5 8.0 7.5 7.0 6.5 6.0 5.5 5.0 4.5 4.0 3.5 3.0 2.5 2.0 1.5 1.0 0.5 0.0
f1 (ppm)

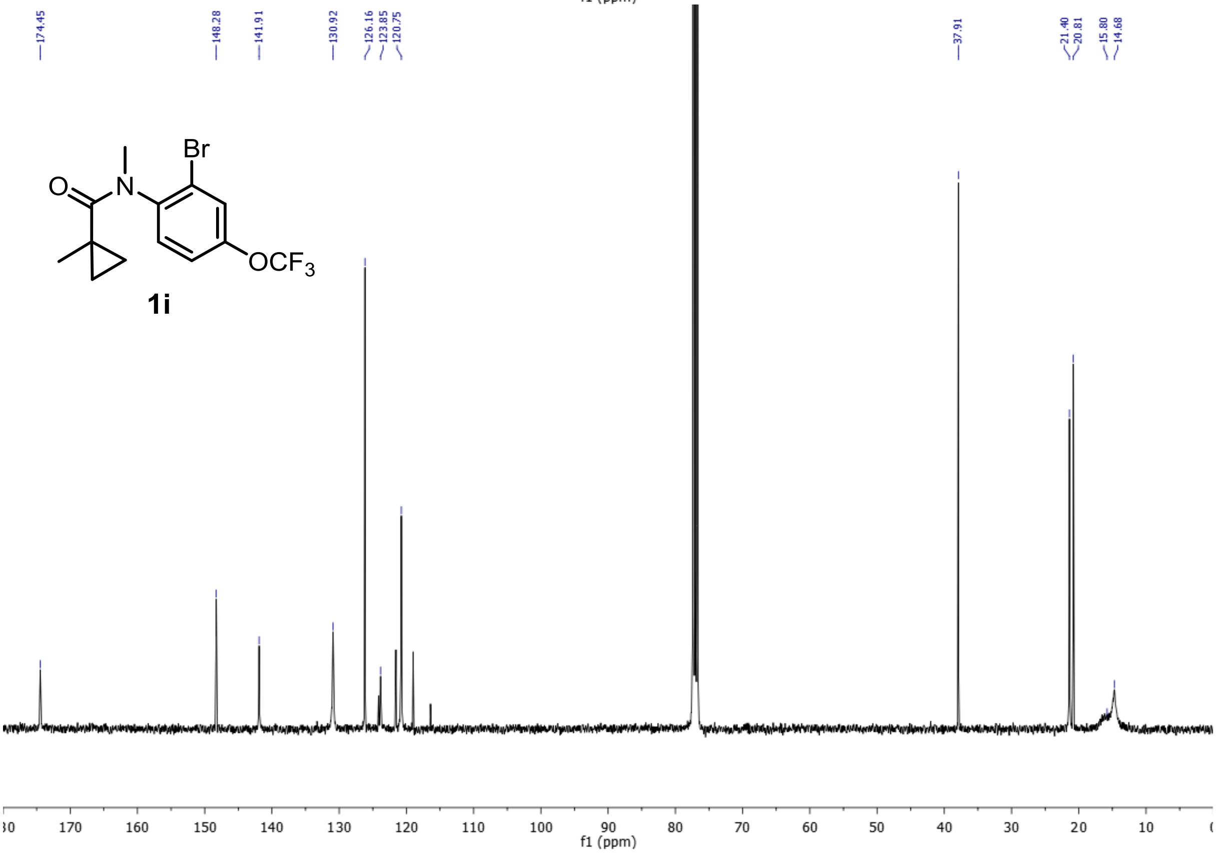
174.45
148.28
141.91
130.92
126.16
123.85
120.75
37.91
21.40
20.81
15.80
14.68
Br
O
N
OCF3
1i
170 160 150 140 130 120 110 100 90 80 70 60 50 40 30 20 10
f1 (ppm)

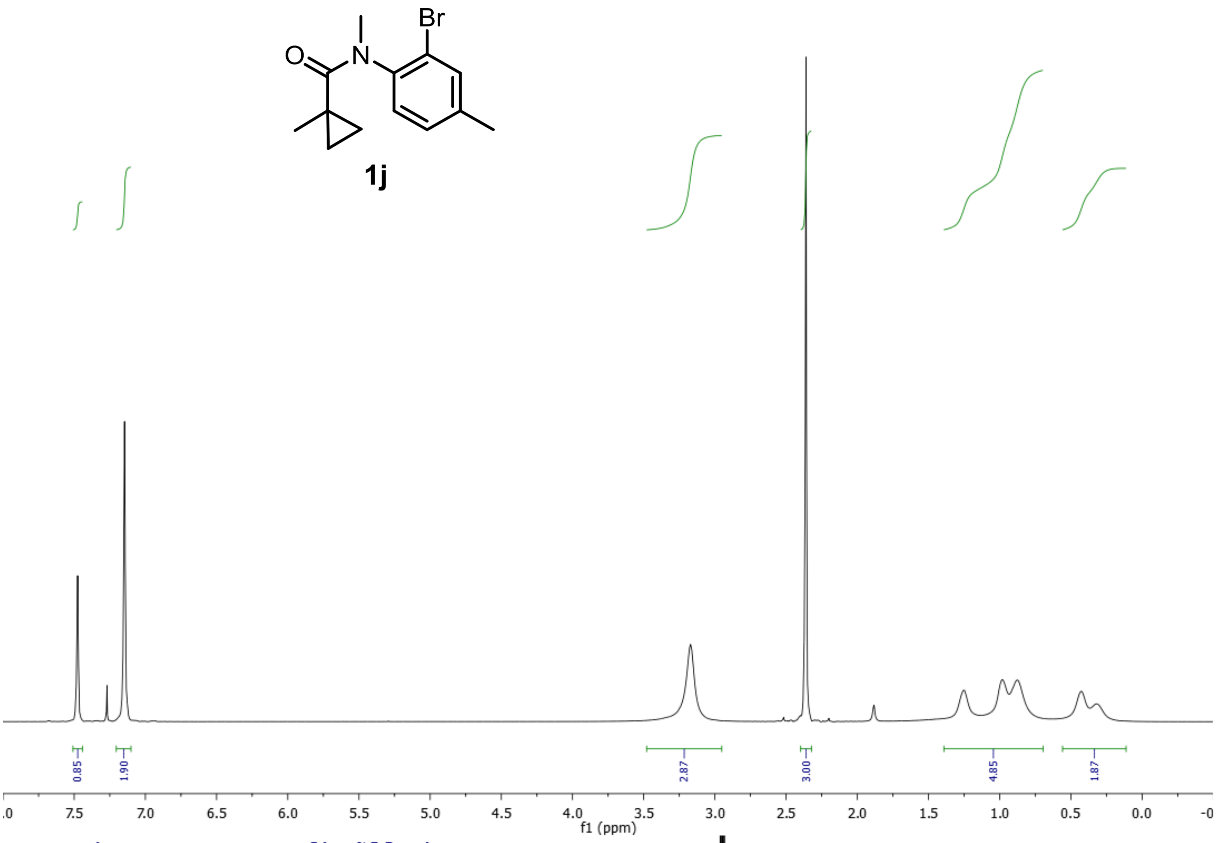

Br
O
N
1j
0.85
1.90
2.87
3.00
4.85
1.87
7.5 7.0 6.5 6.0 5.5 5.0 4.5 4.0 3.5 3.0 2.5 2.0 1.5 1.0 0.5 0.0
f1 (ppm)

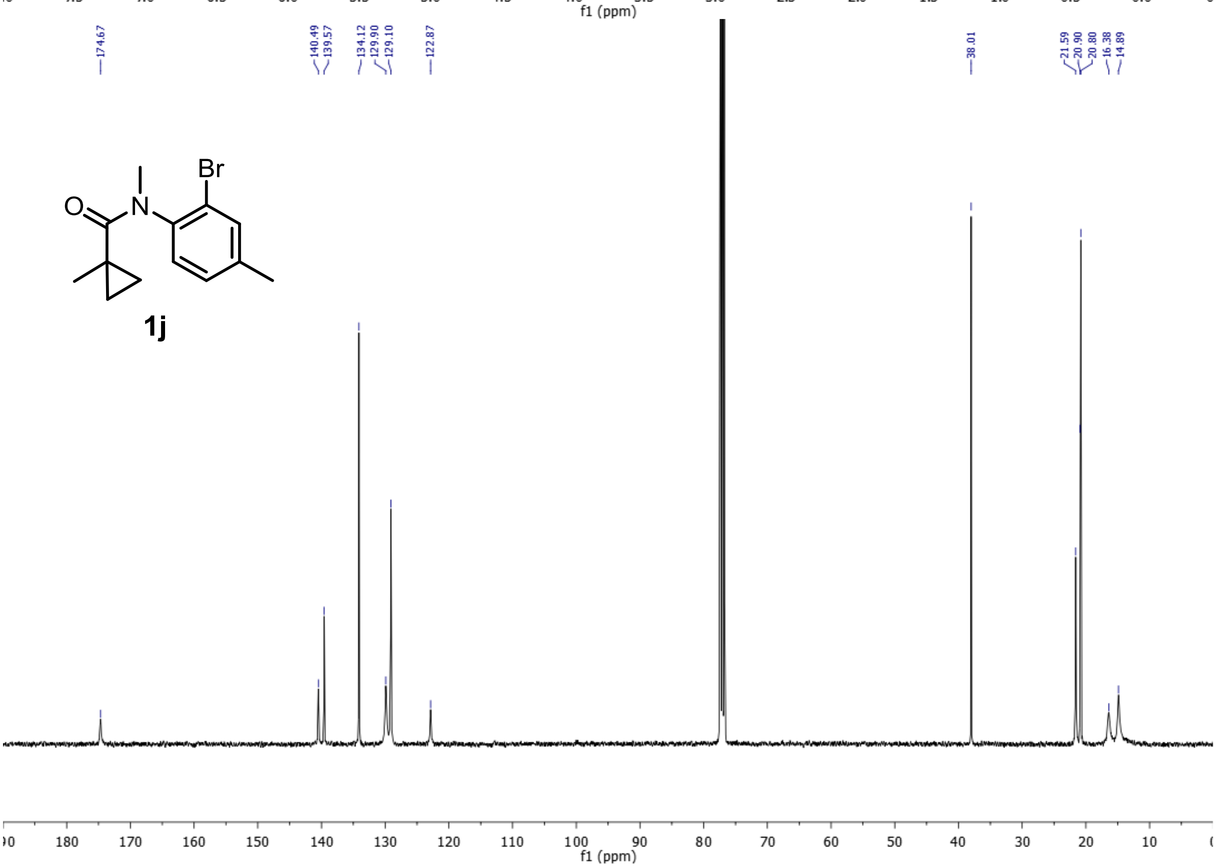

174.67
140.49
139.57
134.12
129.90
129.10
122.87
38.01
21.59
20.90
20.80
16.38
14.89
Br
O
N
1j
180 170 160 150 140 130 120 110 100 90 80 70 60 50 40 30 20 10
f1 (ppm)

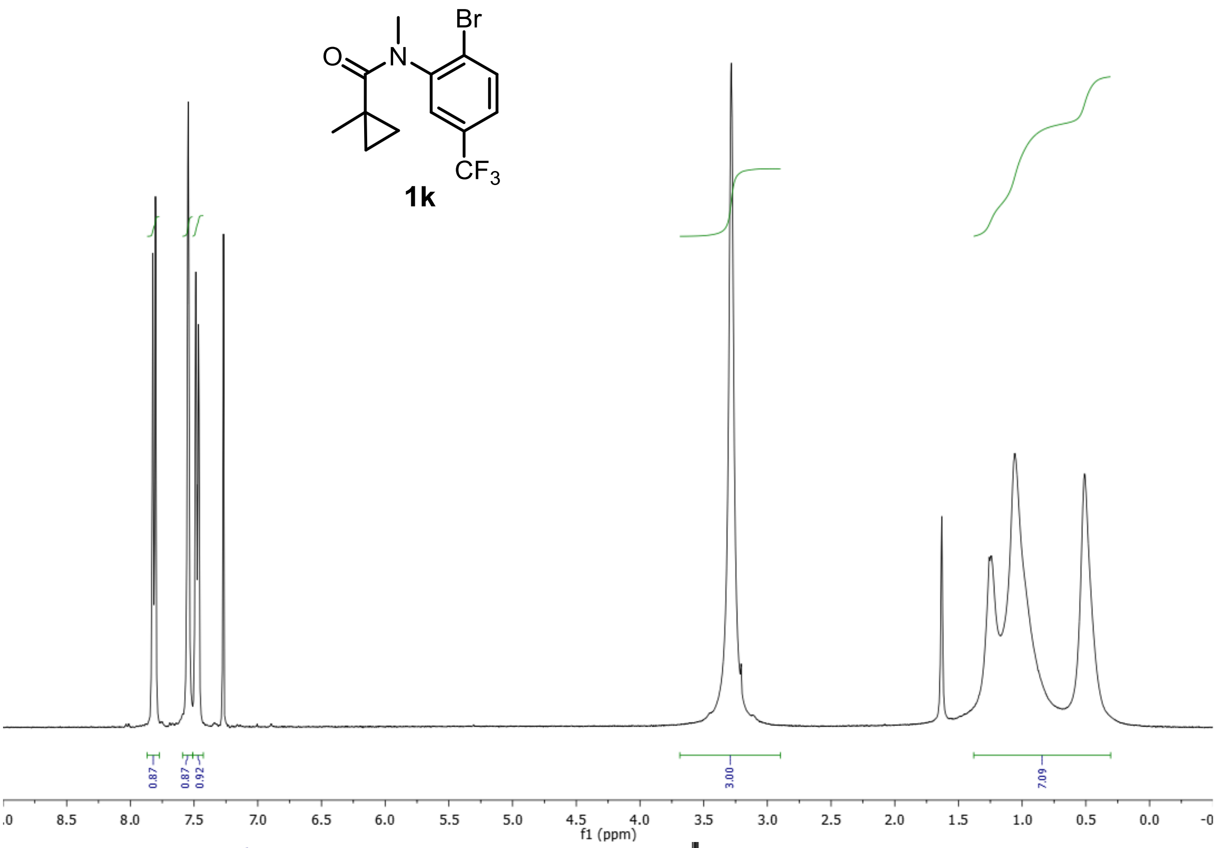

Br
O
N
CF3
1k
f1 (ppm)

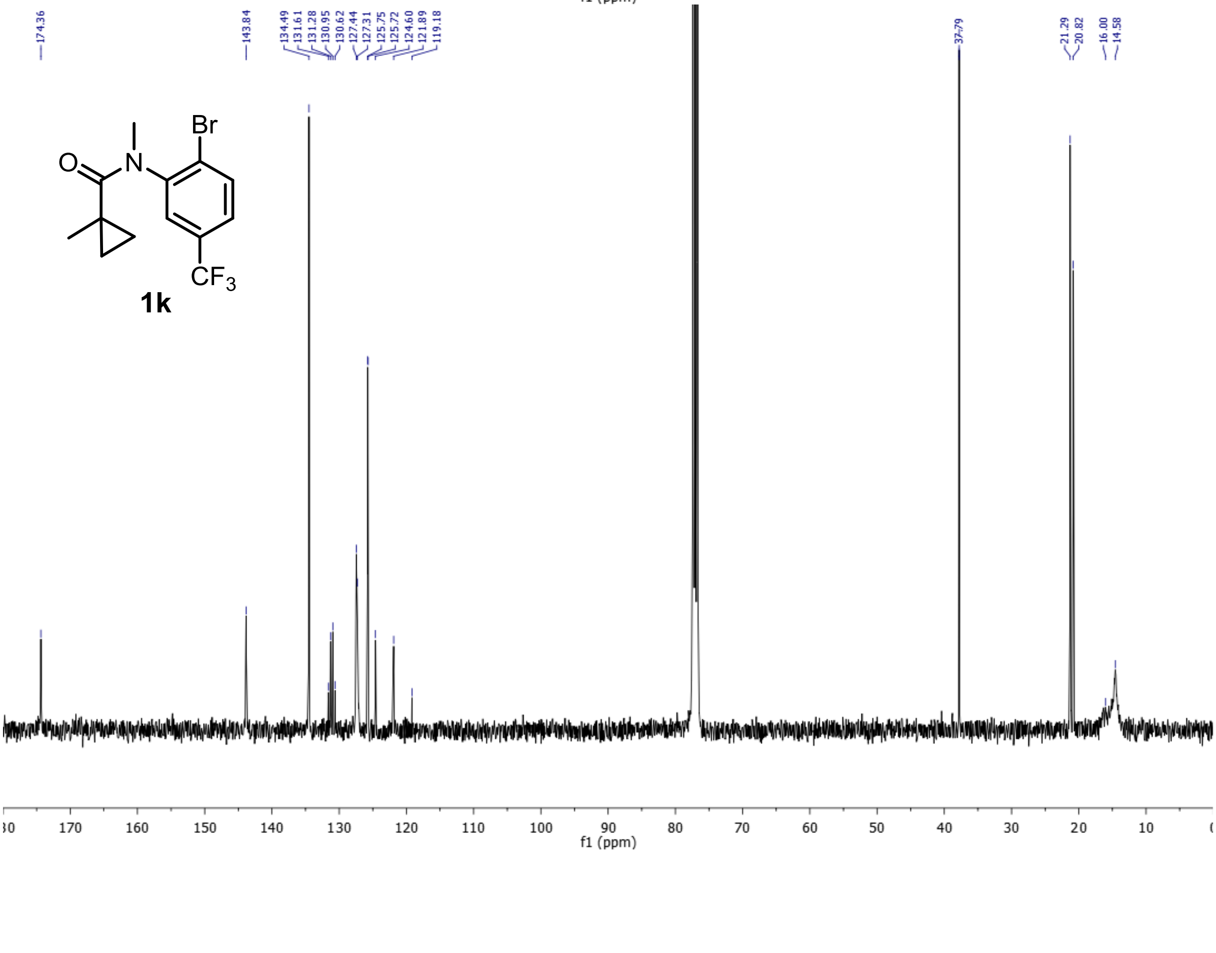

Br
O
N
CF3
1k
f1 (ppm)

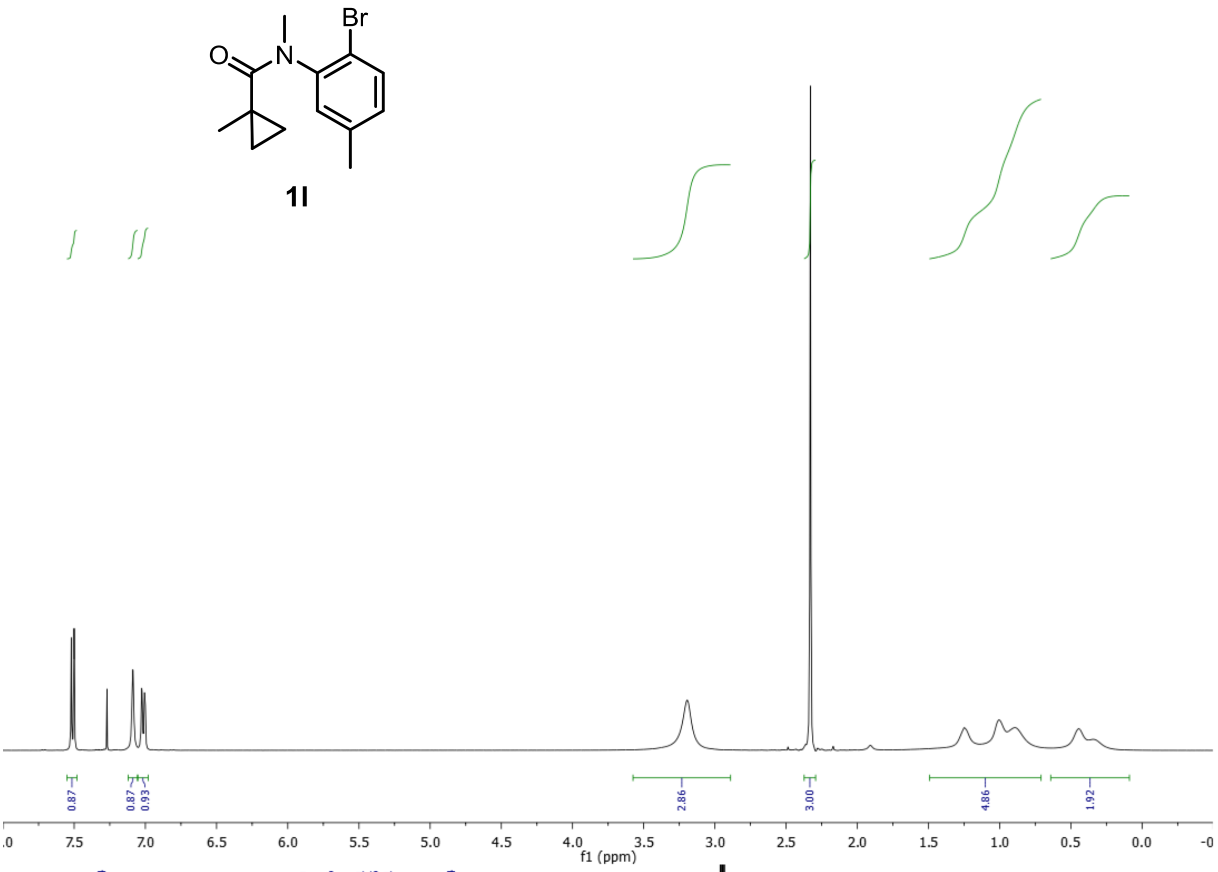
Br
O
N
1l
0.87
0.87
0.93
2.86
3.00
4.86
1.92
7.5
7.0
6.5
6.0
5.5
5.0
4.5
4.0
3.5
3.0
2.5
2.0
1.5
1.0
0.5
0.0
f1 (ppm)

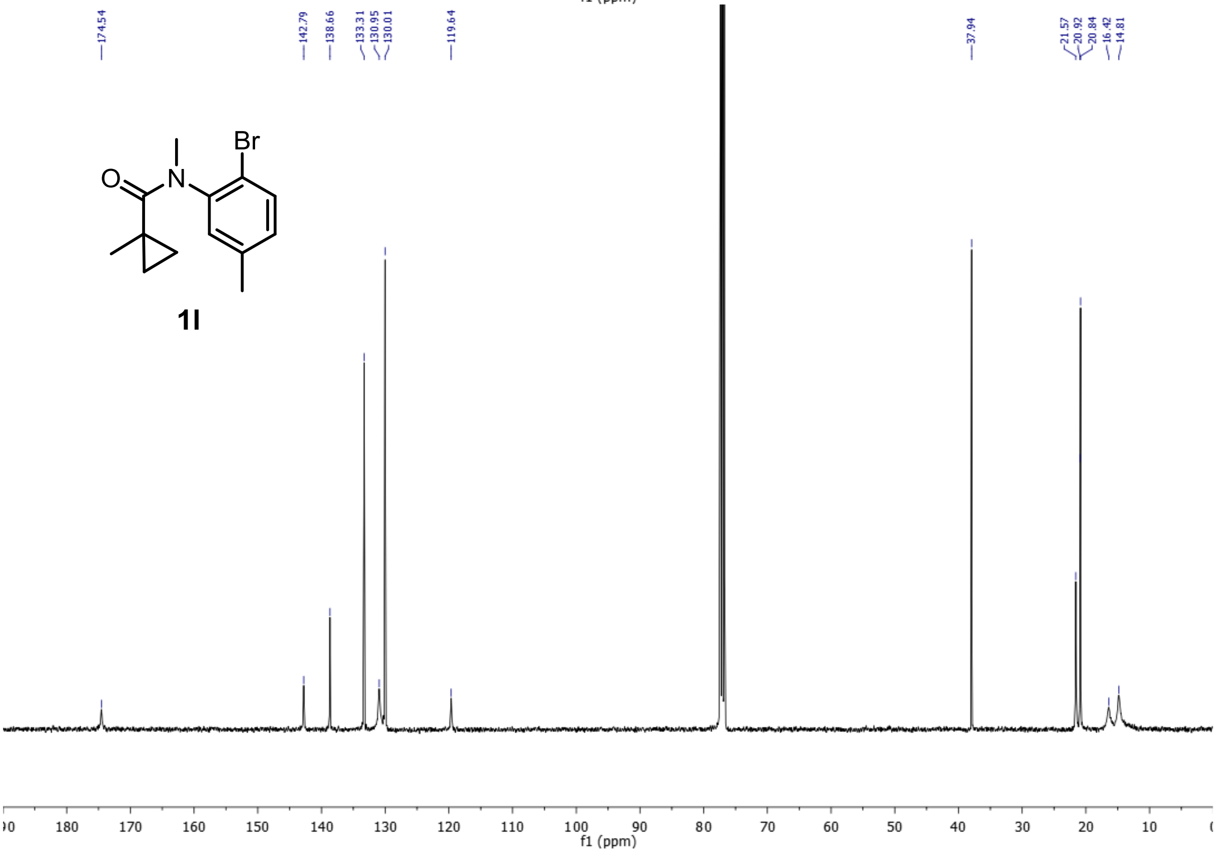
174.54
142.79
138.66
133.31
130.95
130.01
119.64
37.94
21.57
20.92
20.84
16.42
14.81
Br
O
N
1l
180
170
160
150
140
130
120
110
100
90
80
70
60
50
40
30
20
10
f1 (ppm)

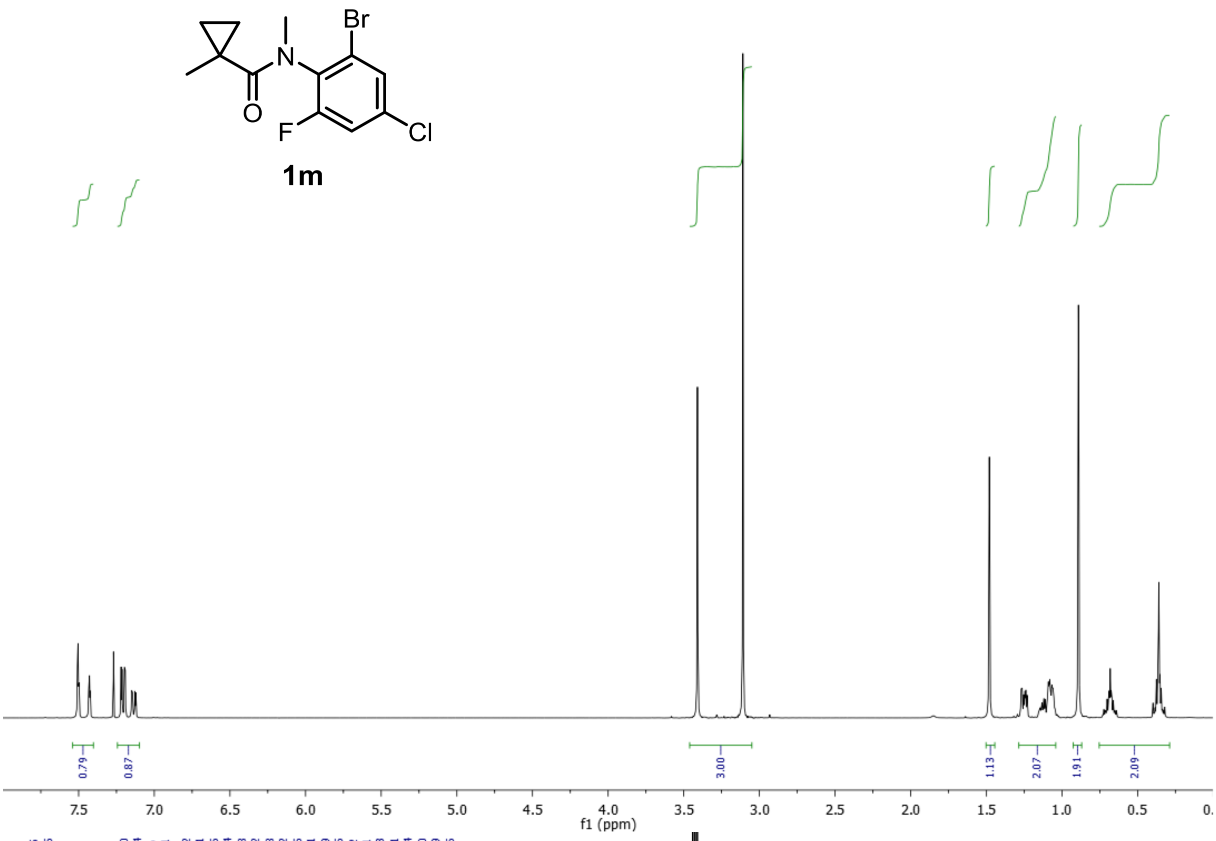
Br
N
O
F
Cl
1m
0.79
0.87
3.00
1.13
2.07
1.91
2.09
f1 (ppm)

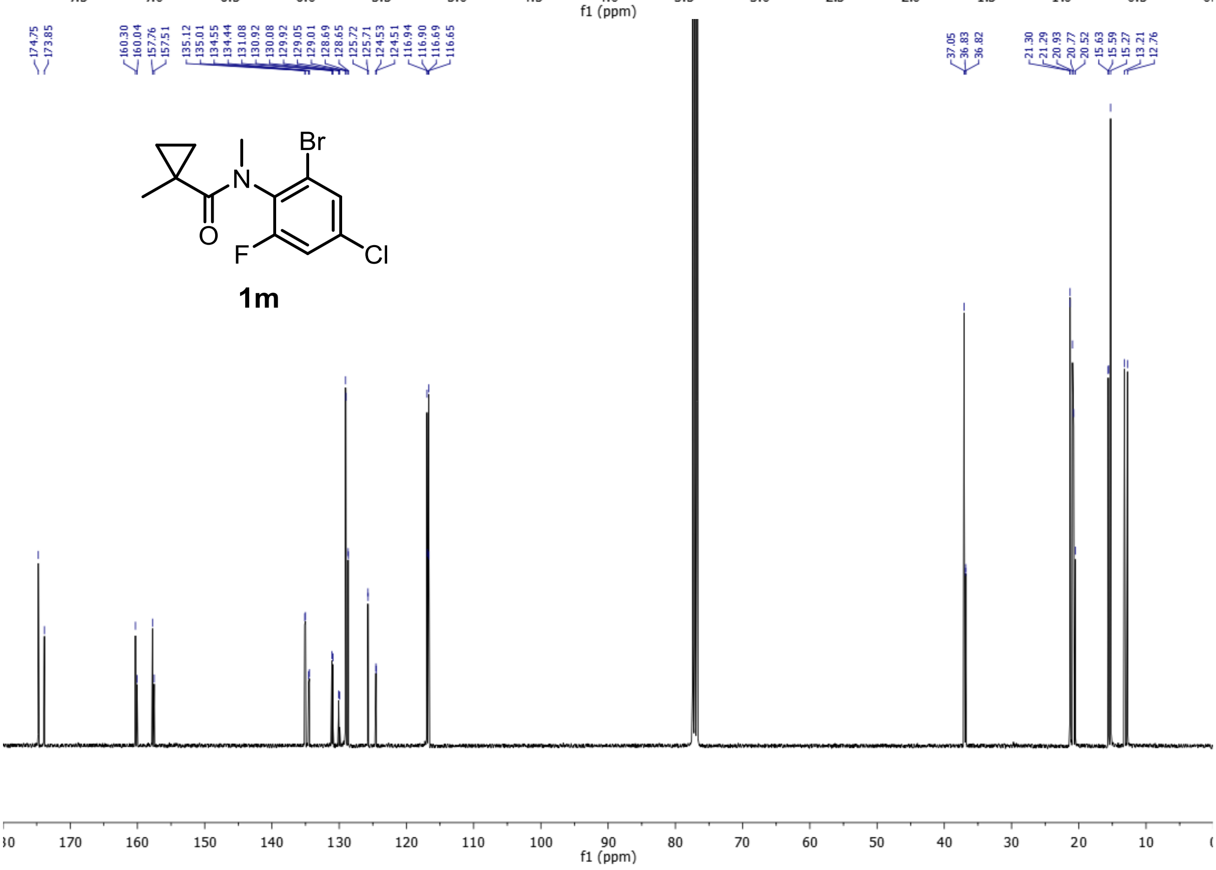
174.75
173.85
160.30
160.04
157.76
157.51
135.12
135.01
134.55
134.44
131.08
130.92
130.08
129.92
129.05
129.01
128.69
128.65
125.72
125.71
124.53
124.51
116.94
116.90
116.69
116.65
37.05
36.83
36.82
21.30
21.29
20.93
20.77
20.52
15.63
15.59
15.27
13.21
12.76
Br
N
O
F
Cl
1m
f1 (ppm)

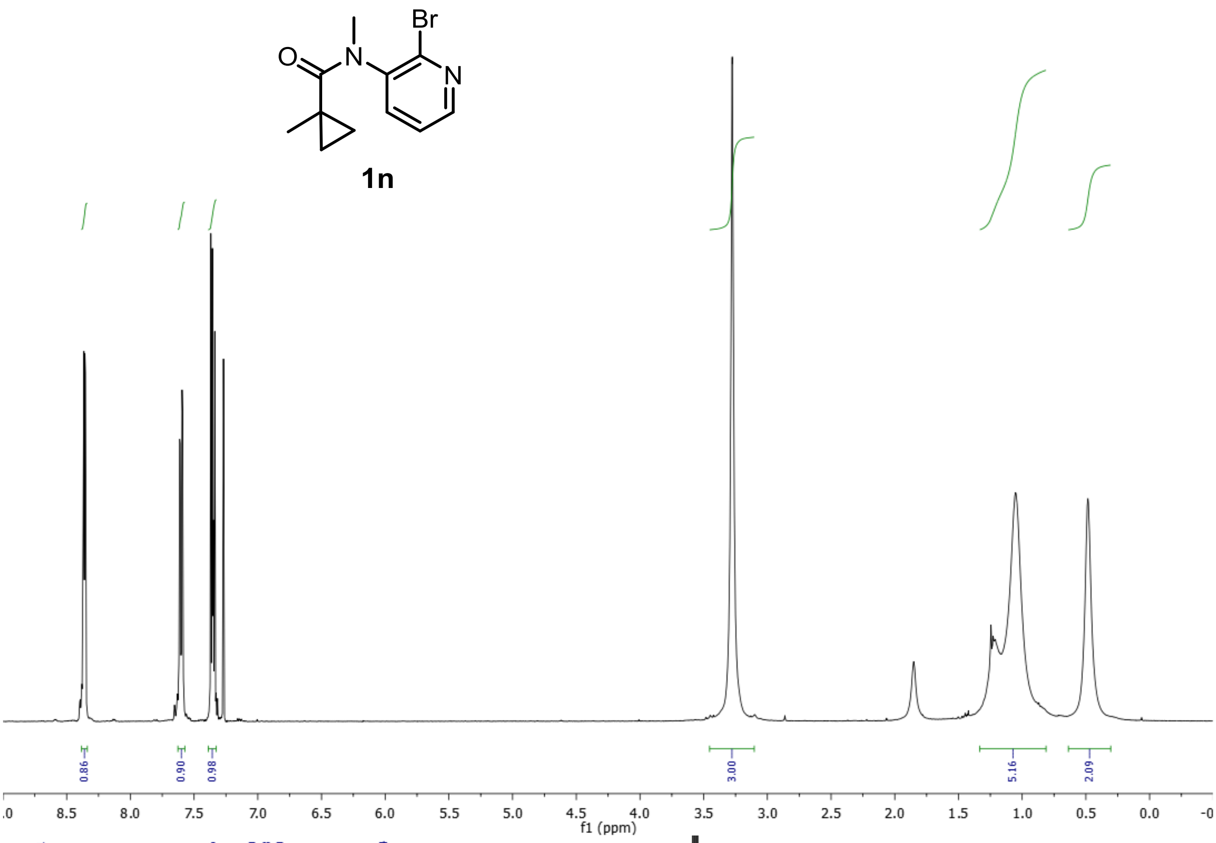
Br
O
N
N
1n
0.86
0.90
0.98
3.00
5.16
2.09
f1 (ppm)

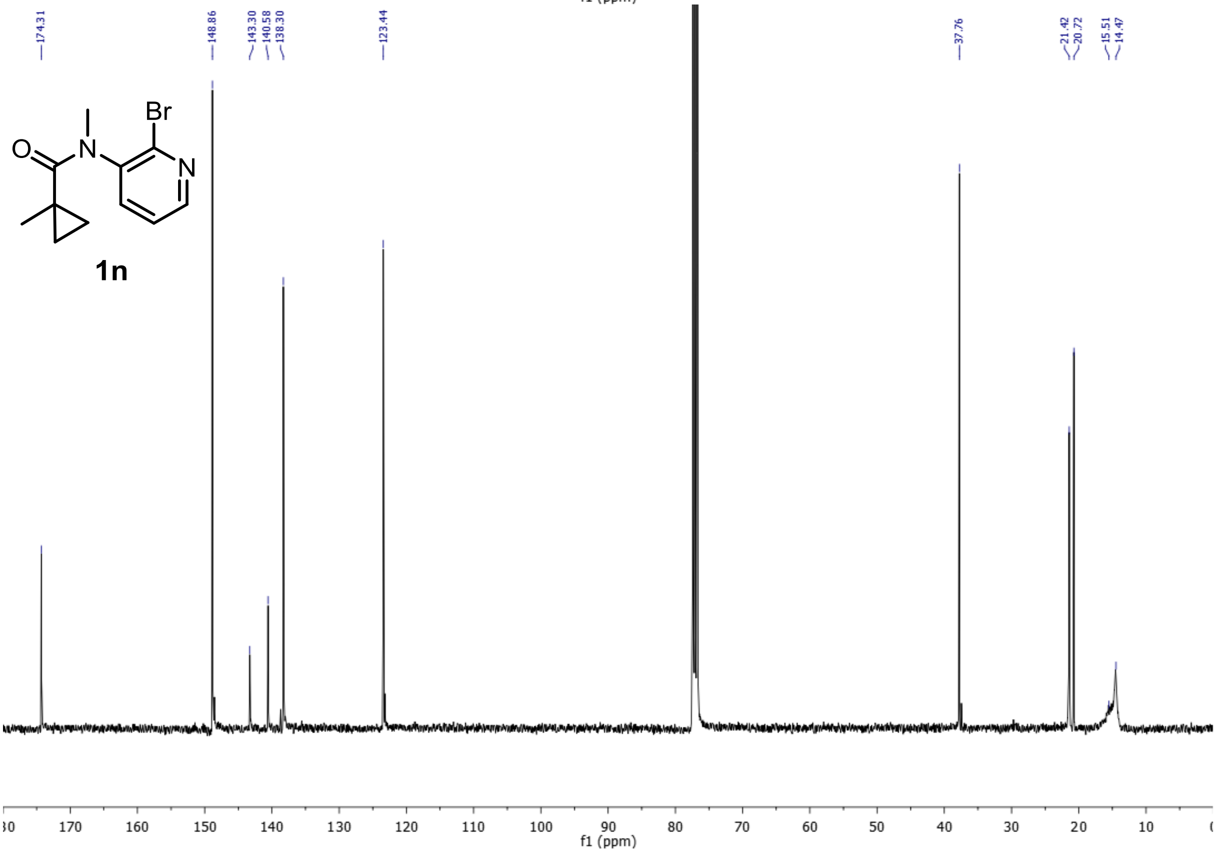
174.31
148.86
143.30
140.58
138.30
123.44
37.76
21.42
20.72
15.51
14.47
Br
O
N
N
1n
f1 (ppm)

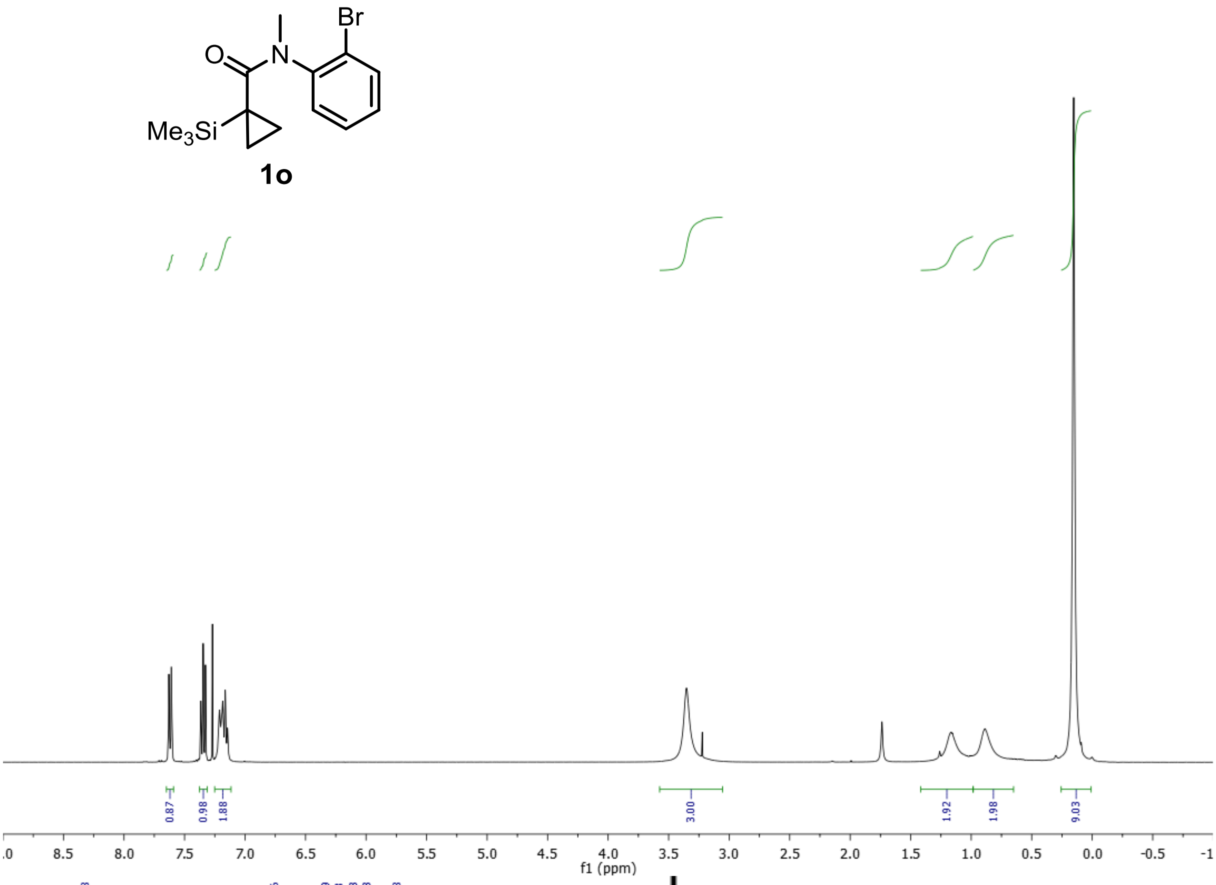
Br
O
N
Me3Si
1o
0.87
0.98
1.88
3.00
1.92
1.98
9.03
f1 (ppm)

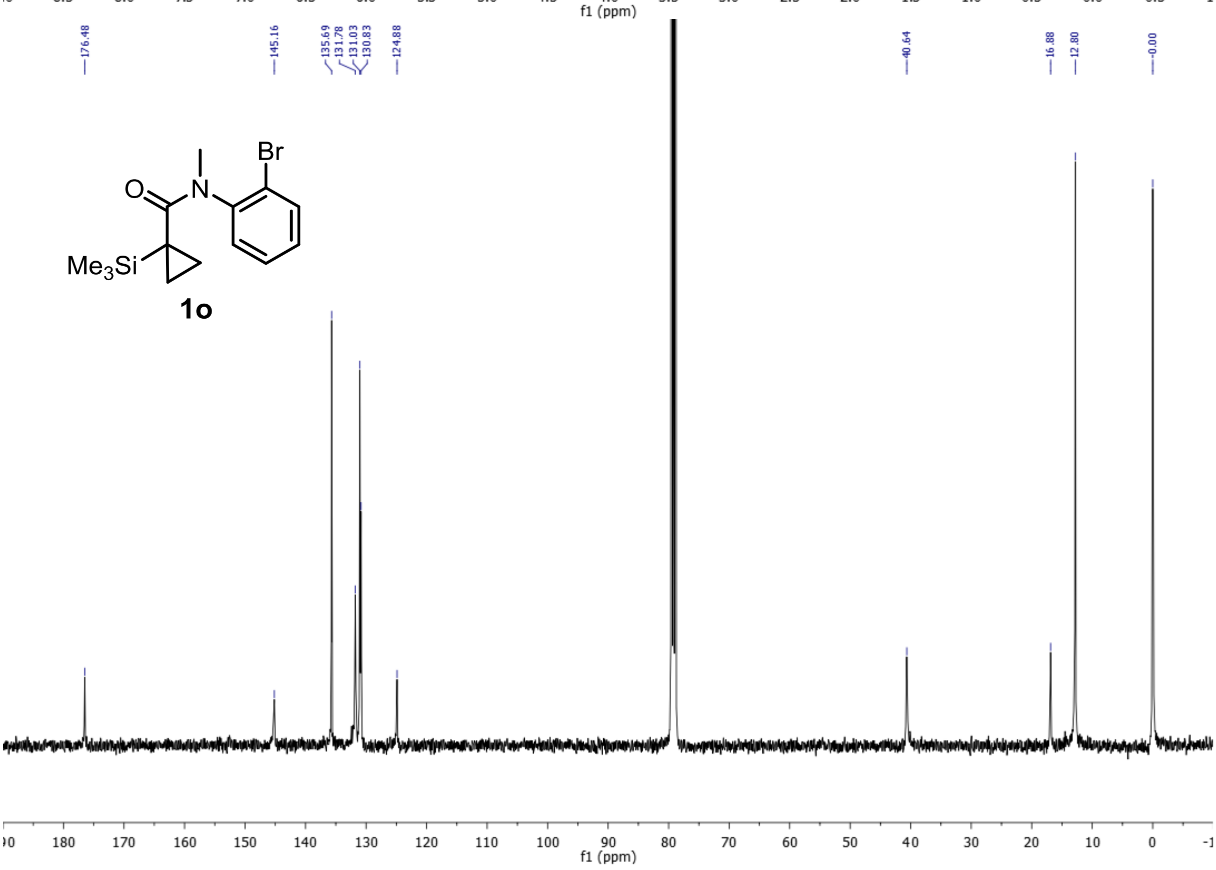
176.48
145.16
135.69
131.78
131.03
130.83
124.88
40.64
16.88
12.80
0.00
Br
O
N
Me3Si
1o
f1 (ppm)

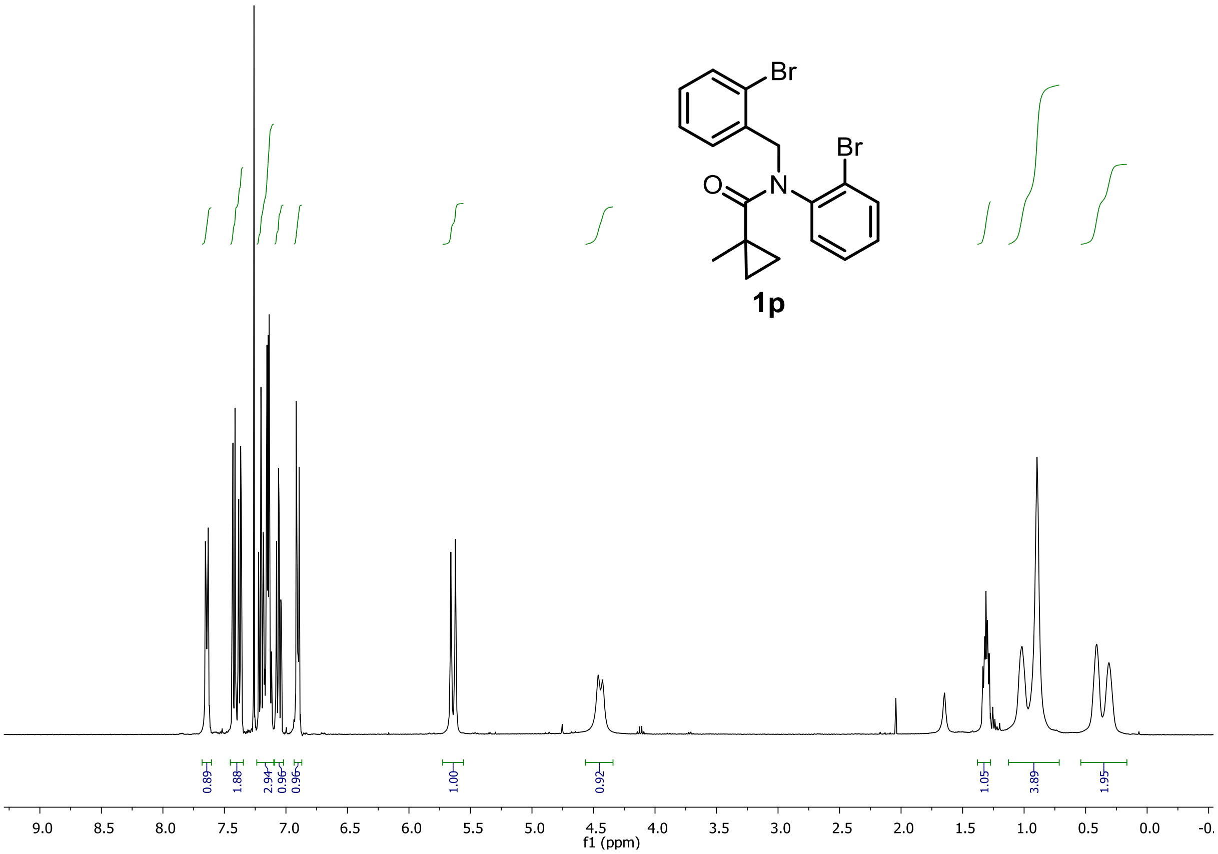
Br
Br
O
N
1p
0.89
1.88
2.94
0.96
0.96
1.00
0.92
1.05
3.89
1.95
9.0 8.5 8.0 7.5 7.0 6.5 6.0 5.5 5.0 4.5 4.0 3.5 3.0 2.5 2.0 1.5 1.0 0.5 0.0
f1 (ppm)

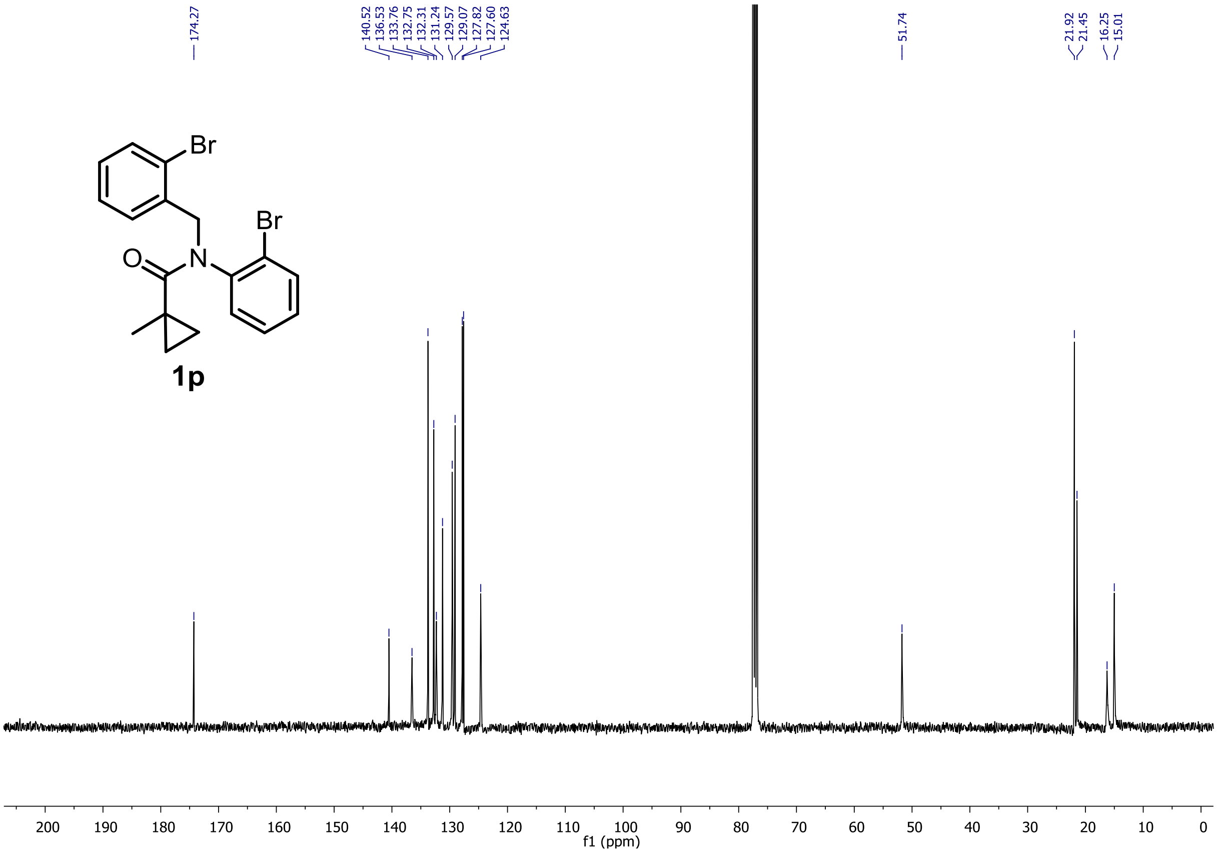
174.27
140.52
136.53
133.76
132.75
132.31
131.24
129.57
129.07
127.82
127.60
124.63
51.74
21.92
21.45
16.25
15.01
Br
Br
O
N
1p
200 190 180 170 160 150 140 130 120 110 100 90 80 70 60 50 40 30 20 10 0
f1 (ppm)

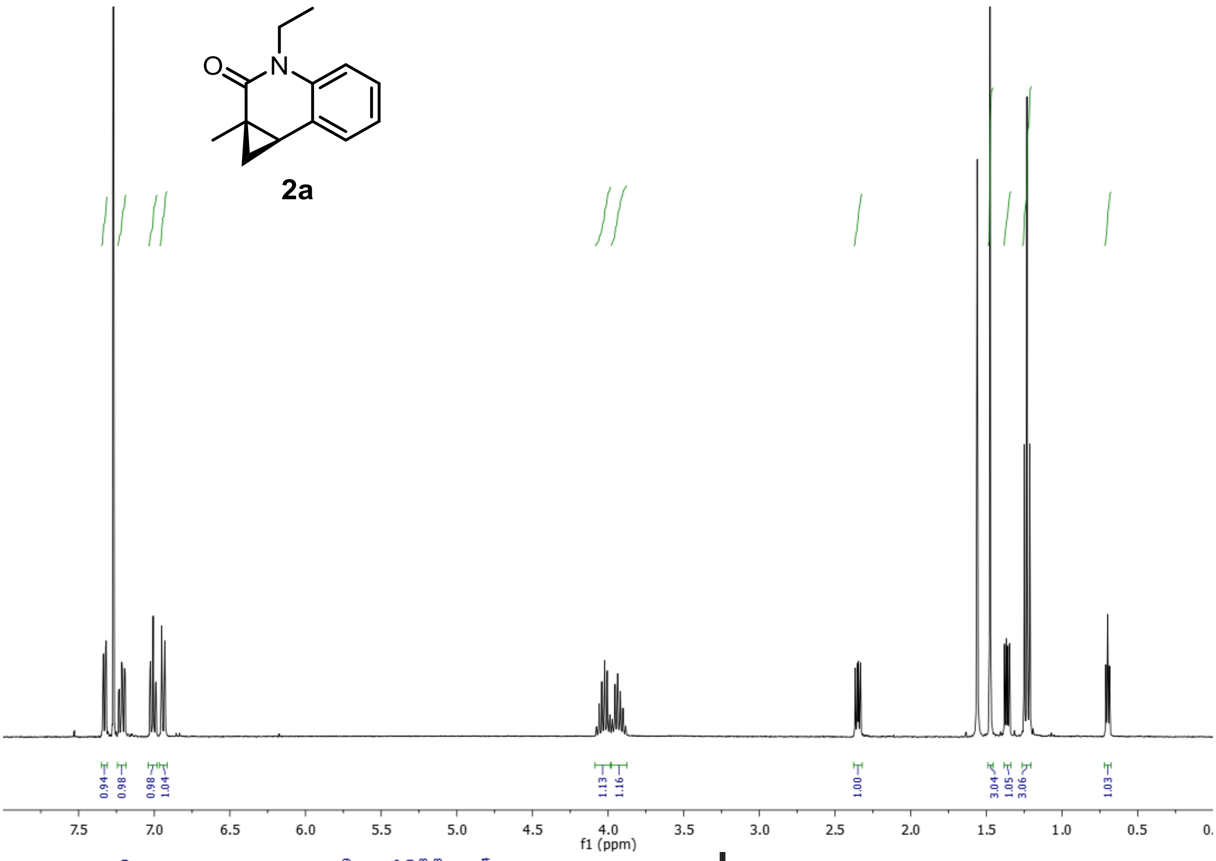

2a
0.94
0.98
0.98
1.04
1.13
1.16
1.00
3.04
1.05
3.06
1.03
7.5 7.0 6.5 6.0 5.5 5.0 4.5 4.0 3.5 3.0 2.5 2.0 1.5 1.0 0.5
f1 (ppm)

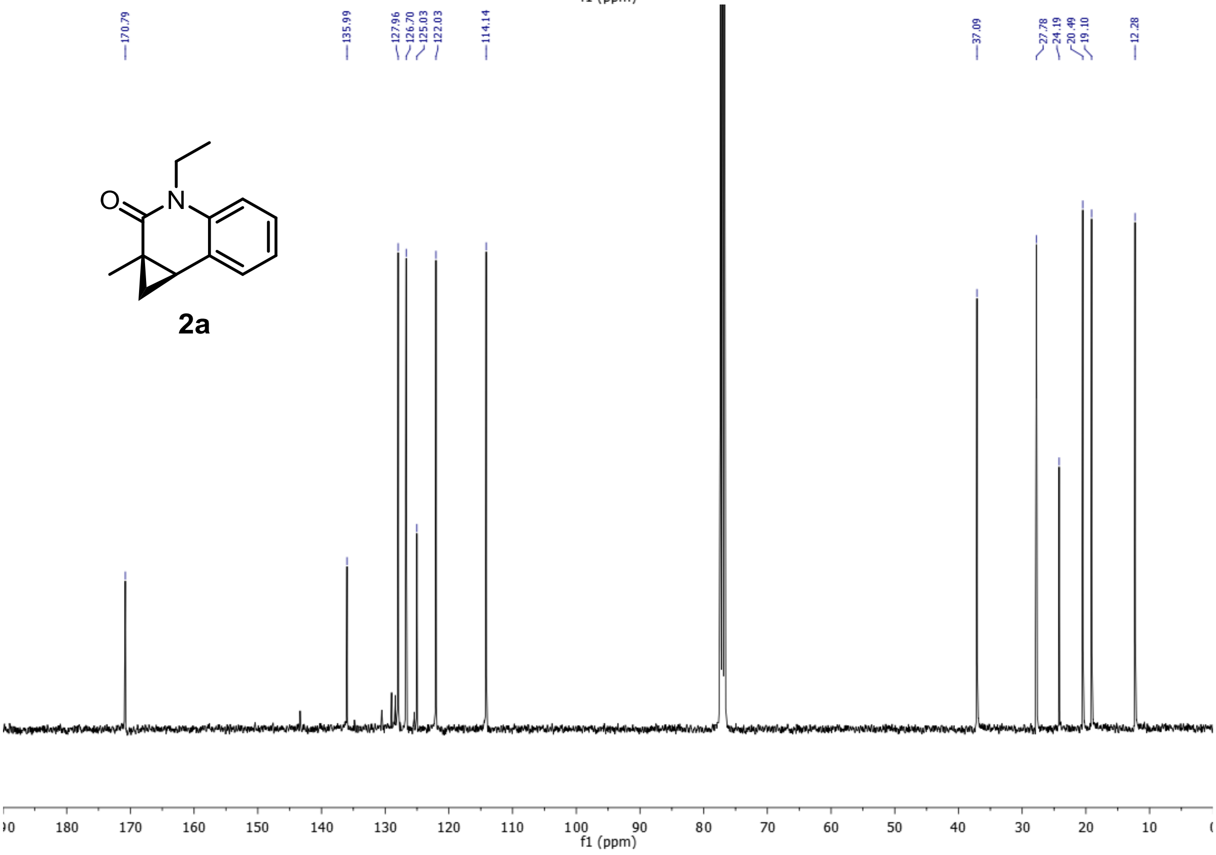

170.79
135.99
127.96
126.70
125.03
122.03
114.14
37.09
27.78
24.19
20.49
19.10
12.28
2a
180 170 160 150 140 130 120 110 100 90 80 70 60 50 40 30 20 10
f1 (ppm)

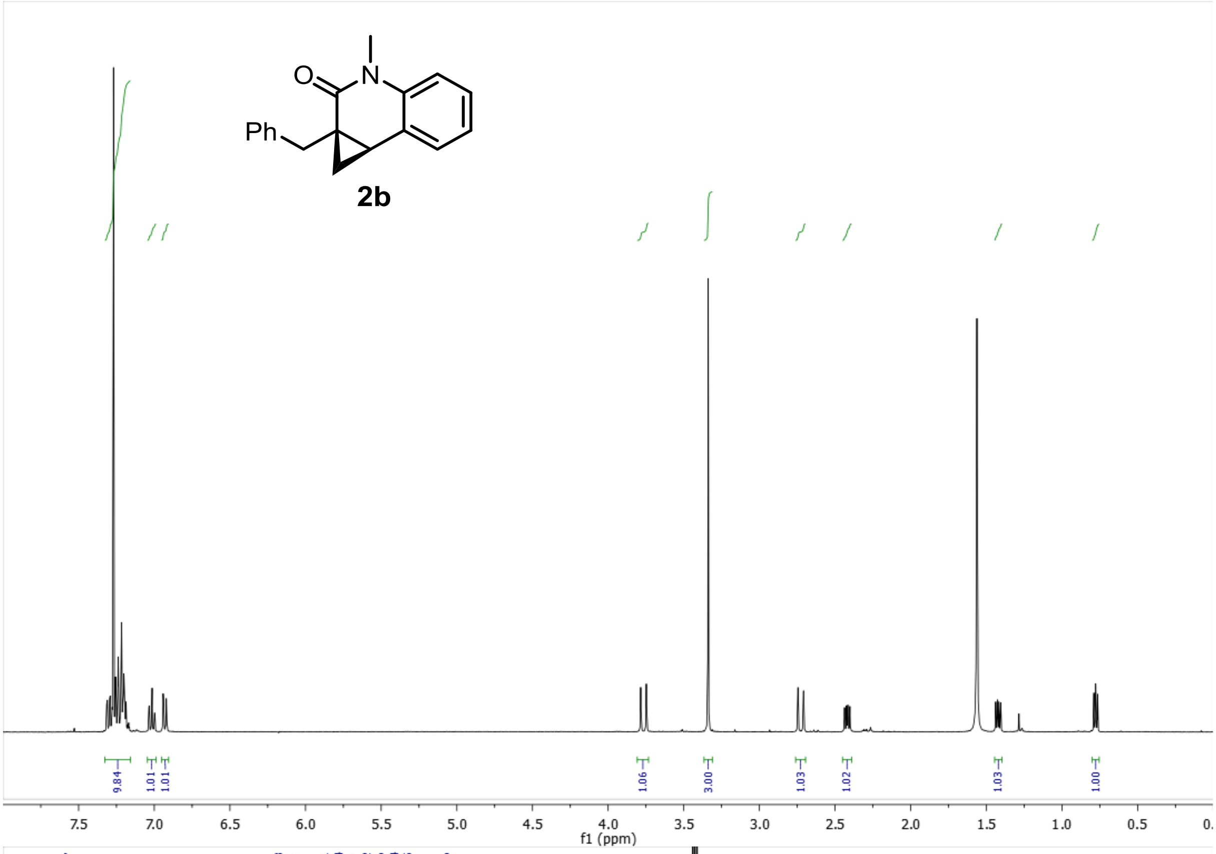

Ph
O
N
2b
9.84
1.01
1.01
1.06
3.00
1.03
1.02
1.03
1.00
f1 (ppm)

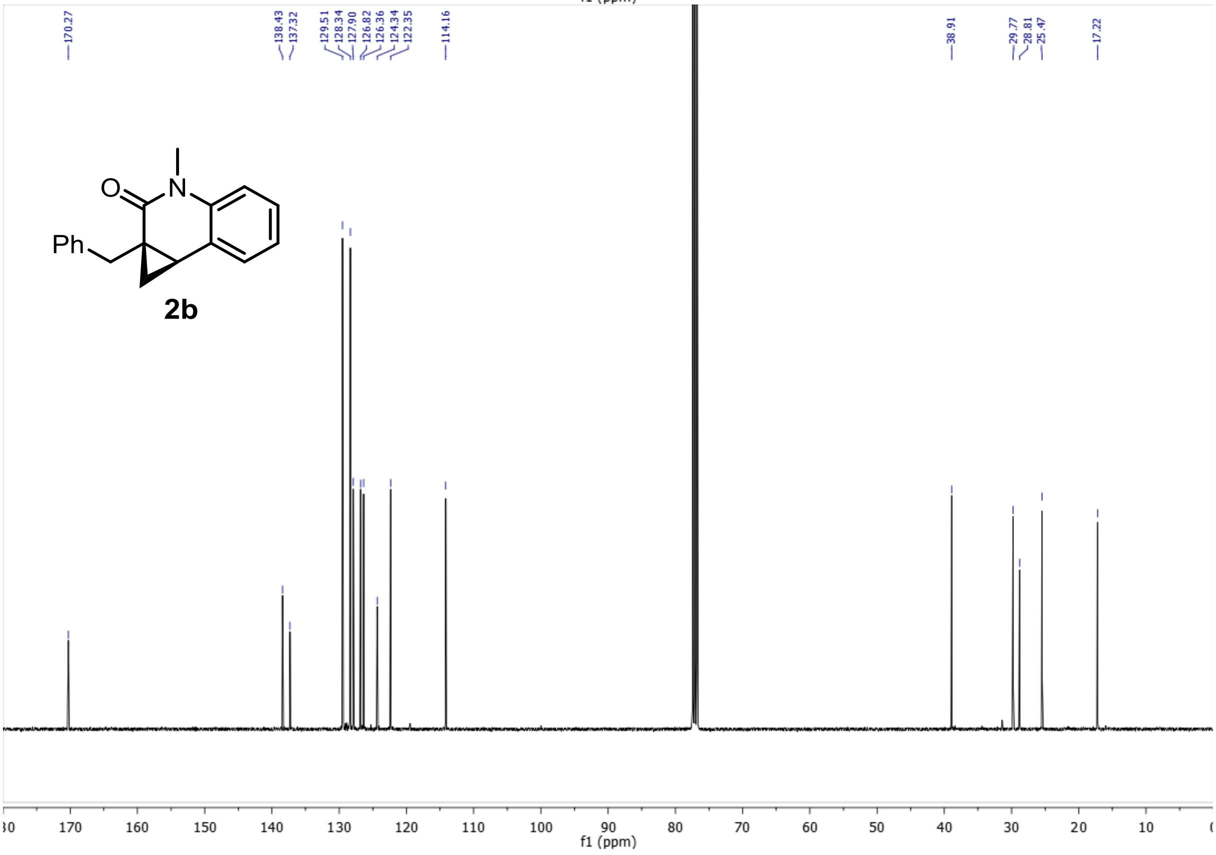

170.27
138.43
137.32
129.51
128.34
127.90
126.82
126.36
124.34
122.35
114.16
38.91
29.77
28.81
25.47
17.22
Ph
O
N
2b
f1 (ppm)

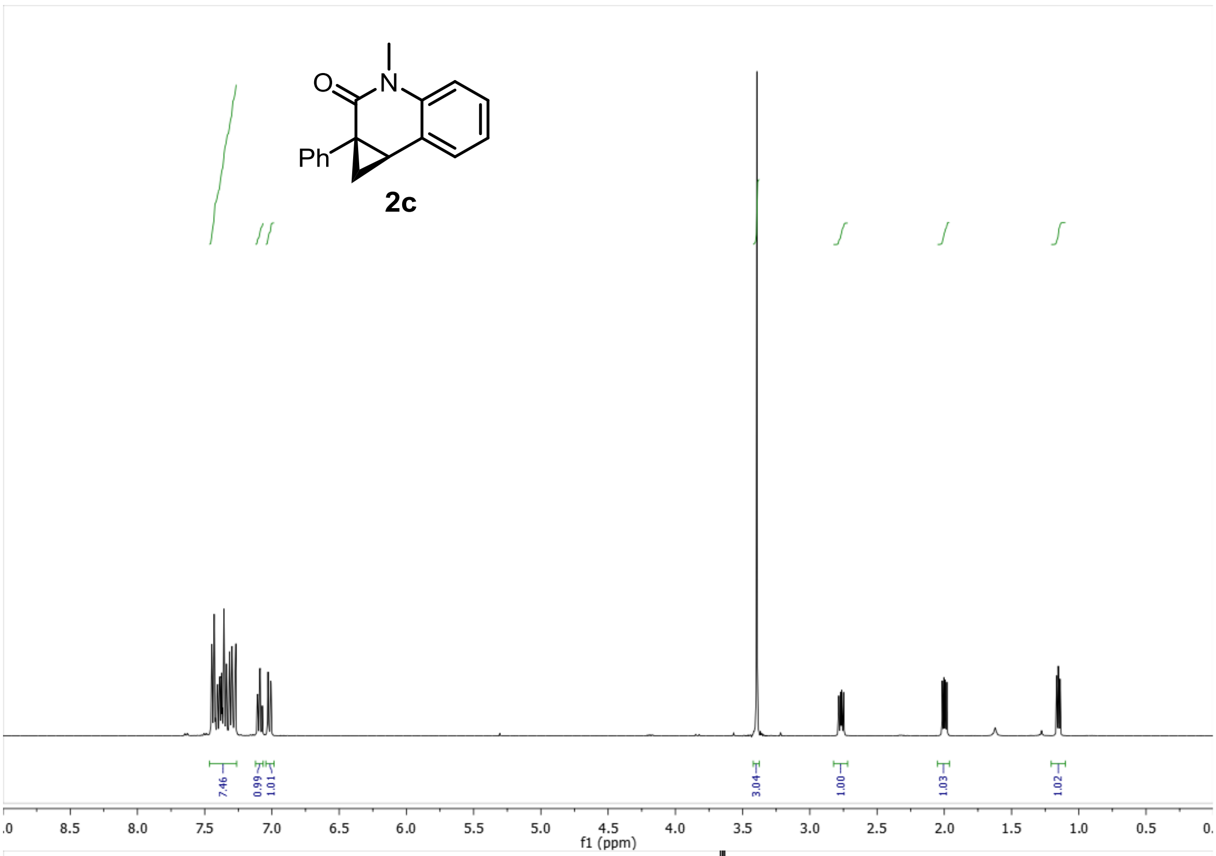
O
N
Ph
2c
7.46
0.99
1.01
3.04
1.00
1.03
1.02
f1 (ppm)

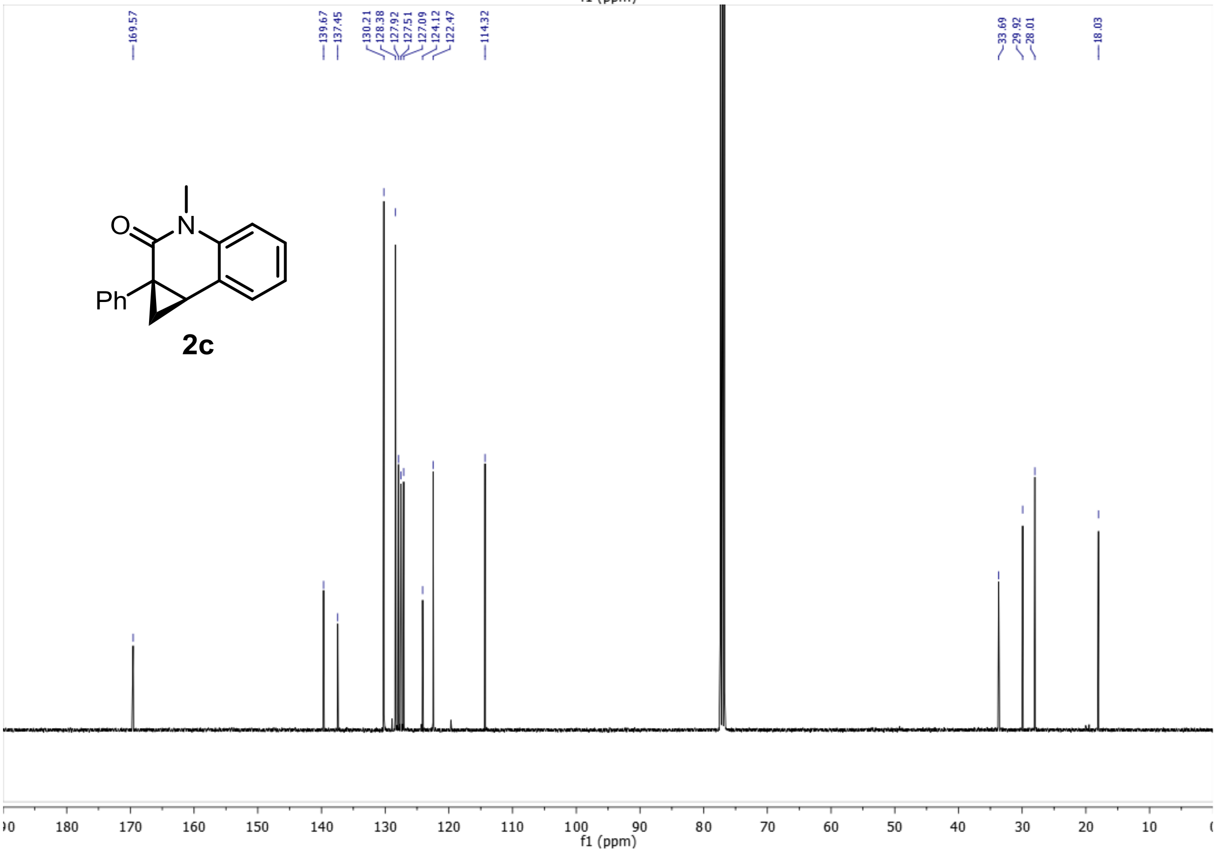
169.57
139.67
137.45
130.21
128.38
127.92
127.51
127.09
124.12
122.47
114.32
33.69
29.92
28.01
18.03
O
N
Ph
2c
f1 (ppm)

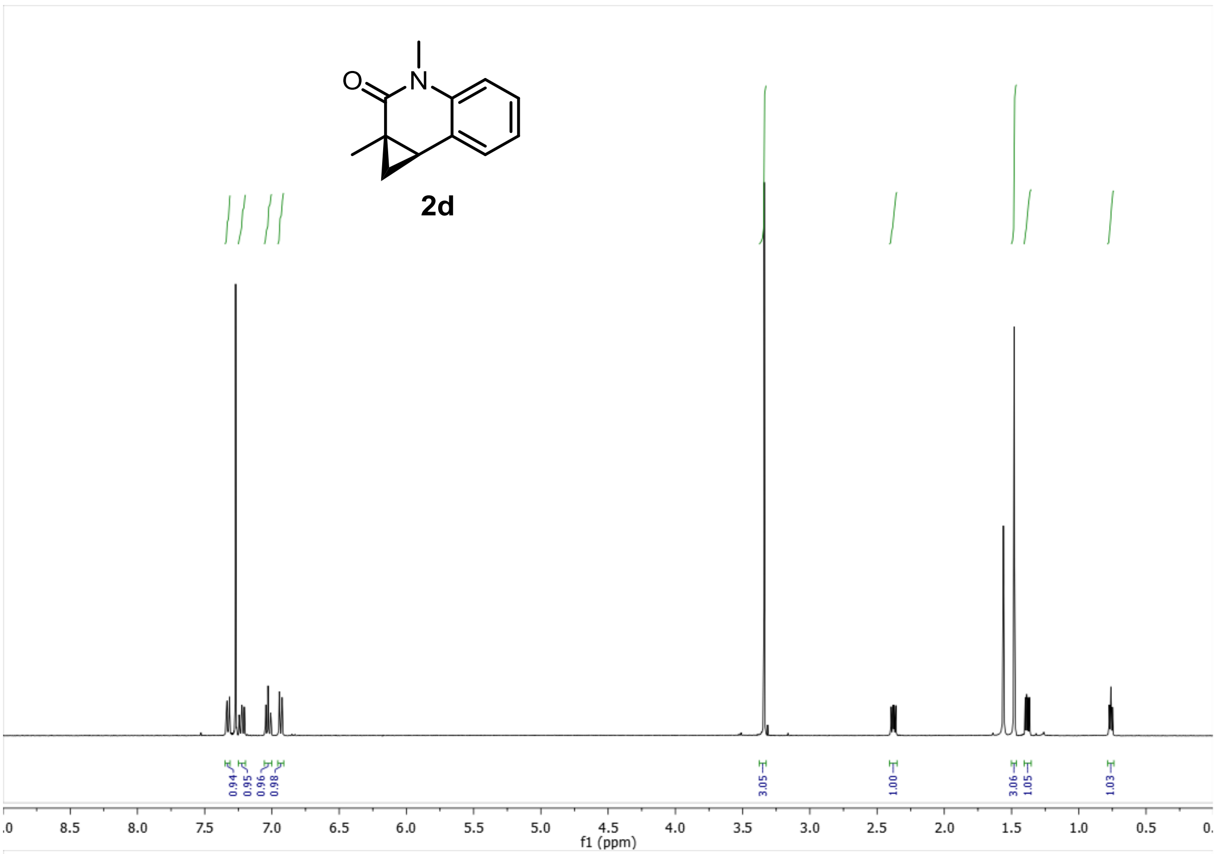
O
N
2d
0.94
0.95
0.96
0.98
3.05
1.00
3.06
1.05
1.03
f1 (ppm)

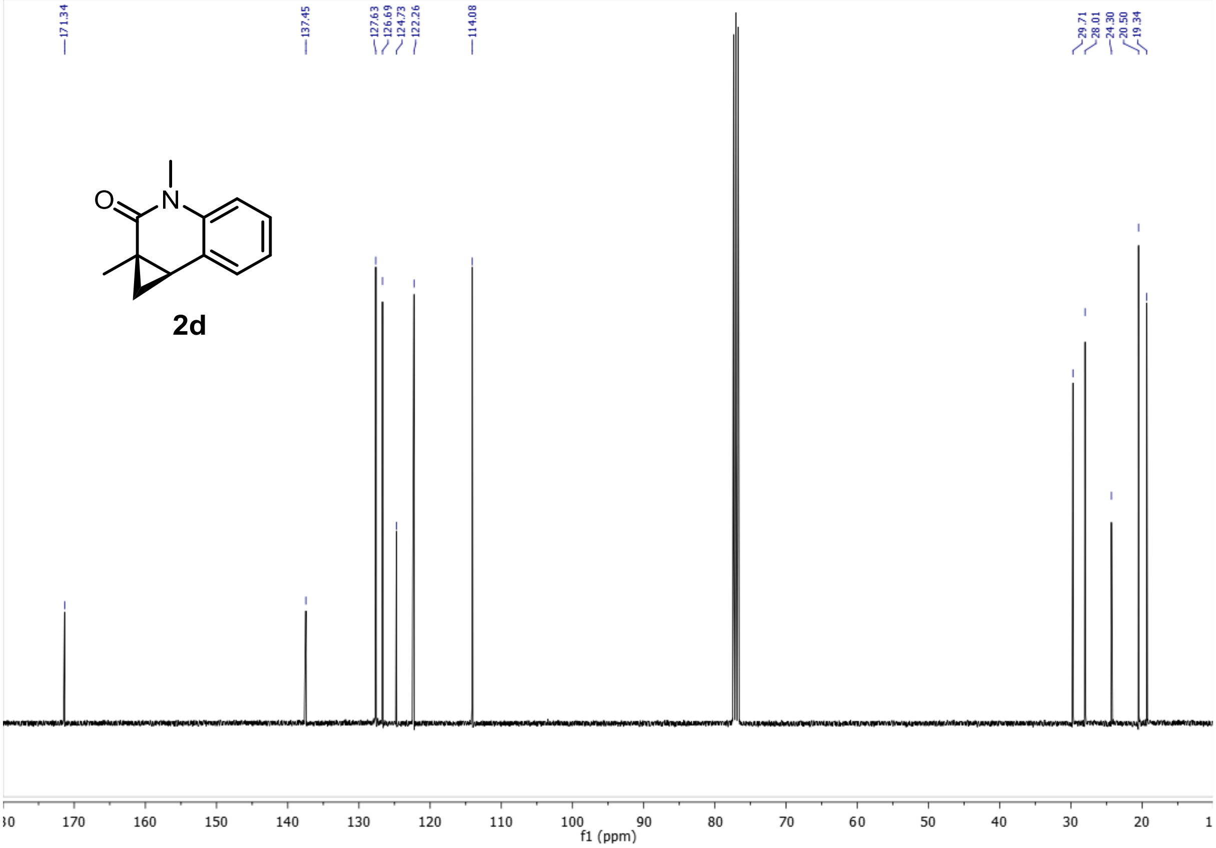
171.34
137.45
127.63
126.69
124.73
122.26
114.08
29.71
28.01
24.30
20.50
19.34
O
N
2d
f1 (ppm)

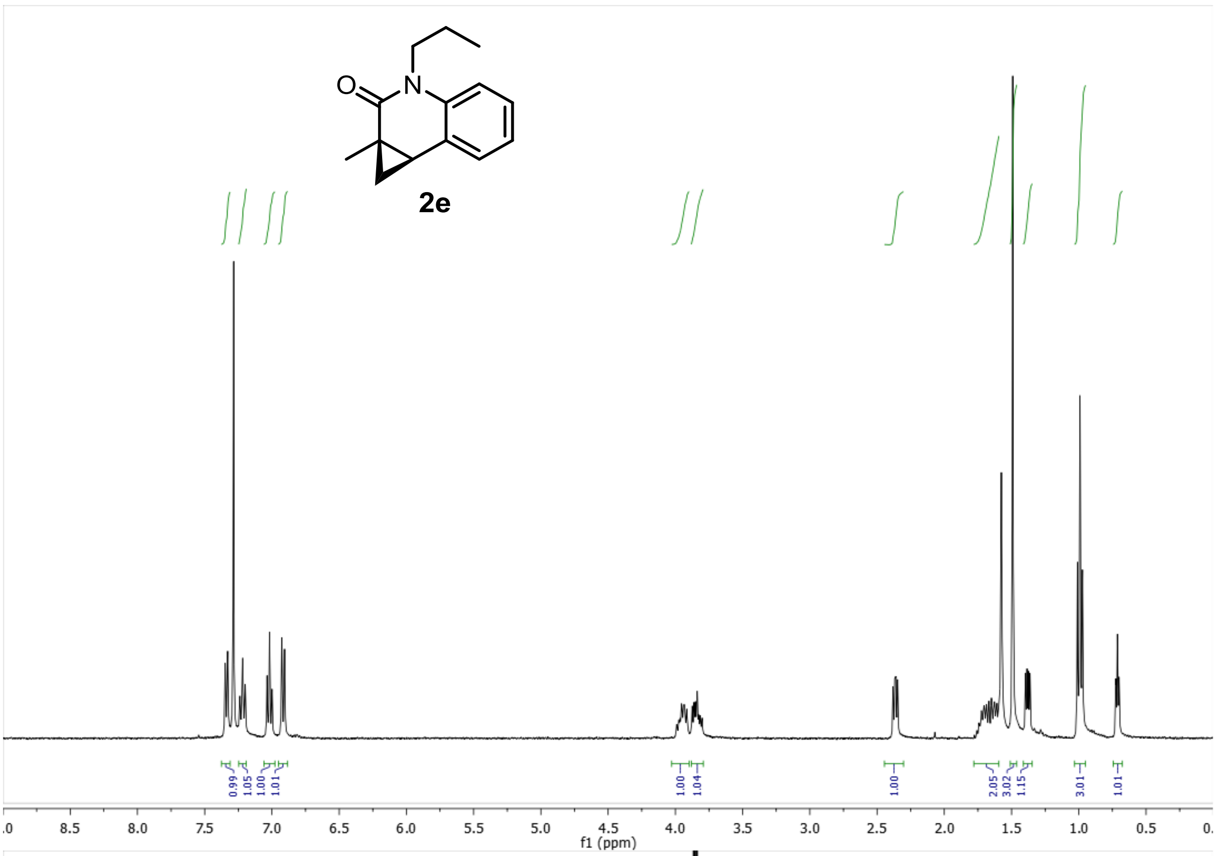
2e
f1 (ppm)

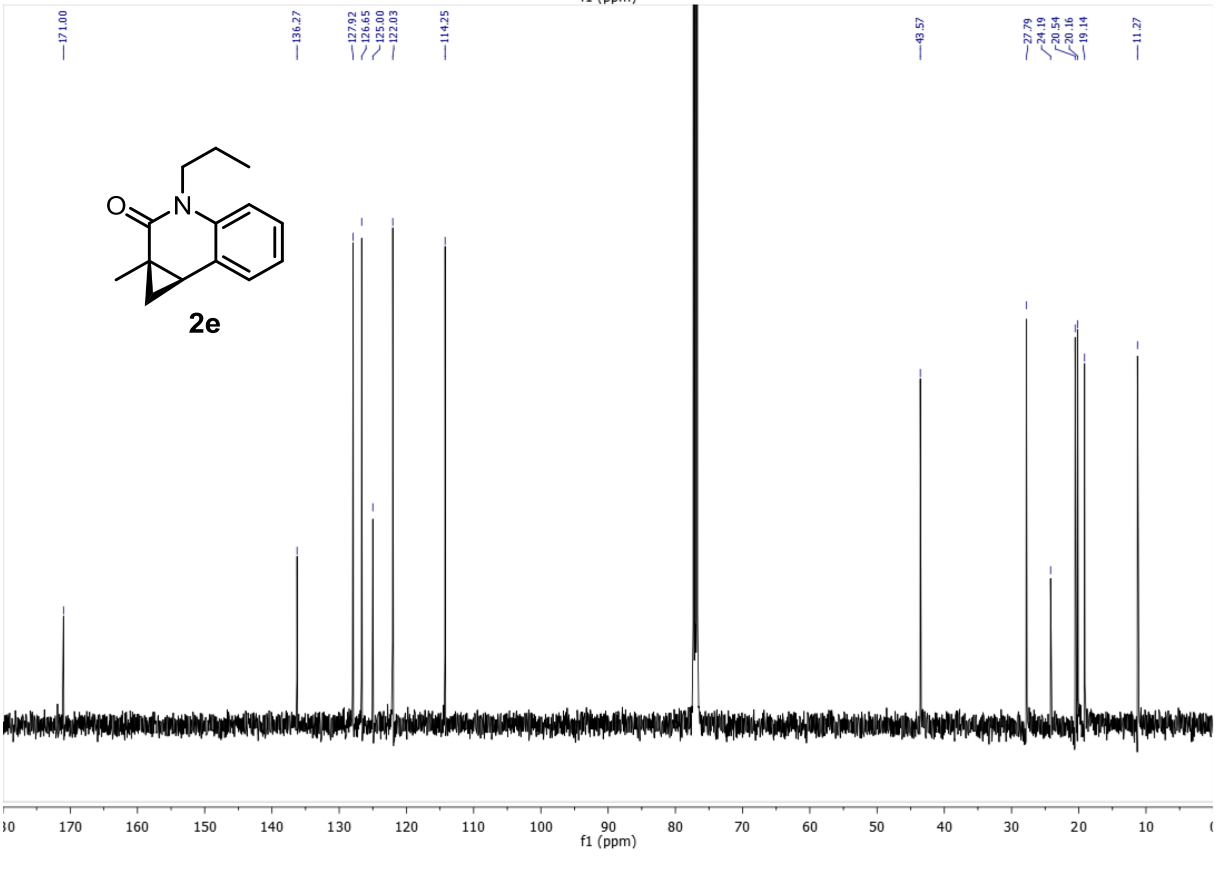
2e
f1 (ppm)

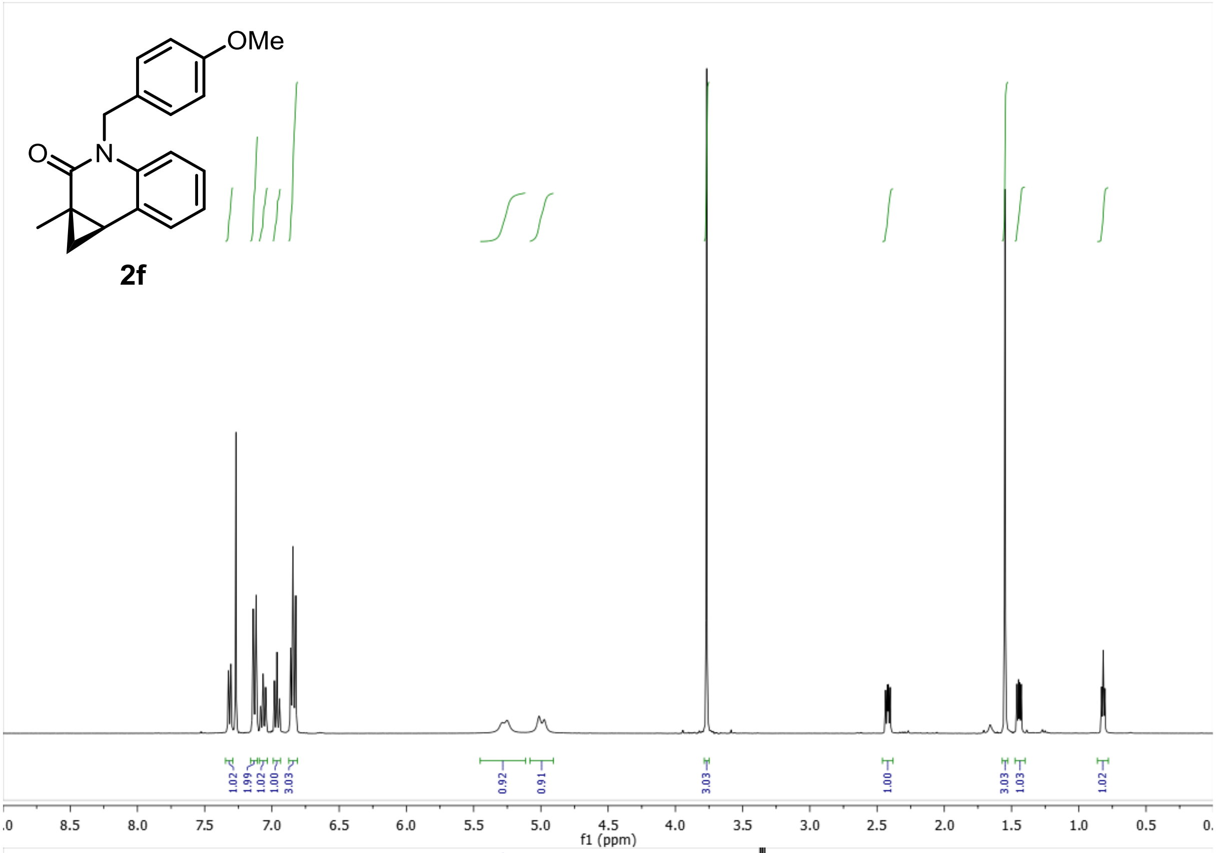
OMe
O
N
2f
1.02
1.99
1.02
1.00
3.03
0.92
0.91
3.03
1.00
3.03
1.03
1.02
f1 (ppm)

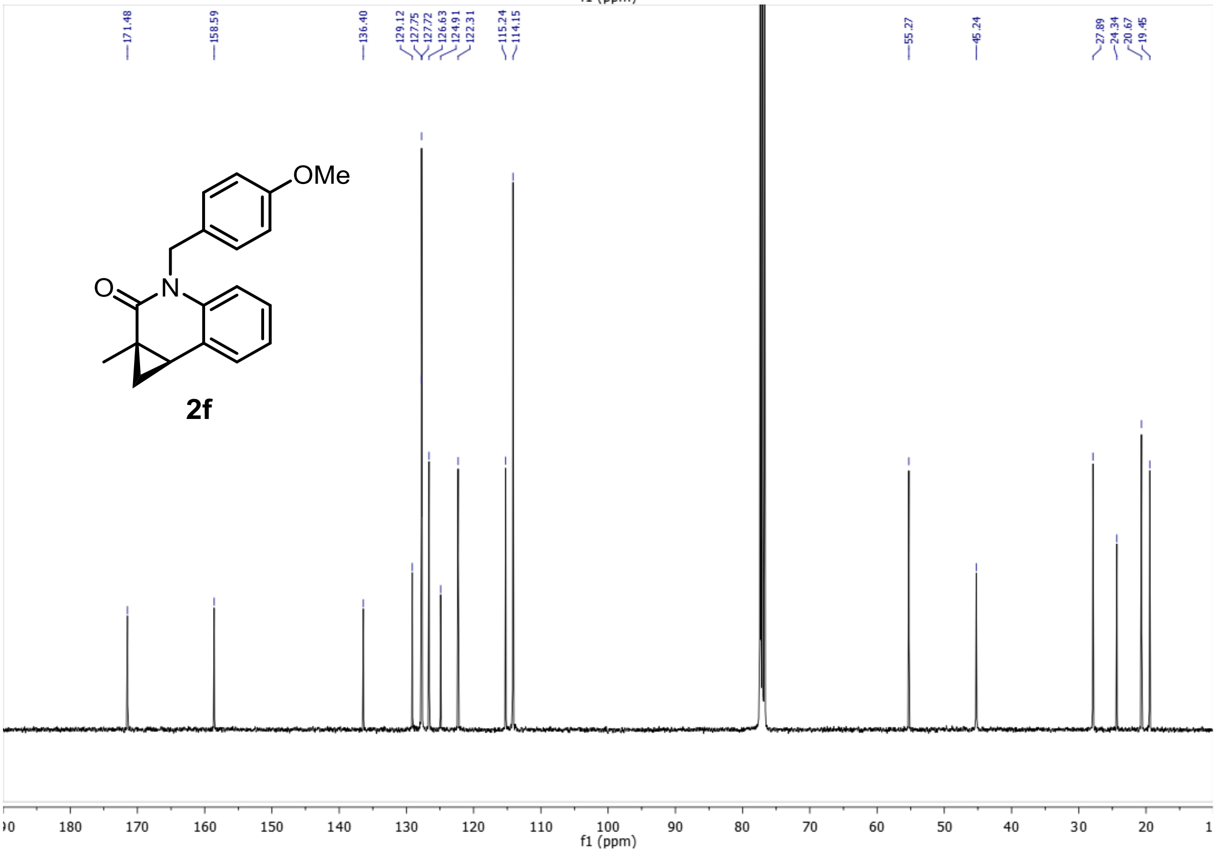
171.48
158.59
136.40
129.12
127.75
127.72
126.63
124.91
122.31
115.24
114.15
55.27
45.24
27.89
24.34
20.67
19.45
OMe
O
N
2f
f1 (ppm)

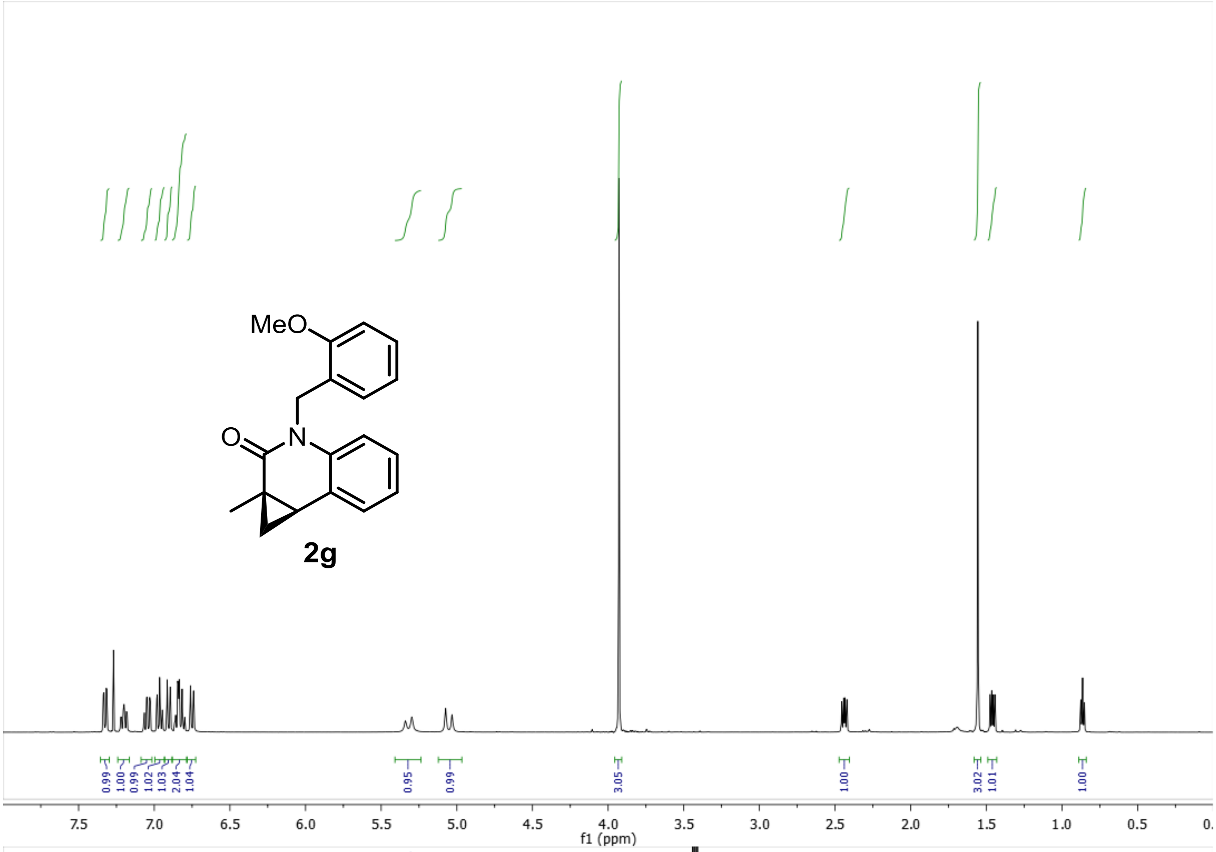
MeO
N
O
2g
0.99
1.00
0.99
1.02
1.03
2.04
1.04
0.95
0.99
3.05
1.00
3.02
1.01
1.00
7.5
7.0
6.5
6.0
5.5
5.0
4.5
4.0
3.5
3.0
2.5
2.0
1.5
1.0
0.5
f1 (ppm)

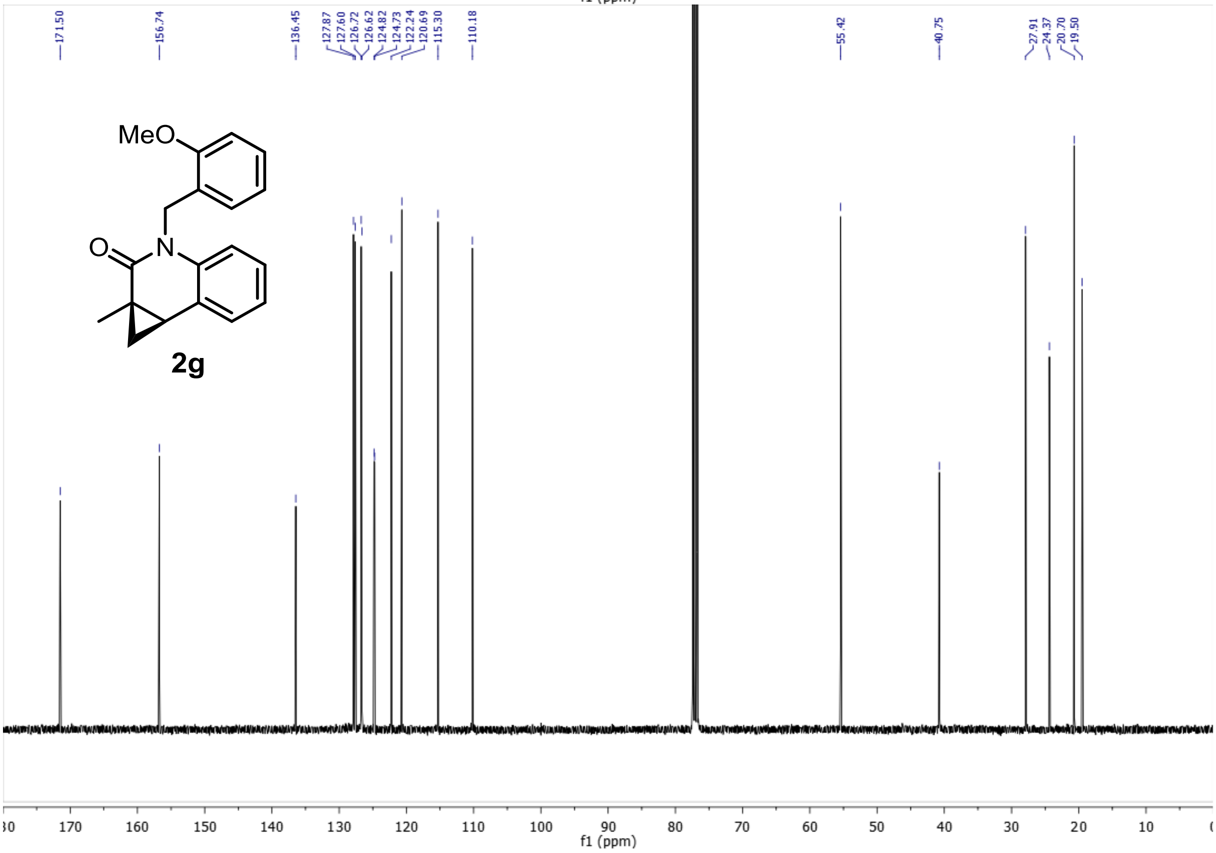
171.50
156.74
136.45
127.87
127.60
126.72
126.62
124.82
124.73
122.24
120.69
115.30
110.18
55.42
40.75
27.91
24.37
20.70
19.50
MeO
N
O
2g
170
160
150
140
130
120
110
100
90
80
70
60
50
40
30
20
10
f1 (ppm)

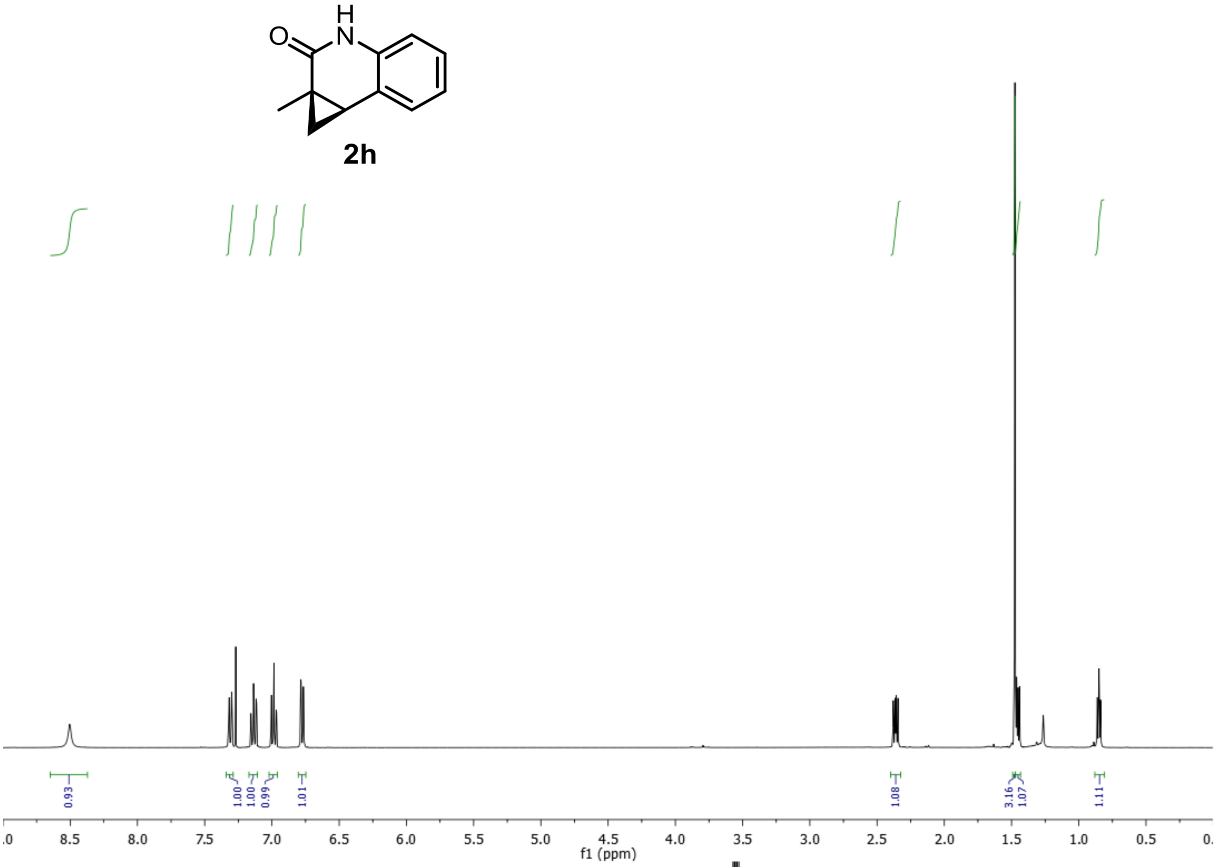
2h
0.93
1.00
1.00
0.99
1.01
1.08
3.16
1.07
1.11
8.5 8.0 7.5 7.0 6.5 6.0 5.5 5.0 4.5 4.0 3.5 3.0 2.5 2.0 1.5 1.0 0.5
f1 (ppm)

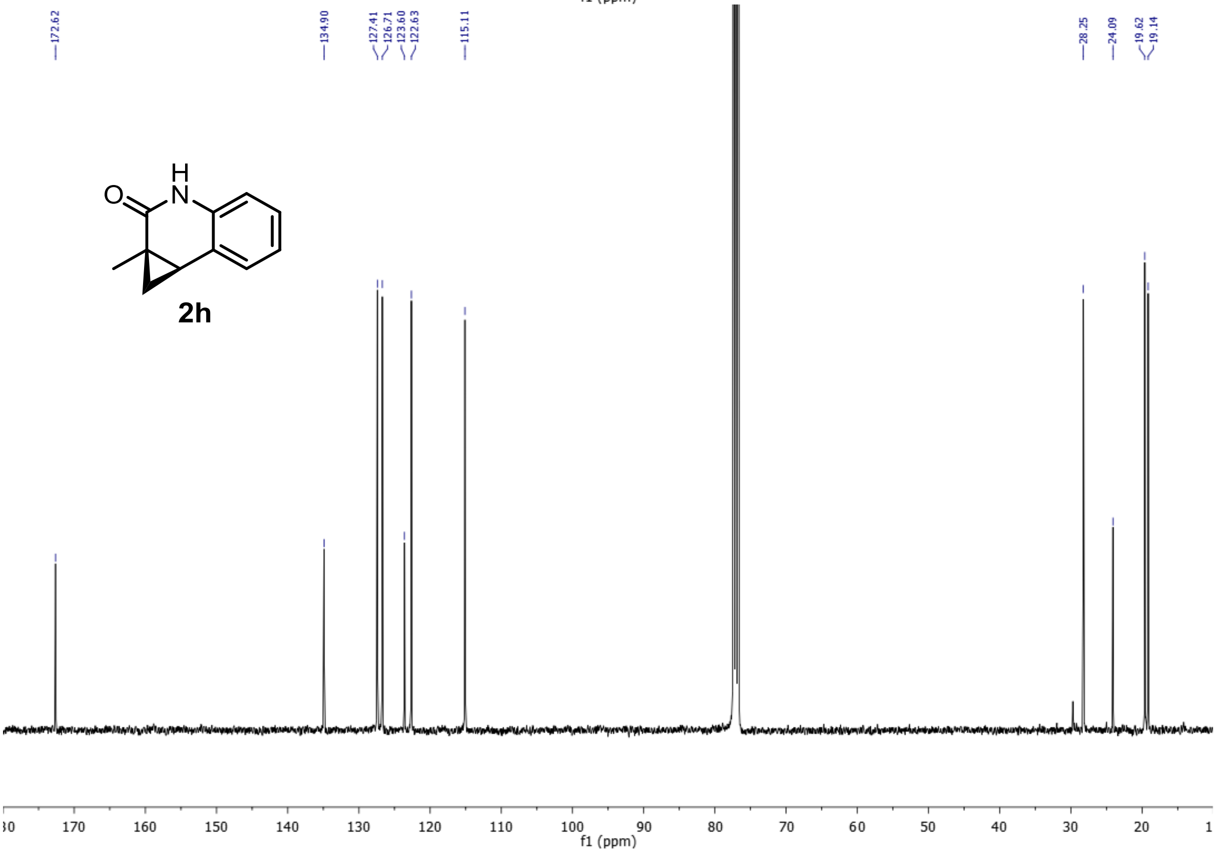
172.62
134.90
127.41
126.71
123.60
122.63
115.11
28.25
24.09
19.62
19.14
2h
170 160 150 140 130 120 110 100 90 80 70 60 50 40 30 20
f1 (ppm)

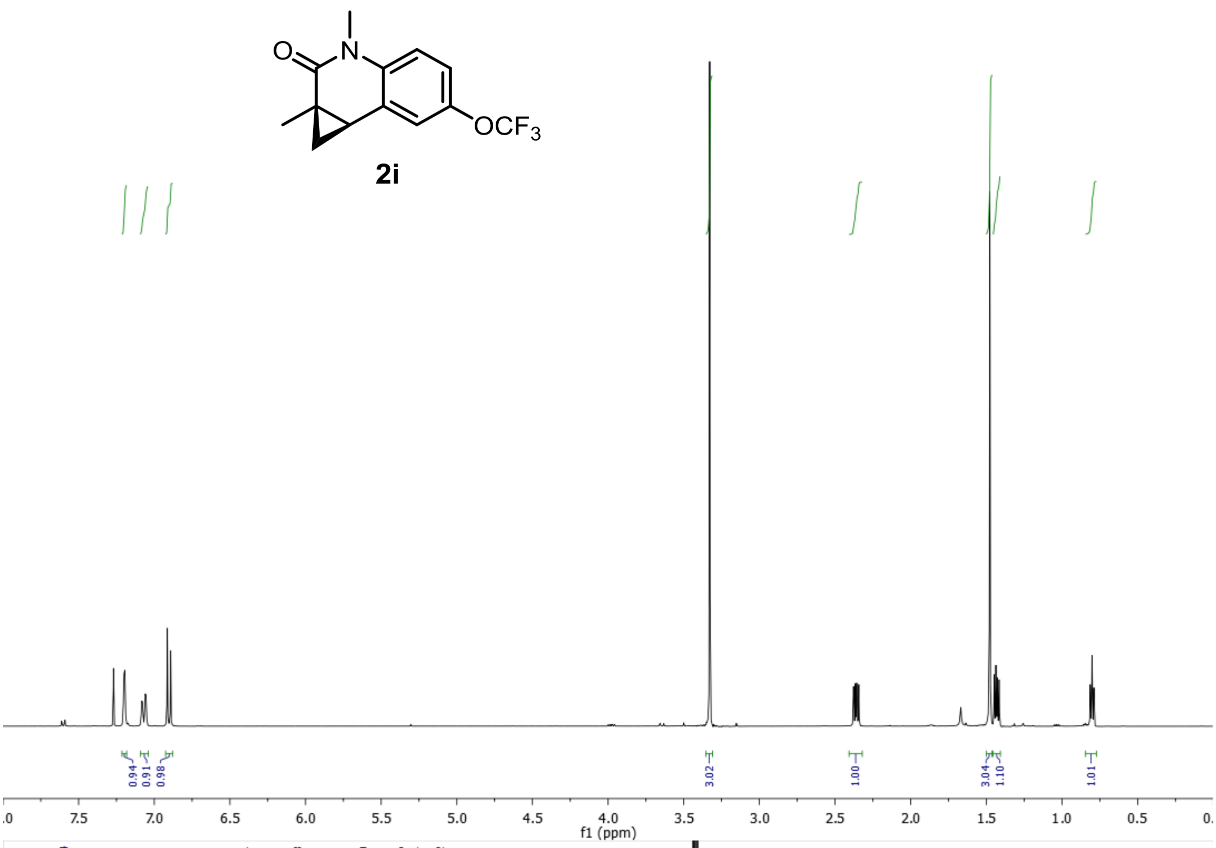
O
N
OCF3
2i
0.94
0.91
0.98
3.02
1.00
3.04
1.10
1.01
f1 (ppm)

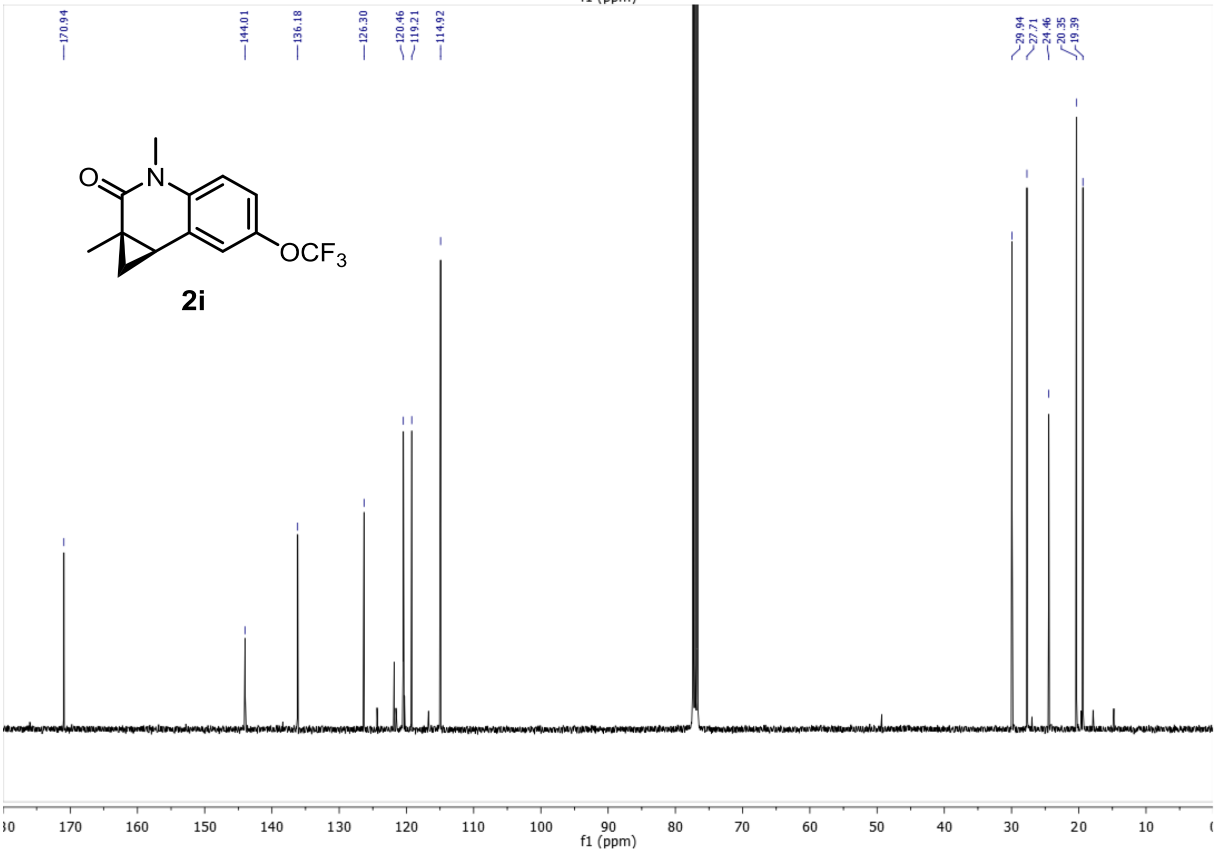
170.94
144.01
136.18
126.30
120.46
119.21
114.92
29.94
27.71
24.46
20.35
19.39
O
N
OCF3
2i
f1 (ppm)

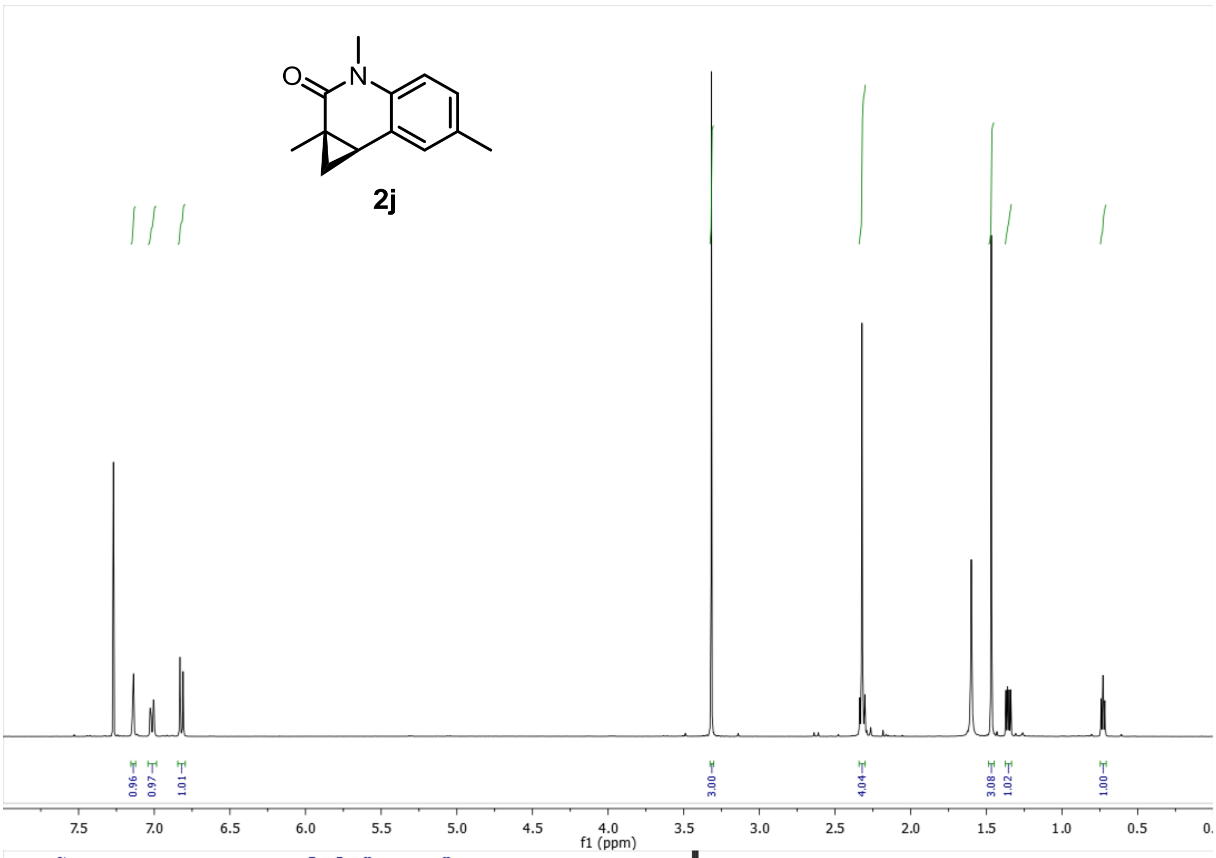
O
N
2j
0.96
0.97
1.01
3.00
4.04
3.08
1.02
1.00
f1 (ppm)

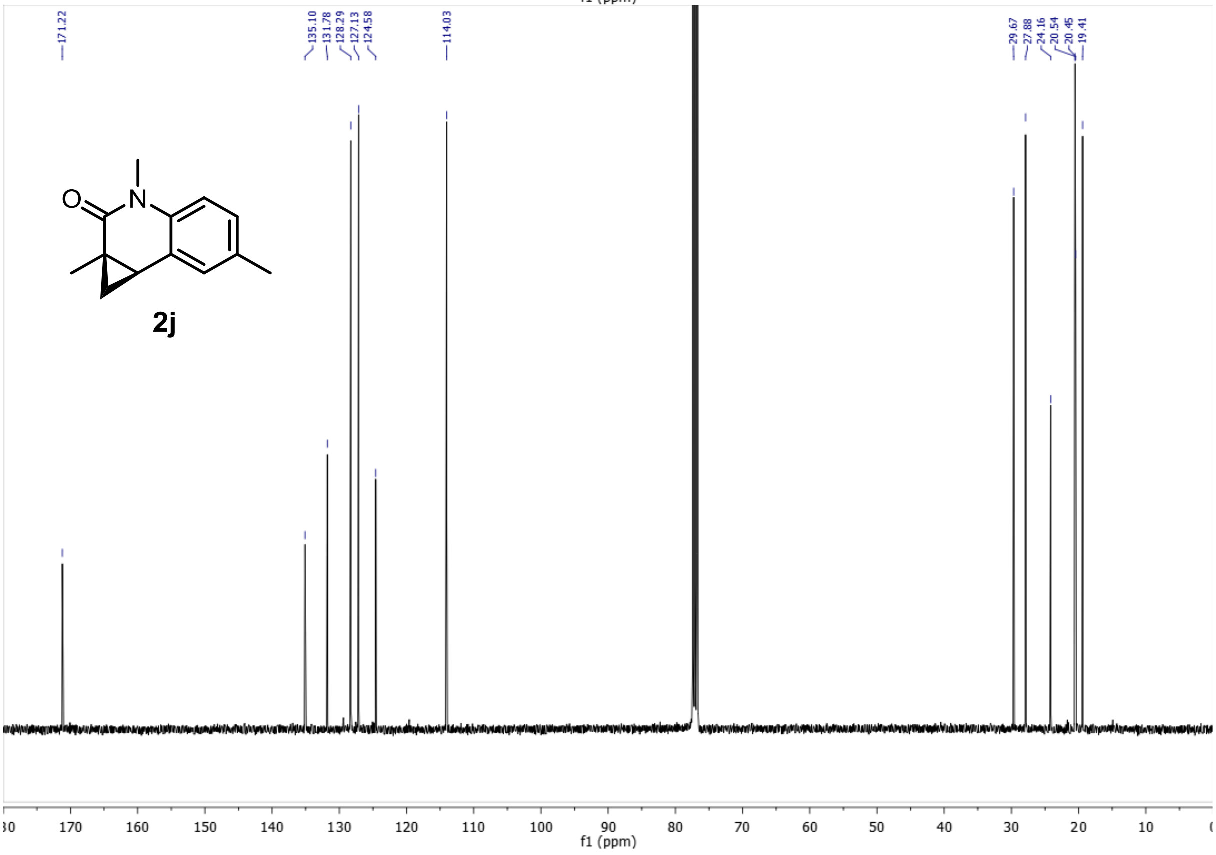
171.22
135.10
131.78
128.29
127.13
124.58
114.03
29.67
27.88
24.16
20.54
20.45
19.41
O
N
2j
f1 (ppm)

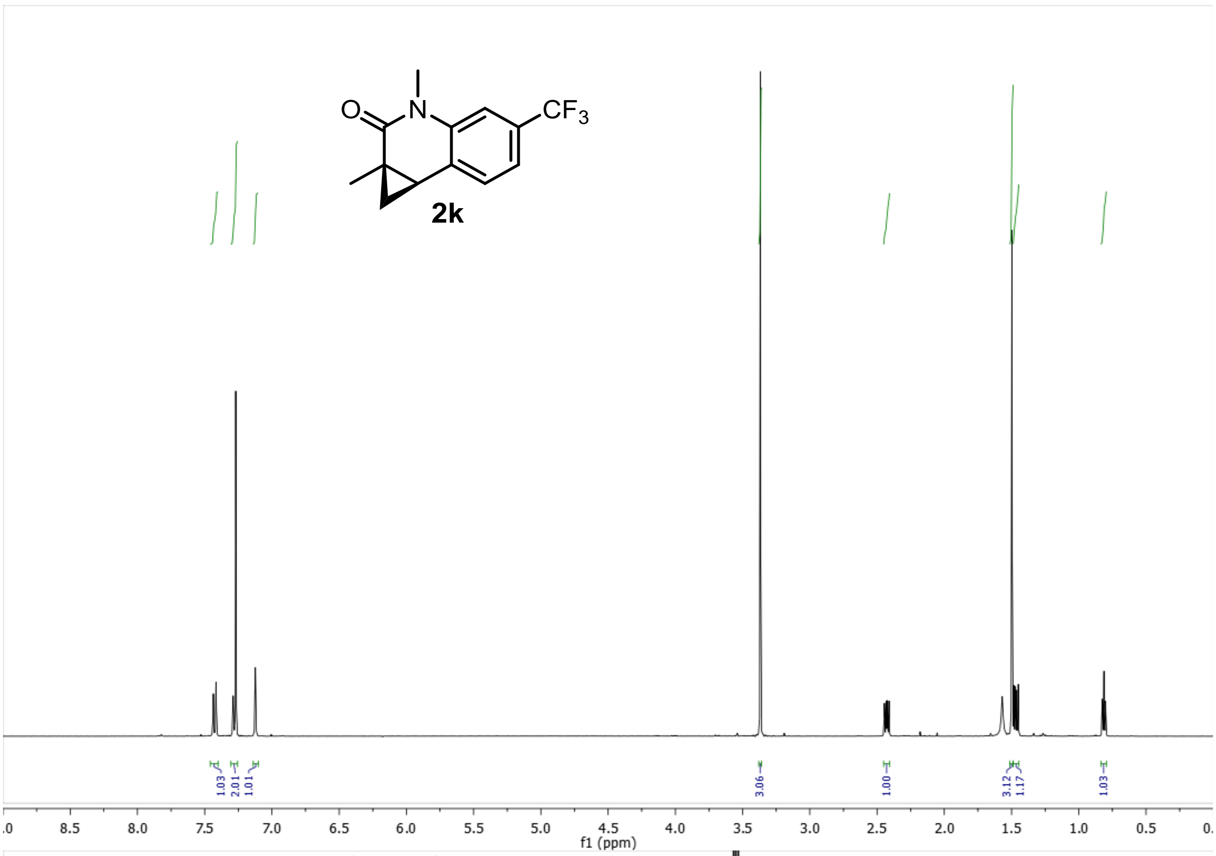
CF3
2k
f1 (ppm)

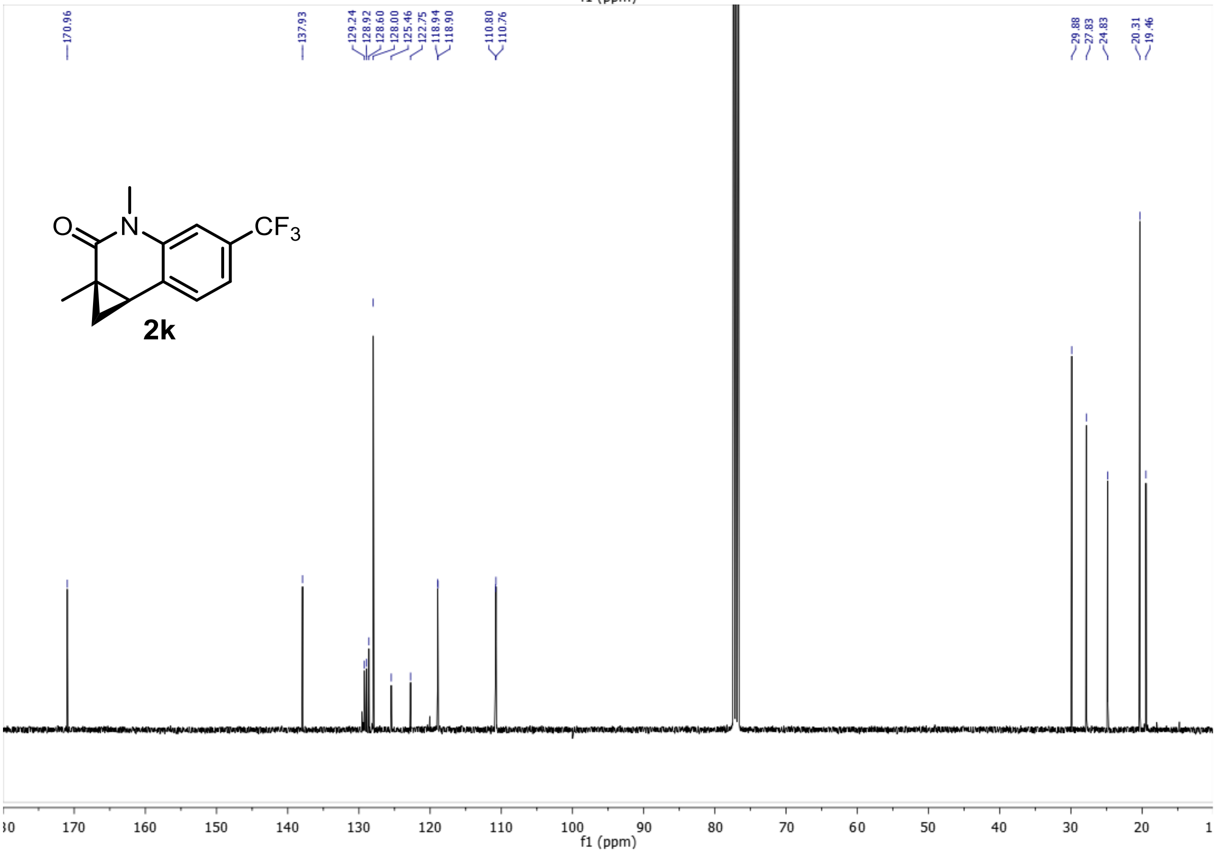
CF3
2k
f1 (ppm)

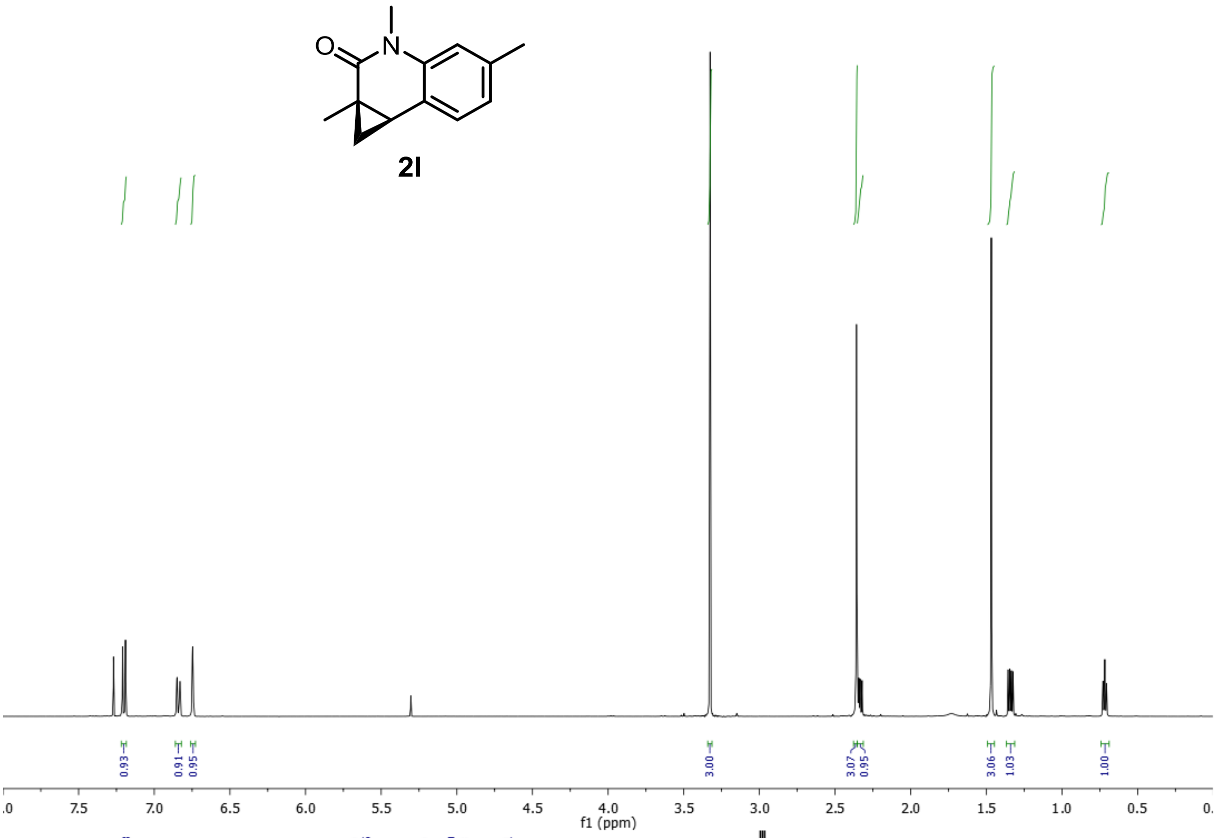
O
N
2l
0.93
0.91
0.95
3.00
3.07
0.95
3.06
1.03
1.00
f1 (ppm)

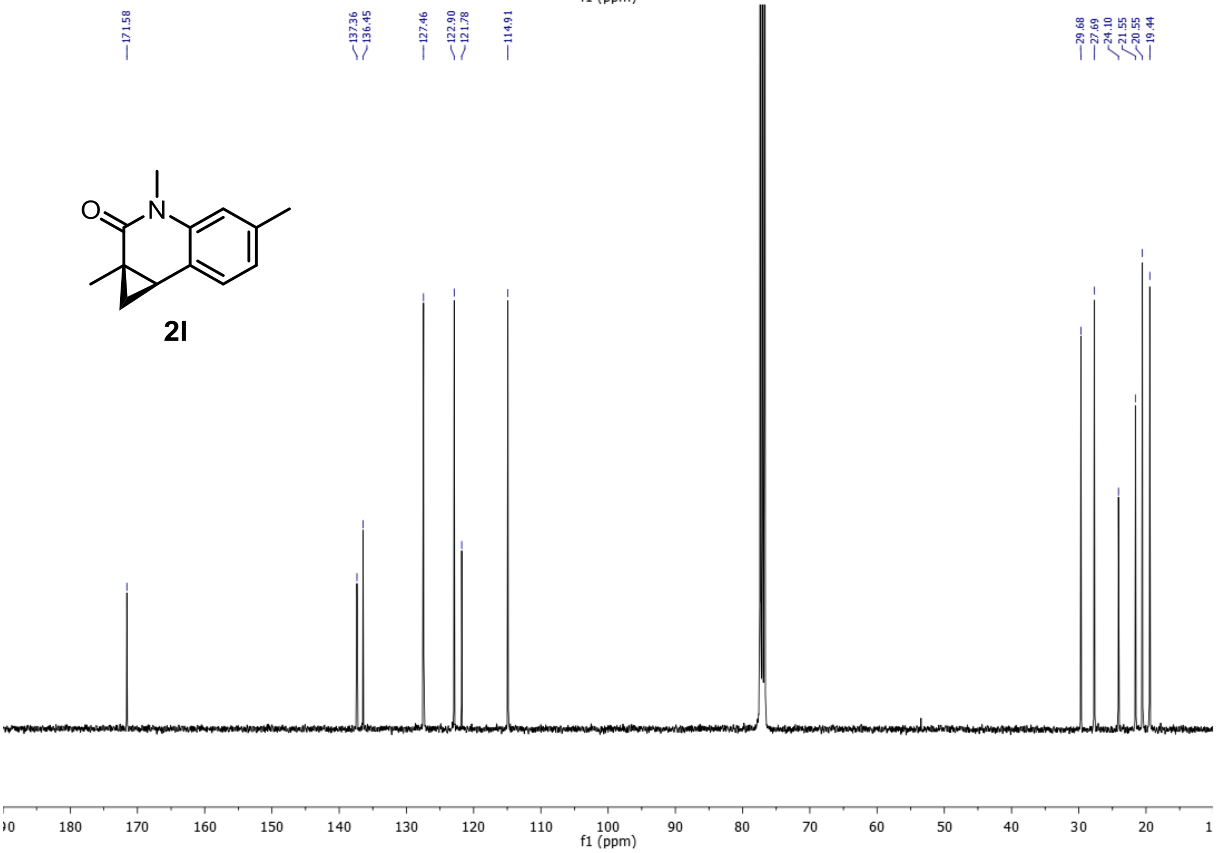
171.58
137.36
136.45
127.46
122.90
121.78
114.91
29.68
27.69
24.10
21.55
20.55
19.44
O
N
2l
f1 (ppm)

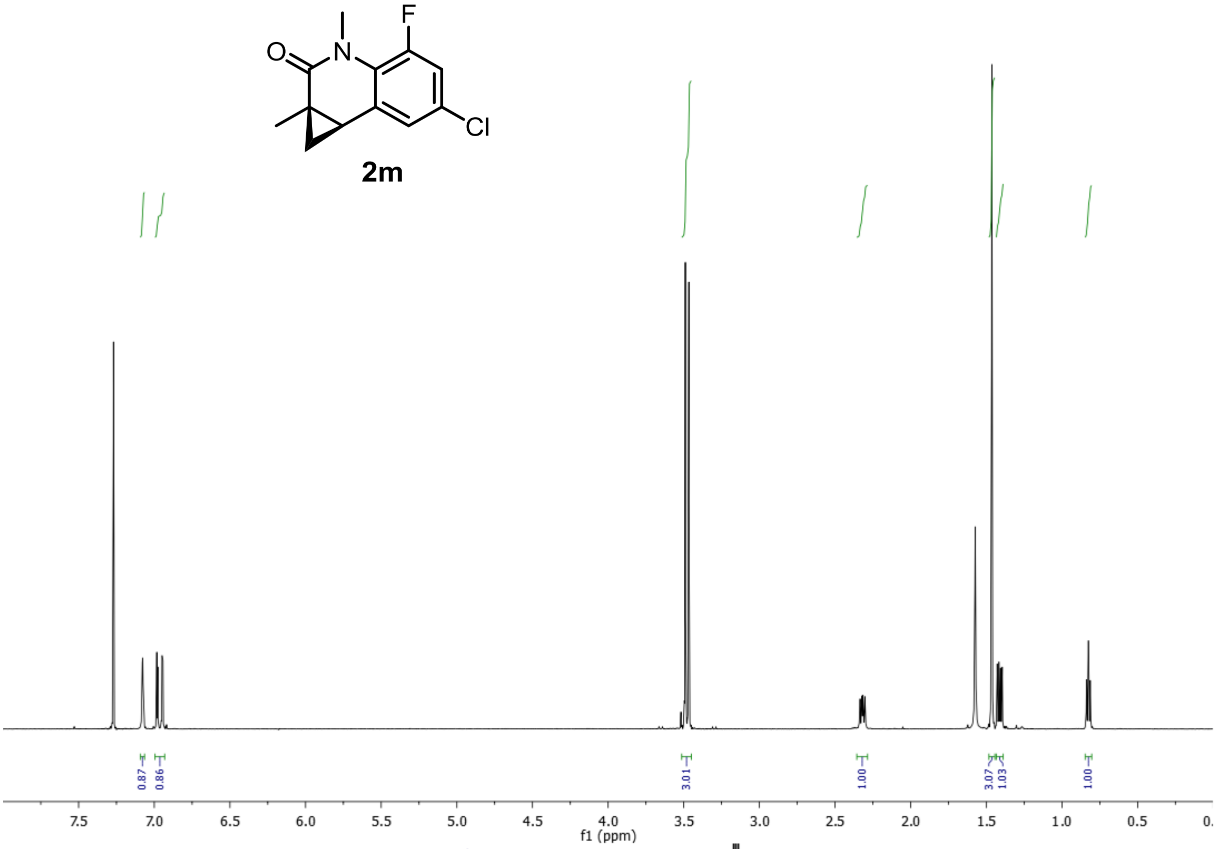
2m
0.87
0.86
3.01
1.00
3.07
1.03
1.00
7.5
7.0
6.5
6.0
5.5
5.0
4.5
4.0
3.5
3.0
2.5
2.0
1.5
1.0
0.5
f1 (ppm)

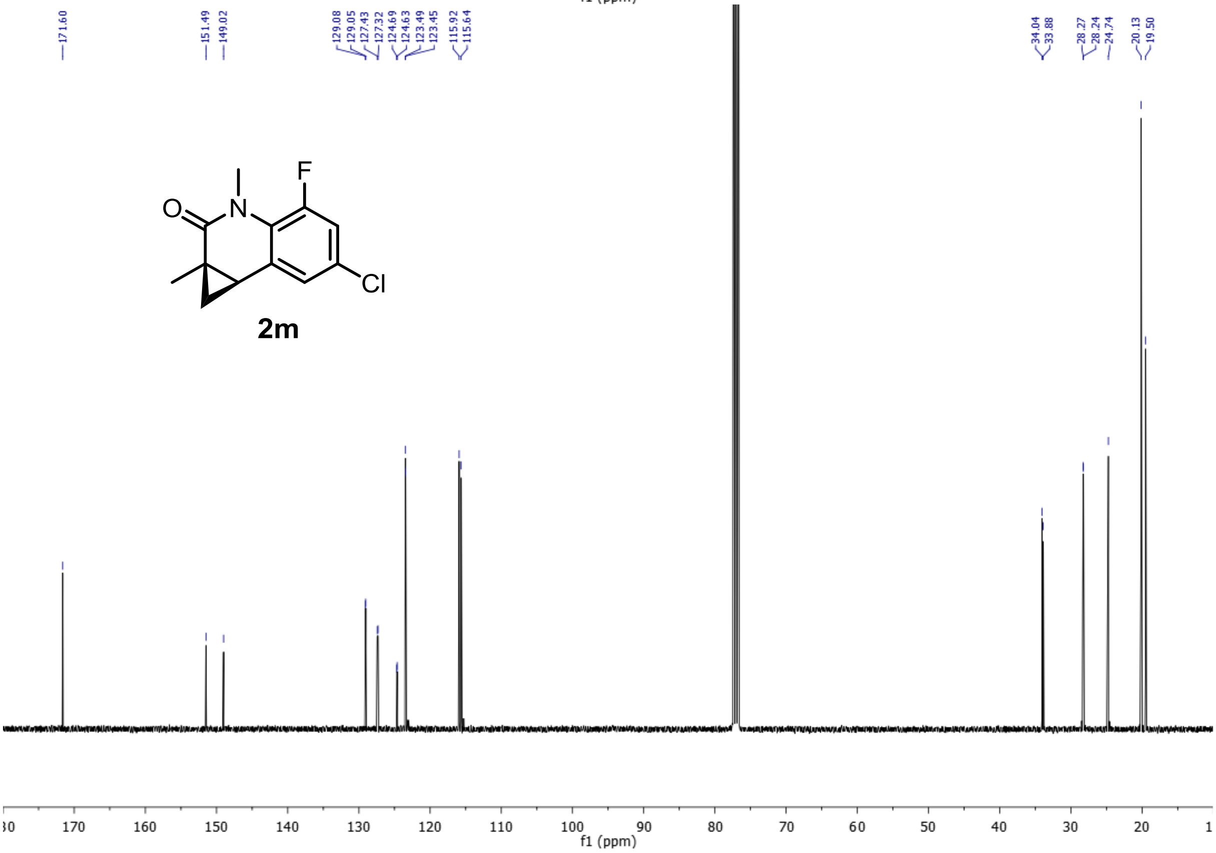
171.60
151.49
149.02
129.08
129.05
127.43
127.32
124.69
124.63
123.49
123.45
115.92
115.64
34.04
33.88
28.27
28.24
24.74
20.13
19.50
2m
170
160
150
140
130
120
110
100
90
80
70
60
50
40
30
20
f1 (ppm)

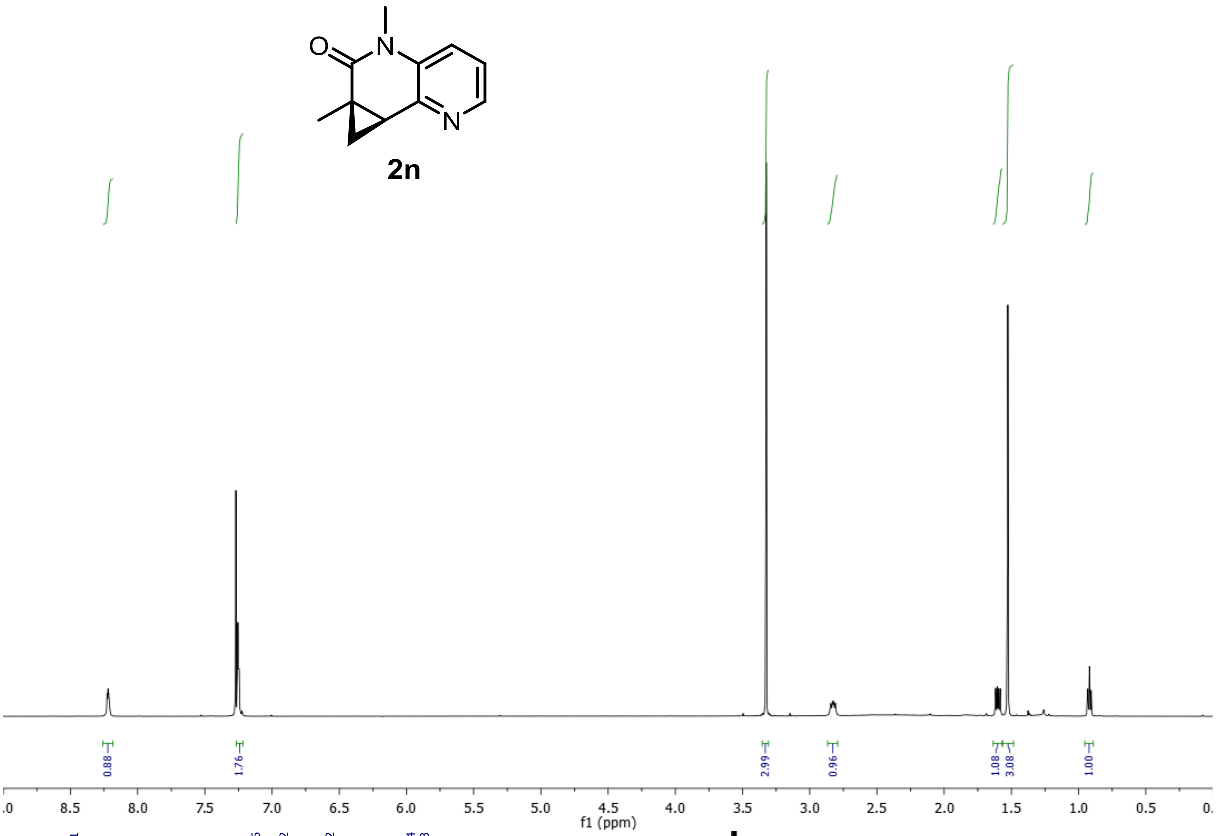
2n
0.88
1.76
2.99
0.96
1.08
3.08
1.00
f1 (ppm)

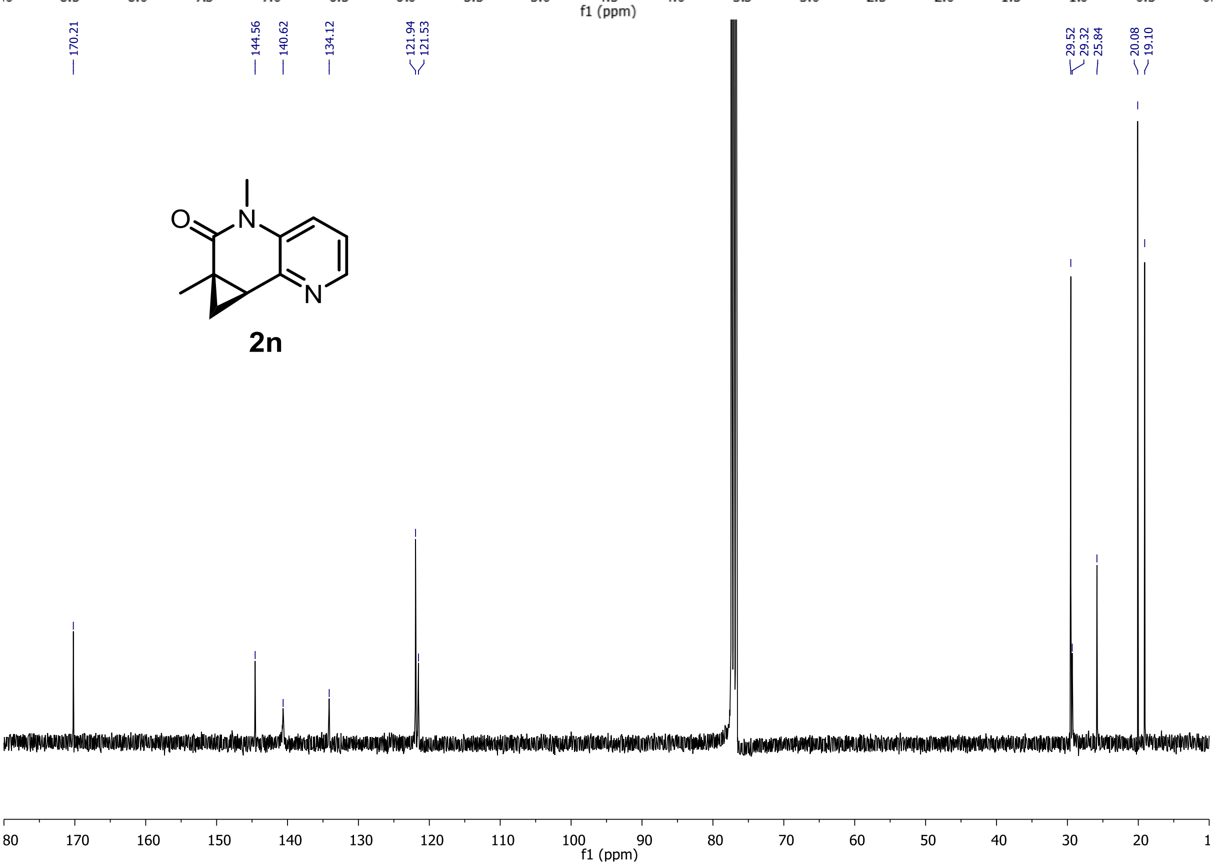
170.21
144.56
140.62
134.12
121.94
121.53
29.52
29.32
25.84
20.08
19.10
2n
f1 (ppm)

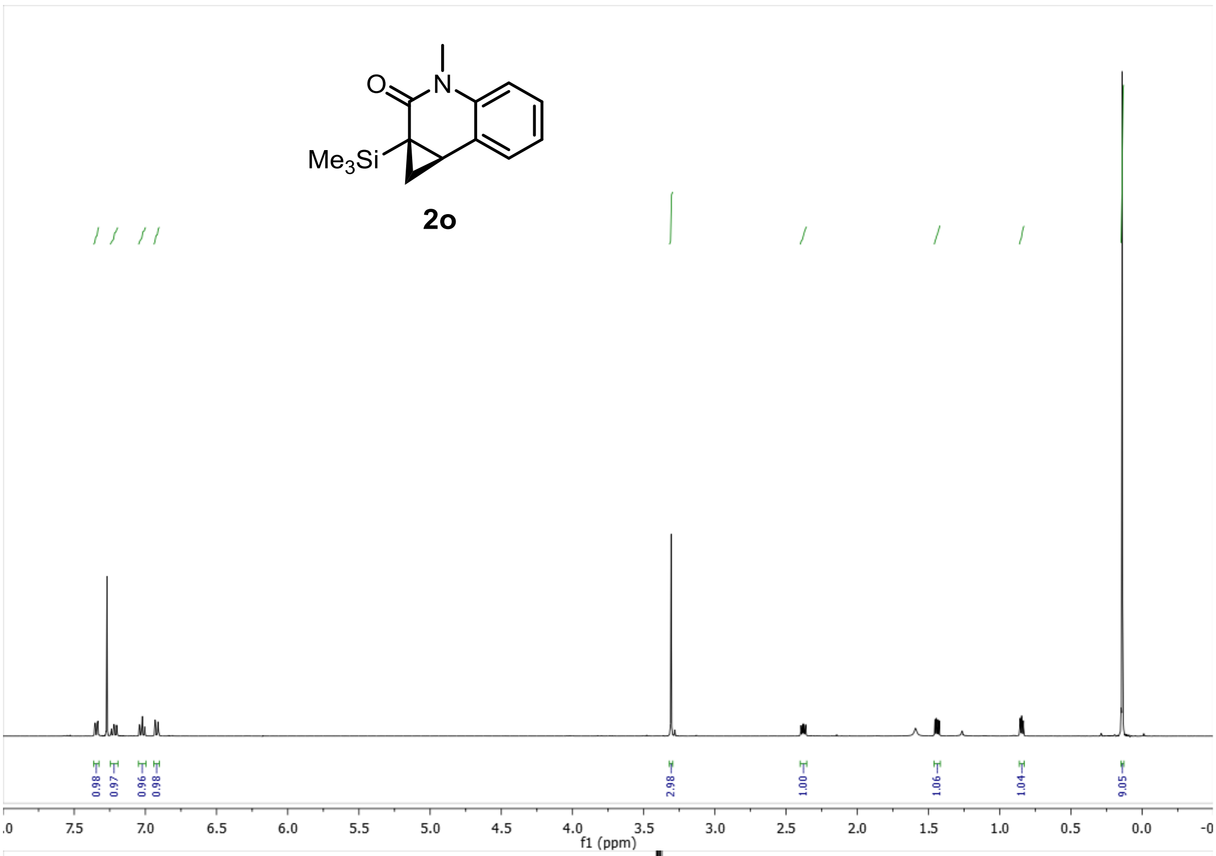

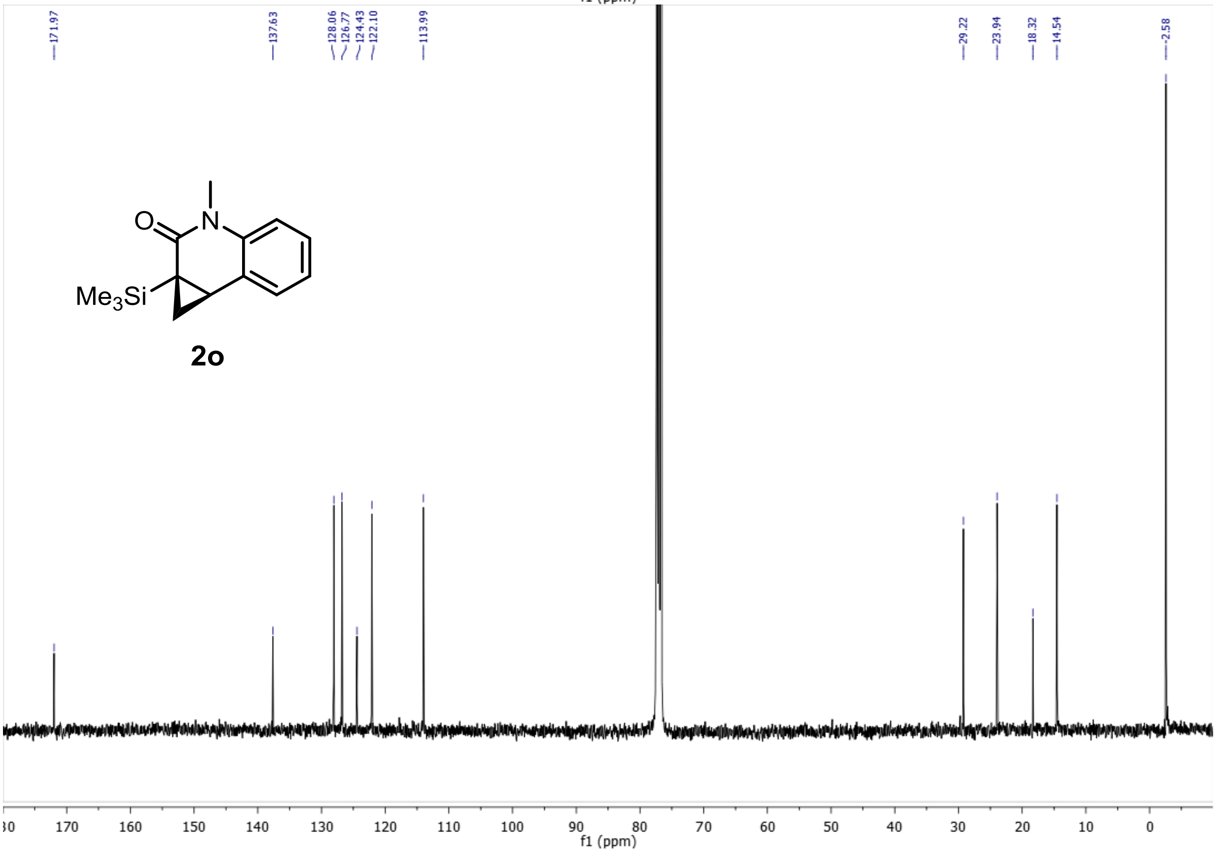

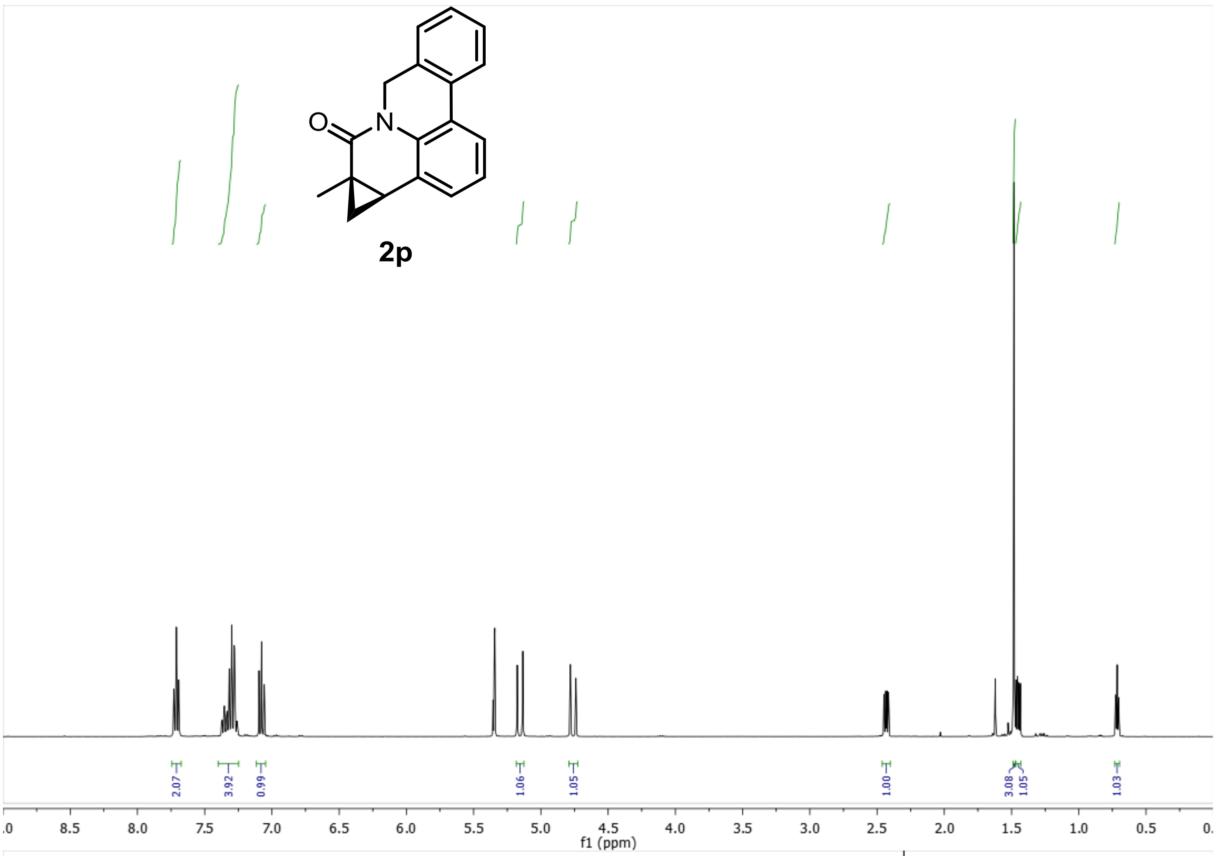

2p
2.07
3.92
0.99
1.06
1.05
1.00
3.08
1.05
1.03
f1 (ppm)

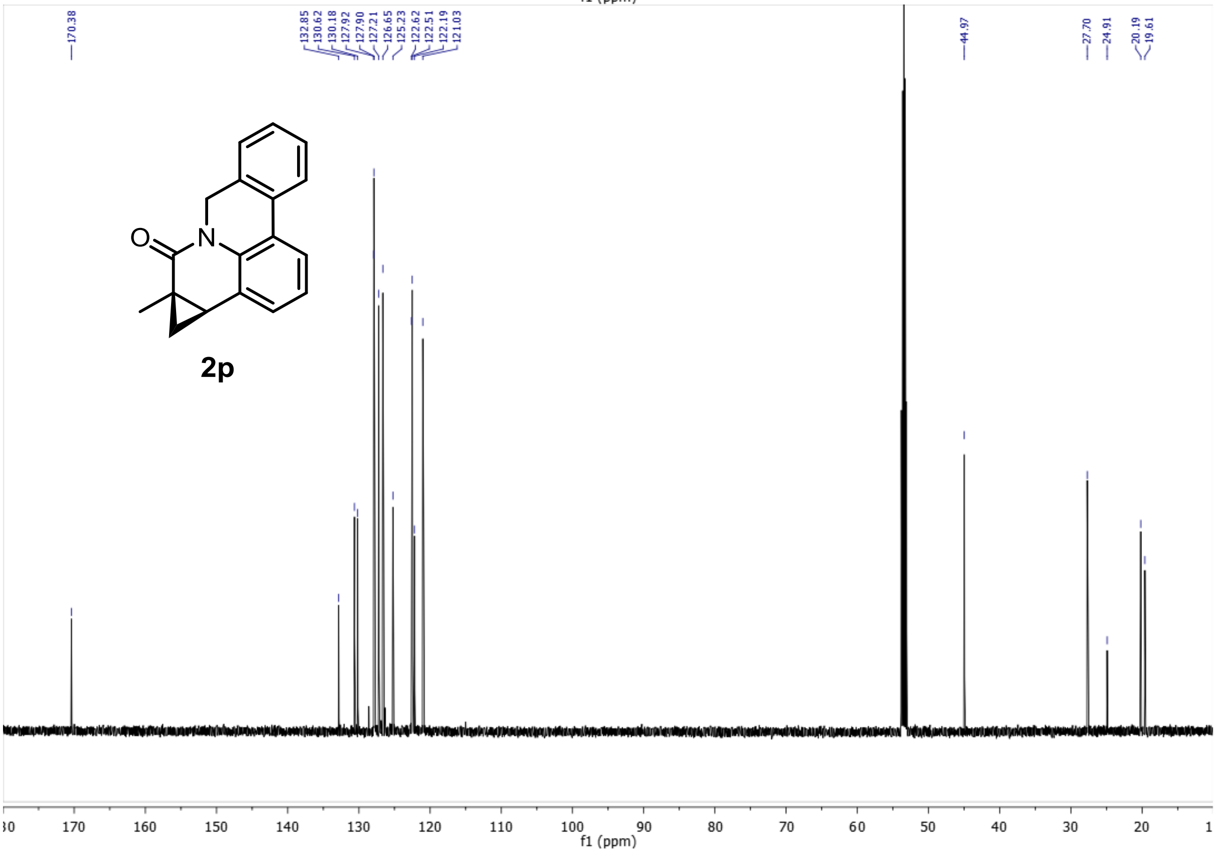

170.38
132.85
130.62
130.18
127.92
127.90
127.21
126.65
125.23
122.62
122.51
122.19
121.03
44.97
27.70
24.91
20.19
19.61
2p
f1 (ppm)

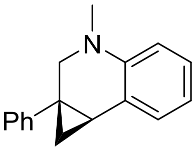

N
Ph

9

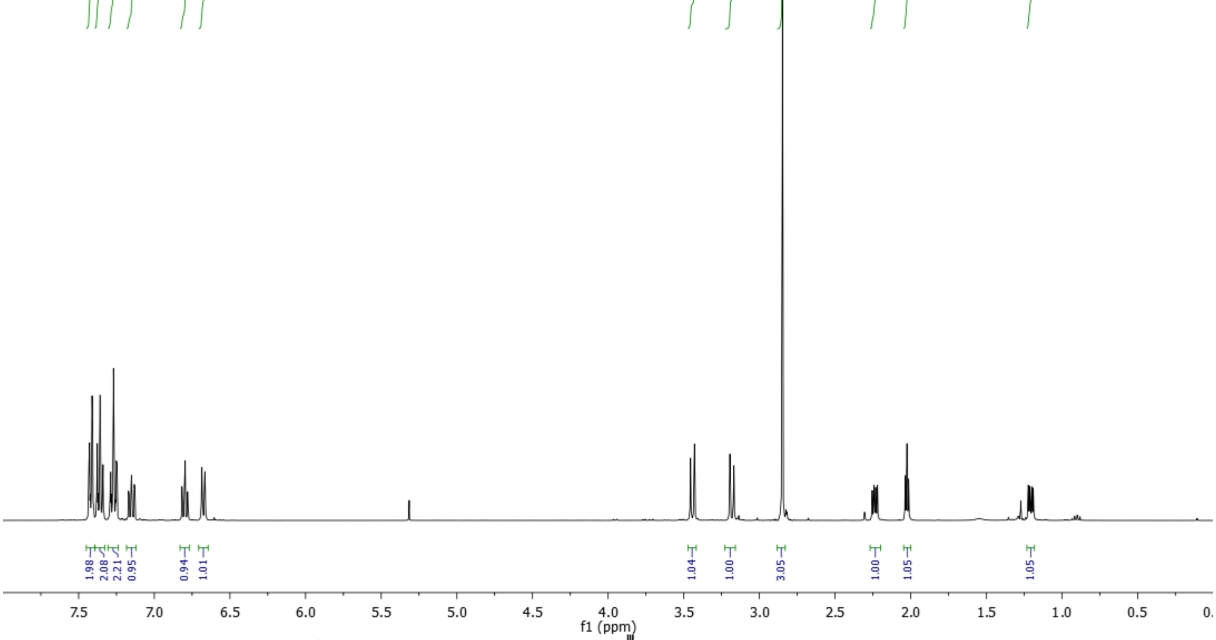

1.98
2.08
2.21
0.95
0.94
1.01
1.04
1.00
3.05
1.00
1.05
1.05
7.5 7.0 6.5 6.0 5.5 5.0 4.5 4.0 3.5 3.0 2.5 2.0 1.5 1.0 0.5
f1 (ppm)

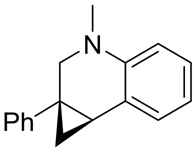

N
Ph

9

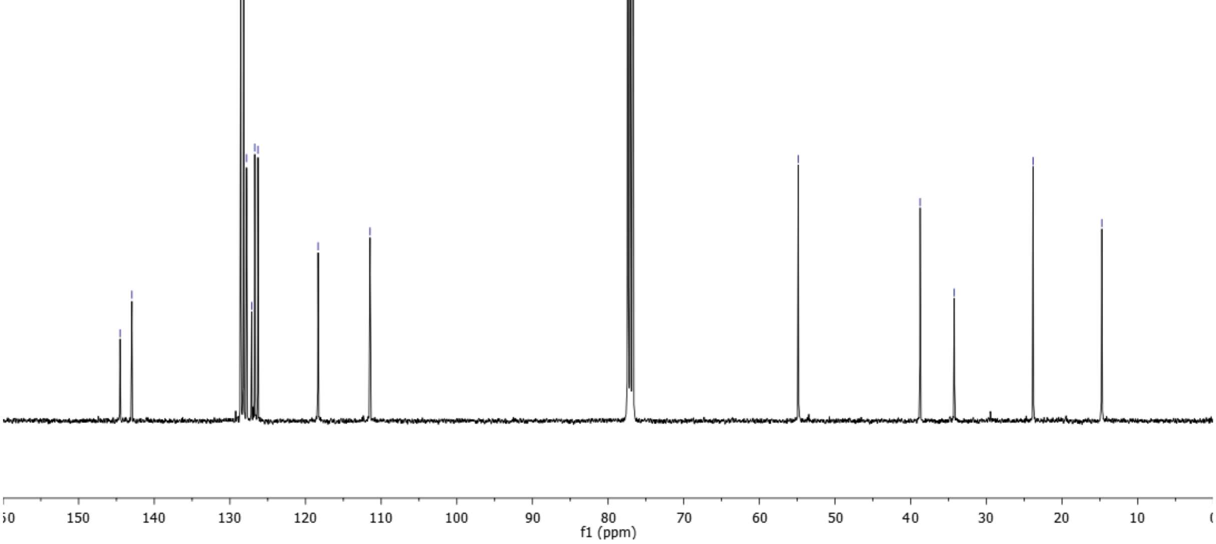

144.52
142.96
128.55
128.18
127.80
127.13
126.72
126.29
118.34
111.47
54.86
38.75
34.24
23.82
14.74
150 140 130 120 110 100 90 80 70 60 50 40 30 20 10
f1 (ppm)

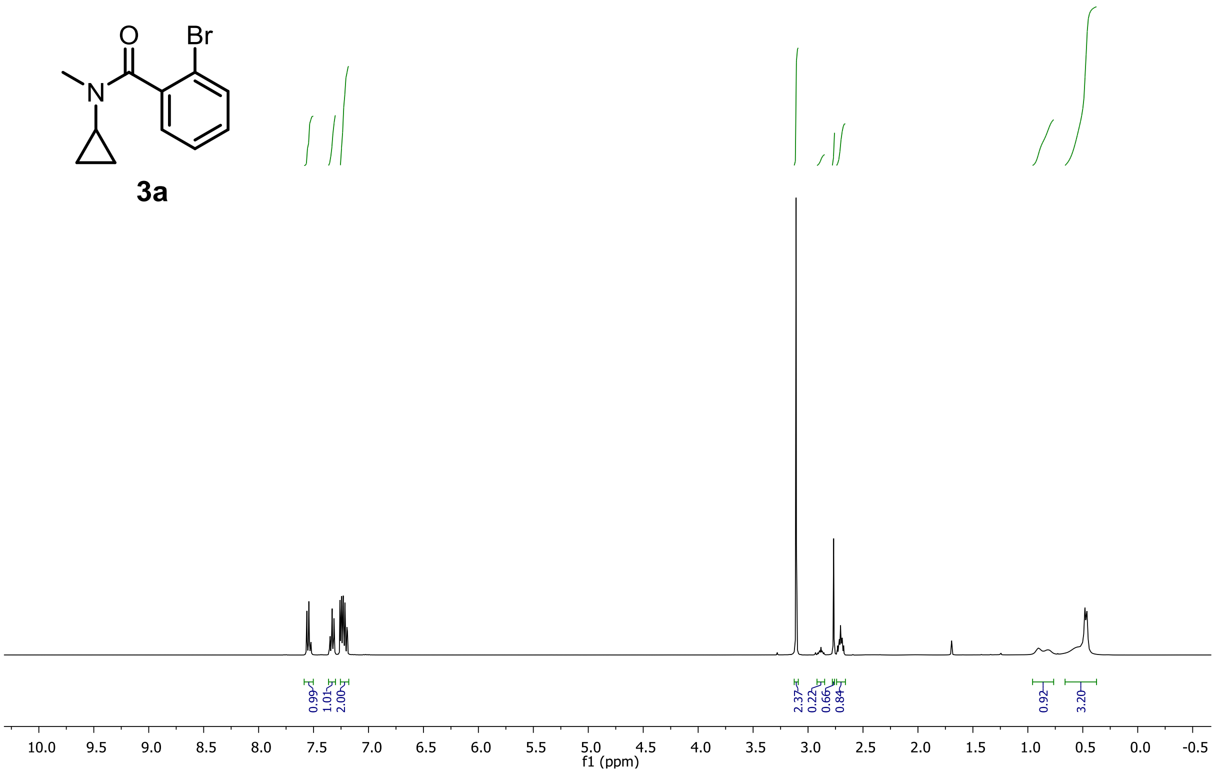
Br
O
N
3a
0.99
1.01
2.00
2.37
0.22
0.66
0.84
0.92
3.20
10.0
9.5
9.0
8.5
8.0
7.5
7.0
6.5
6.0
5.5
5.0
4.5
4.0
3.5
3.0
2.5
2.0
1.5
1.0
0.5
0.0
-0.5
f1 (ppm)

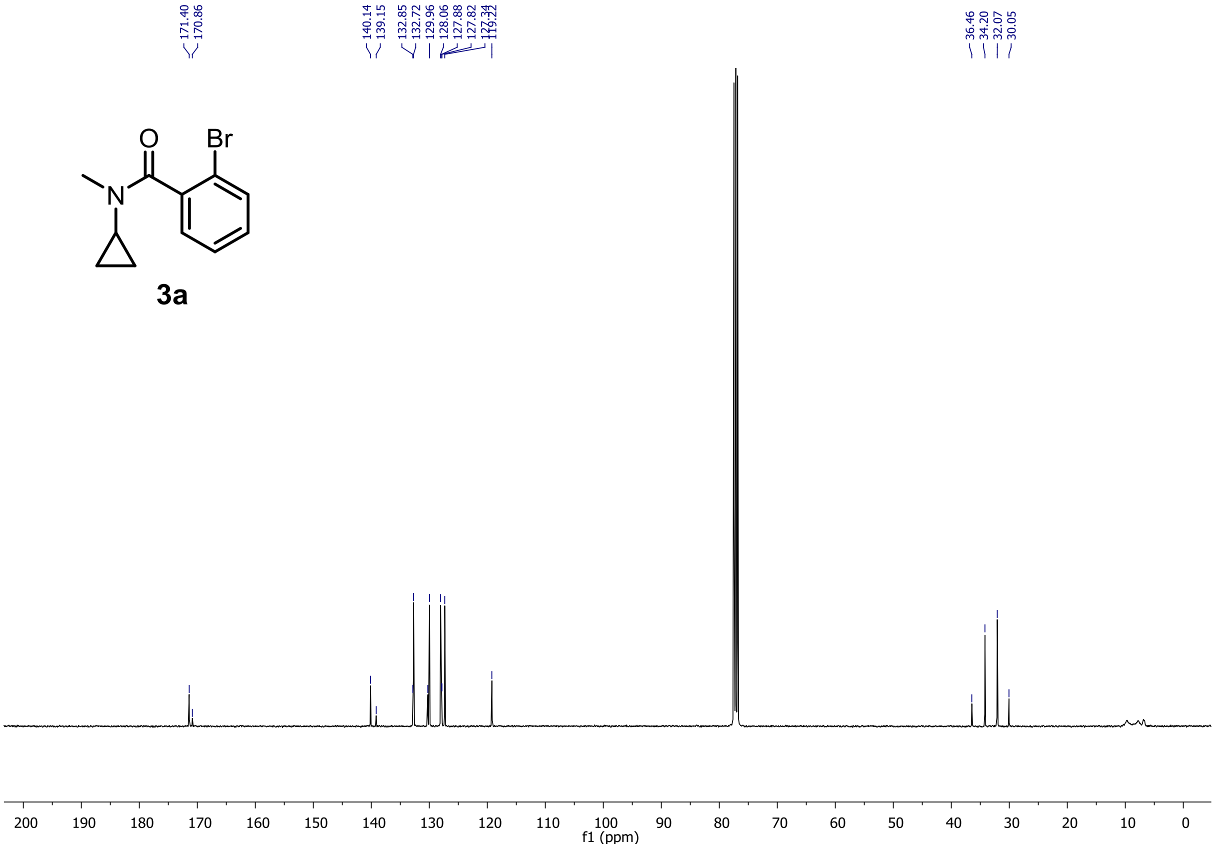
171.40
170.86
140.14
139.15
132.85
132.72
129.96
128.06
127.88
127.82
127.34
36.46
34.20
32.07
30.05
Br
O
N
3a
200
190
180
170
160
150
140
130
120
110
100
90
80
70
60
50
40
30
20
10
0
f1 (ppm)

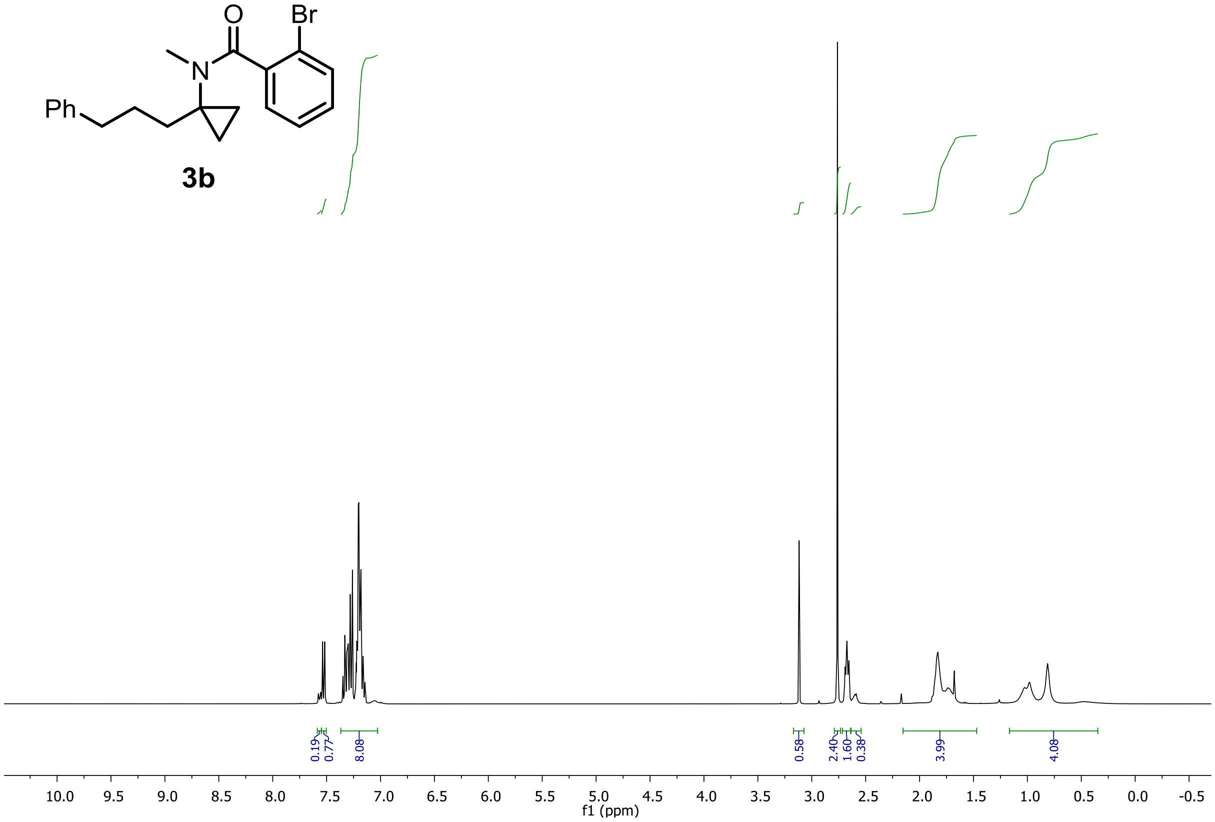
O
Br
N
Ph
3b
0.19
0.77
8.08
0.58
2.40
1.60
0.38
3.99
4.08
10.0 9.5 9.0 8.5 8.0 7.5 7.0 6.5 6.0 5.5 5.0 4.5 4.0 3.5 3.0 2.5 2.0 1.5 1.0 0.5 0.0 -0.5
f1 (ppm)

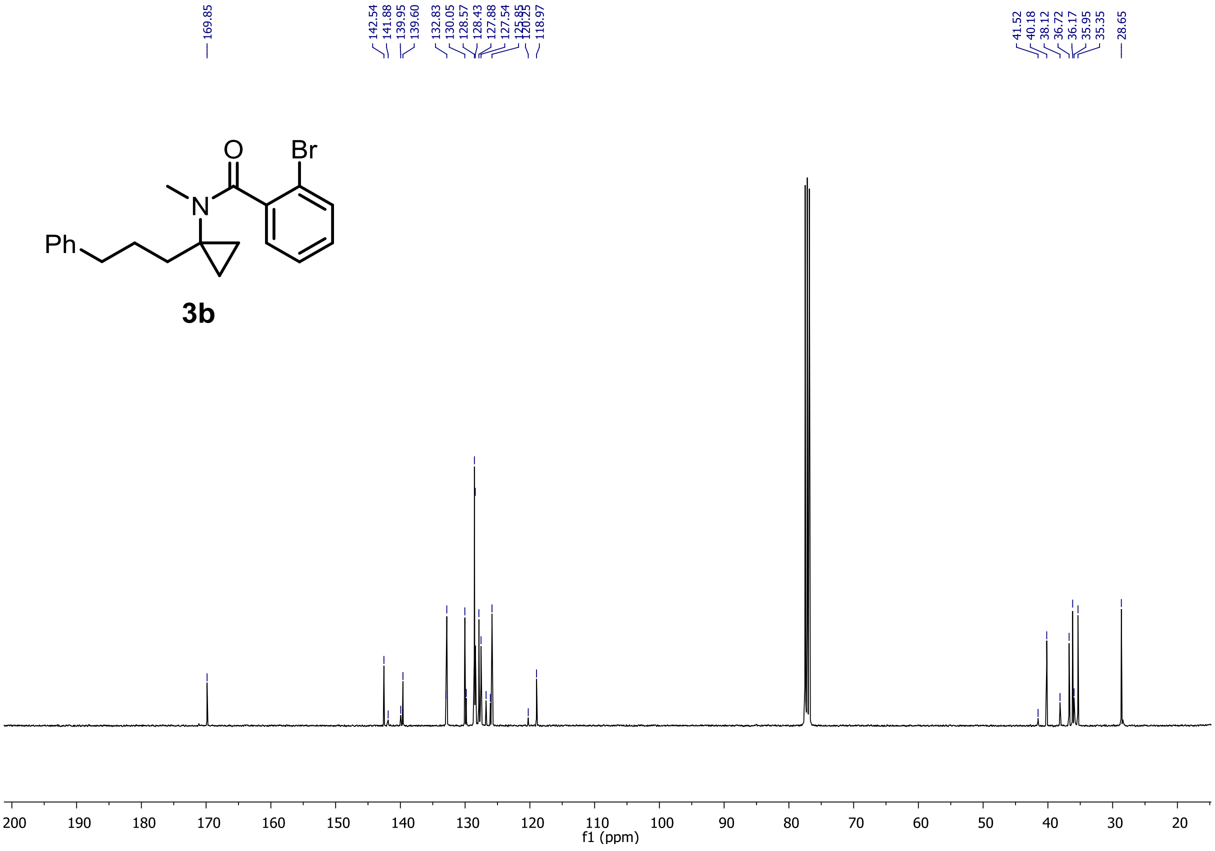
169.85
142.54
141.88
139.95
139.60
132.83
130.05
128.57
128.43
127.88
127.54
125.85
120.25
118.97
41.52
40.18
38.12
36.72
36.17
35.95
35.35
28.65
O
Br
N
Ph
3b
200 190 180 170 160 150 140 130 120 110 100 90 80 70 60 50 40 30 20
f1 (ppm)

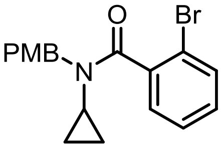

3c

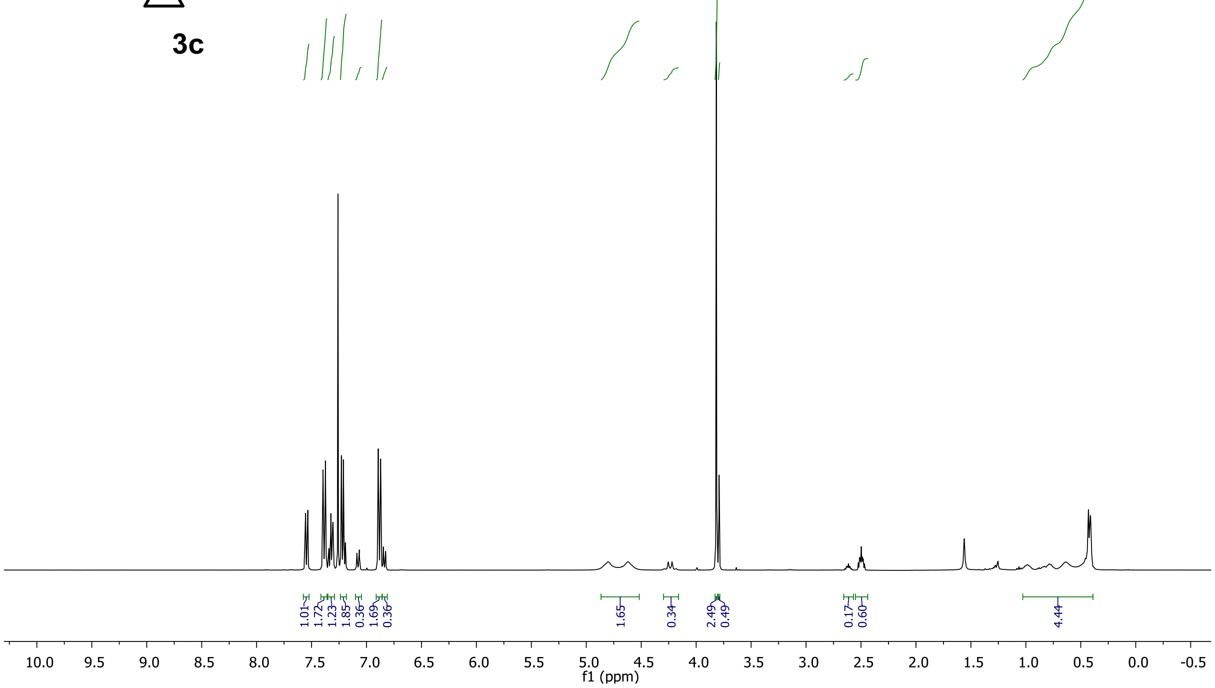
1.01
1.72
1.23
1.85
0.36
1.69
0.36
1.65
0.34
2.49
0.49
0.17
0.60
4.44
10.0 9.5 9.0 8.5 8.0 7.5 7.0 6.5 6.0 5.5 5.0 4.5 4.0 3.5 3.0 2.5 2.0 1.5 1.0 0.5 0.0 -0.5
f1 (ppm)

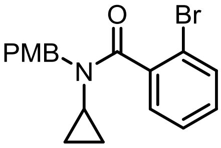

3c

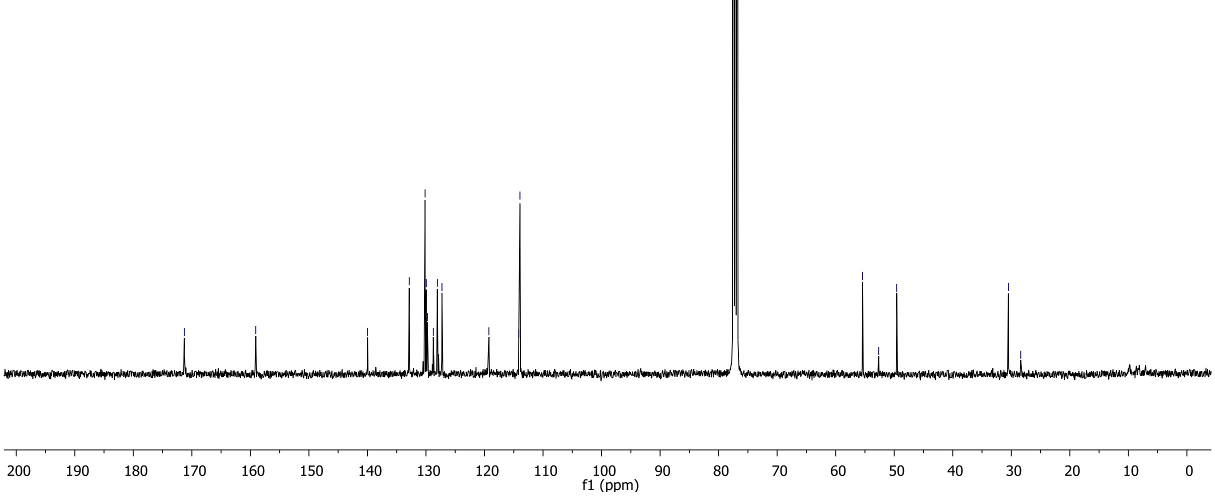
171.28
159.08
139.97
132.85
130.15
129.95
129.75
128.74
128.06
127.27
119.25
114.15
113.94
55.41
52.65
49.58
30.49
28.39
200 190 180 170 160 150 140 130 120 110 100 90 80 70 60 50 40 30 20 10 0
f1 (ppm)

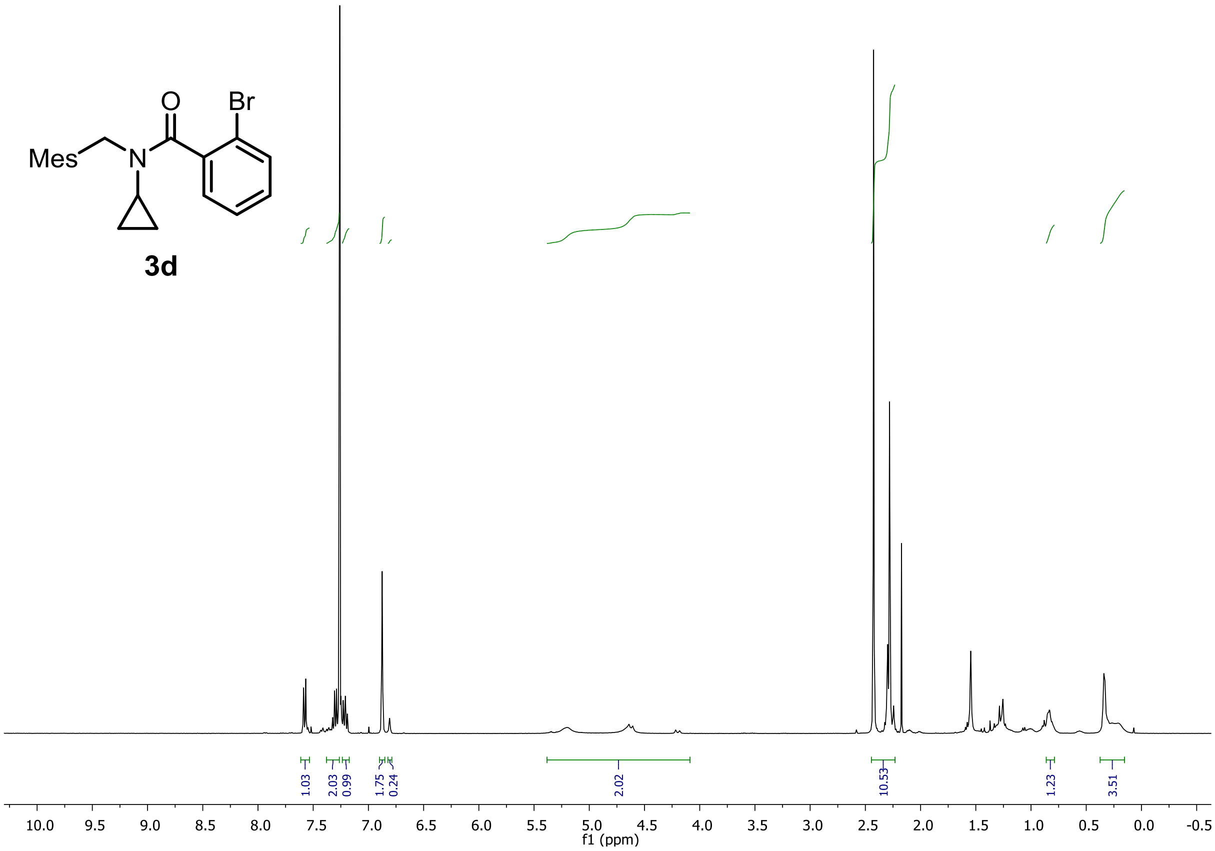
Mes
N
O
Br
3d
1.03
2.03
0.99
1.75
0.24
2.02
10.53
1.23
3.51
f1 (ppm)

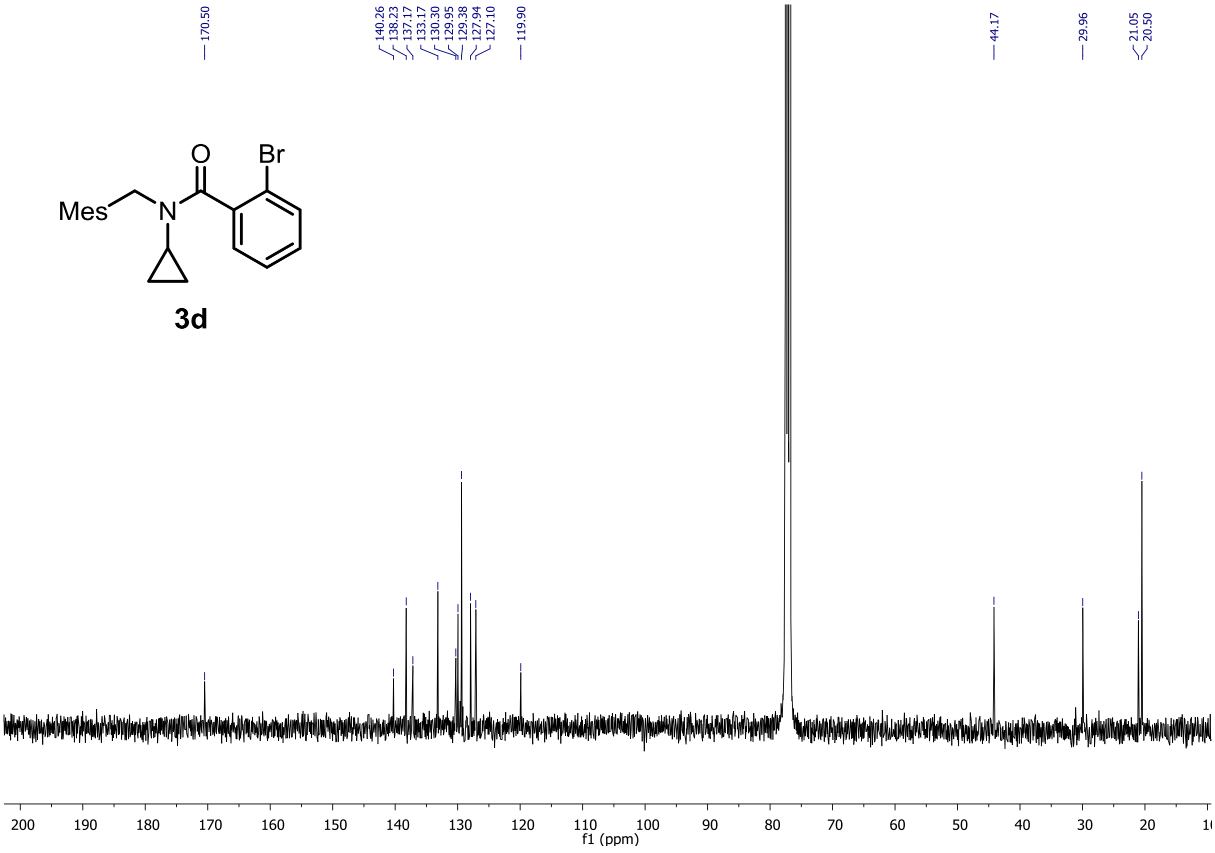
170.50
140.26
138.23
137.17
133.17
130.30
129.95
129.38
127.94
127.10
119.90
44.17
29.96
21.05
20.50
Mes
N
O
Br
3d
f1 (ppm)

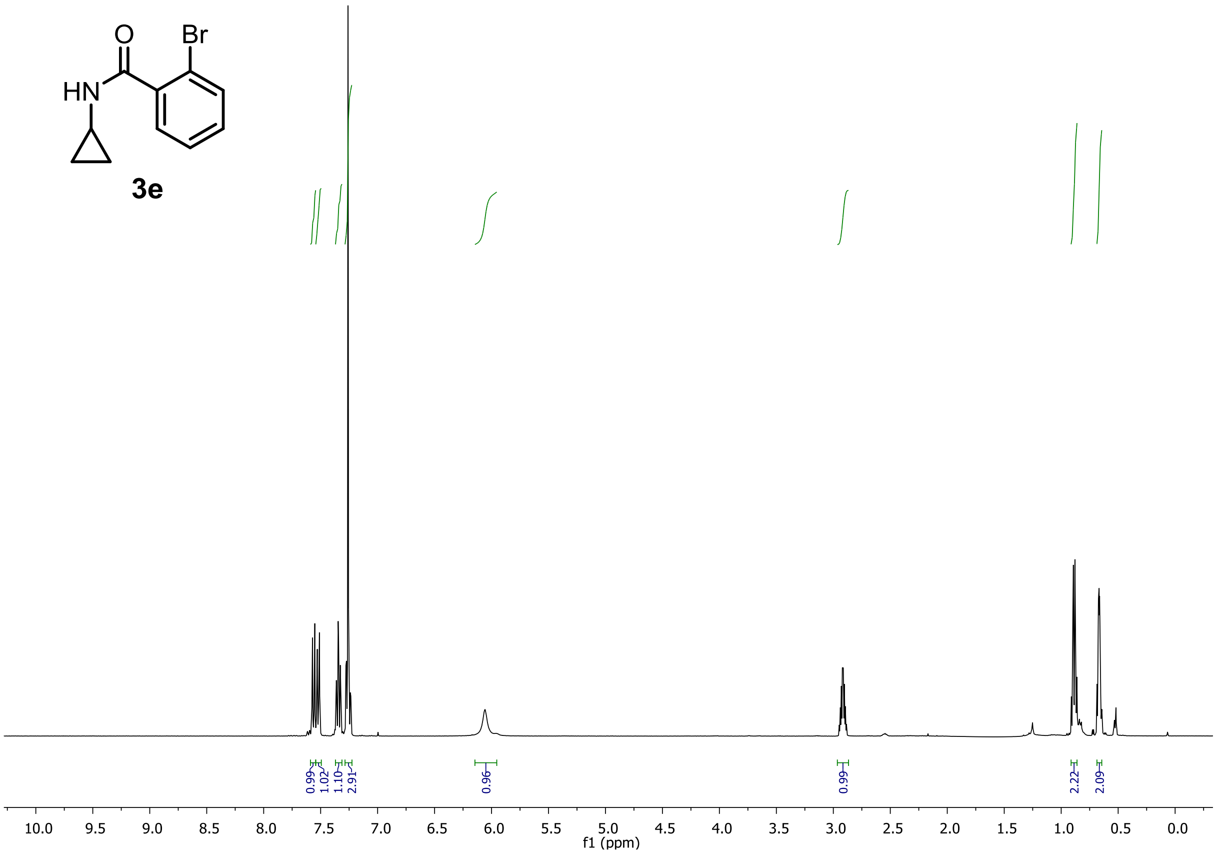
O
Br
HN
3e
0.99
1.02
1.10
2.91
0.96
0.99
2.22
2.09
10.0 9.5 9.0 8.5 8.0 7.5 7.0 6.5 6.0 5.5 5.0 4.5 4.0 3.5 3.0 2.5 2.0 1.5 1.0 0.5 0.0
f1 (ppm)

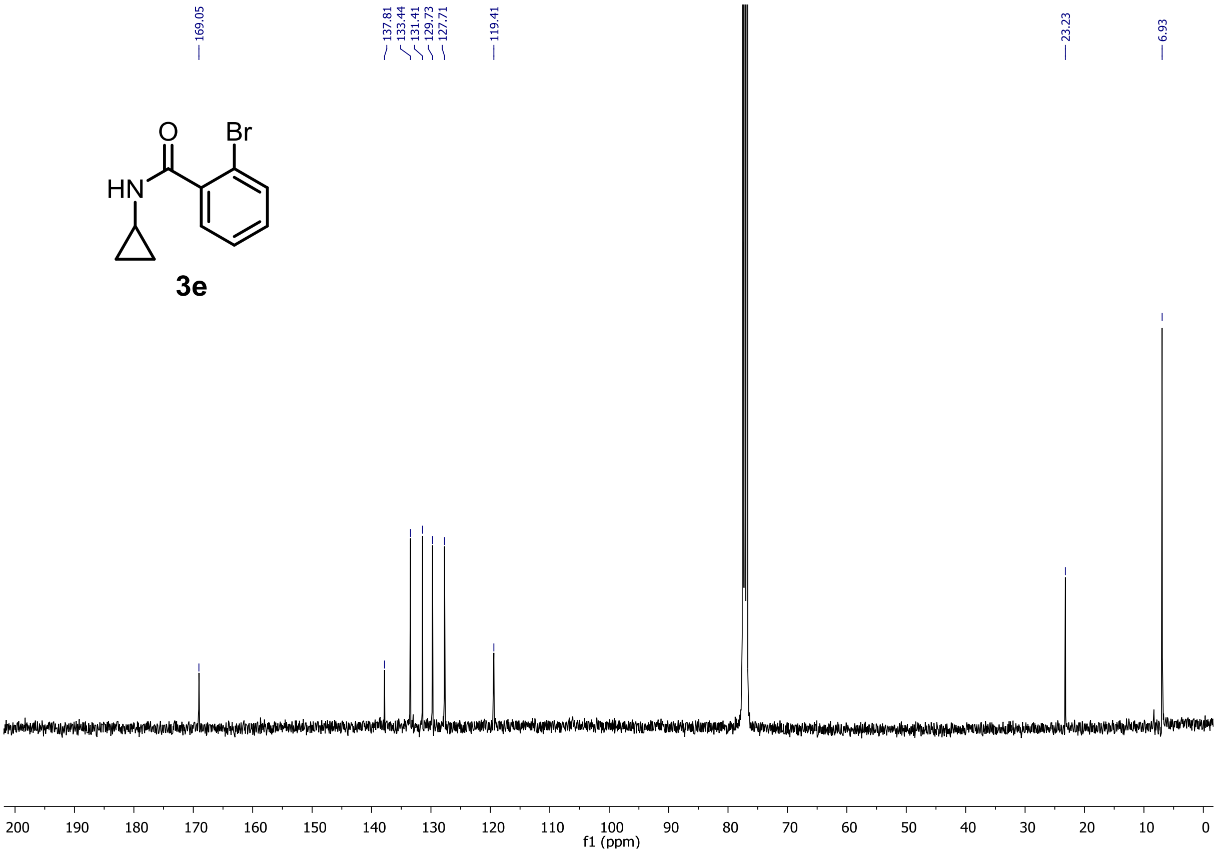
169.05
137.81
133.44
131.41
129.73
127.71
119.41
23.23
6.93
O
Br
HN
3e
200 190 180 170 160 150 140 130 120 110 100 90 80 70 60 50 40 30 20 10 0
f1 (ppm)

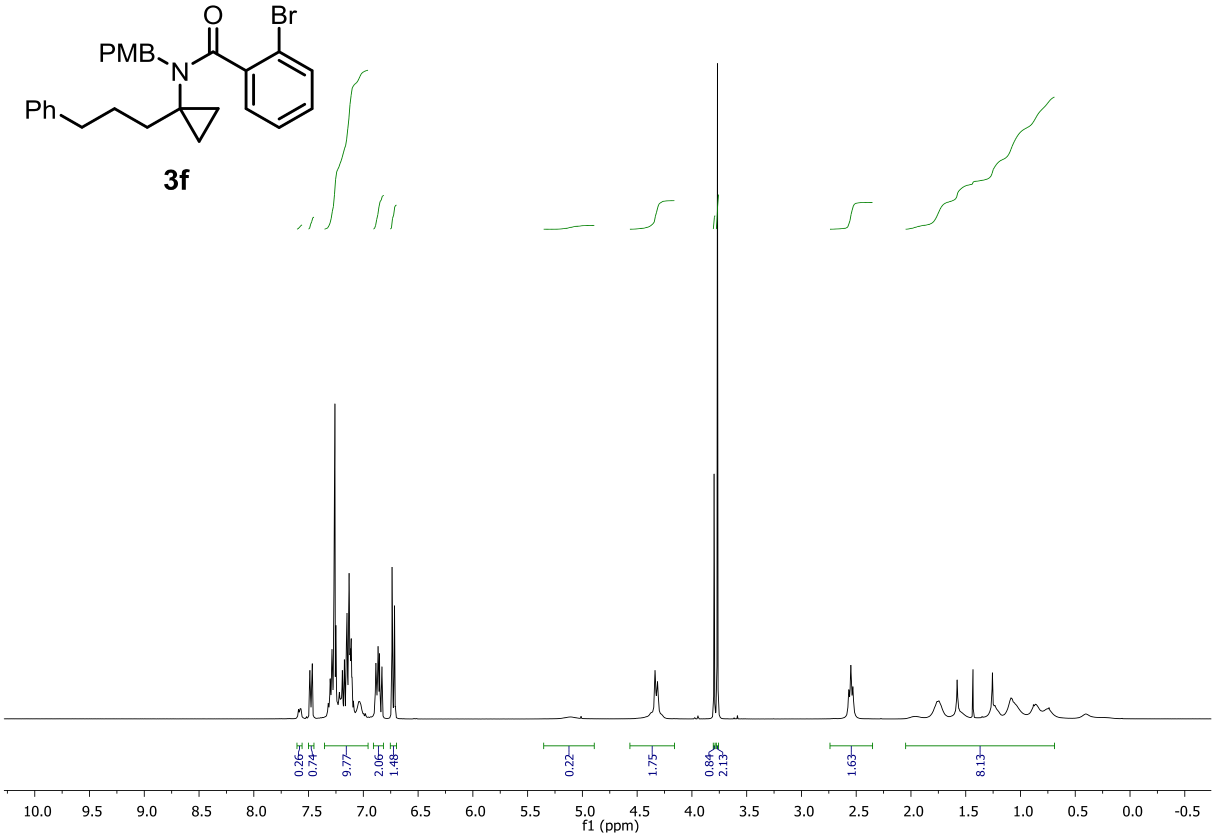

PMB
N
O
Br
Ph
3f
0.26
0.74
9.77
2.06
1.48
0.22
1.75
0.84
2.13
1.63
8.13
f1 (ppm)

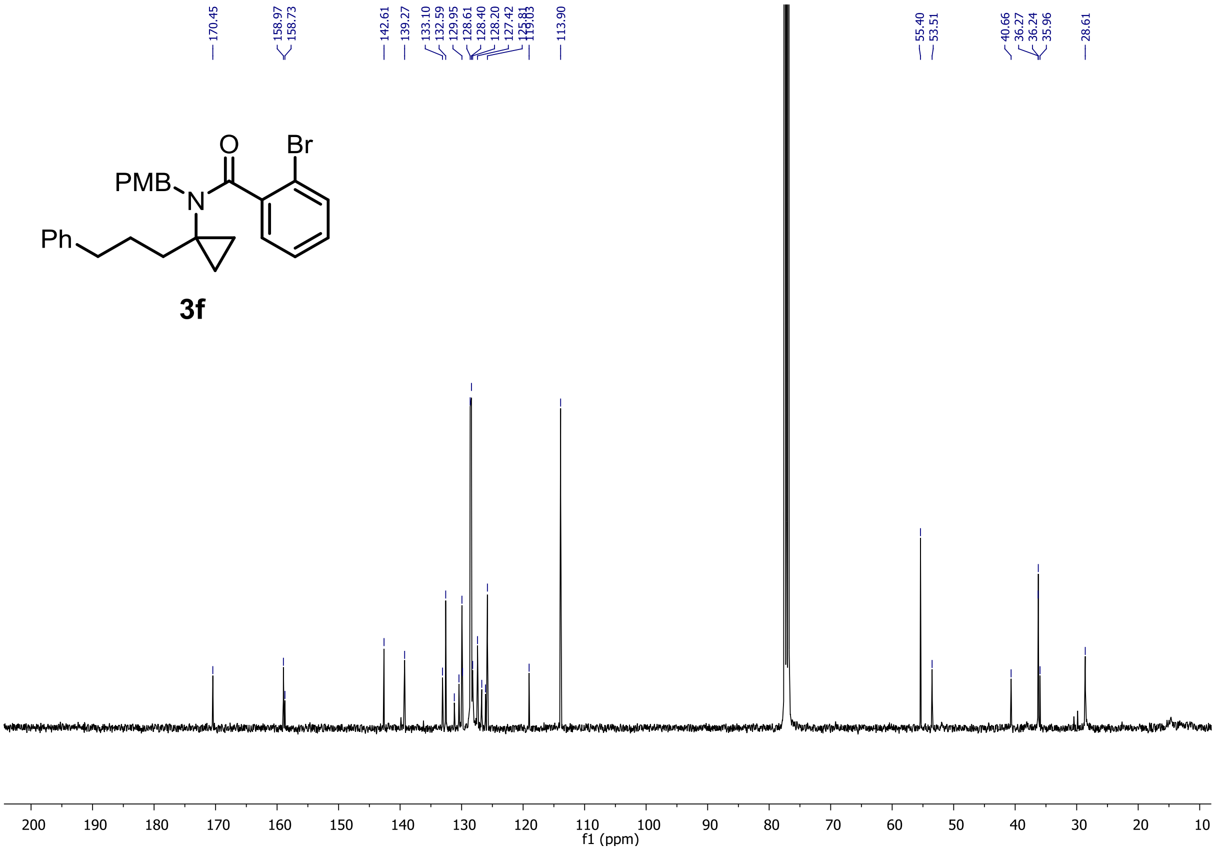

170.45
158.97
158.73
142.61
139.27
133.10
132.59
129.95
128.61
128.40
128.20
127.42
125.81
119.03
113.90
55.40
53.51
40.66
36.27
36.24
35.96
28.61
PMB
N
O
Br
Ph
3f
f1 (ppm)

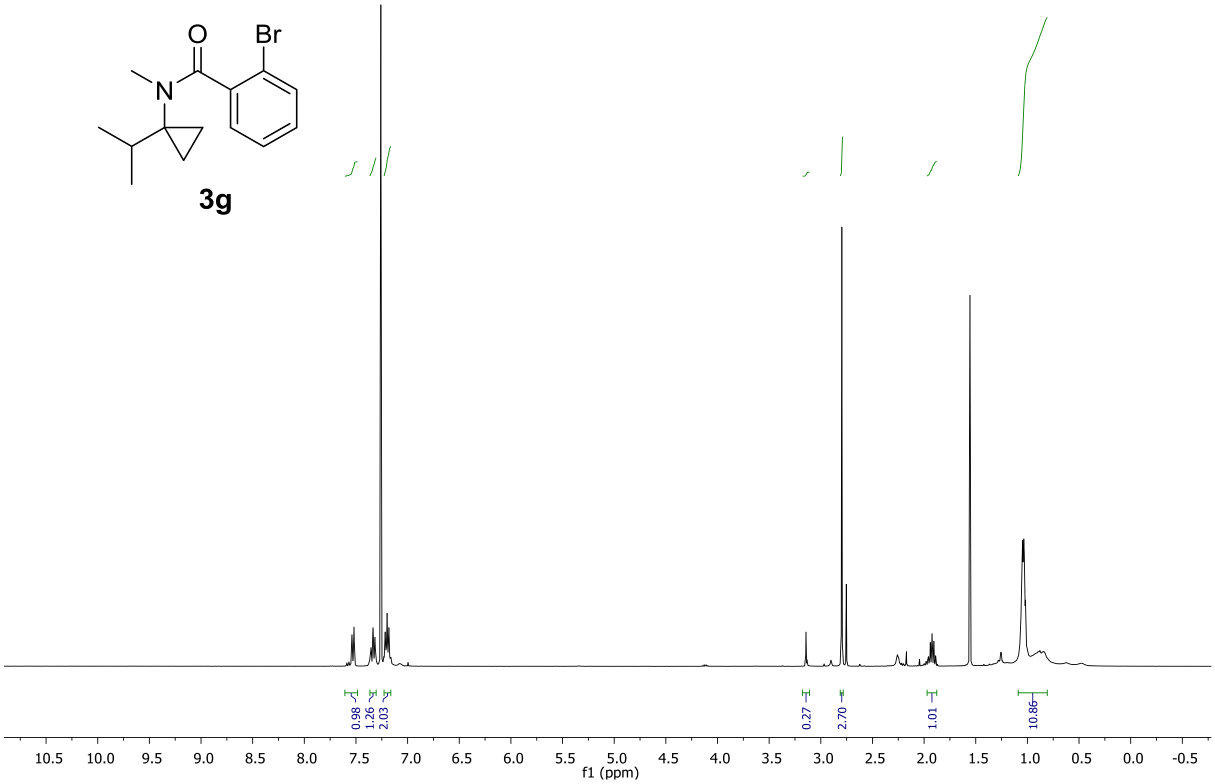
O
Br
N
3g
0.98
1.26
2.03
0.27
2.70
1.01
10.86
10.5 10.0 9.5 9.0 8.5 8.0 7.5 7.0 6.5 6.0 5.5 5.0 4.5 4.0 3.5 3.0 2.5 2.0 1.5 1.0 0.5 0.0 -0.5
f1 (ppm)

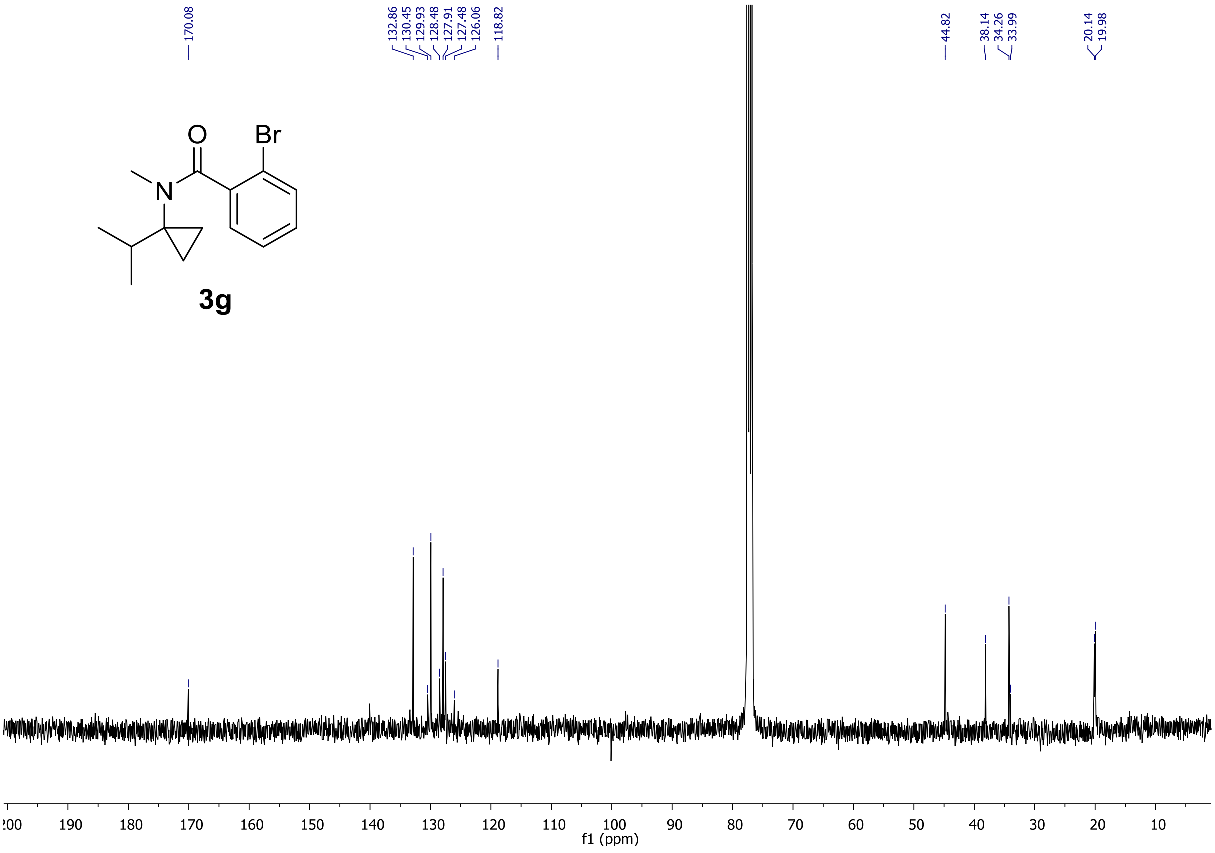
170.08
132.86
130.45
129.93
128.48
127.91
127.48
126.06
118.82
44.82
38.14
34.26
33.99
20.14
19.98
O
Br
N
3g
200 190 180 170 160 150 140 130 120 110 100 90 80 70 60 50 40 30 20 10
f1 (ppm)

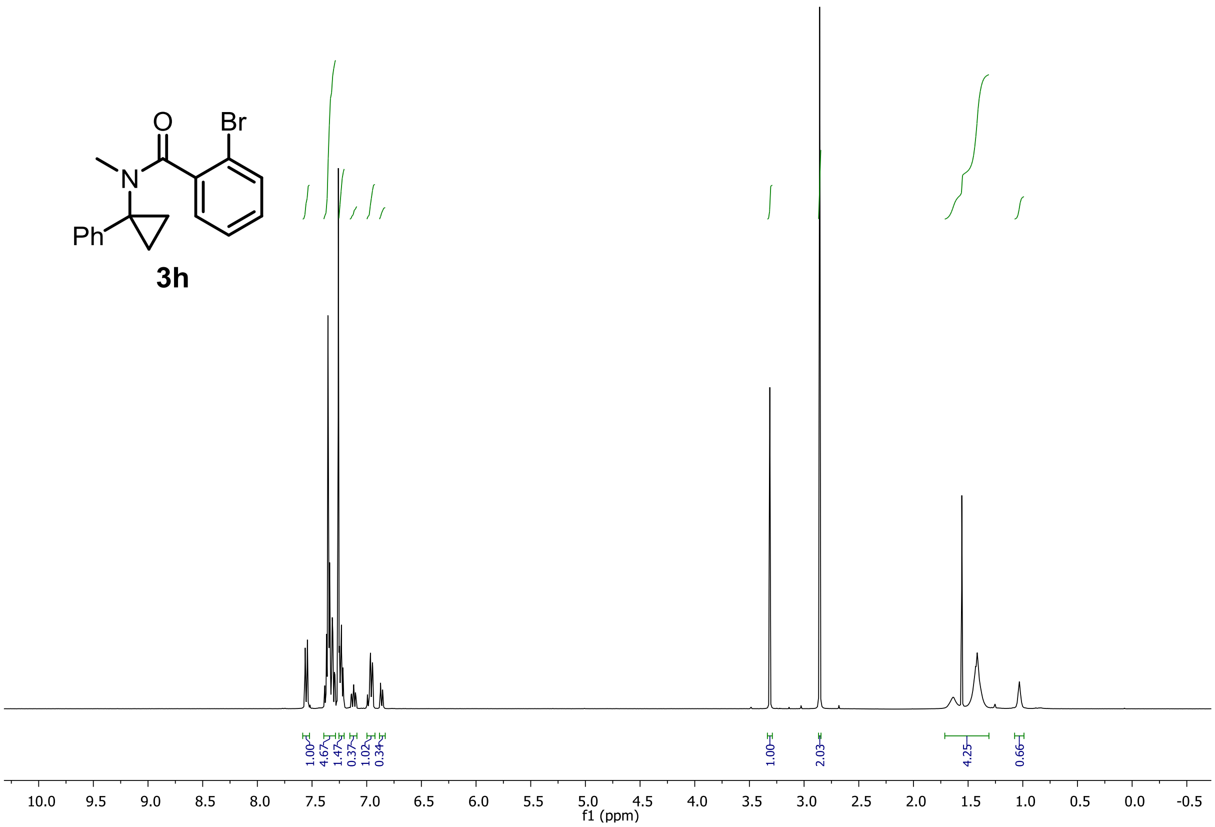

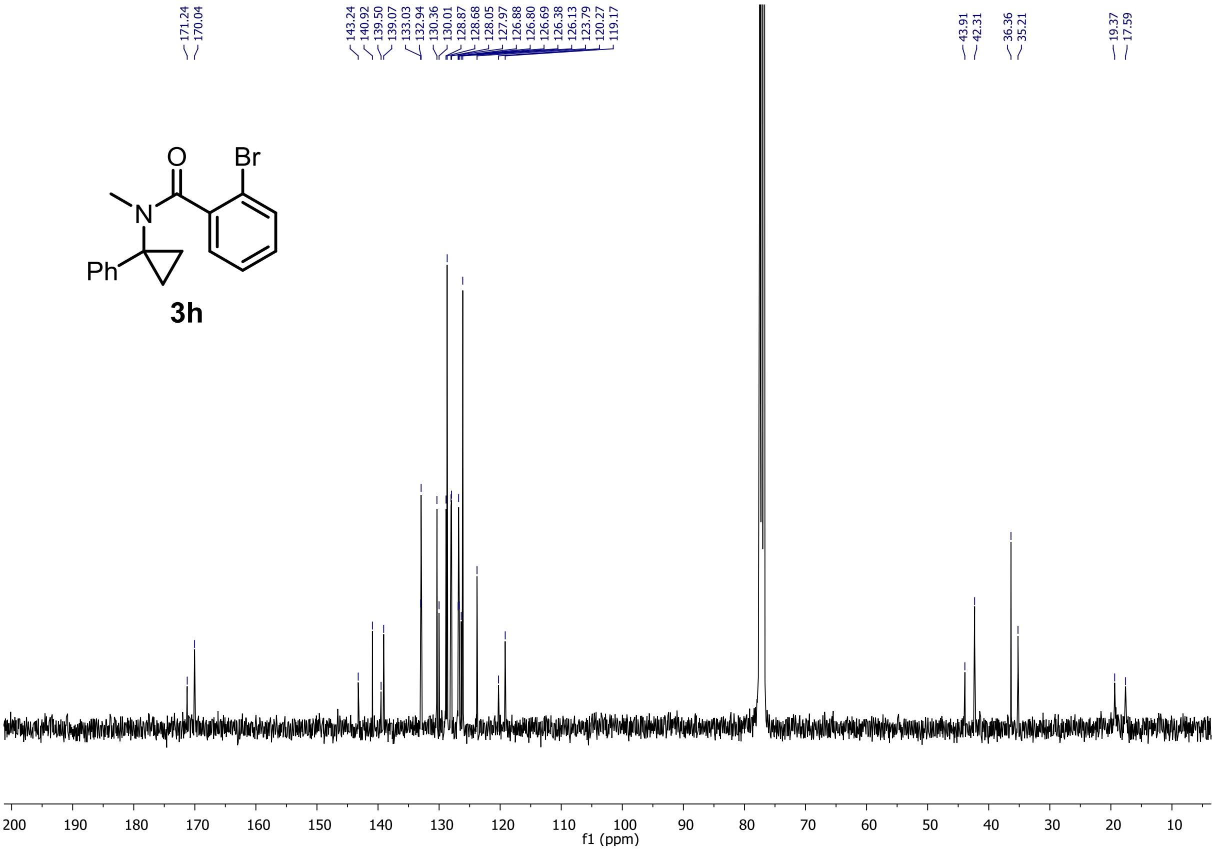

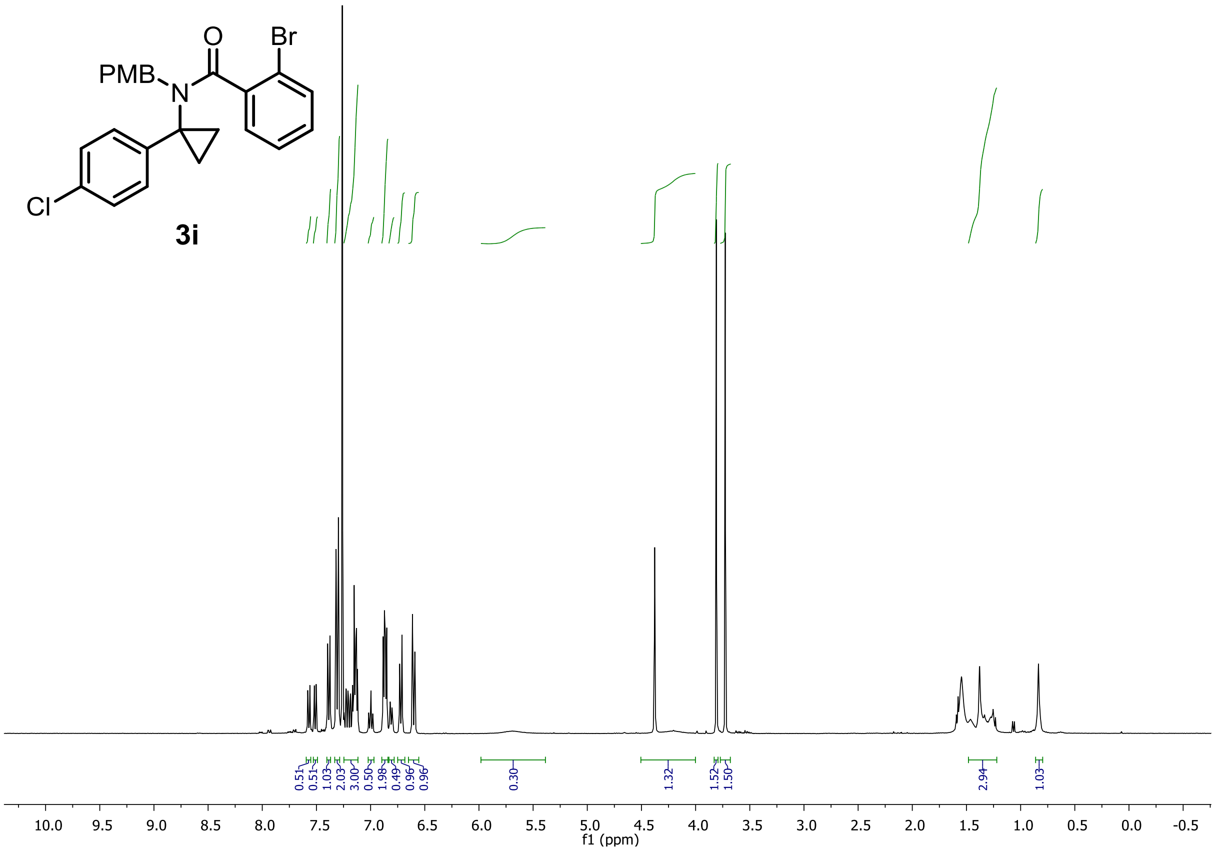
PMB
N
O
Br
Cl
3i
f1 (ppm)

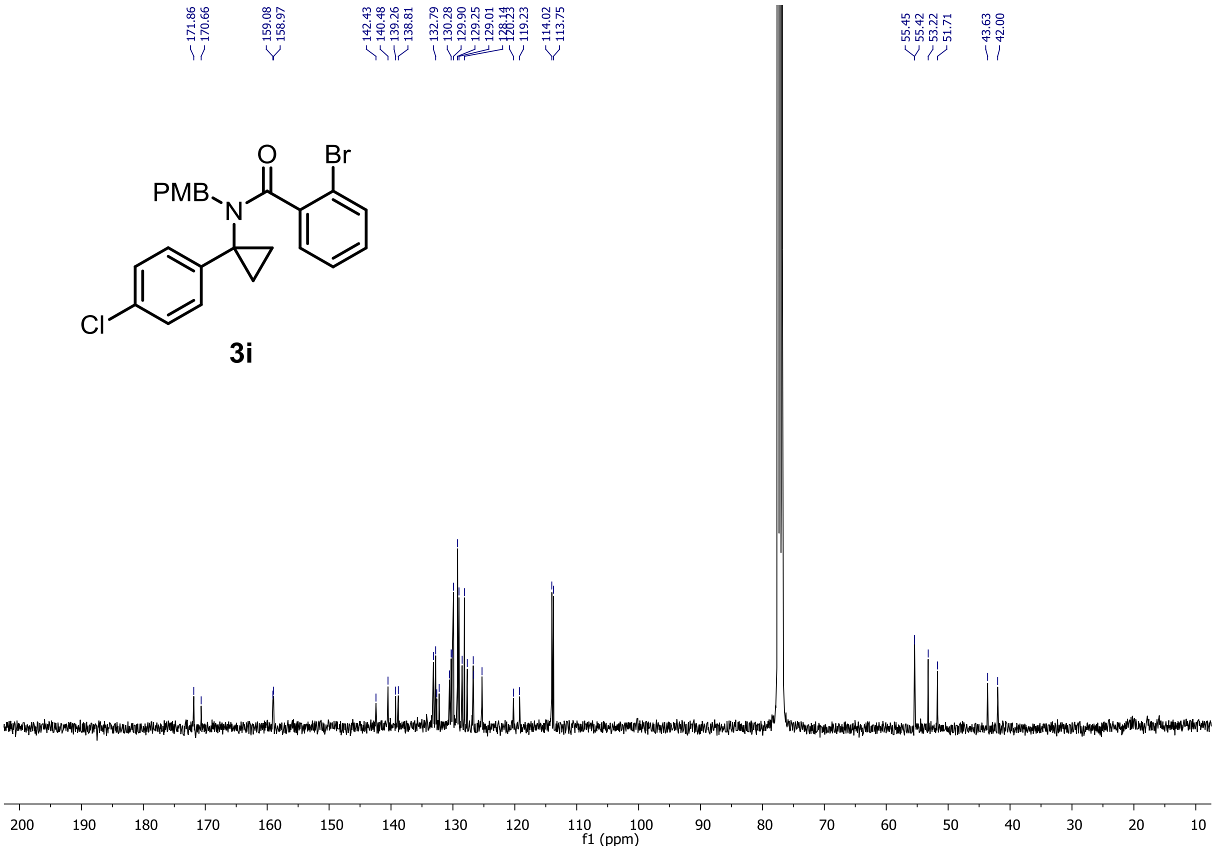
PMB
N
O
Br
Cl
3i
f1 (ppm)

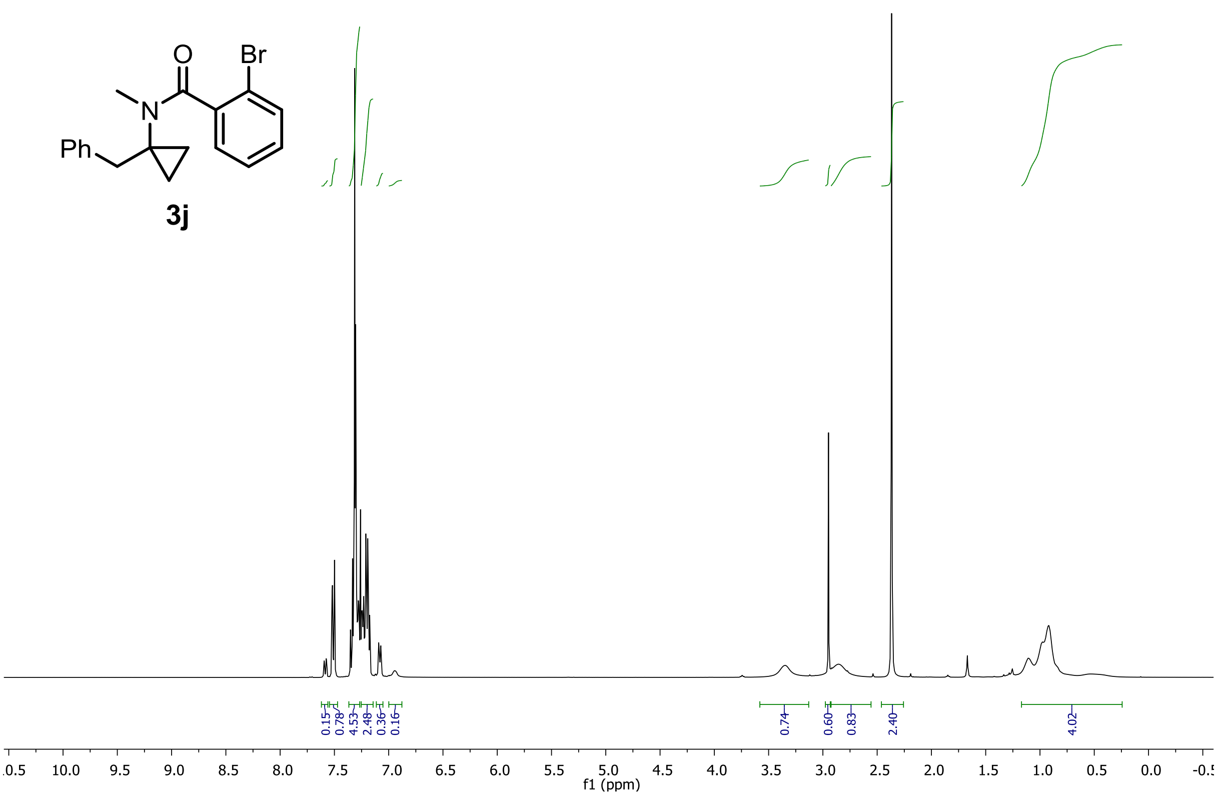
O
Br
N
Ph
0.15
0.78
4.53
2.48
0.36
0.16
0.74
0.60
0.83
2.40
4.02
f1 (ppm)

3j

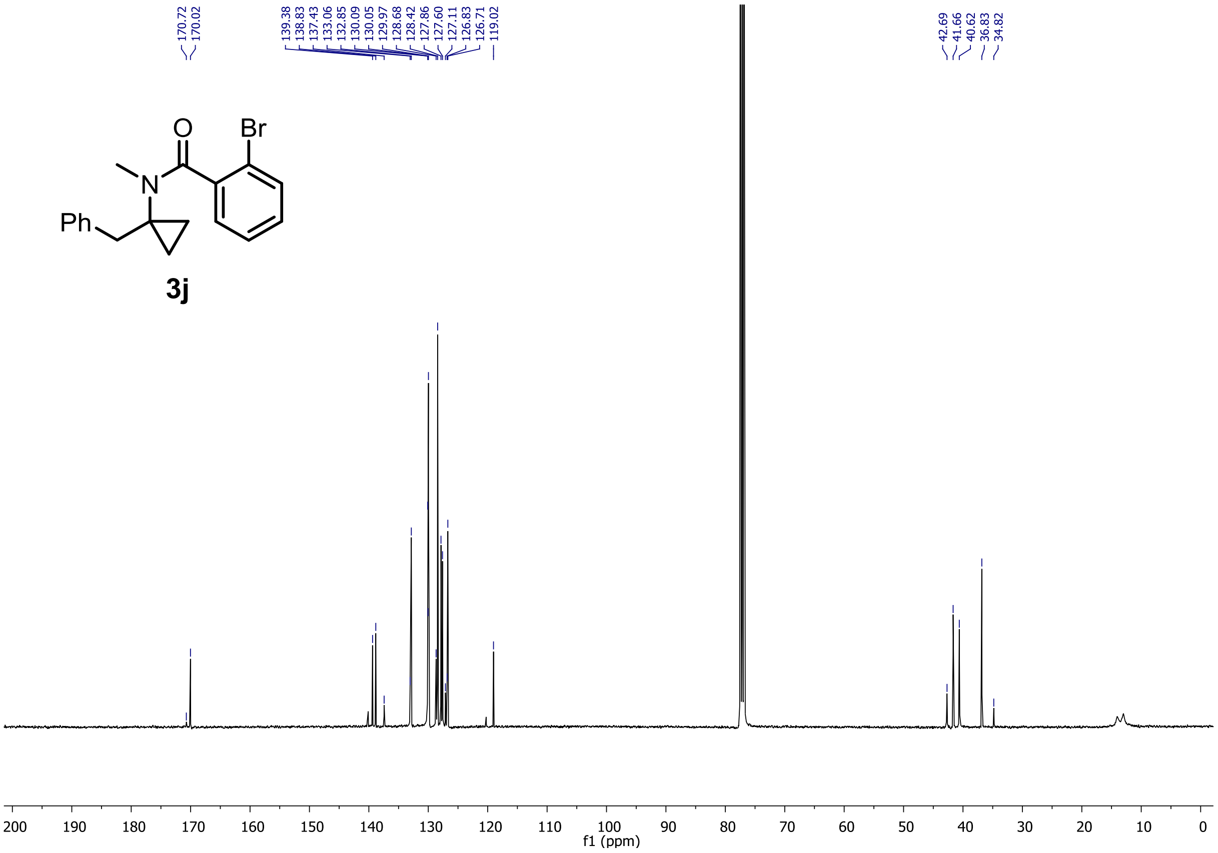
170.72
170.02
139.38
138.83
137.43
133.06
132.85
130.09
130.05
129.97
128.68
128.42
127.86
127.60
127.11
126.83
126.71
119.02
42.69
41.66
40.62
36.83
34.82
O
Br
N
Ph
f1 (ppm)

3j

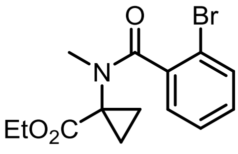
O
Br
N
EtO2C

3k

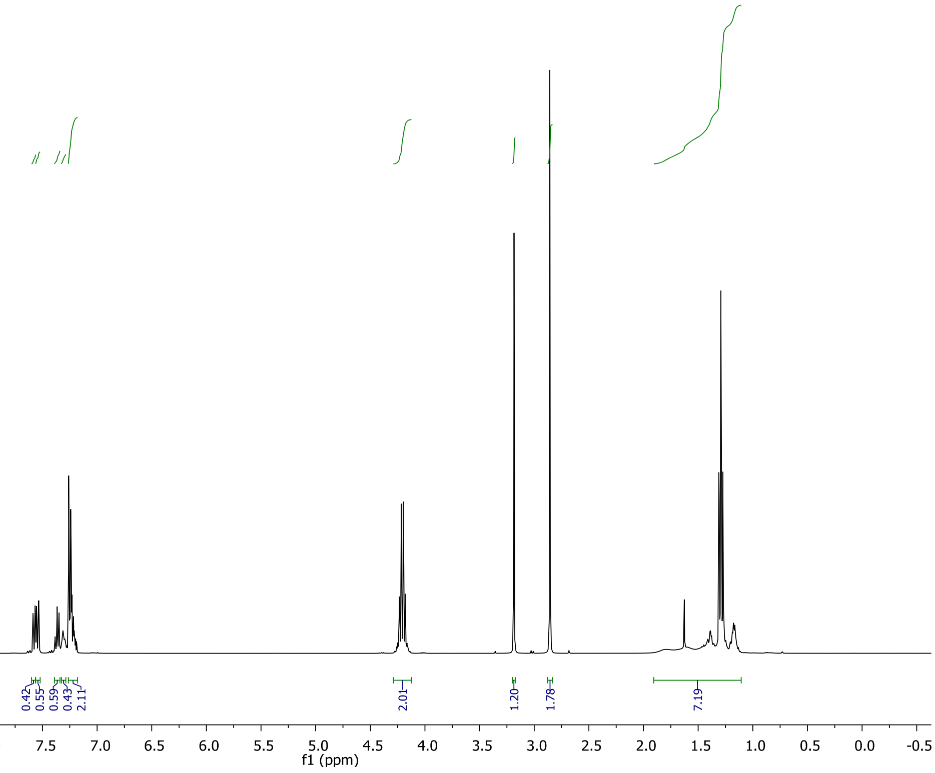
0.42
0.55
0.59
0.43
2.11
2.01
1.20
1.78
7.19
10.0 9.5 9.0 8.5 8.0 7.5 7.0 6.5 6.0 5.5 5.0 4.5 4.0 3.5 3.0 2.5 2.0 1.5 1.0 0.5 0.0 -0.5
f1 (ppm)

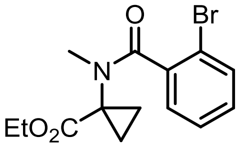
O
Br
N
EtO2C

3k

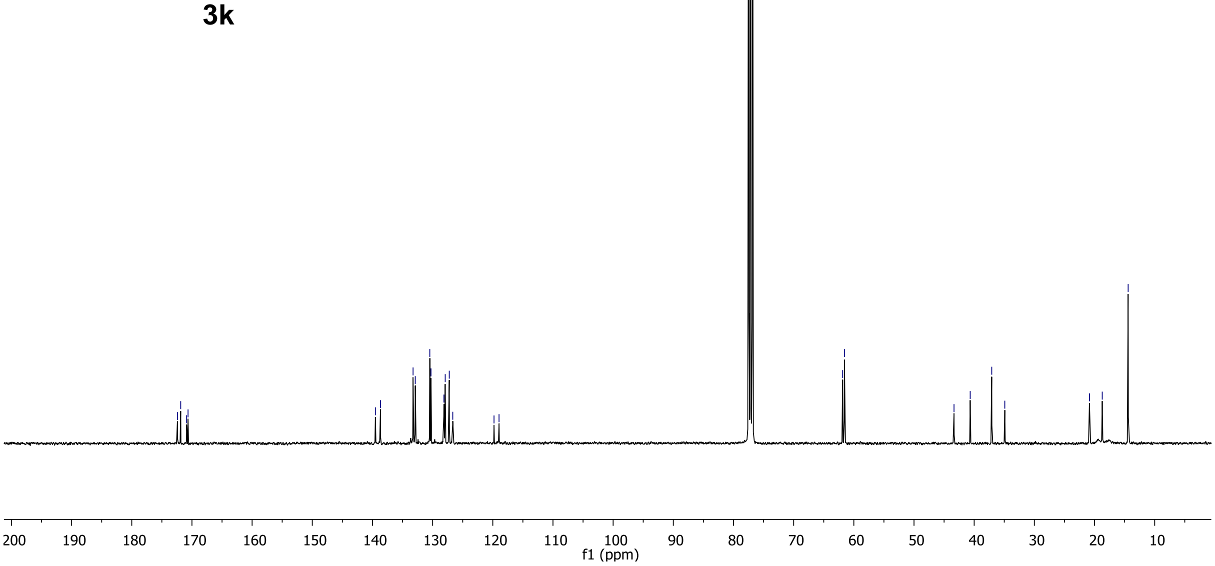
172.41
171.86
170.88
170.65
139.51
138.67
133.25
132.89
130.47
130.27
127.91
127.23
119.80
118.96
61.86
61.56
43.35
40.66
37.08
34.91
20.84
18.72
14.41
200 190 180 170 160 150 140 130 120 110 100 90 80 70 60 50 40 30 20 10
f1 (ppm)

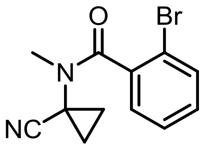
O
Br
N
NC

3l

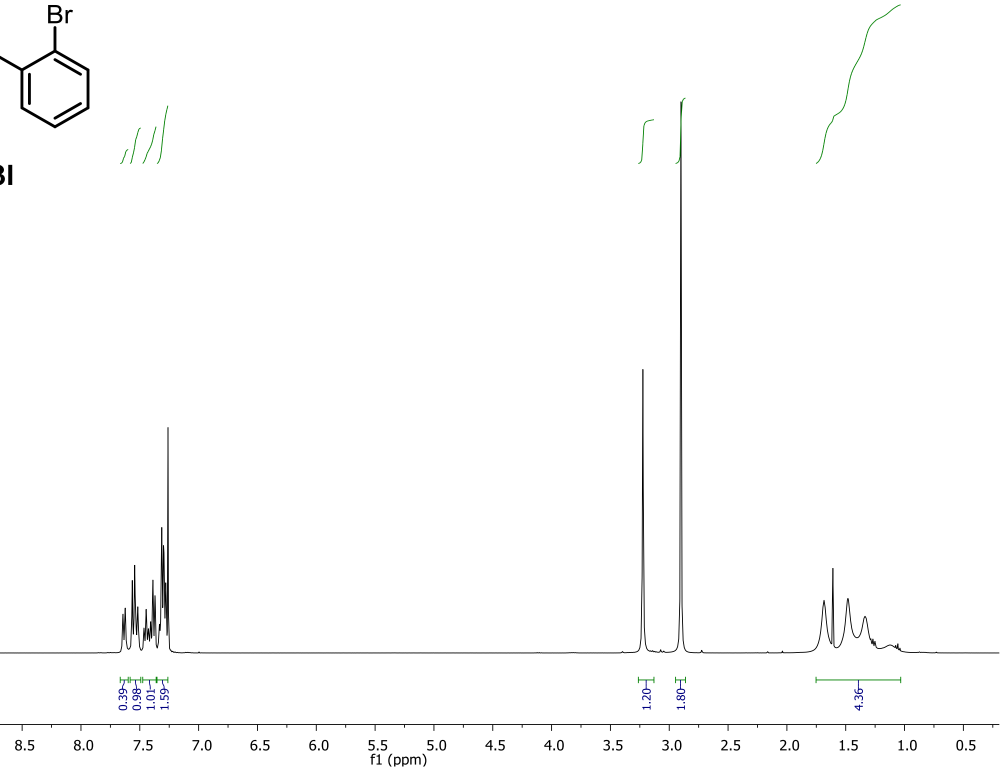
0.39
0.98
1.01
1.59
1.20
1.80
4.36
10.0 9.5 9.0 8.5 8.0 7.5 7.0 6.5 6.0 5.5 5.0 4.5 4.0 3.5 3.0 2.5 2.0 1.5 1.0 0.5
f1 (ppm)

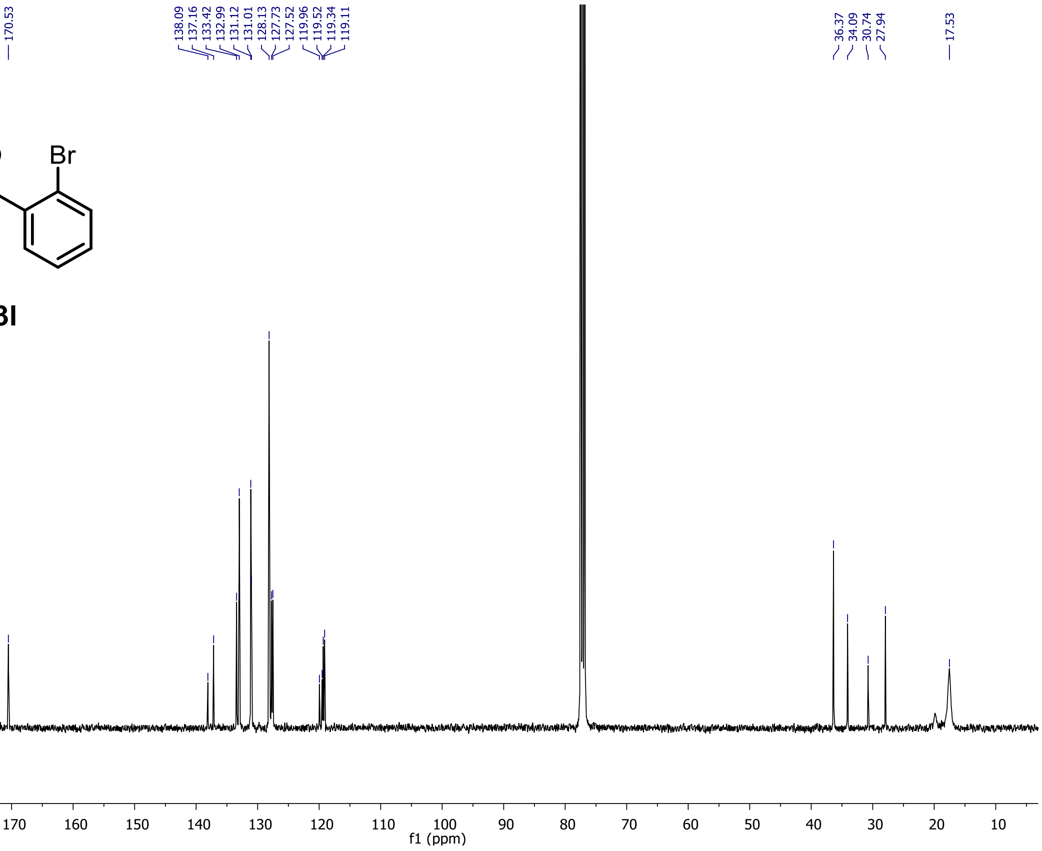
170.53
138.09
137.16
133.42
132.99
131.12
131.01
128.13
127.73
127.52
119.96
119.52
119.34
119.11
36.37
34.09
30.74
27.94
17.53
190 180 170 160 150 140 130 120 110 100 90 80 70 60 50 40 30 20 10
f1 (ppm)

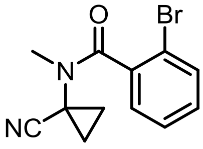
O
Br
N
NC

3l

3m

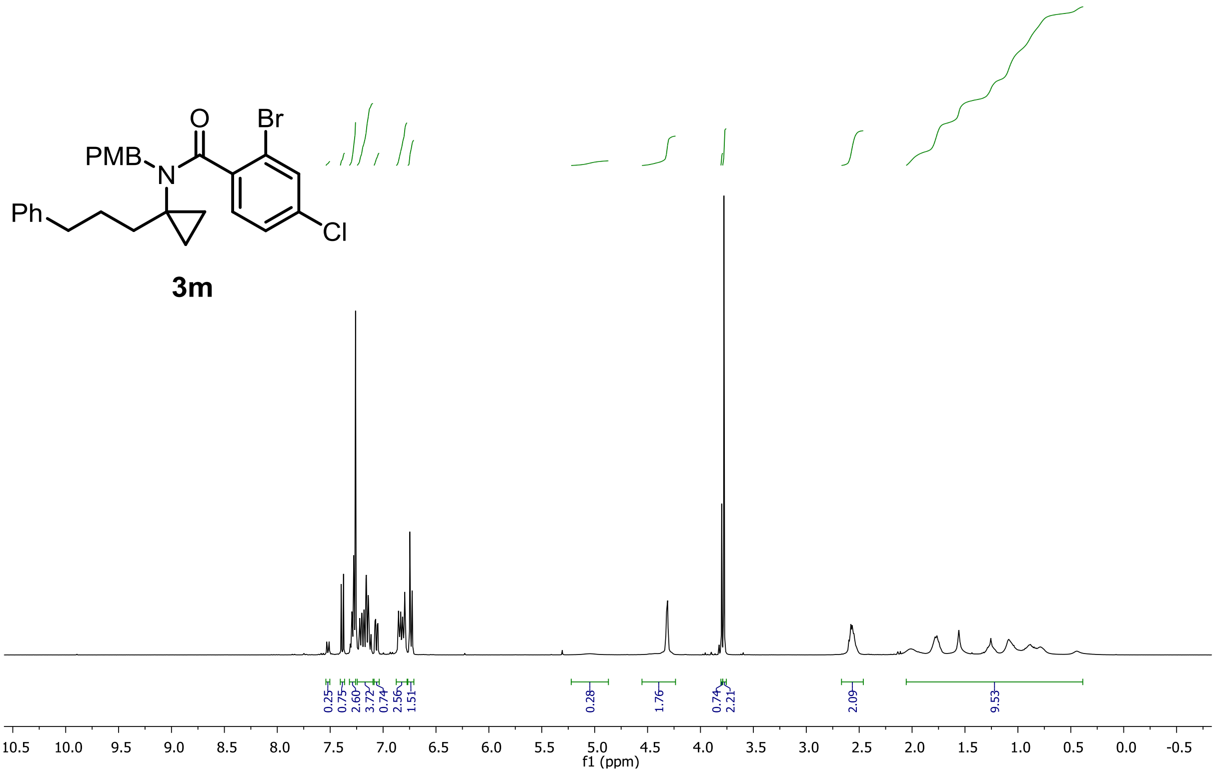
0.25 0.75 2.60 3.72 0.74 2.56 1.51 0.28 1.76 0.74 2.21 2.09 9.53
10.5 10.0 9.5 9.0 8.5 8.0 7.5 7.0 6.5 6.0 5.5 5.0 4.5 4.0 3.5 3.0 2.5 2.0 1.5 1.0 0.5 0.0 -0.5
f1 (ppm)

3m

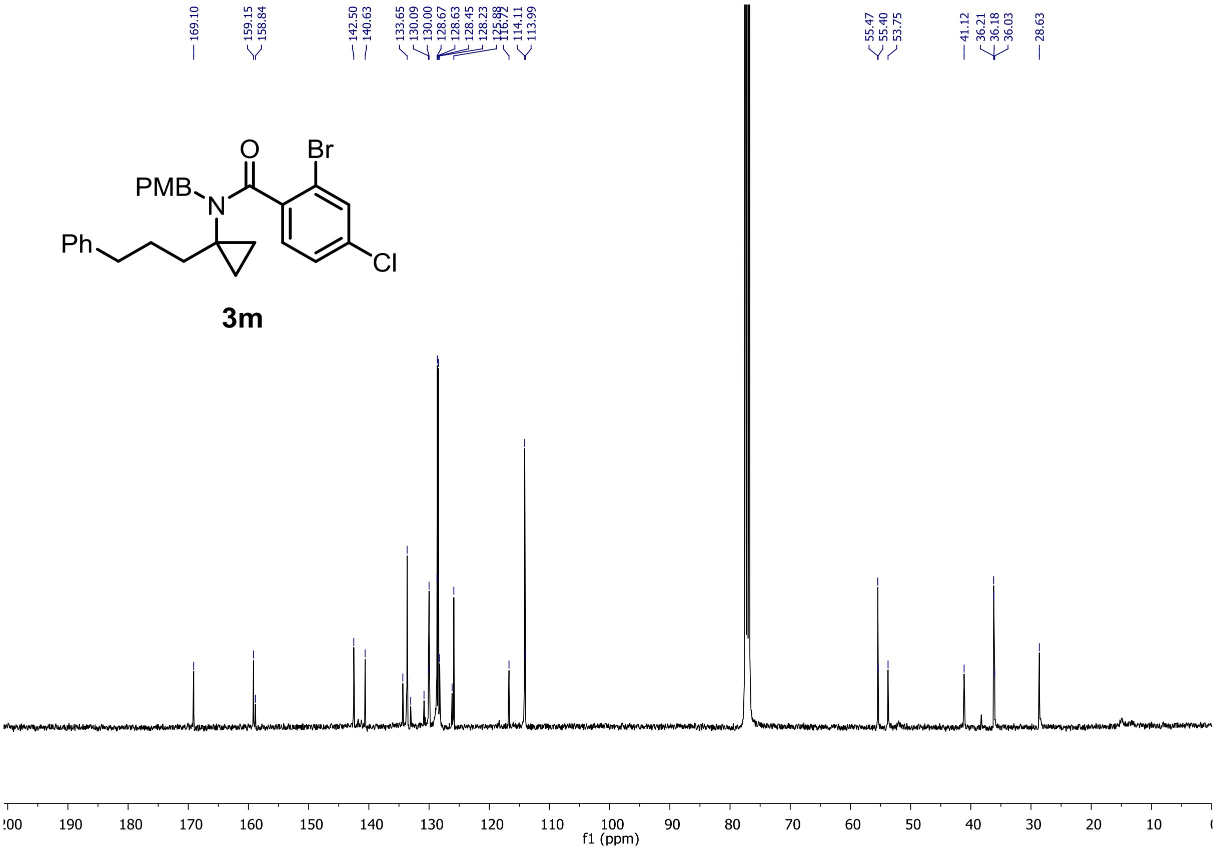
169.10
159.15
158.84
142.50
140.63
133.65
130.09
130.00
128.67
128.63
128.45
128.23
125.88
116.72
114.11
113.99
55.47
55.40
53.75
41.12
36.21
36.18
36.03
28.63
190 180 170 160 150 140 130 120 110 100 90 80 70 60 50 40 30 20 10
f1 (ppm)

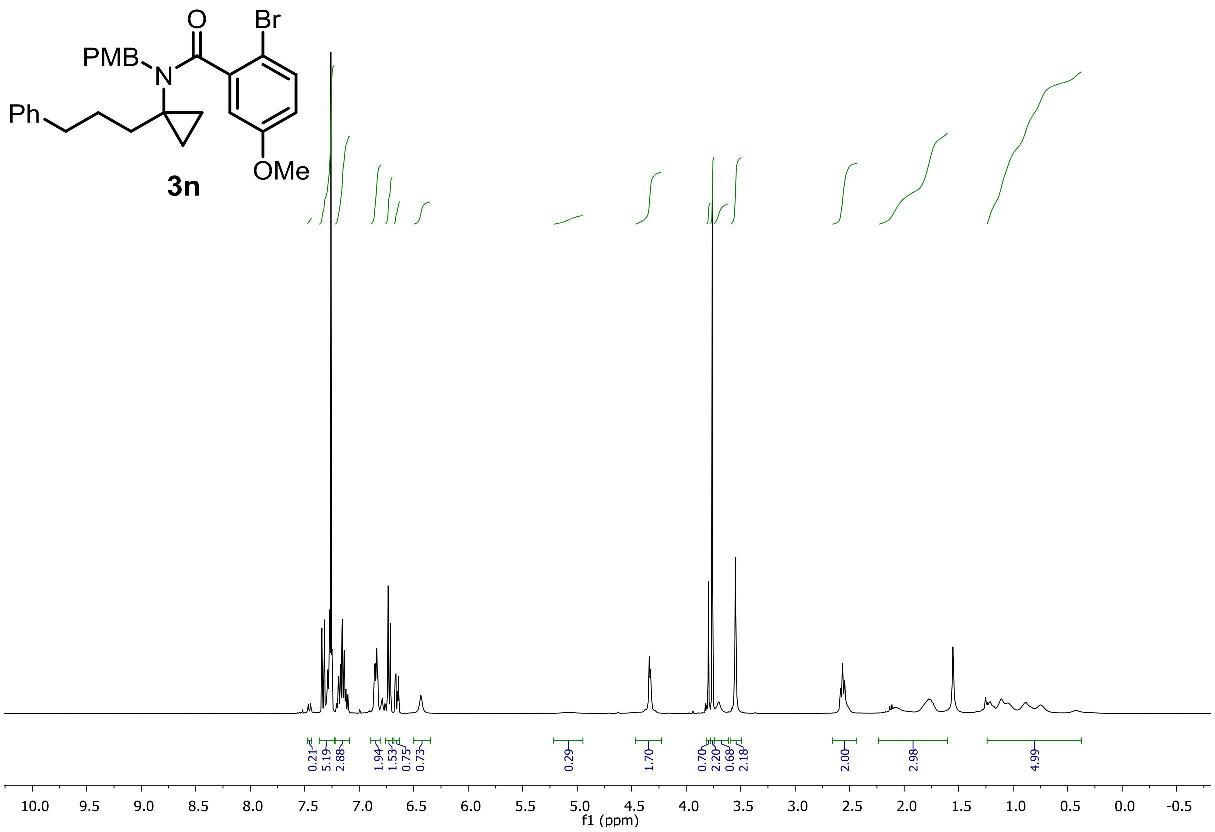
PMB
N
O
Br
Ph
OMe
3n
0.21
5.19
2.88
1.94
1.53
0.75
0.73
0.29
1.70
0.70
2.20
0.68
2.18
2.00
2.98
4.99
10.0 9.5 9.0 8.5 8.0 7.5 7.0 6.5 6.0 5.5 5.0 4.5 4.0 3.5 3.0 2.5 2.0 1.5 1.0 0.5 0.0 -0.5
f1 (ppm)

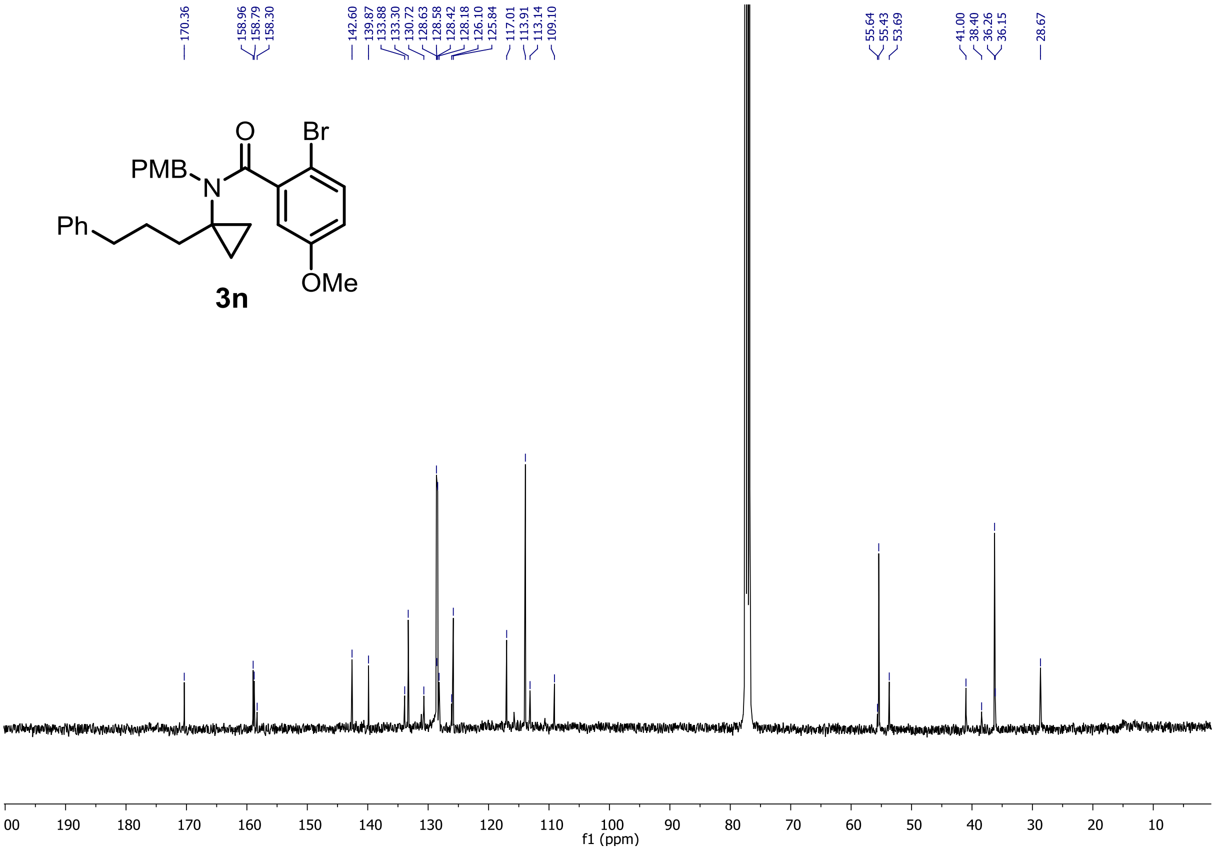
170.36
158.96
158.79
158.30
142.60
139.87
133.88
133.30
130.72
128.63
128.58
128.42
128.18
126.10
125.84
117.01
113.91
113.14
109.10
55.64
55.43
53.69
41.00
38.40
36.26
36.15
28.67
PMB
N
O
Br
Ph
OMe
3n
00 190 180 170 160 150 140 130 120 110 100 90 80 70 60 50 40 30 20 10
f1 (ppm)

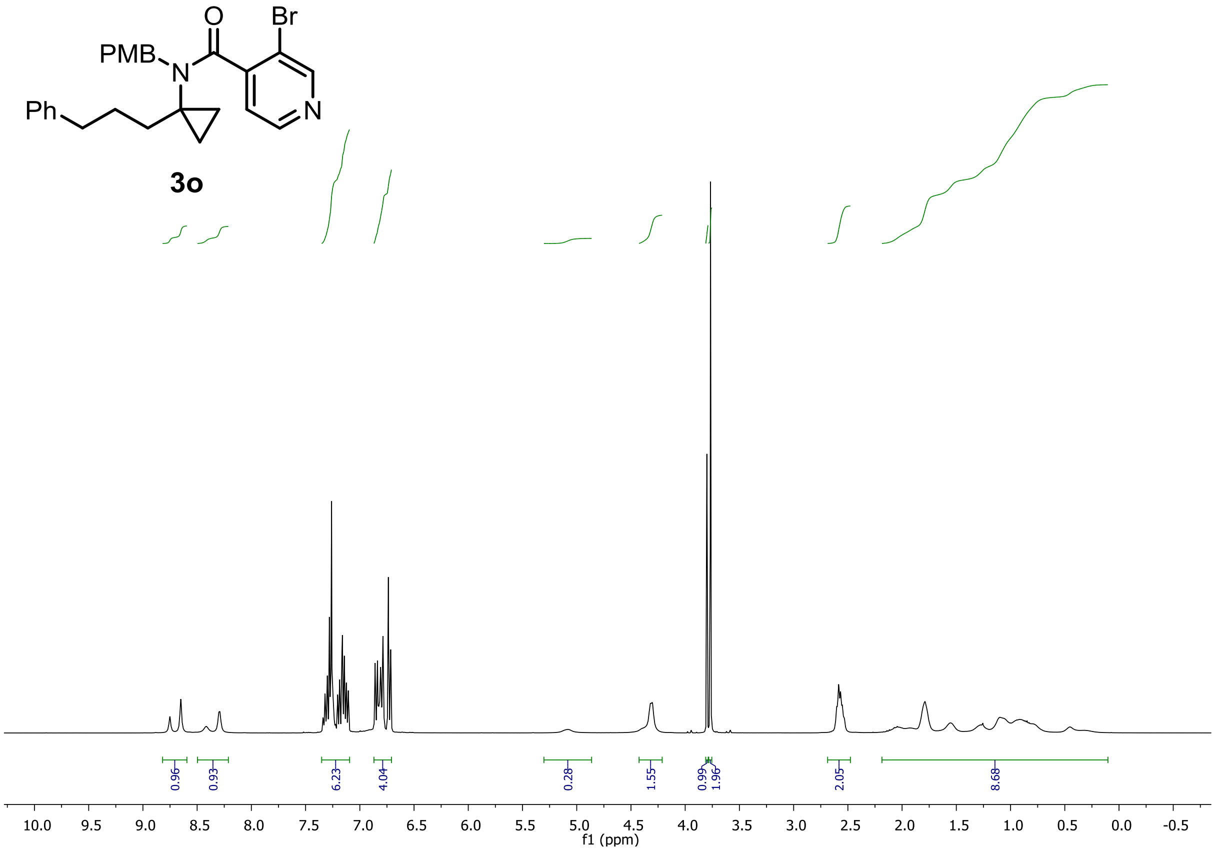
PMB
N
O
Br
N
Ph
3o
0.96
0.93
6.23
4.04
0.28
1.55
0.99
1.96
2.05
8.68
10.0 9.5 9.0 8.5 8.0 7.5 7.0 6.5 6.0 5.5 5.0 4.5 4.0 3.5 3.0 2.5 2.0 1.5 1.0 0.5 0.0 -0.5
f1 (ppm)

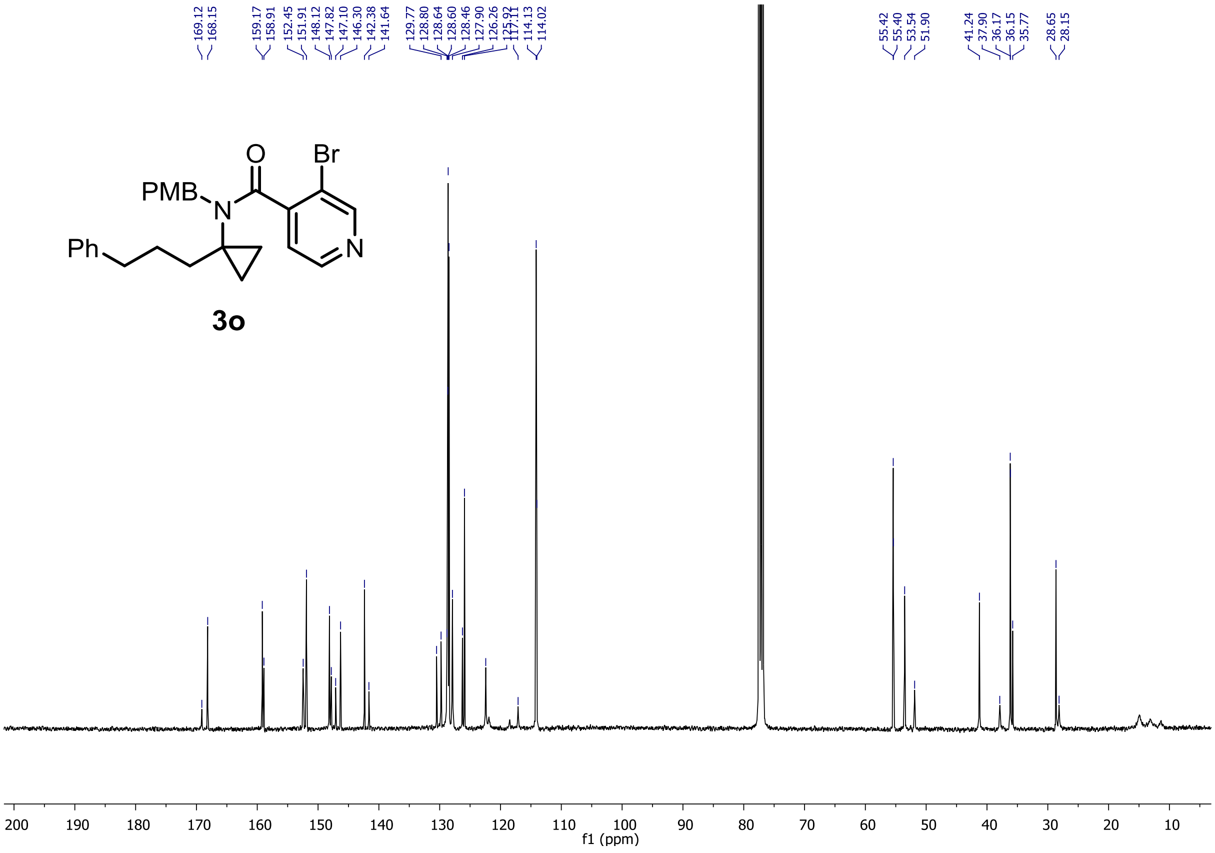
169.12
168.15
159.17
158.91
152.45
151.91
148.12
147.82
147.10
146.30
142.38
141.64
129.77
128.80
128.64
128.60
128.46
127.90
126.26
125.92
117.11
114.13
114.02
55.42
55.40
53.54
51.90
41.24
37.90
36.17
36.15
35.77
28.65
28.15
PMB
N
O
Br
N
Ph
3o
200 190 180 170 160 150 140 130 120 110 100 90 80 70 60 50 40 30 20 10
f1 (ppm)

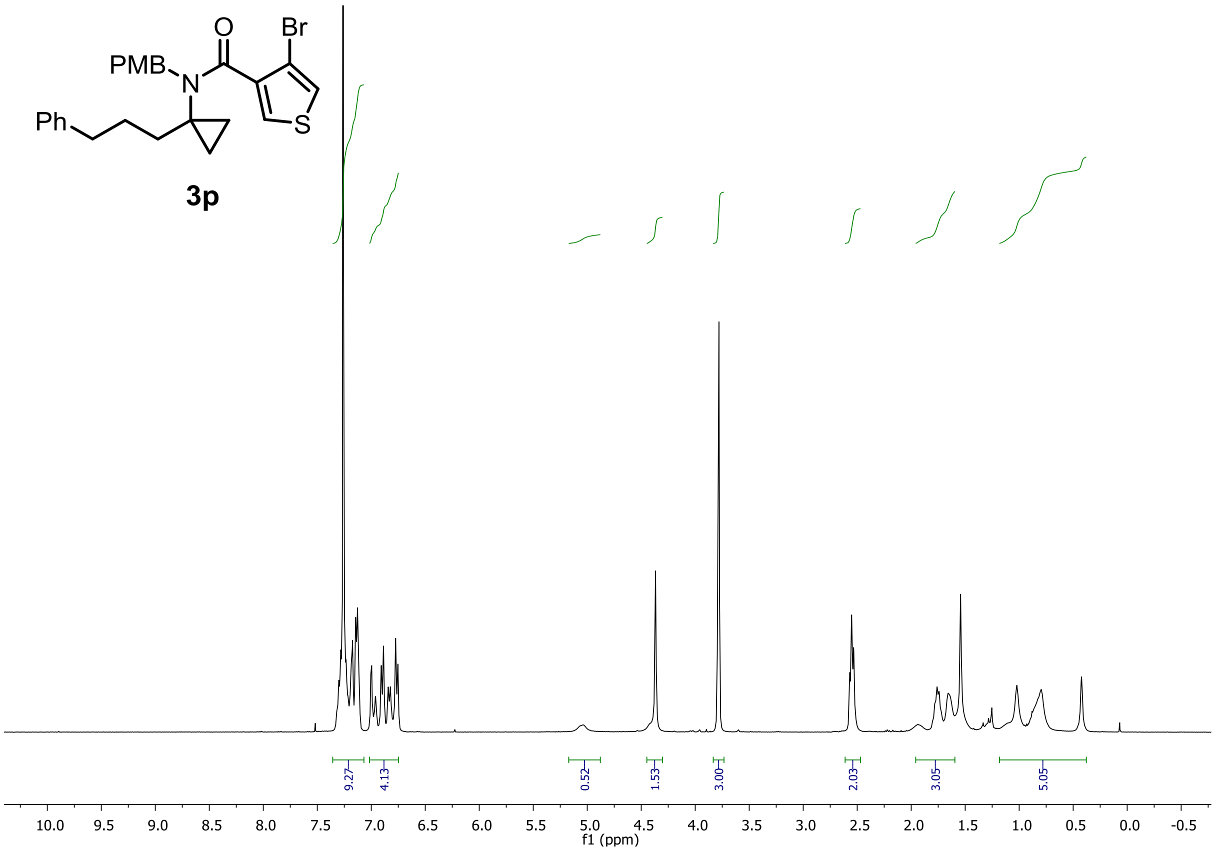
PMB
N
O
Br
S
Ph
3p
9.27
4.13
0.52
1.53
3.00
2.03
3.05
5.05
10.0 9.5 9.0 8.5 8.0 7.5 7.0 6.5 6.0 5.5 5.0 4.5 4.0 3.5 3.0 2.5 2.0 1.5 1.0 0.5 0.0 -0.5
f1 (ppm)

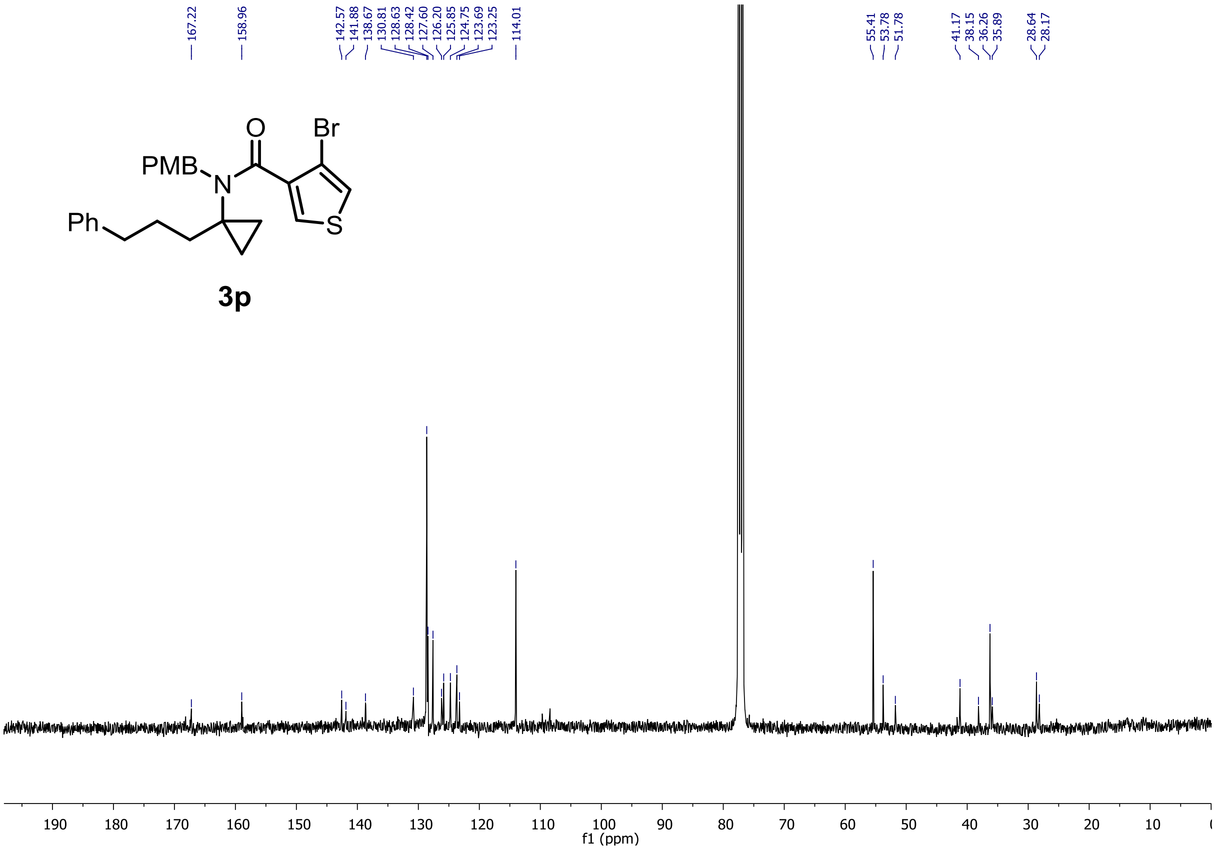
167.22
158.96
142.57
141.88
138.67
130.81
128.63
128.42
127.60
126.20
125.85
124.75
123.69
123.25
114.01
55.41
53.78
51.78
41.17
38.15
36.26
35.89
28.64
28.17
PMB
N
O
Br
S
Ph
3p
190 180 170 160 150 140 130 120 110 100 90 80 70 60 50 40 30 20 10
f1 (ppm)

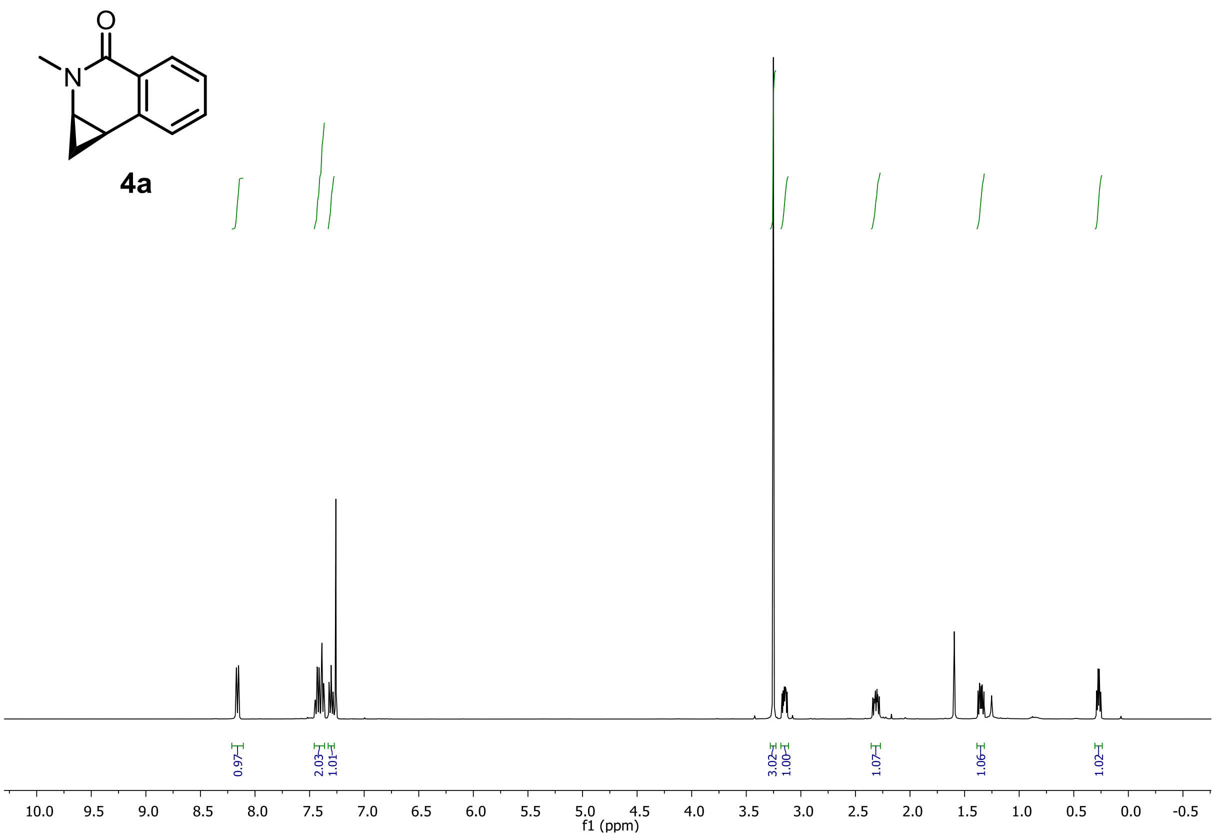
4a
0.97
2.03
1.01
3.02
1.00
1.07
1.06
1.02
10.0
9.5
9.0
8.5
8.0
7.5
7.0
6.5
6.0
5.5
5.0
4.5
4.0
3.5
3.0
2.5
2.0
1.5
1.0
0.5
0.0
-0.5
f1 (ppm)

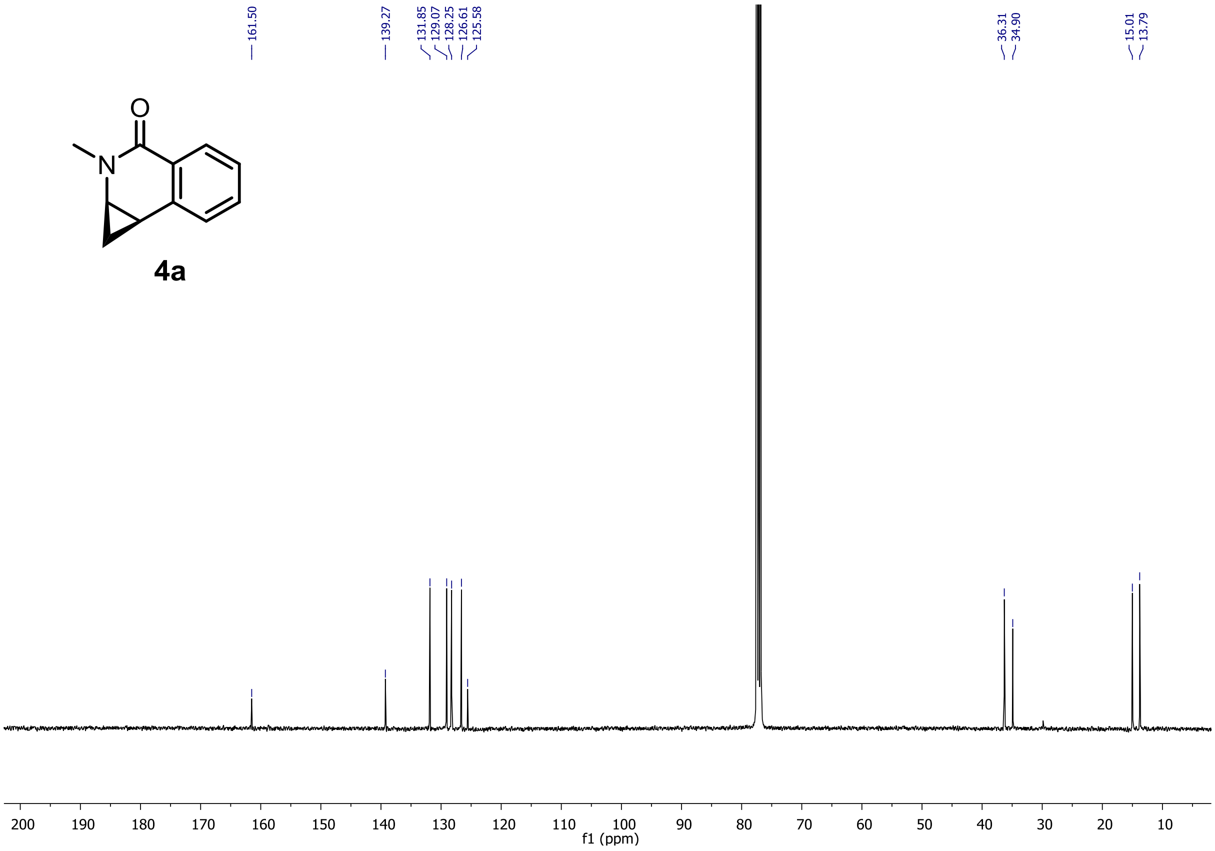
161.50
139.27
131.85
129.07
128.25
126.61
125.58
36.31
34.90
15.01
13.79
4a
200
190
180
170
160
150
140
130
120
110
100
90
80
70
60
50
40
30
20
10
f1 (ppm)

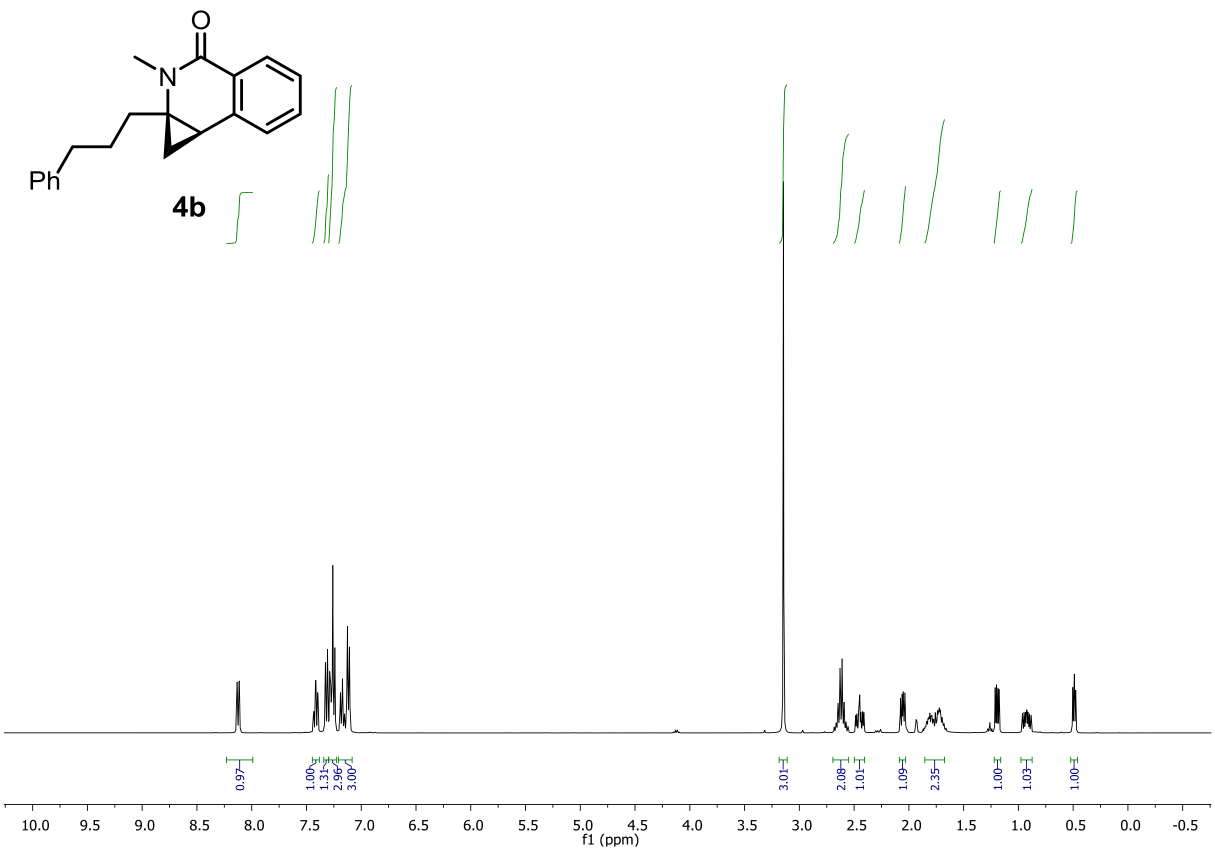
4b
0.97
1.00
1.31
2.96
3.00
3.01
2.08
1.01
1.09
2.35
1.00
1.03
1.00
f1 (ppm)

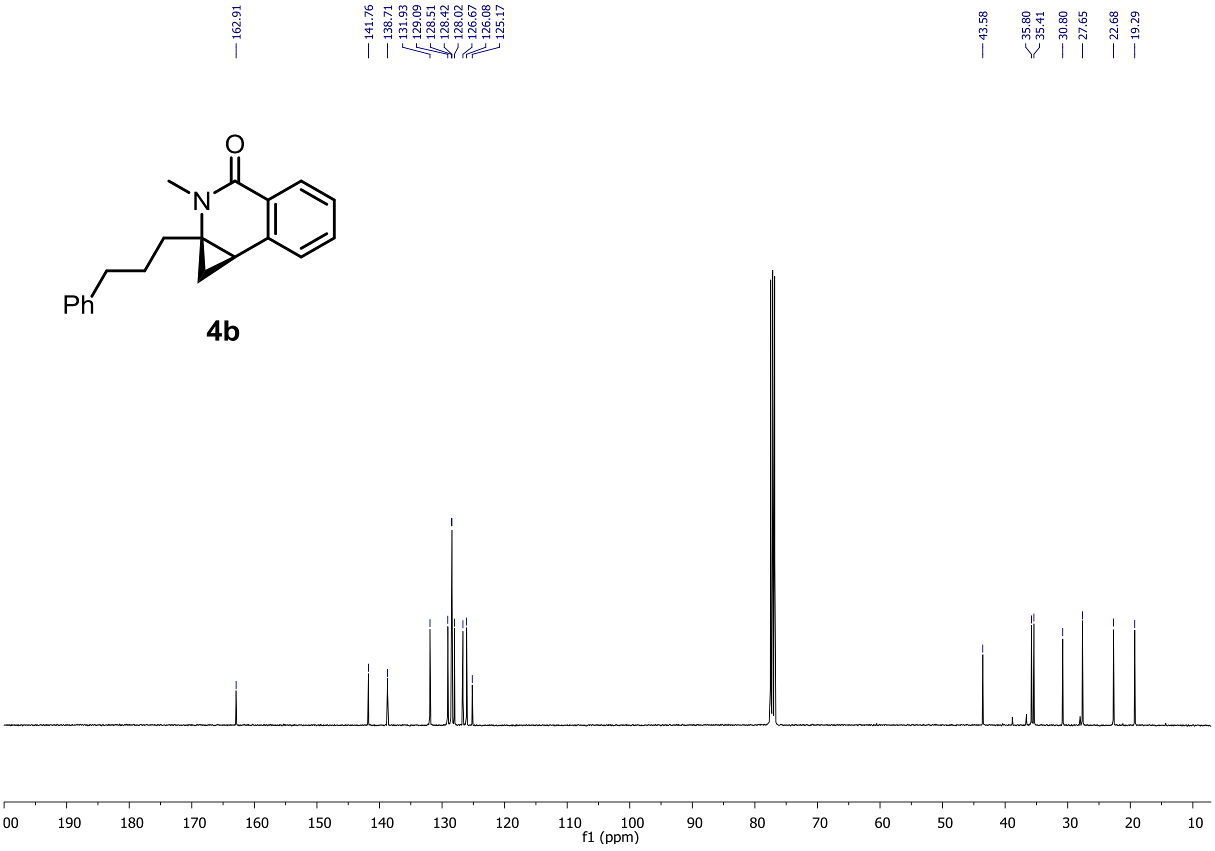
162.91
141.76
138.71
131.93
129.09
128.51
128.42
128.02
126.67
126.08
125.17
43.58
35.80
35.41
30.80
27.65
22.68
19.29
4b
f1 (ppm)

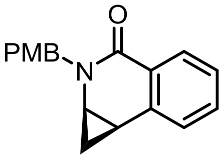
O
PMB
N

4c

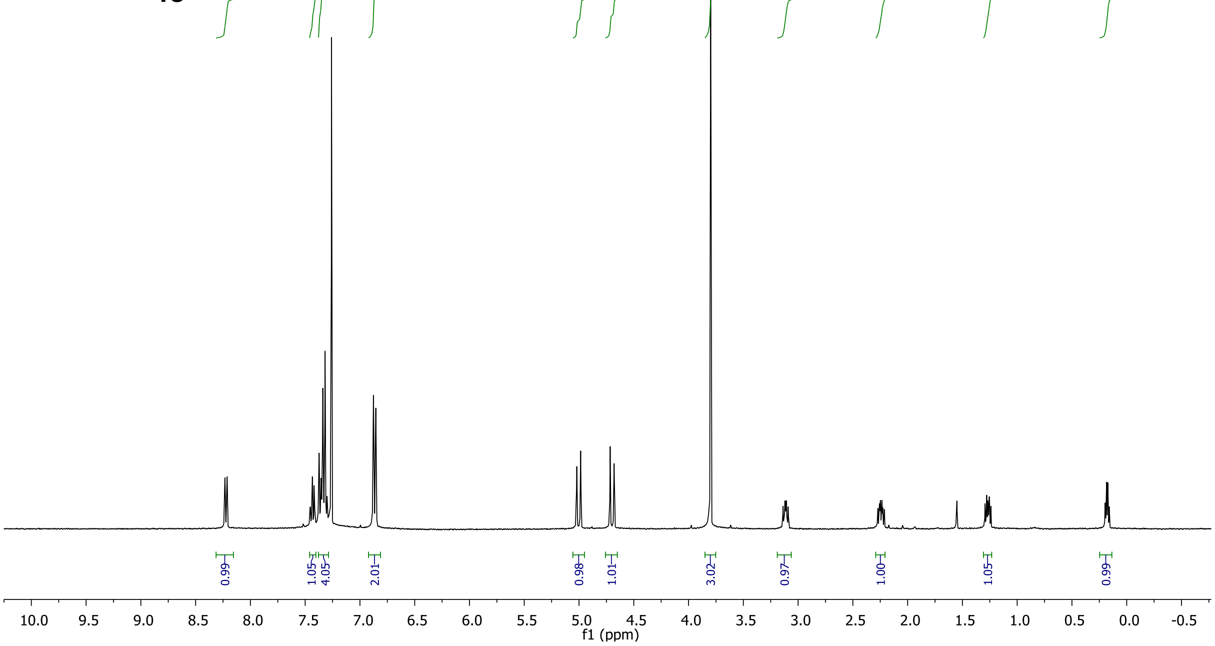
0.99
1.05
4.05
2.01
0.98
1.01
3.02
0.97
1.00
1.05
0.99
10.0 9.5 9.0 8.5 8.0 7.5 7.0 6.5 6.0 5.5 5.0 4.5 4.0 3.5 3.0 2.5 2.0 1.5 1.0 0.5 0.0 -0.5
f1 (ppm)

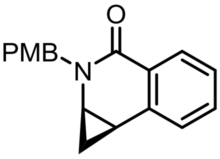
O
PMB
N

4c

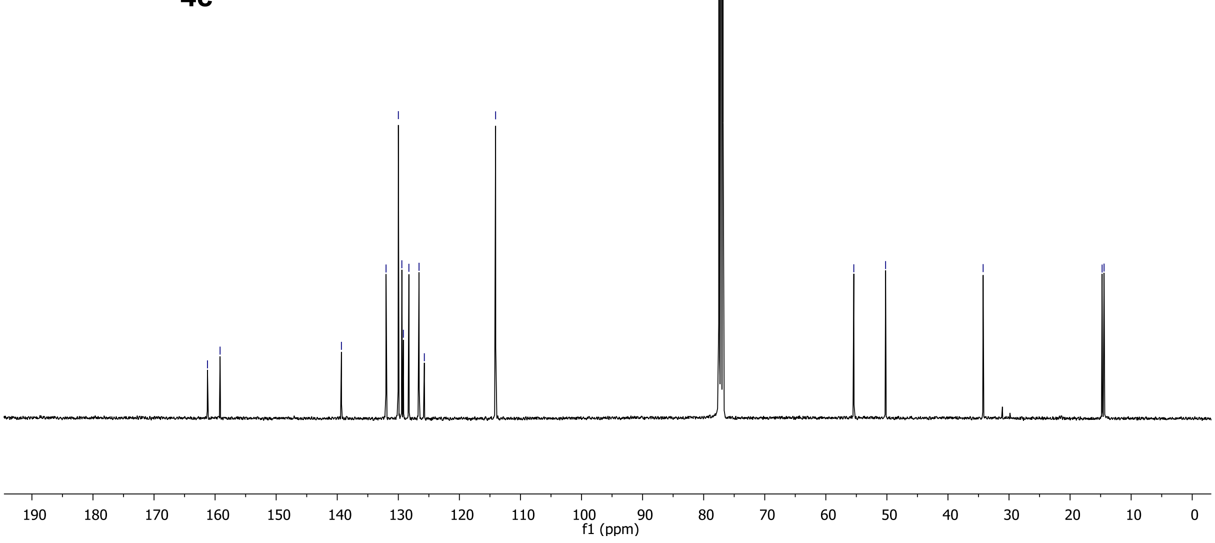
161.25
159.19
139.32
132.01
130.00
129.42
129.17
128.27
126.61
125.74
114.07
55.42
50.21
34.24
14.76
14.43
190 180 170 160 150 140 130 120 110 100 90 80 70 60 50 40 30 20 10 0
f1 (ppm)

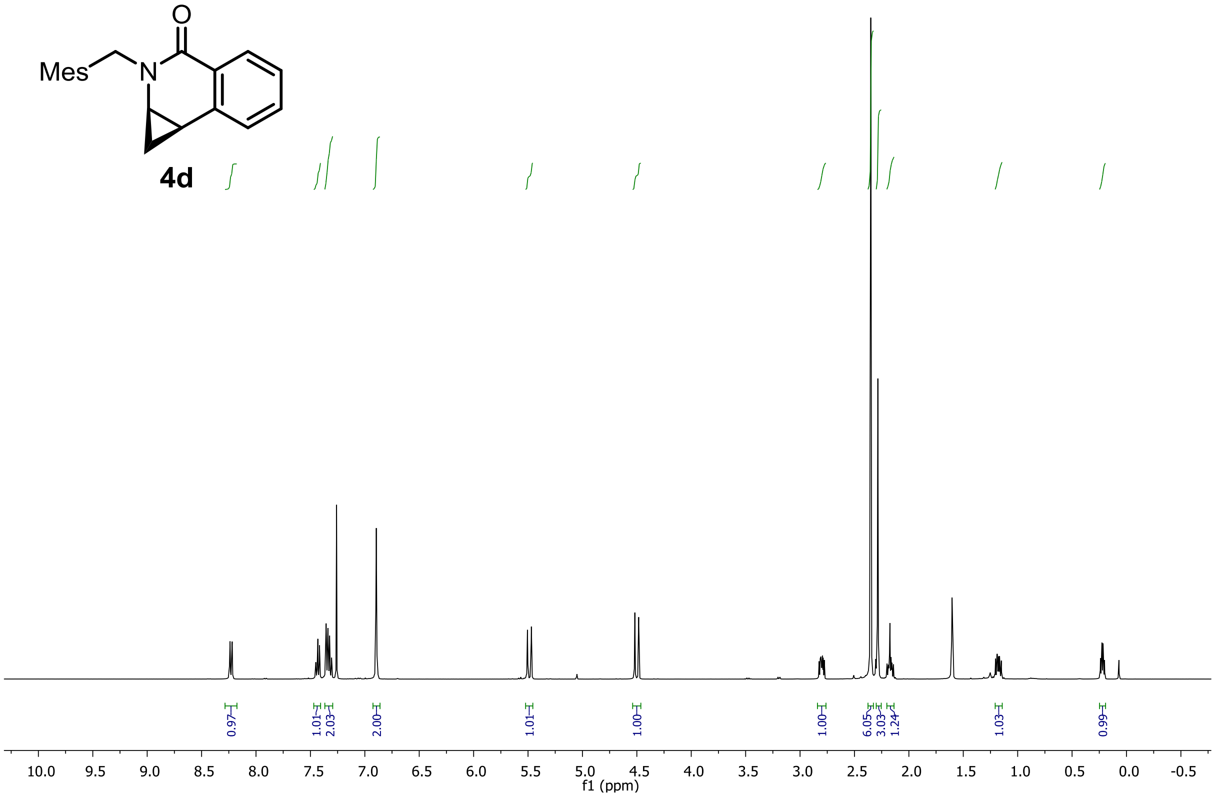
Mes
N
O
4d
0.97
1.01
2.03
2.00
1.01
1.00
1.00
6.05
3.03
1.24
1.03
0.99
10.0 9.5 9.0 8.5 8.0 7.5 7.0 6.5 6.0 5.5 5.0 4.5 4.0 3.5 3.0 2.5 2.0 1.5 1.0 0.5 0.0 -0.5
f1 (ppm)

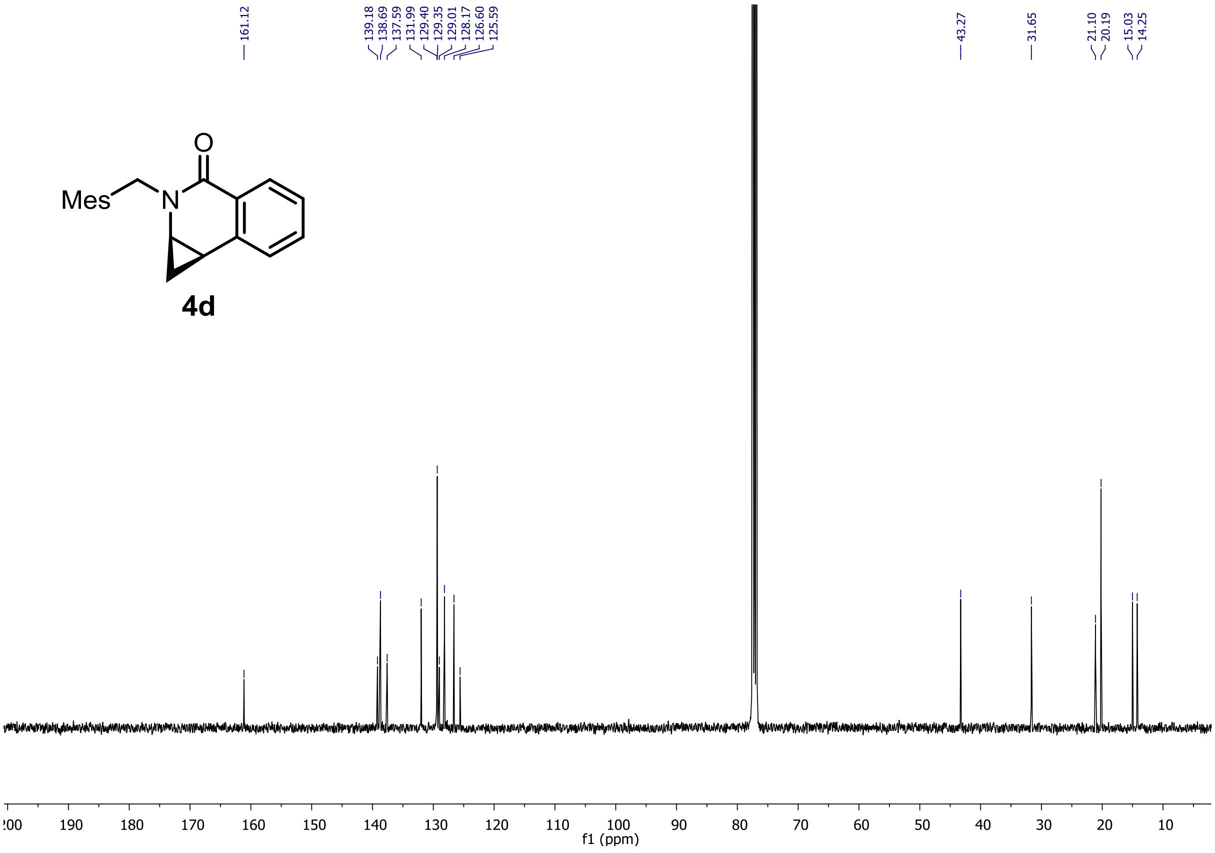
161.12
139.18
138.69
137.59
131.99
129.40
129.35
129.01
128.17
126.60
125.59
43.27
31.65
21.10
20.19
15.03
14.25
Mes
N
O
4d
200 190 180 170 160 150 140 130 120 110 100 90 80 70 60 50 40 30 20 10
f1 (ppm)

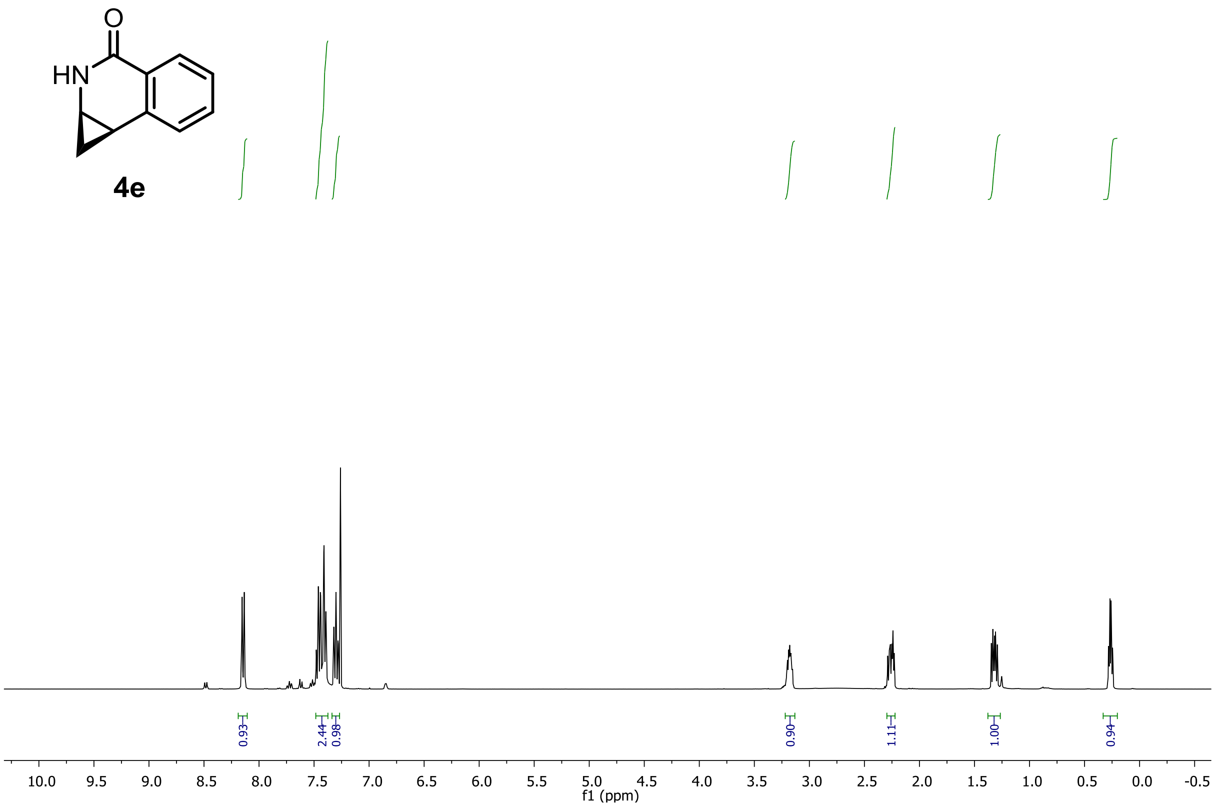

0.93
2.44
0.98
0.90
1.11
1.00
0.94
10.0 9.5 9.0 8.5 8.0 7.5 7.0 6.5 6.0 5.5 5.0 4.5 4.0 3.5 3.0 2.5 2.0 1.5 1.0 0.5 0.0 -0.5
f1 (ppm)

4e

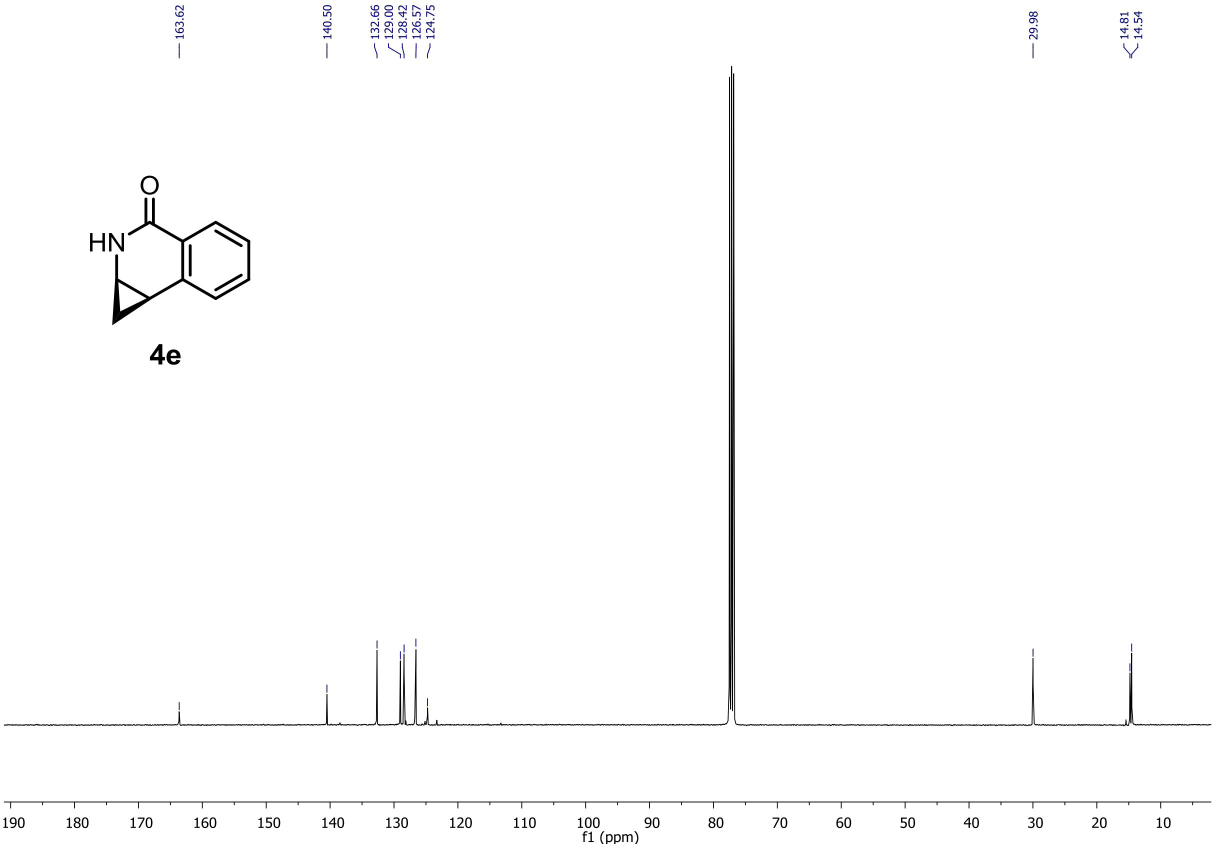

163.62
140.50
132.66
129.00
128.42
126.57
124.75
29.98
14.81
14.54
190 180 170 160 150 140 130 120 110 100 90 80 70 60 50 40 30 20 10
f1 (ppm)

4e

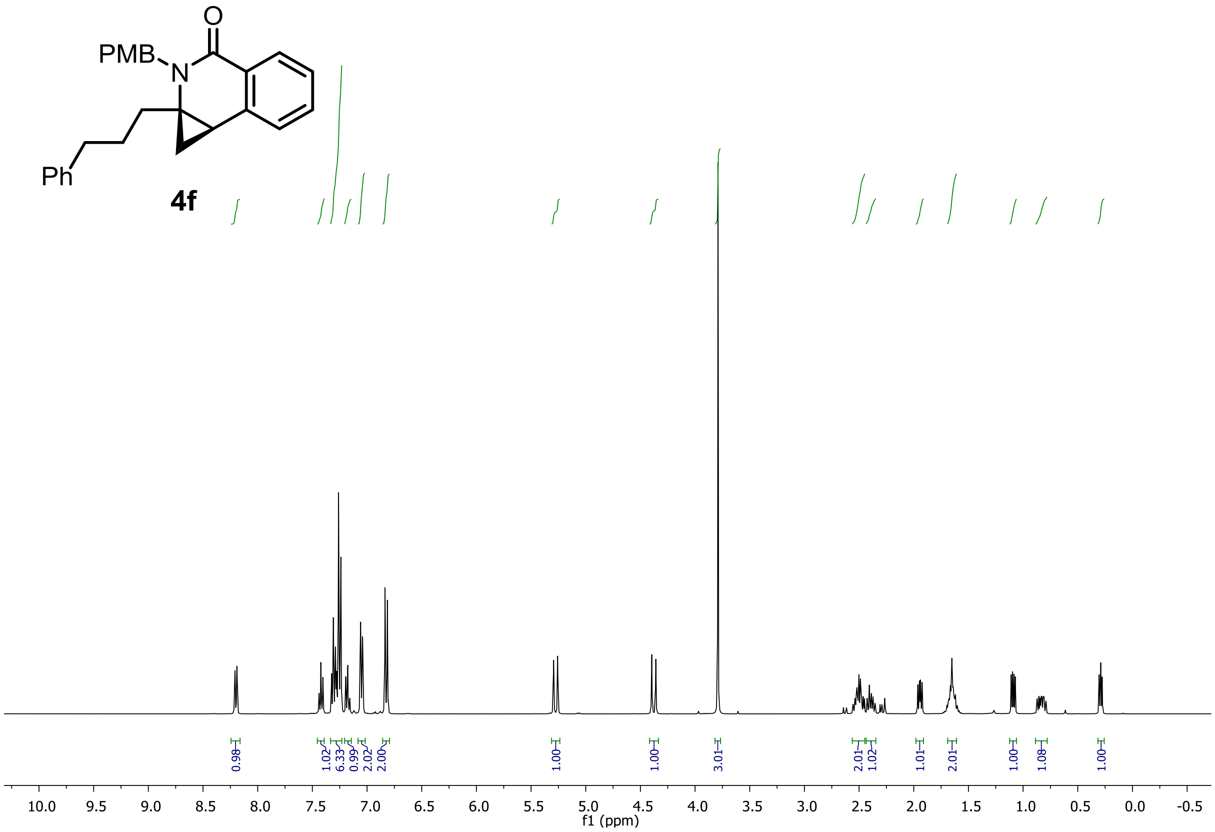
PMB
N
O
Ph
4f
0.98
1.02
6.33
0.99
2.02
2.00
1.00
1.00
3.01
2.01
1.02
1.01
2.01
1.00
1.08
1.00
f1 (ppm)

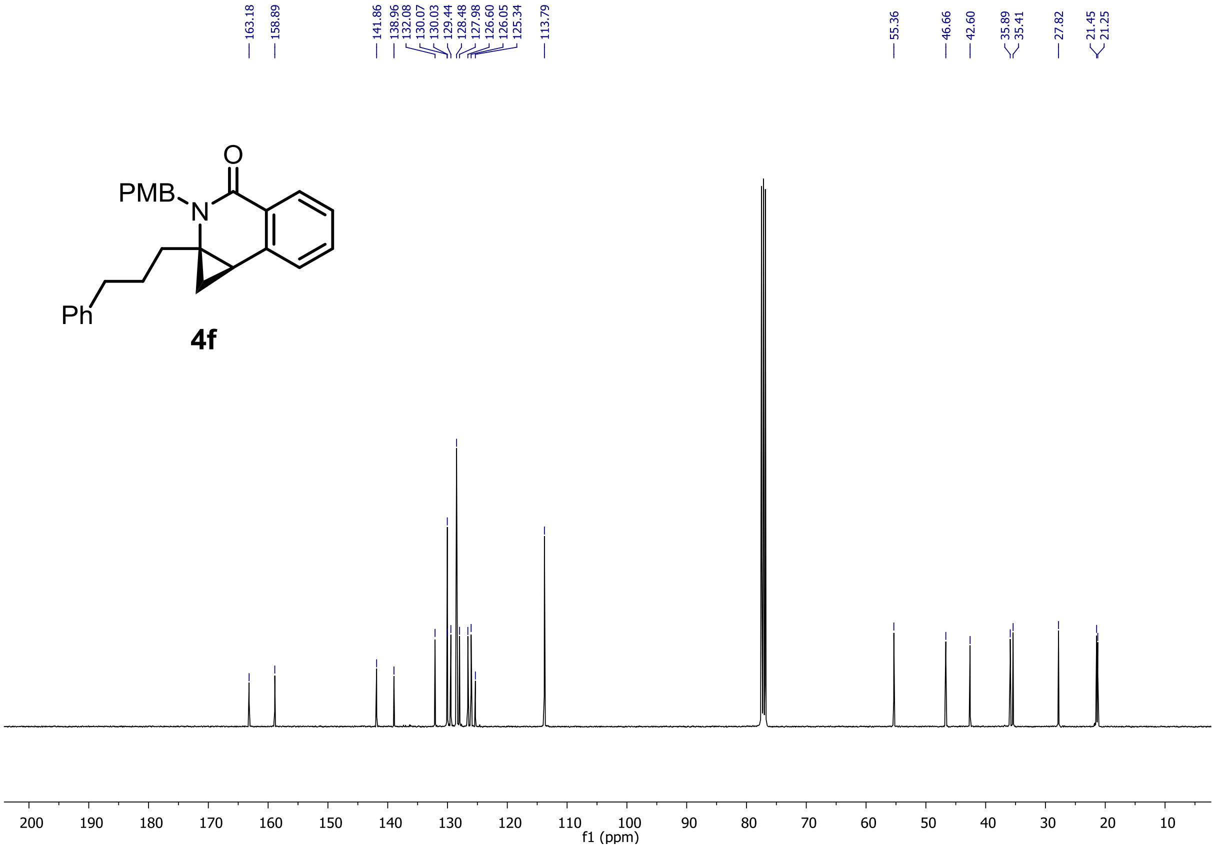
163.18
158.89
141.86
138.96
132.08
130.07
130.03
129.44
128.48
127.98
126.60
126.05
125.34
113.79
55.36
46.66
42.60
35.89
35.41
27.82
21.45
21.25
PMB
N
O
Ph
4f
f1 (ppm)

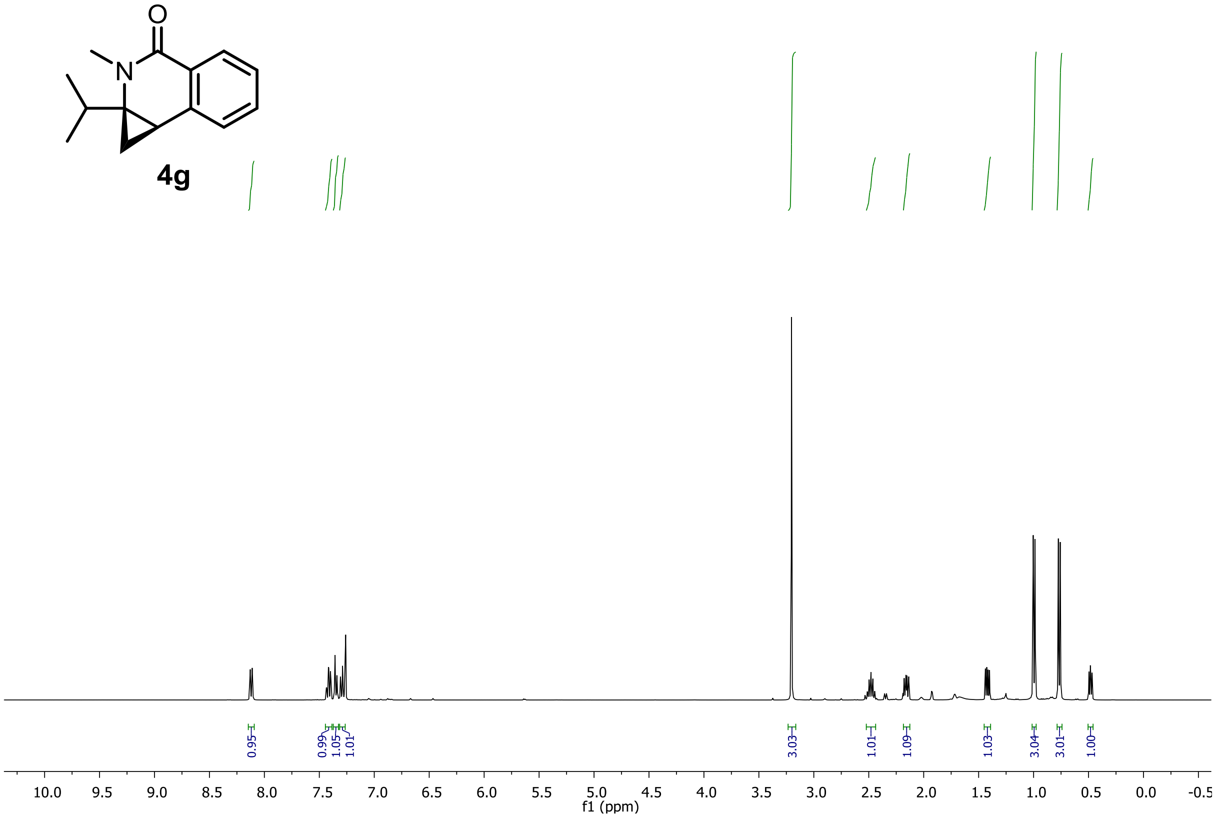

4g
0.95
0.99
1.05
1.01
3.03
1.01
1.09
1.03
3.04
3.01
1.00
10.0 9.5 9.0 8.5 8.0 7.5 7.0 6.5 6.0 5.5 5.0 4.5 4.0 3.5 3.0 2.5 2.0 1.5 1.0 0.5 0.0 -0.5
f1 (ppm)

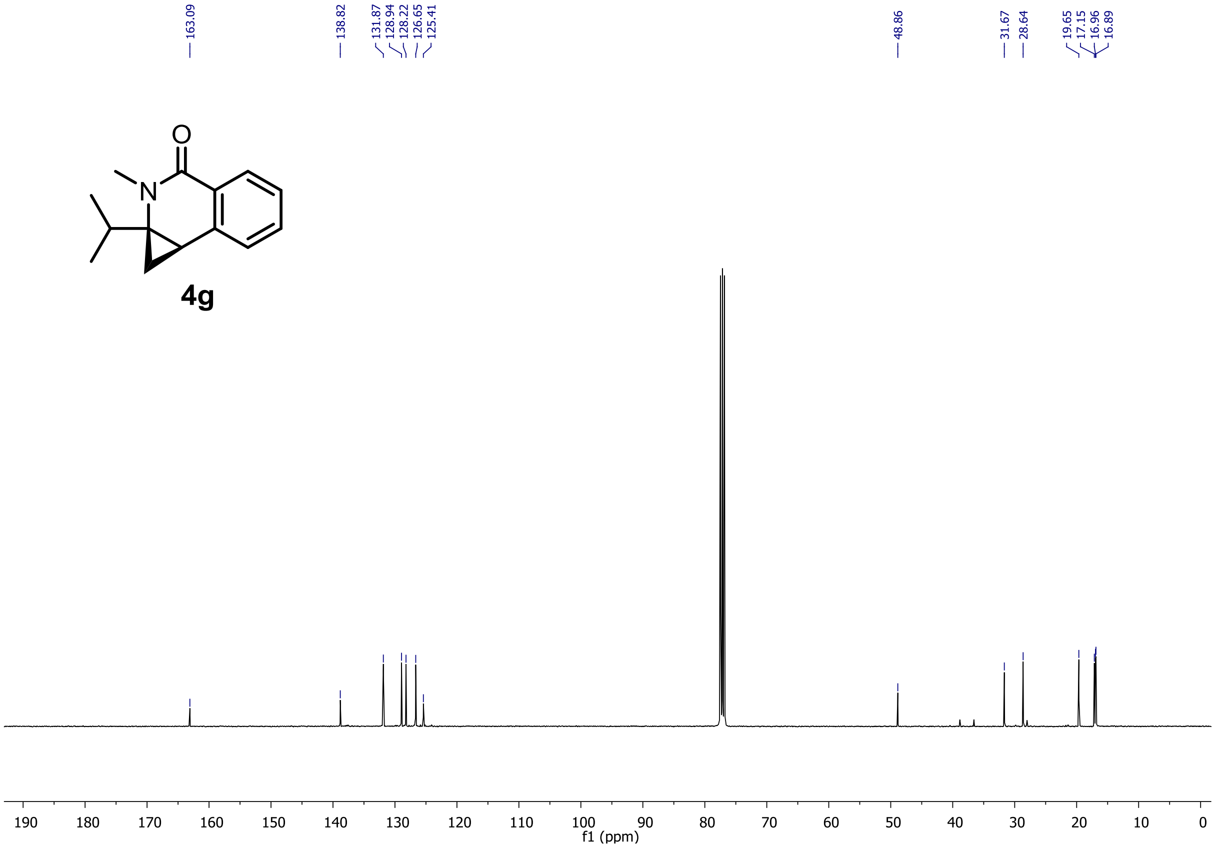

4g
163.09
138.82
131.87
128.94
128.22
126.65
125.41
48.86
31.67
28.64
19.65
17.15
16.96
16.89
190 180 170 160 150 140 130 120 110 100 90 80 70 60 50 40 30 20 10 0
f1 (ppm)

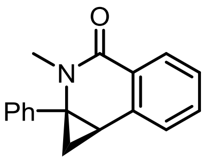
O
N
Ph

4h

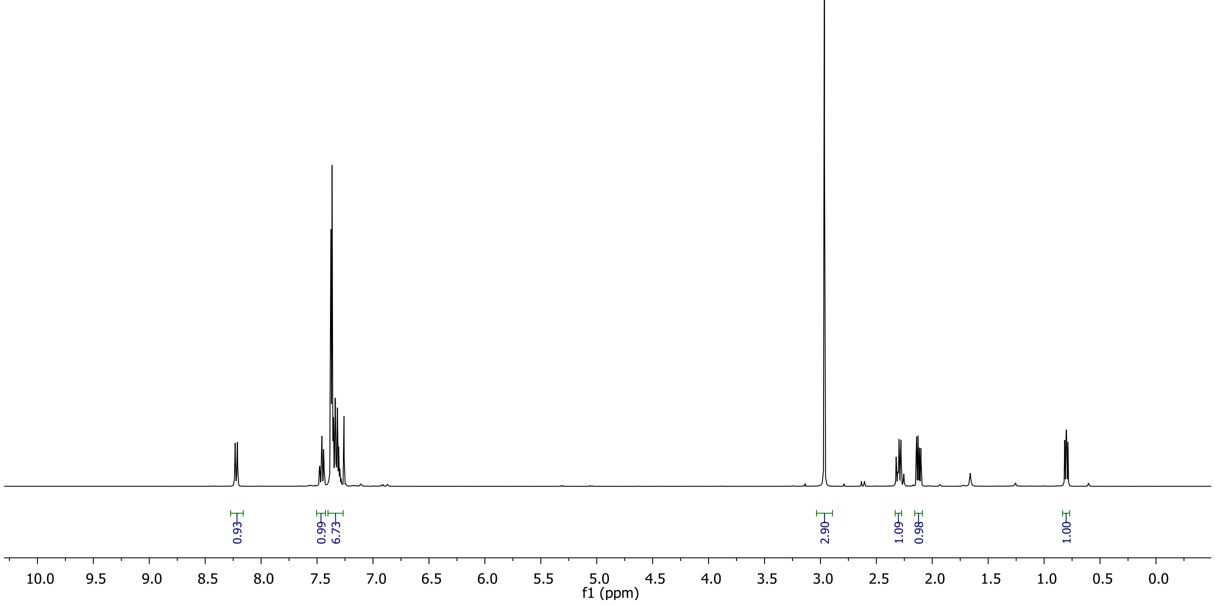
0.93
0.99
6.73
2.90
1.09
0.98
1.00
10.0 9.5 9.0 8.5 8.0 7.5 7.0 6.5 6.0 5.5 5.0 4.5 4.0 3.5 3.0 2.5 2.0 1.5 1.0 0.5 0.0
f1 (ppm)

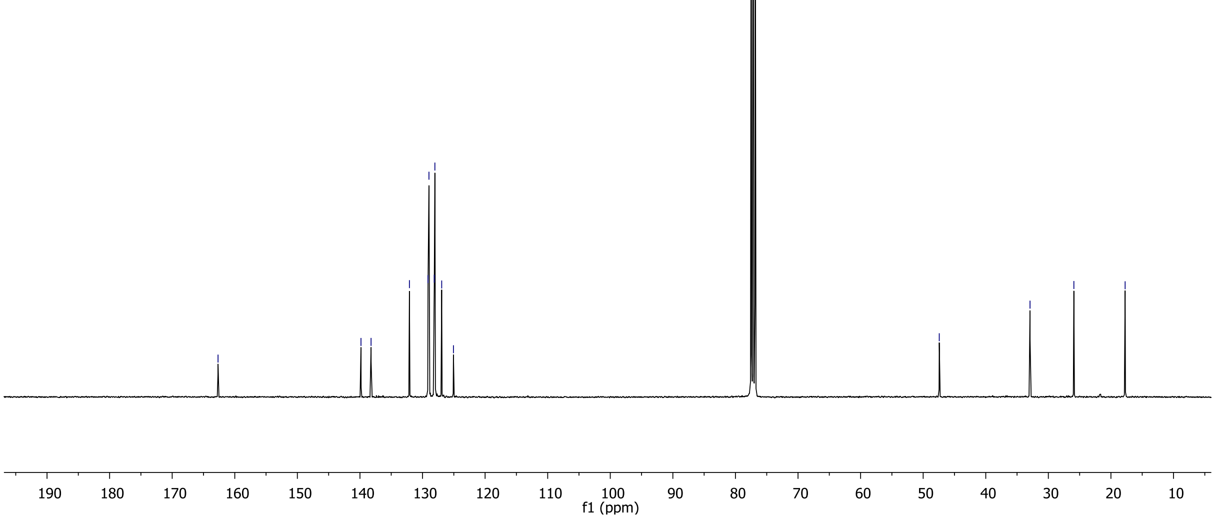
162.67
139.83
138.22
132.09
129.12
128.97
128.12
128.03
126.94
125.06
47.43
32.93
25.92
17.73
190 180 170 160 150 140 130 120 110 100 90 80 70 60 50 40 30 20 10
f1 (ppm)

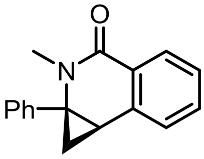
O
N
Ph

4h

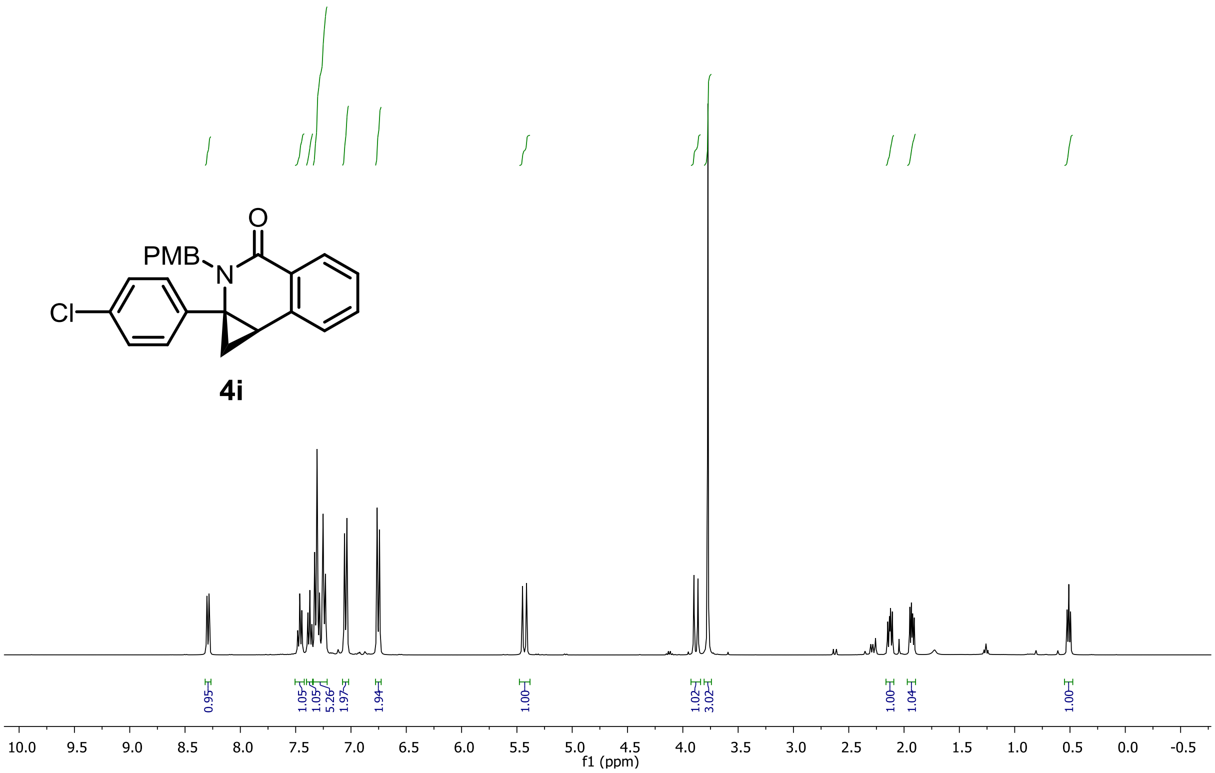
PMB
N
O
Cl
4i
0.95
1.05
1.05
5.26
1.97
1.94
1.00
1.02
3.02
1.00
1.04
1.00
10.0 9.5 9.0 8.5 8.0 7.5 7.0 6.5 6.0 5.5 5.0 4.5 4.0 3.5 3.0 2.5 2.0 1.5 1.0 0.5 0.0 -0.5
f1 (ppm)

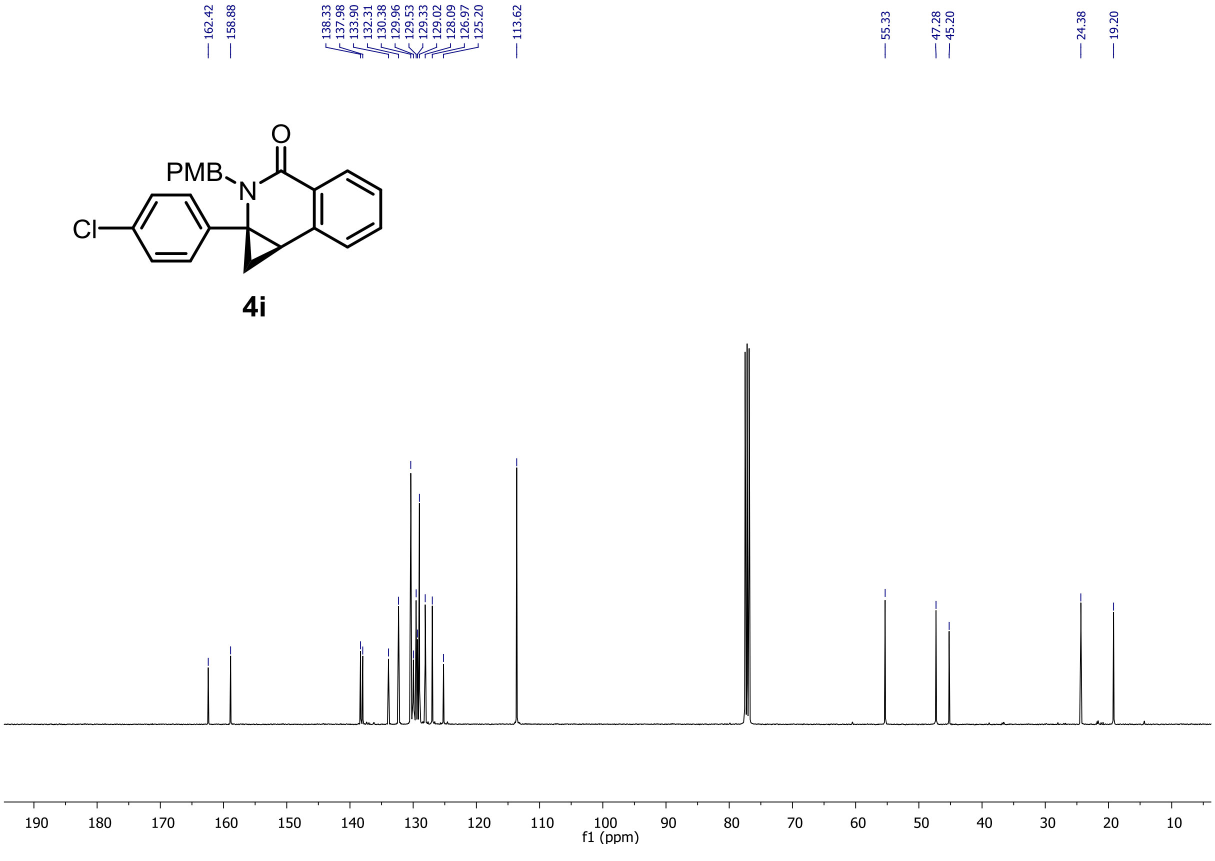
162.42
158.88
138.33
137.98
133.90
132.31
130.38
129.96
129.53
129.33
129.02
128.09
126.97
125.20
113.62
55.33
47.28
45.20
24.38
19.20
PMB
N
O
Cl
4i
190 180 170 160 150 140 130 120 110 100 90 80 70 60 50 40 30 20 10
f1 (ppm)

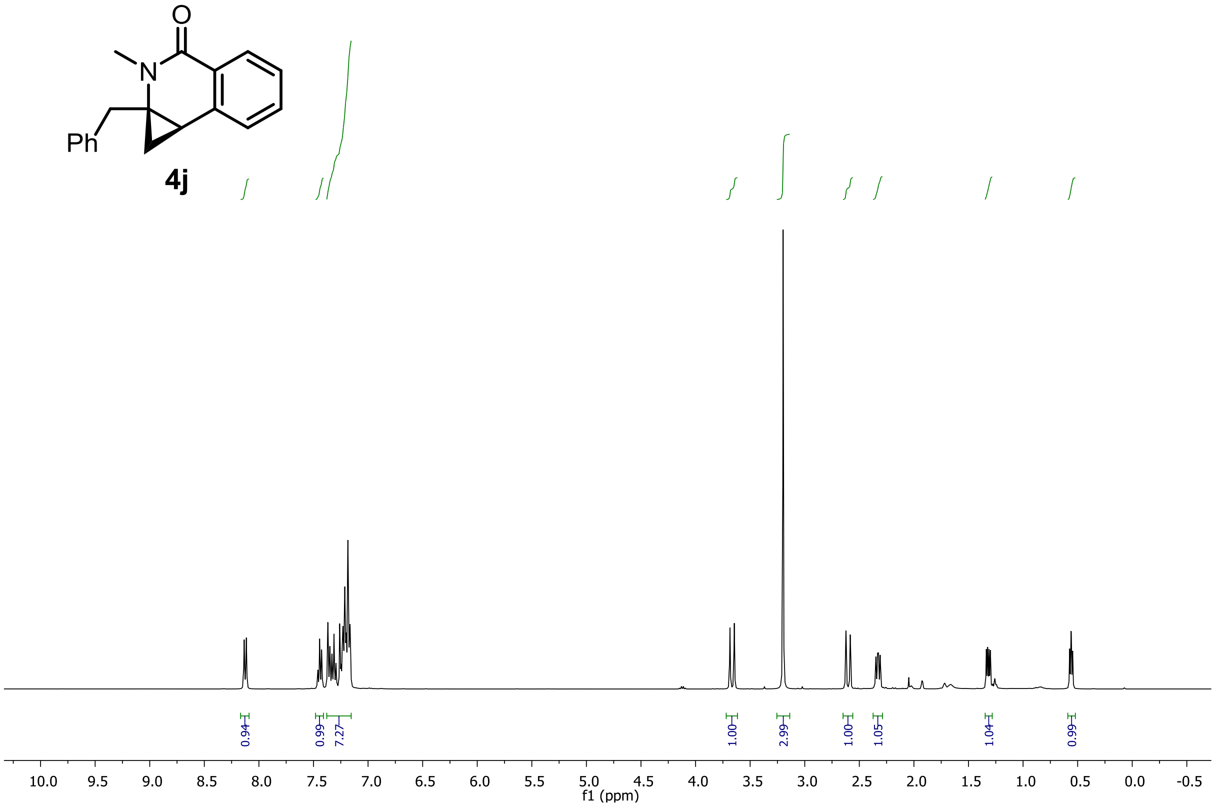
Ph
4j
0.94
0.99
7.27
1.00
2.99
1.00
1.05
1.04
0.99
10.0 9.5 9.0 8.5 8.0 7.5 7.0 6.5 6.0 5.5 5.0 4.5 4.0 3.5 3.0 2.5 2.0 1.5 1.0 0.5 0.0 -0.5
f1 (ppm)

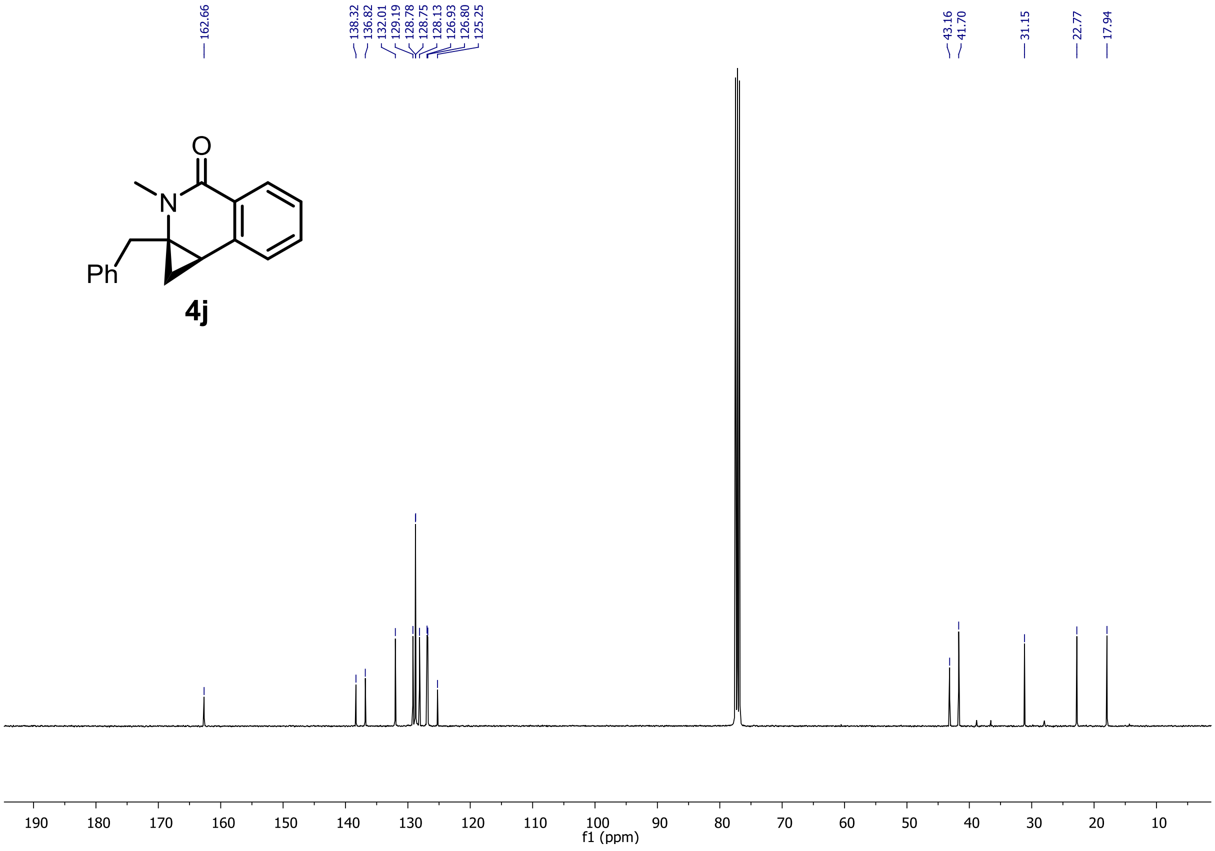
162.66
138.32
136.82
132.01
129.19
128.78
128.75
128.13
126.93
126.80
125.25
43.16
41.70
31.15
22.77
17.94
Ph
4j
190 180 170 160 150 140 130 120 110 100 90 80 70 60 50 40 30 20 10
f1 (ppm)

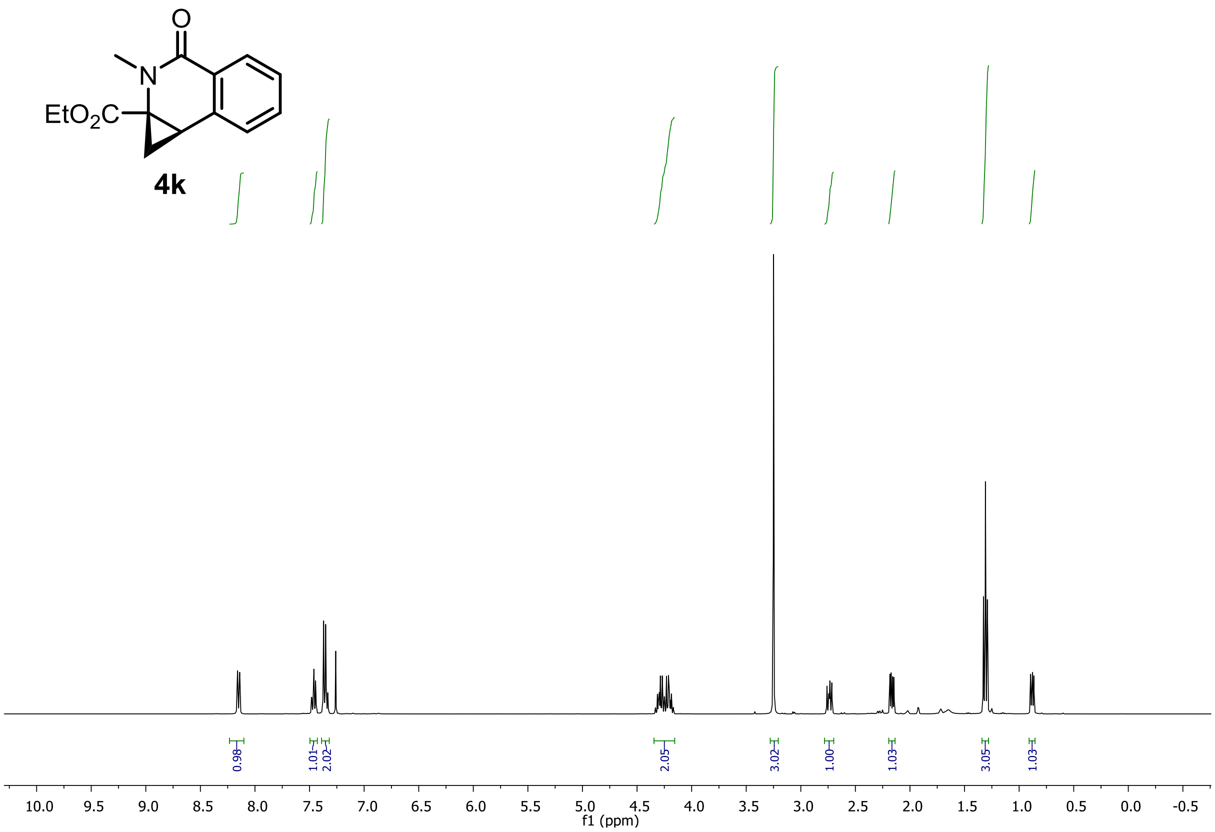
O
N
EtO2C
4k
0.98
1.01
2.02
2.05
3.02
1.00
1.03
3.05
1.03
f1 (ppm)

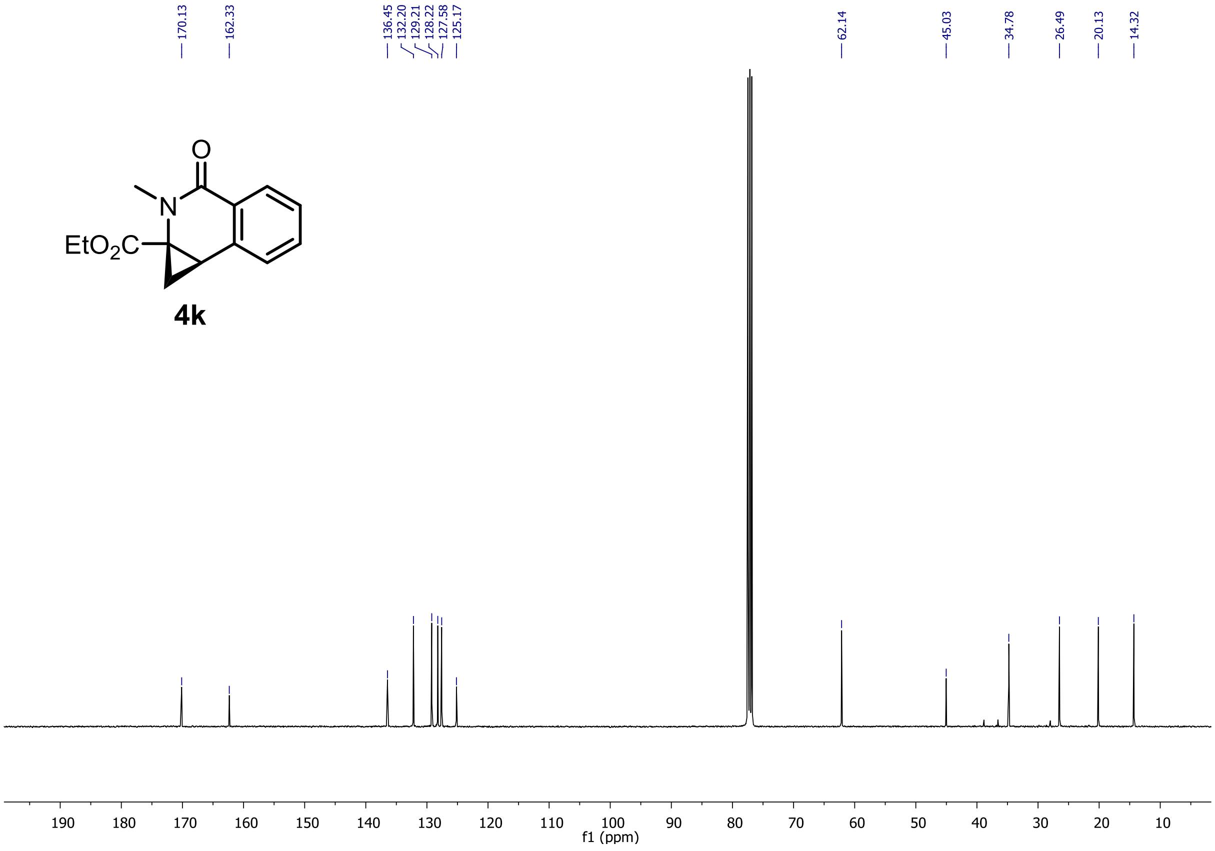
170.13
162.33
136.45
132.20
129.21
128.22
127.58
125.17
62.14
45.03
34.78
26.49
20.13
14.32
O
N
EtO2C
4k
f1 (ppm)

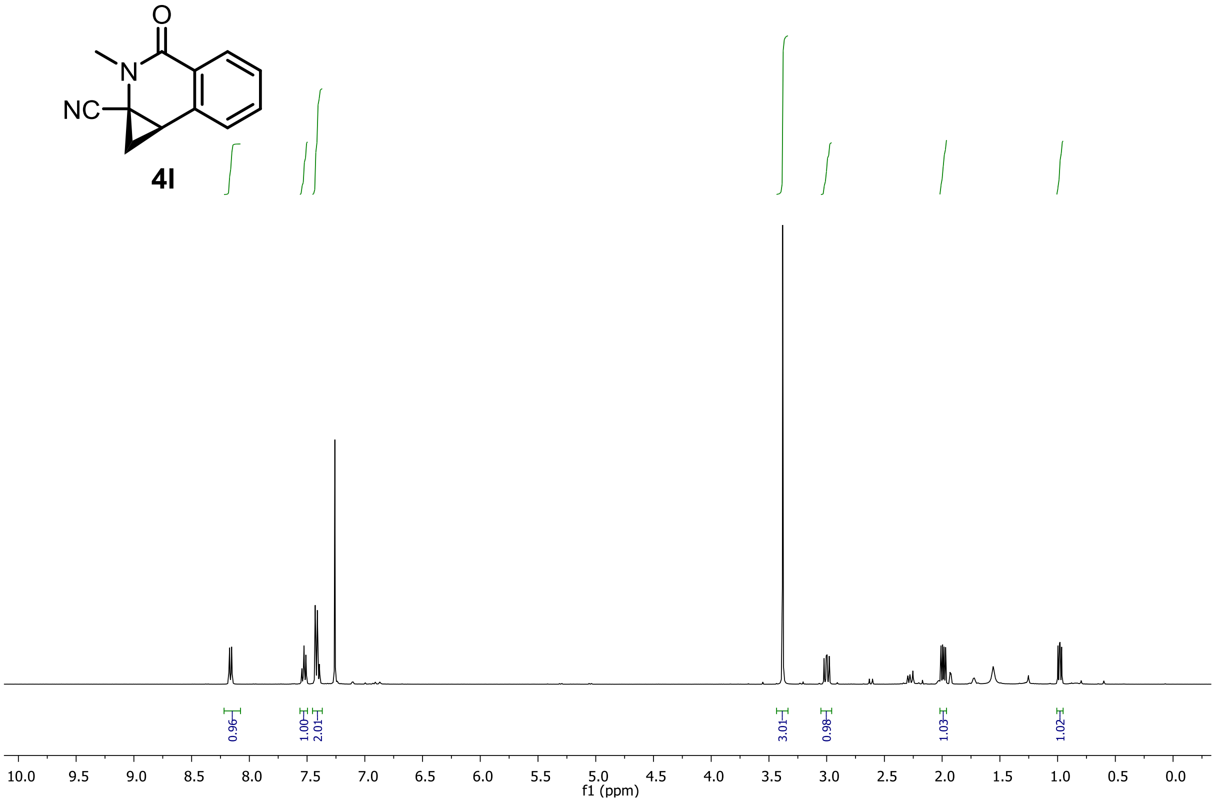

O
N
NC
4l
0.96
1.00
2.01
3.01
0.98
1.03
1.02
10.0 9.5 9.0 8.5 8.0 7.5 7.0 6.5 6.0 5.5 5.0 4.5 4.0 3.5 3.0 2.5 2.0 1.5 1.0 0.5 0.0
f1 (ppm)

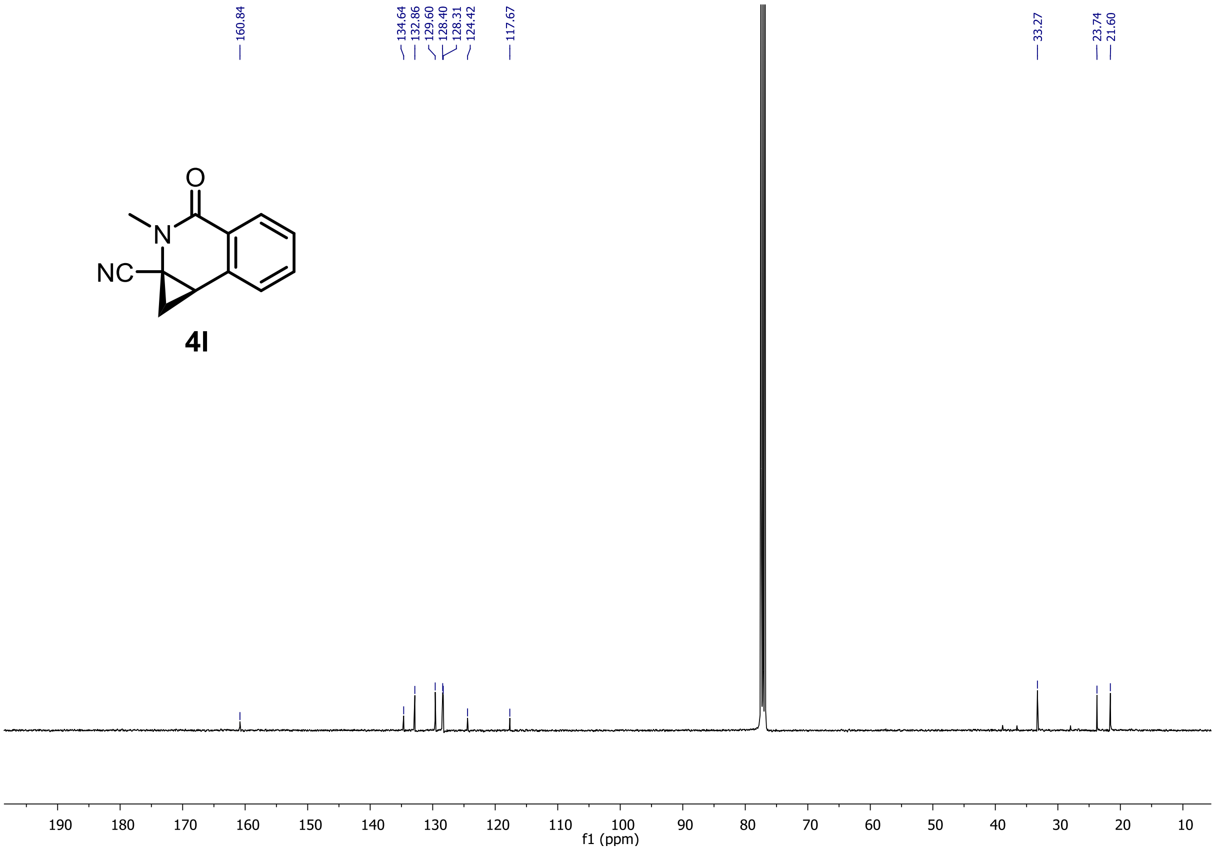

160.84
134.64
132.86
129.60
128.40
128.31
124.42
117.67
33.27
23.74
21.60
O
N
NC
4l
190 180 170 160 150 140 130 120 110 100 90 80 70 60 50 40 30 20 10
f1 (ppm)

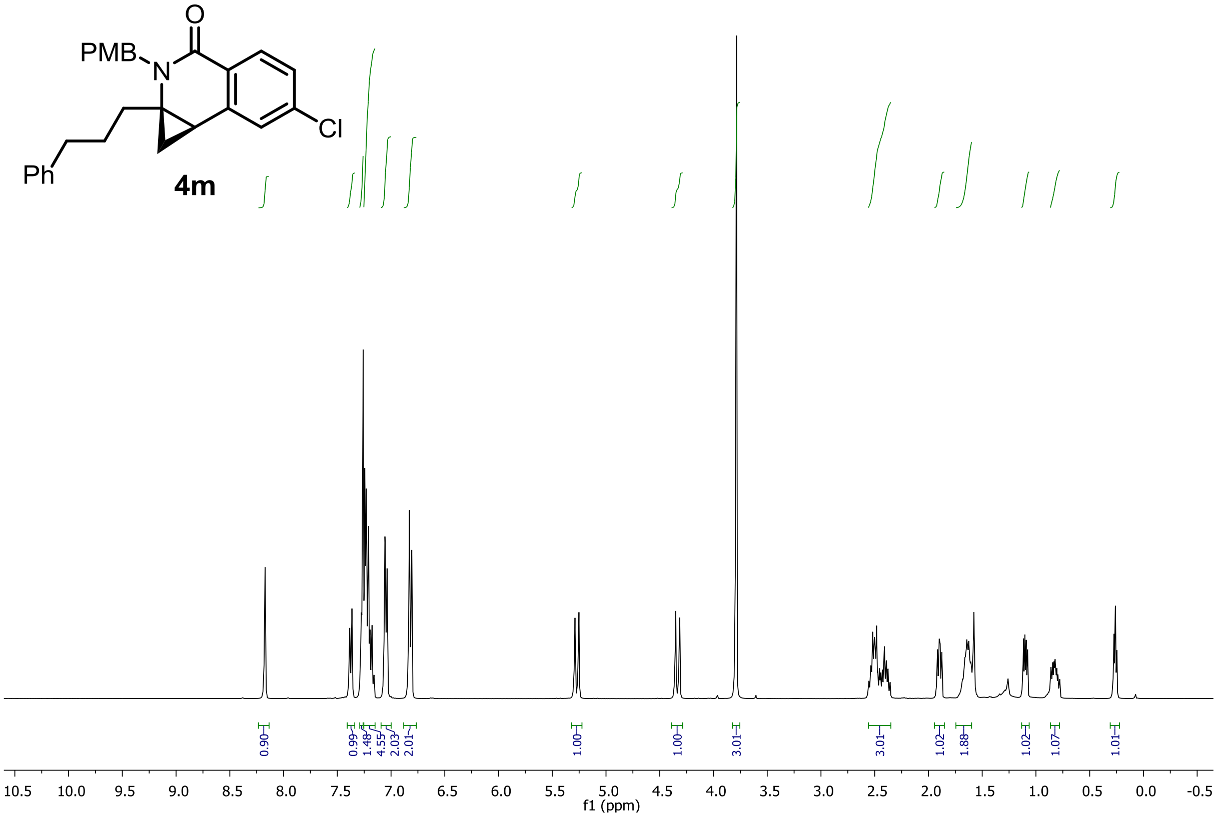
PMB
N
O
Cl
Ph
4m
0.90
0.99
1.48
4.55
2.03
2.01
1.00
1.00
3.01
3.01
1.02
1.88
1.02
1.07
1.01
10.5 10.0 9.5 9.0 8.5 8.0 7.5 7.0 6.5 6.0 5.5 5.0 4.5 4.0 3.5 3.0 2.5 2.0 1.5 1.0 0.5 0.0 -0.5
f1 (ppm)

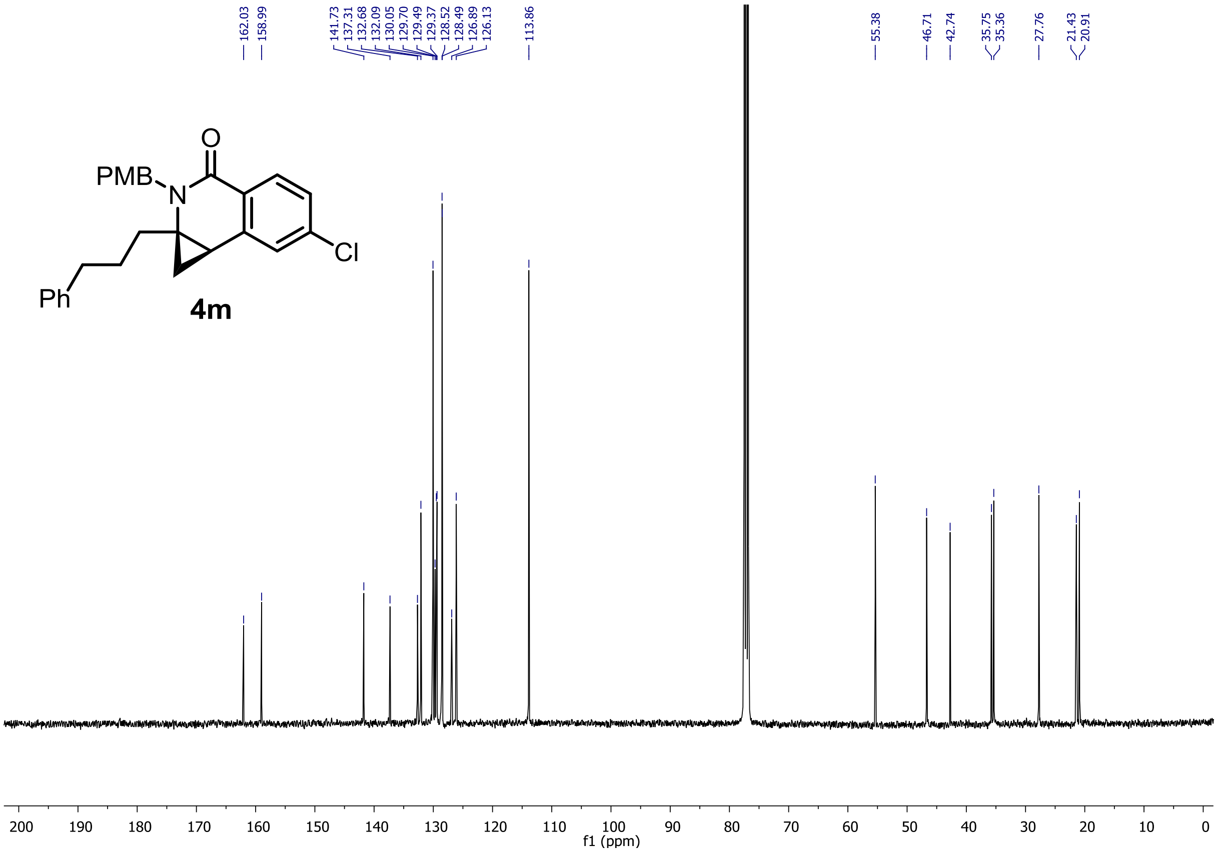
162.03
158.99
141.73
137.31
132.68
132.09
130.05
129.70
129.49
129.37
128.52
128.49
126.89
126.13
113.86
55.38
46.71
42.74
35.75
35.36
27.76
21.43
20.91
PMB
N
O
Cl
Ph
4m
200 190 180 170 160 150 140 130 120 110 100 90 80 70 60 50 40 30 20 10 0
f1 (ppm)

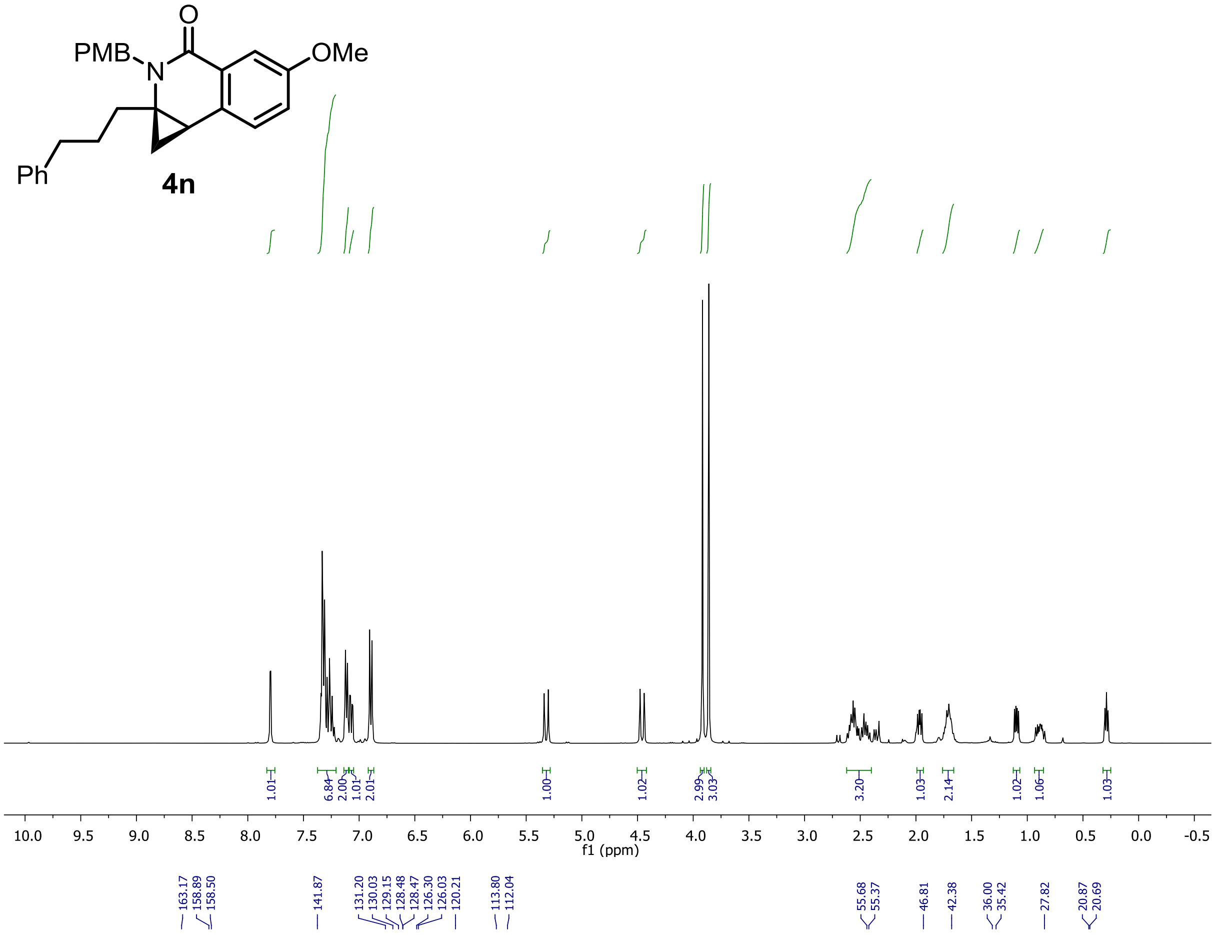
PMB
N
O
OMe
Ph
4n
1.01
6.84
2.00
1.01
2.01
1.00
1.02
2.99
3.03
3.20
1.03
2.14
1.02
1.06
1.03
10.0 9.5 9.0 8.5 8.0 7.5 7.0 6.5 6.0 5.5 5.0 4.5 4.0 3.5 3.0 2.5 2.0 1.5 1.0 0.5 0.0 -0.5
f1 (ppm)

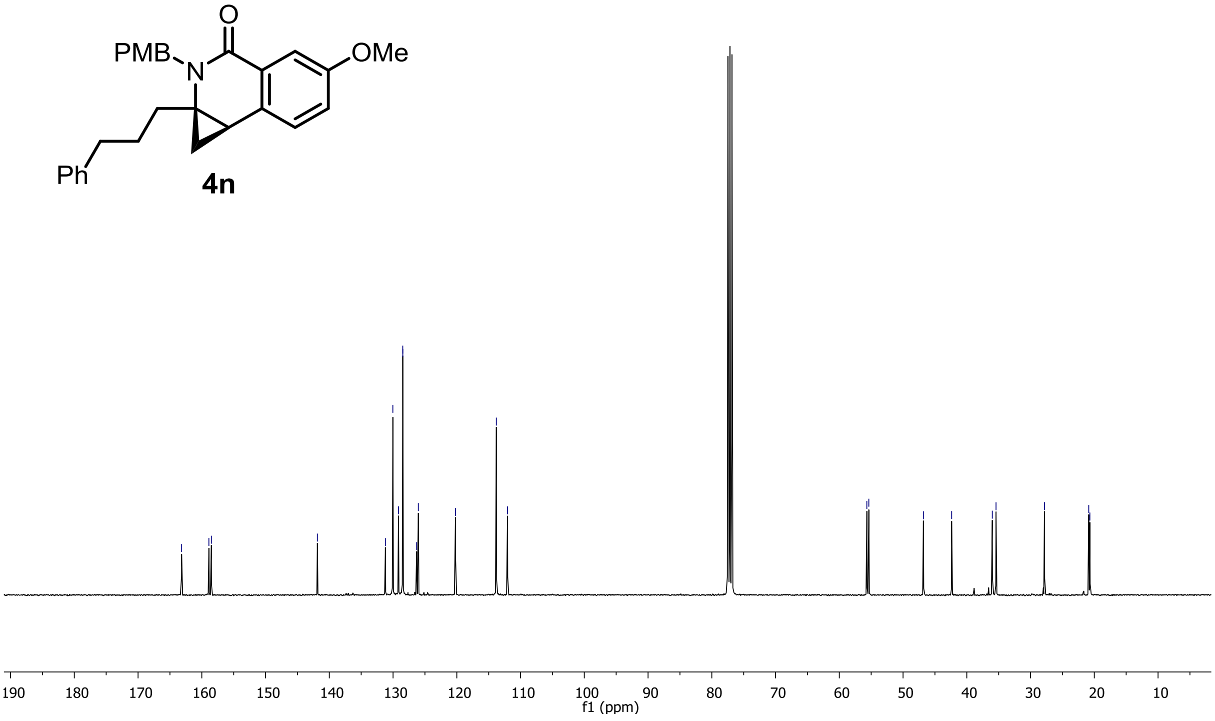
163.17
158.89
158.50
141.87
131.20
130.03
129.15
128.48
128.47
126.30
126.03
120.21
113.80
112.04
55.68
55.37
46.81
42.38
36.00
35.42
27.82
20.87
20.69
PMB
N
O
OMe
Ph
4n
190 180 170 160 150 140 130 120 110 100 90 80 70 60 50 40 30 20 10
f1 (ppm)

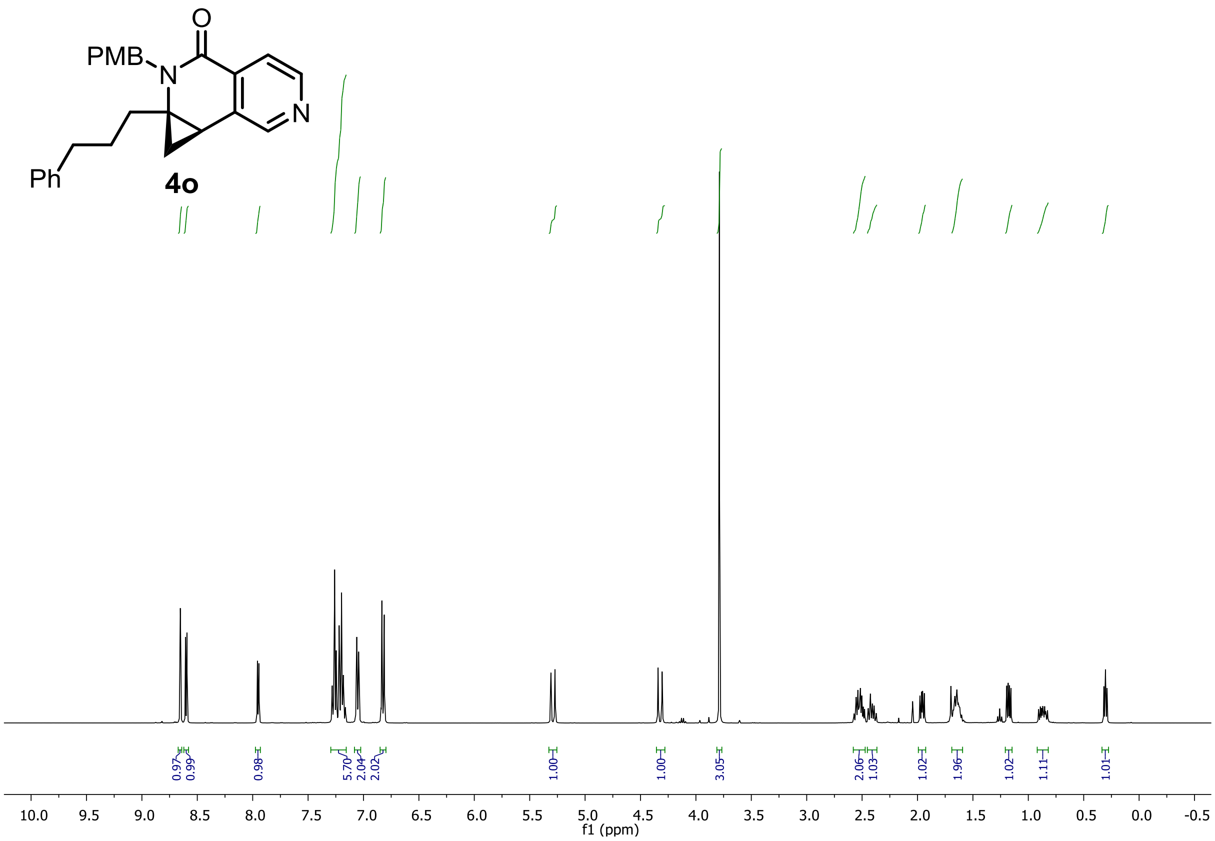

PMB
N
O
N
Ph
4o
0.97
0.99
0.98
5.70
2.04
2.02
1.00
1.00
3.05
2.06
1.03
1.02
1.96
1.02
1.11
1.01
10.0 9.5 9.0 8.5 8.0 7.5 7.0 6.5 6.0 5.5 5.0 4.5 4.0 3.5 3.0 2.5 2.0 1.5 1.0 0.5 0.0 -0.5
f1 (ppm)

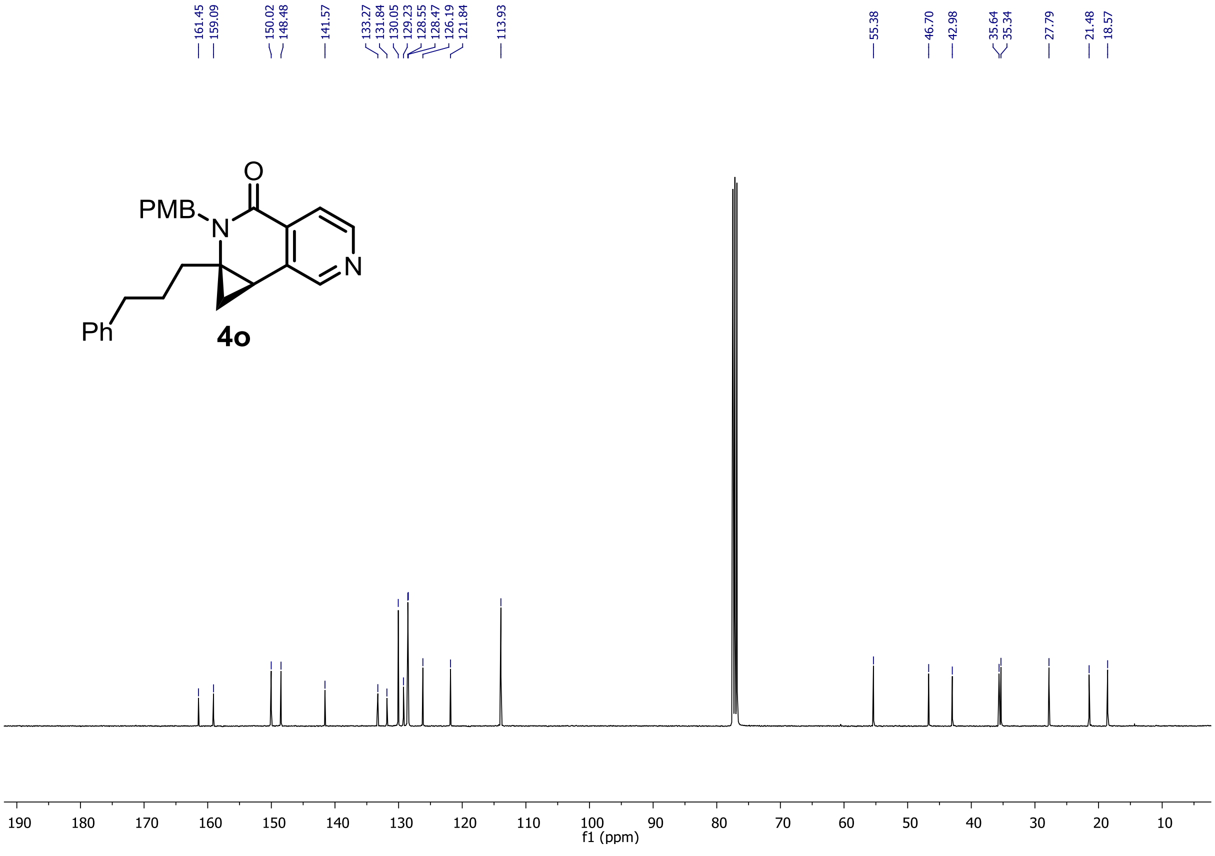

161.45
159.09
150.02
148.48
141.57
133.27
131.84
130.05
129.23
128.55
128.47
126.19
121.84
113.93
55.38
46.70
42.98
35.64
35.34
27.79
21.48
18.57
PMB
N
O
N
Ph
4o
190 180 170 160 150 140 130 120 110 100 90 80 70 60 50 40 30 20 10
f1 (ppm)

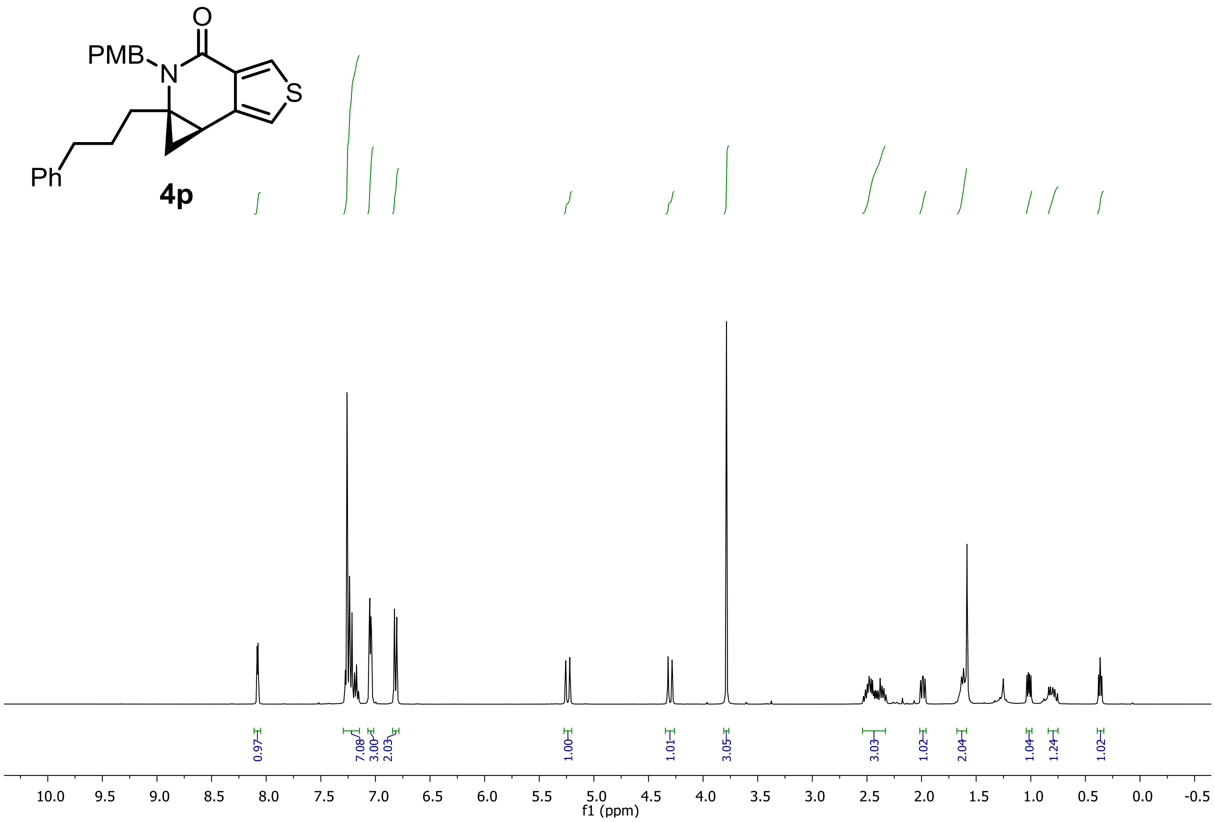

PMB
N
O
S
Ph
4p
0.97
7.08
3.00
2.03
1.00
1.01
3.05
3.03
1.02
2.04
1.04
1.24
1.02
10.0 9.5 9.0 8.5 8.0 7.5 7.0 6.5 6.0 5.5 5.0 4.5 4.0 3.5 3.0 2.5 2.0 1.5 1.0 0.5 0.0 -0.5
f1 (ppm)

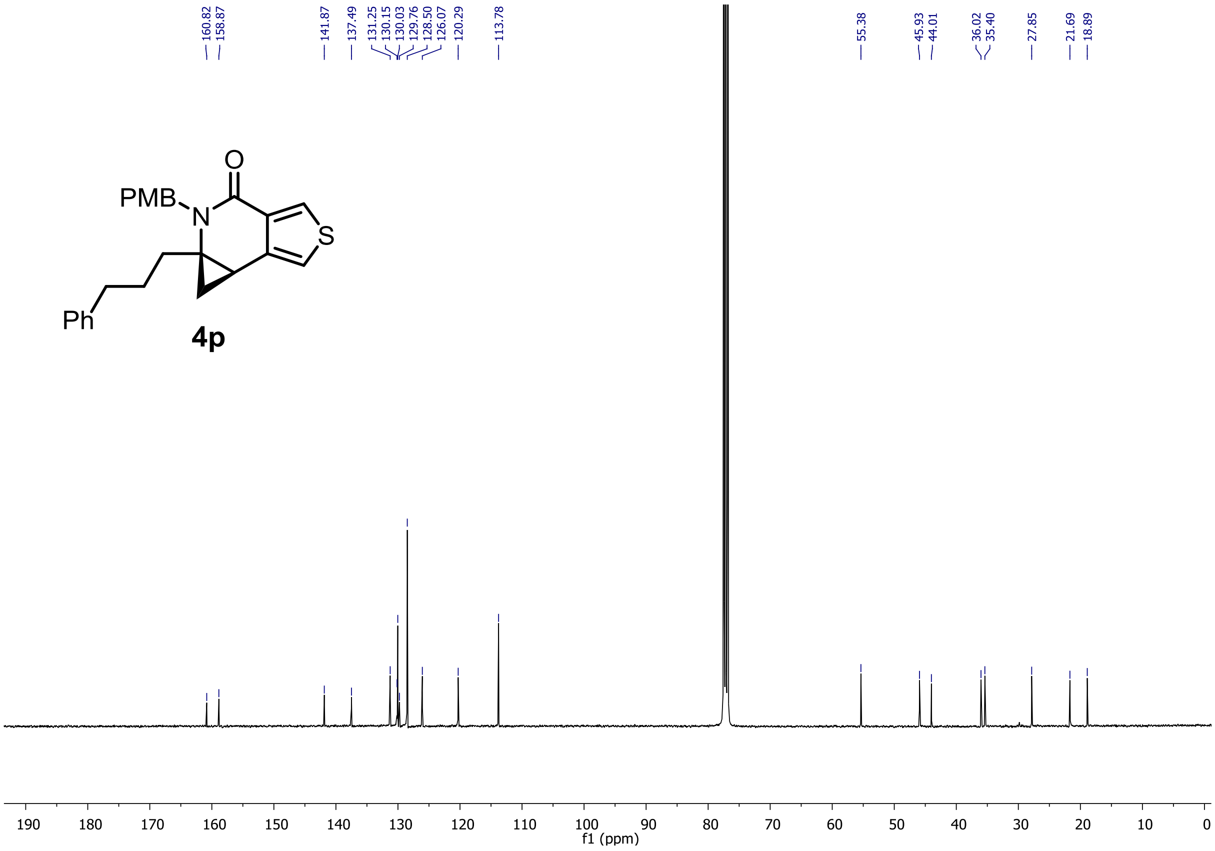

160.82
158.87
141.87
137.49
131.25
130.15
130.03
129.76
128.50
126.07
120.29
113.78
55.38
45.93
44.01
36.02
35.40
27.85
21.69
18.89
PMB
N
O
S
Ph
4p
190 180 170 160 150 140 130 120 110 100 90 80 70 60 50 40 30 20 10 0
f1 (ppm)

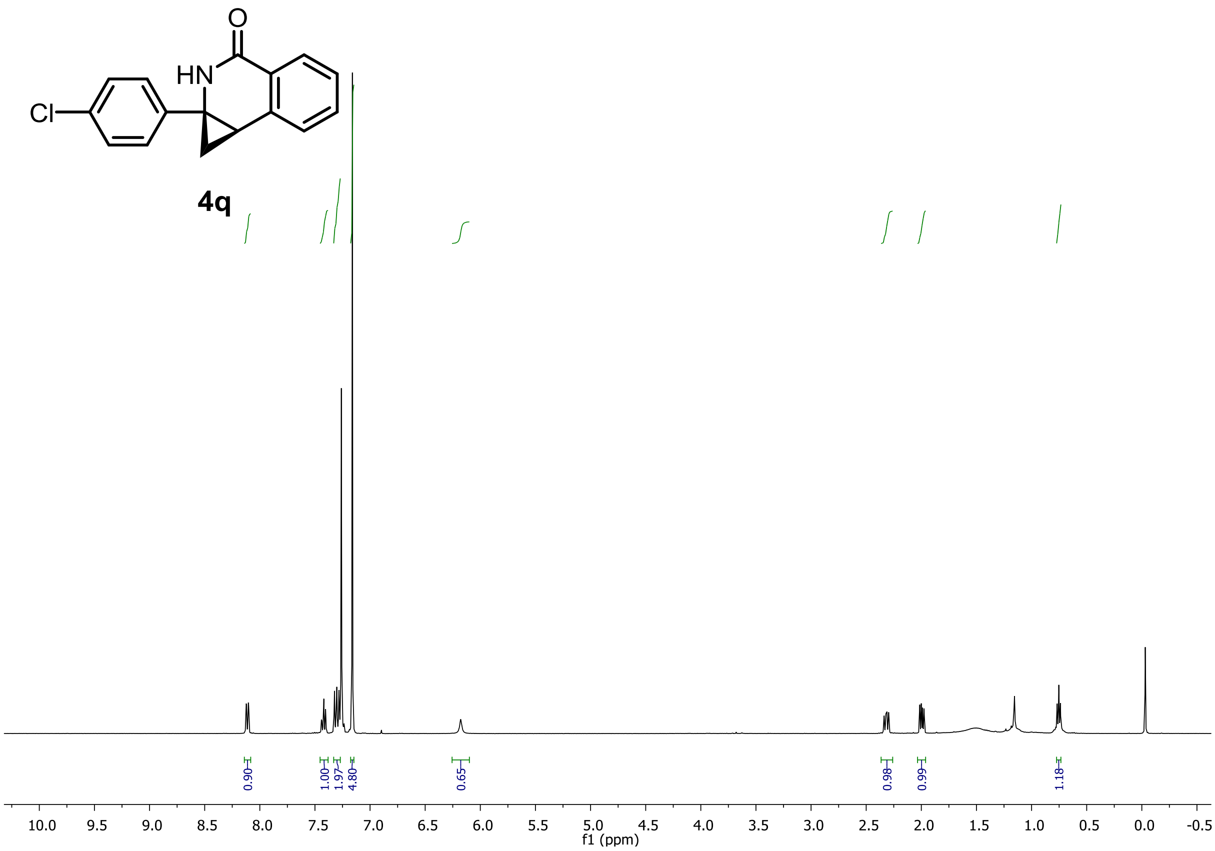
O
HN
Cl
4q
0.90
1.00
1.97
4.80
0.65
0.98
0.99
1.18
10.0 9.5 9.0 8.5 8.0 7.5 7.0 6.5 6.0 5.5 5.0 4.5 4.0 3.5 3.0 2.5 2.0 1.5 1.0 0.5 0.0 -0.5
f1 (ppm)

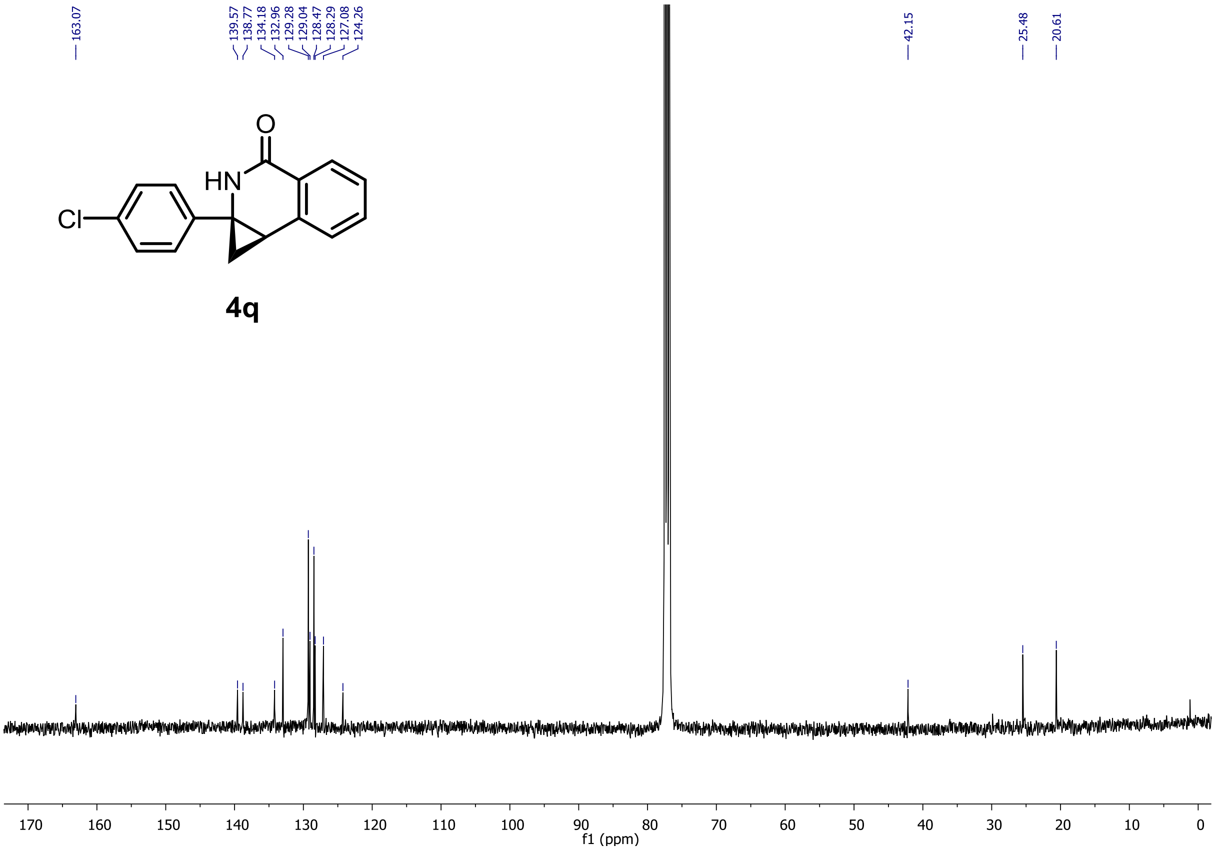
163.07
139.57
138.77
134.18
132.96
129.28
129.04
128.47
128.29
127.08
124.26
42.15
25.48
20.61
O
HN
Cl
4q
170 160 150 140 130 120 110 100 90 80 70 60 50 40 30 20 10 0
f1 (ppm)

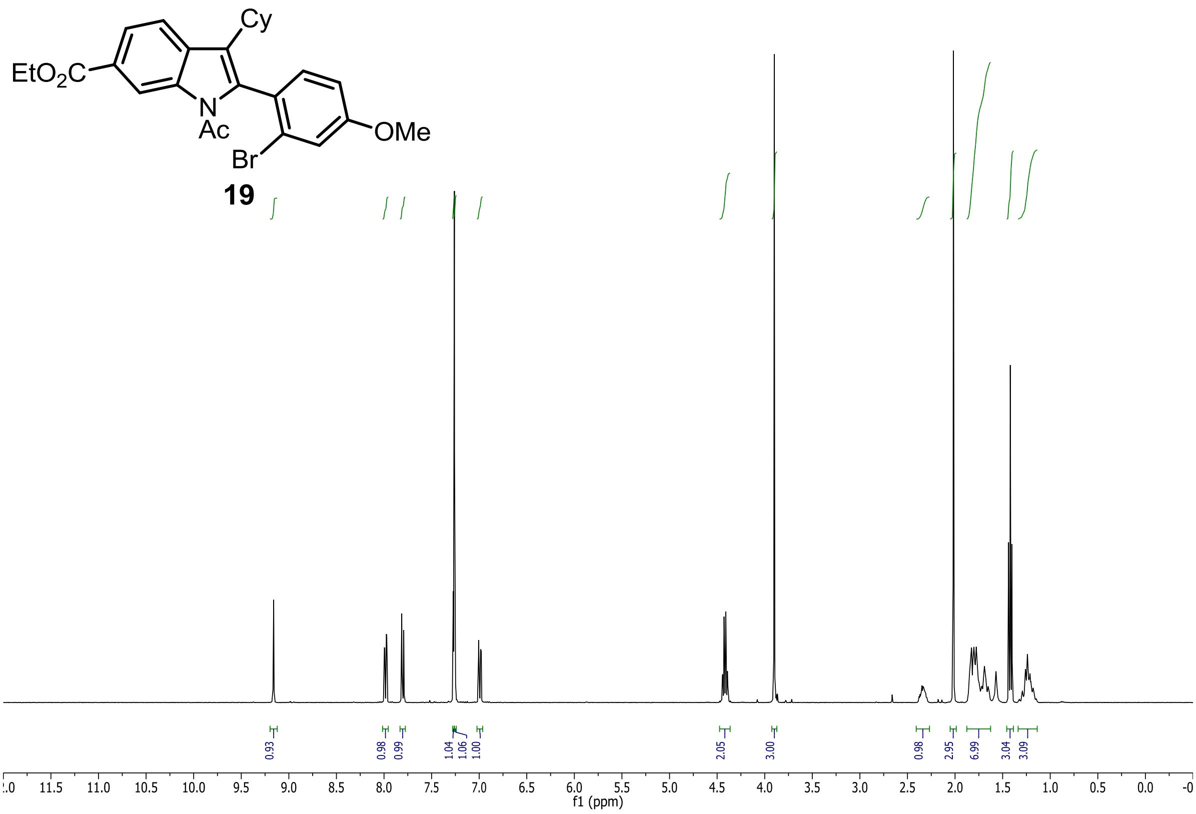

Cy
EtO2C
N
Ac
OMe
Br
19
0.93
0.98
0.99
1.04
1.06
1.00
2.05
3.00
0.98
2.95
6.99
3.04
3.09
f1 (ppm)

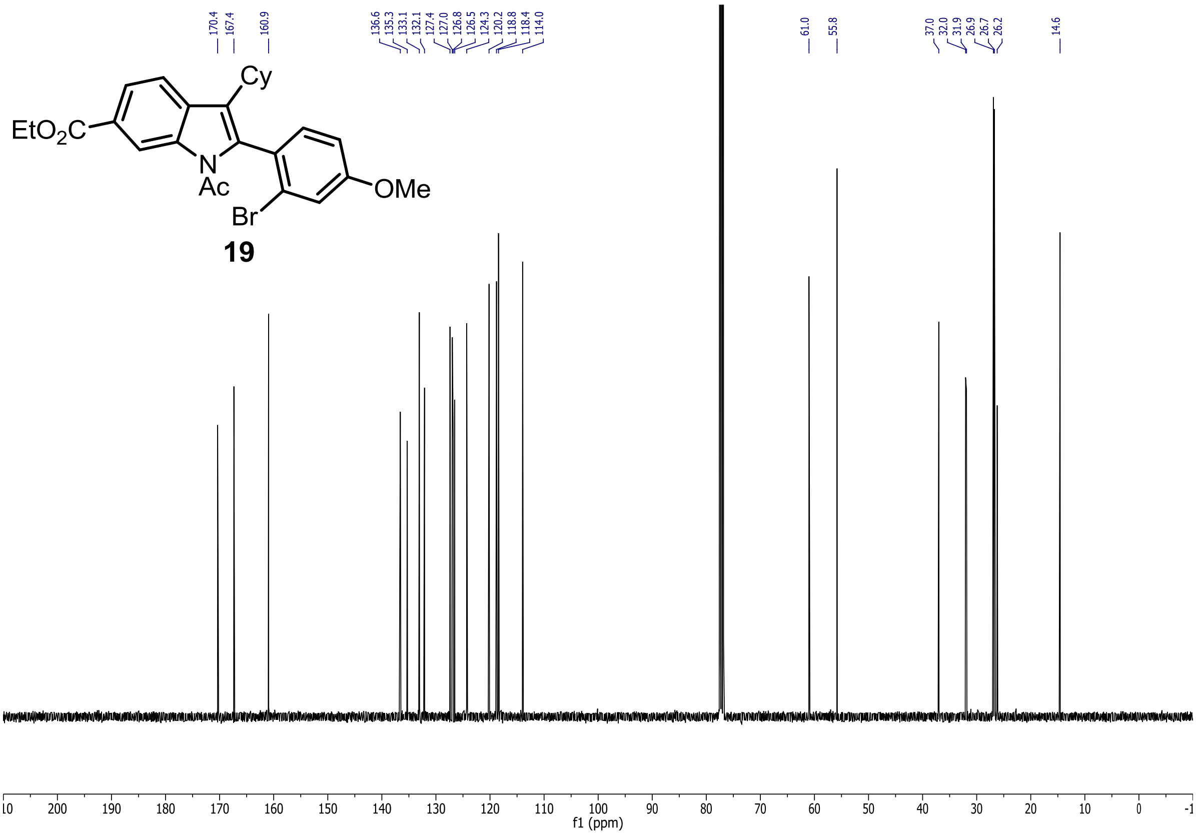

170.4
167.4
160.9
136.6
135.3
133.1
132.1
127.4
127.0
126.8
126.5
124.3
120.2
118.8
118.4
114.0
61.0
55.8
37.0
32.0
31.9
26.9
26.7
26.2
14.6
Cy
EtO2C
N
Ac
OMe
Br
19
f1 (ppm)

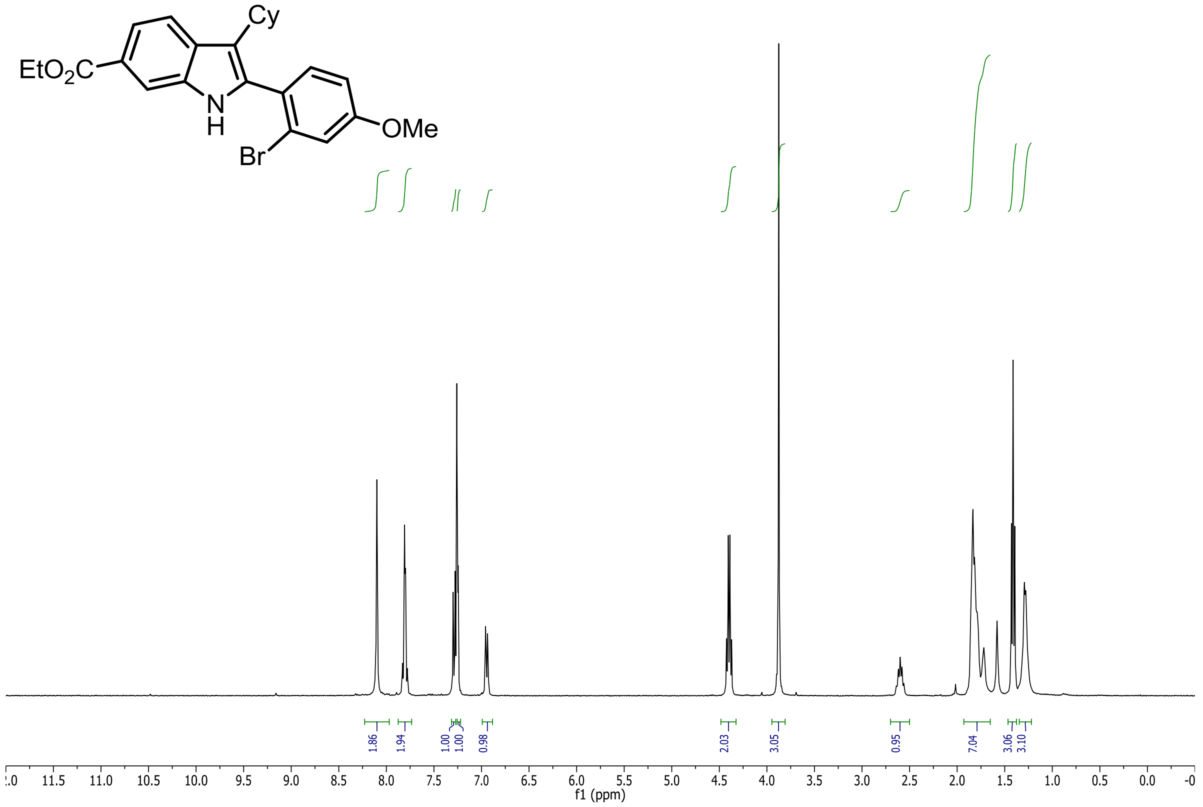
Cy
EtO2C
N
H
OMe
Br
1.86
1.94
1.00
1.00
0.98
2.03
3.05
0.95
7.04
3.06
3.10
f1 (ppm)

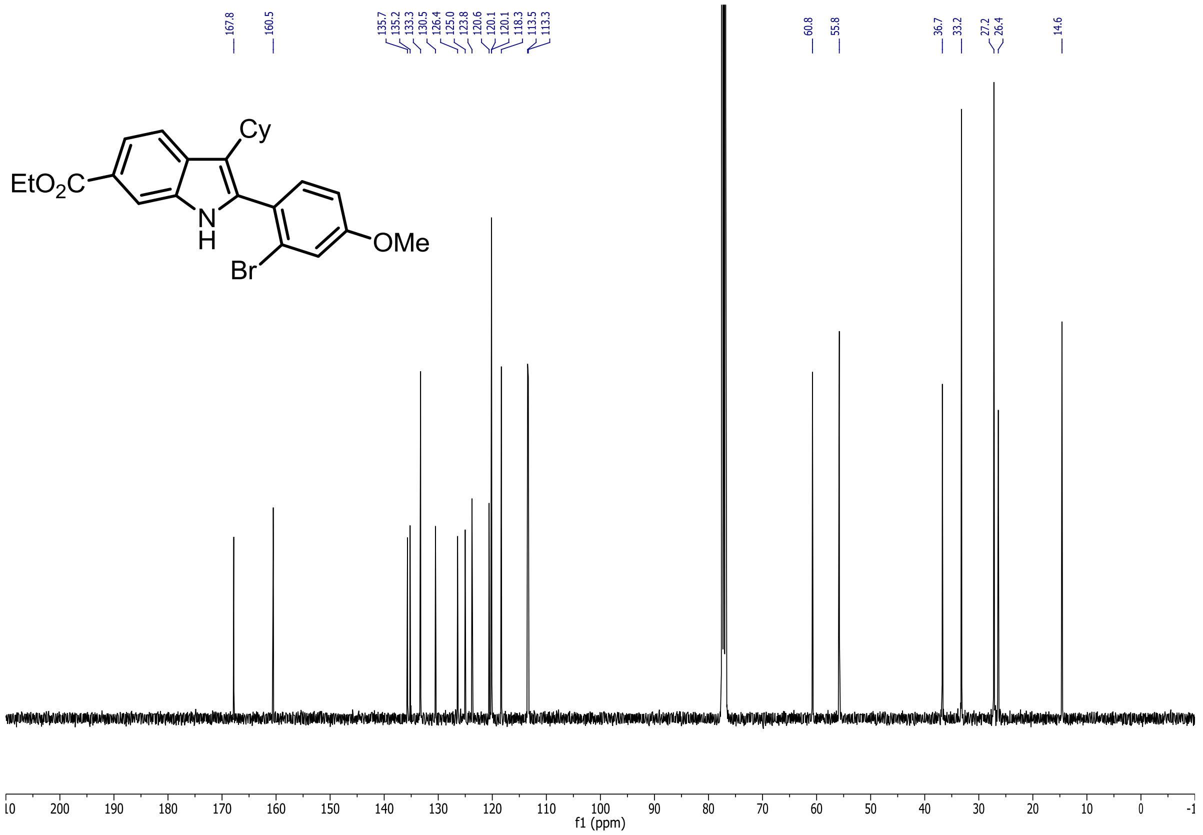
167.8
160.5
135.7
135.2
133.3
130.5
126.4
125.0
123.8
120.6
120.1
120.1
118.3
113.5
113.3
60.8
55.8
36.7
33.2
27.2
26.4
14.6
Cy
EtO2C
N
H
OMe
Br
f1 (ppm)

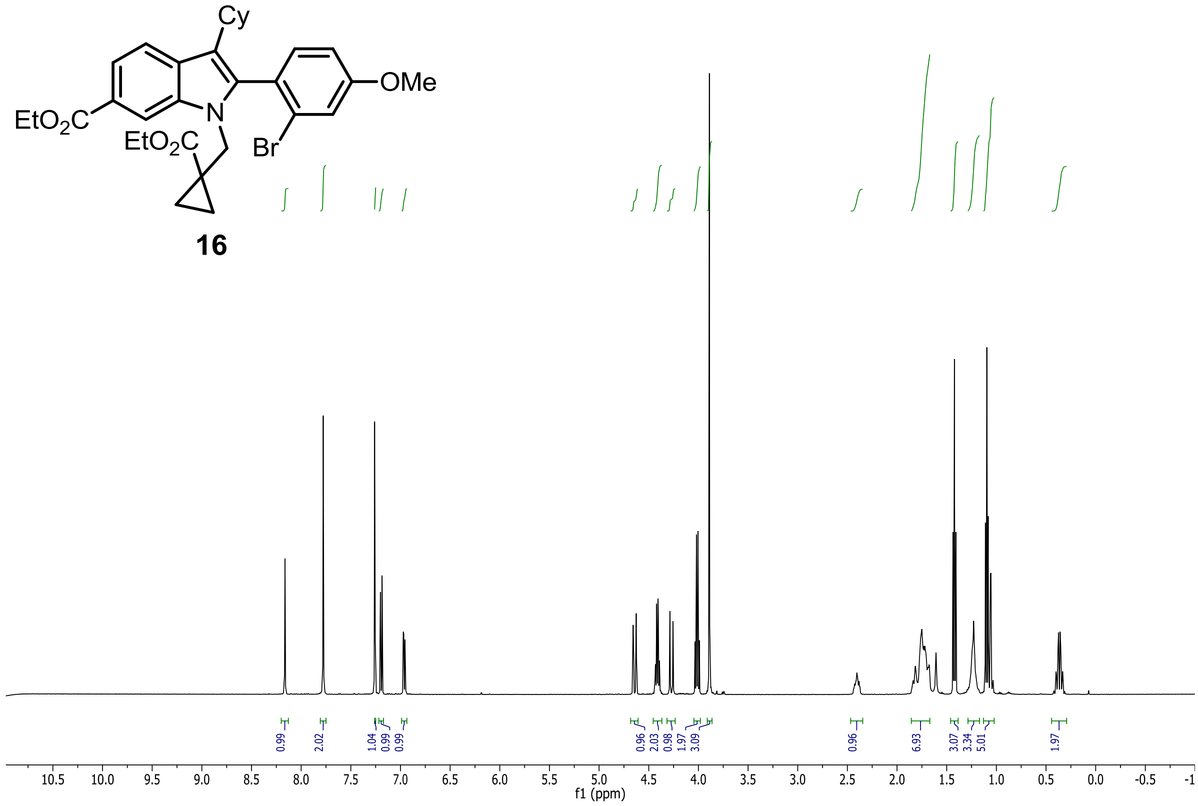
Cy
OMe
EtO2C
N
EtO2C
Br
0.99
2.02
1.04
0.99
0.99
0.96
2.03
0.98
1.97
3.09
0.96
6.93
3.07
3.34
5.01
1.97
f1 (ppm)

16

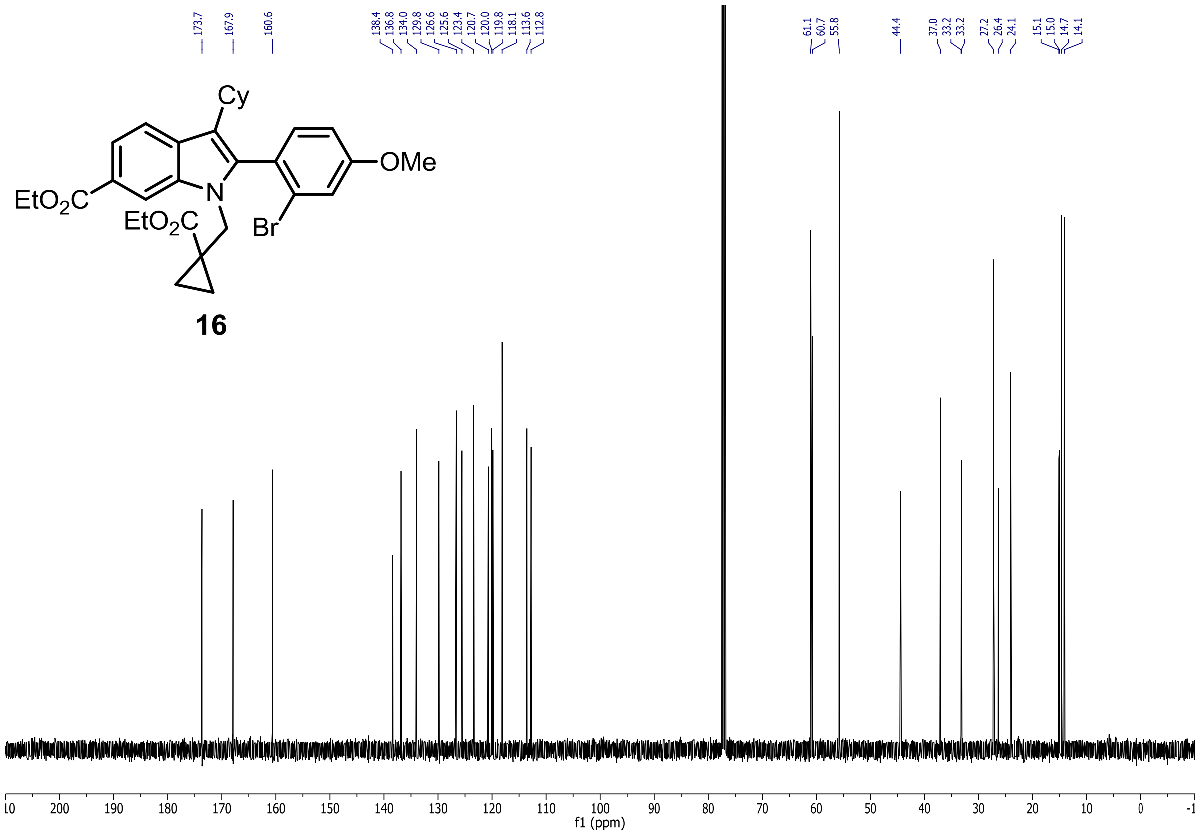
173.7
167.9
160.6
138.4
136.8
134.0
129.8
126.6
125.6
123.4
120.7
120.0
119.8
118.1
113.6
112.8
61.1
60.7
55.8
44.4
37.0
33.2
33.2
27.2
26.4
24.1
15.1
15.0
14.7
14.1
Cy
OMe
EtO2C
N
EtO2C
Br
f1 (ppm)

16

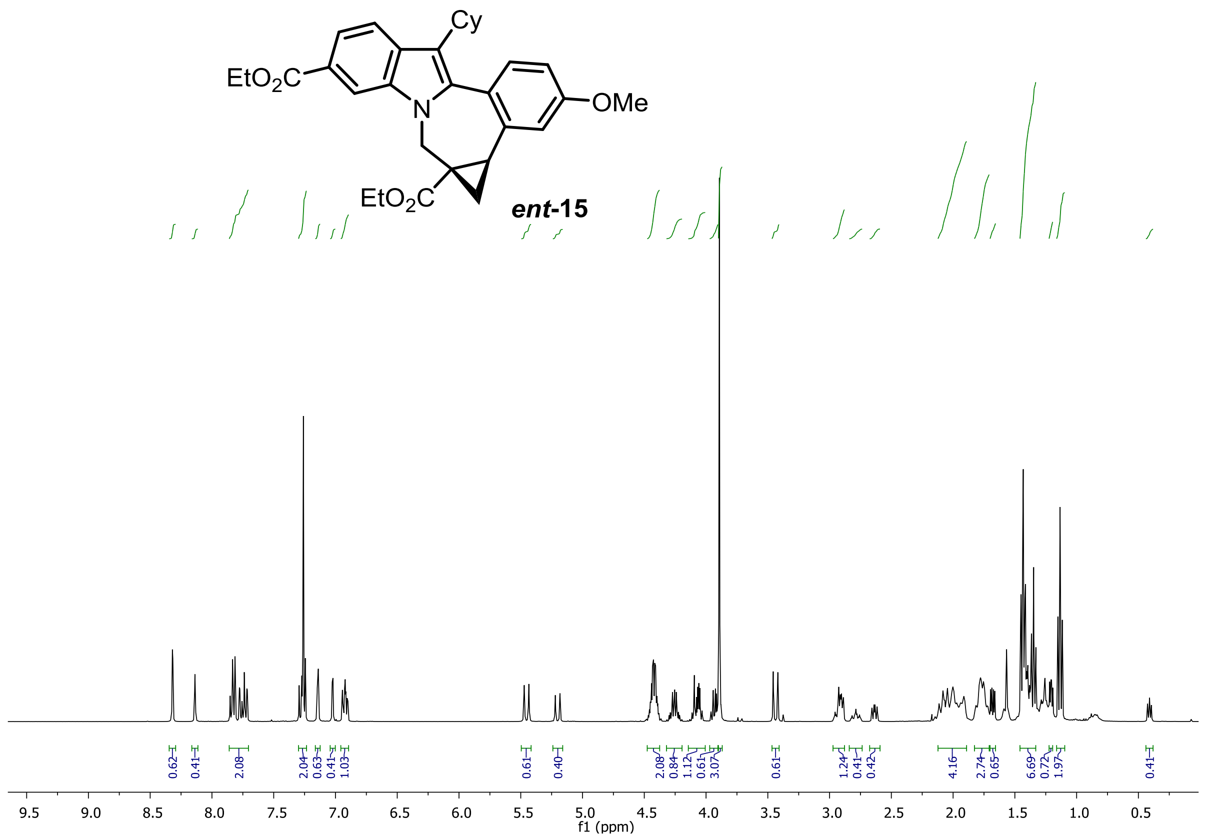
Cy
EtO2C
N
OMe
EtO2C
ent-15
0.62
0.41
2.08
2.04
0.63
0.41
1.03
0.61
0.40
2.08
0.84
1.12
0.61
3.07
0.61
1.24
0.41
0.42
4.16
2.74
0.65
6.69
0.72
1.97
0.41
9.5 9.0 8.5 8.0 7.5 7.0 6.5 6.0 5.5 5.0 4.5 4.0 3.5 3.0 2.5 2.0 1.5 1.0 0.5
f1 (ppm)

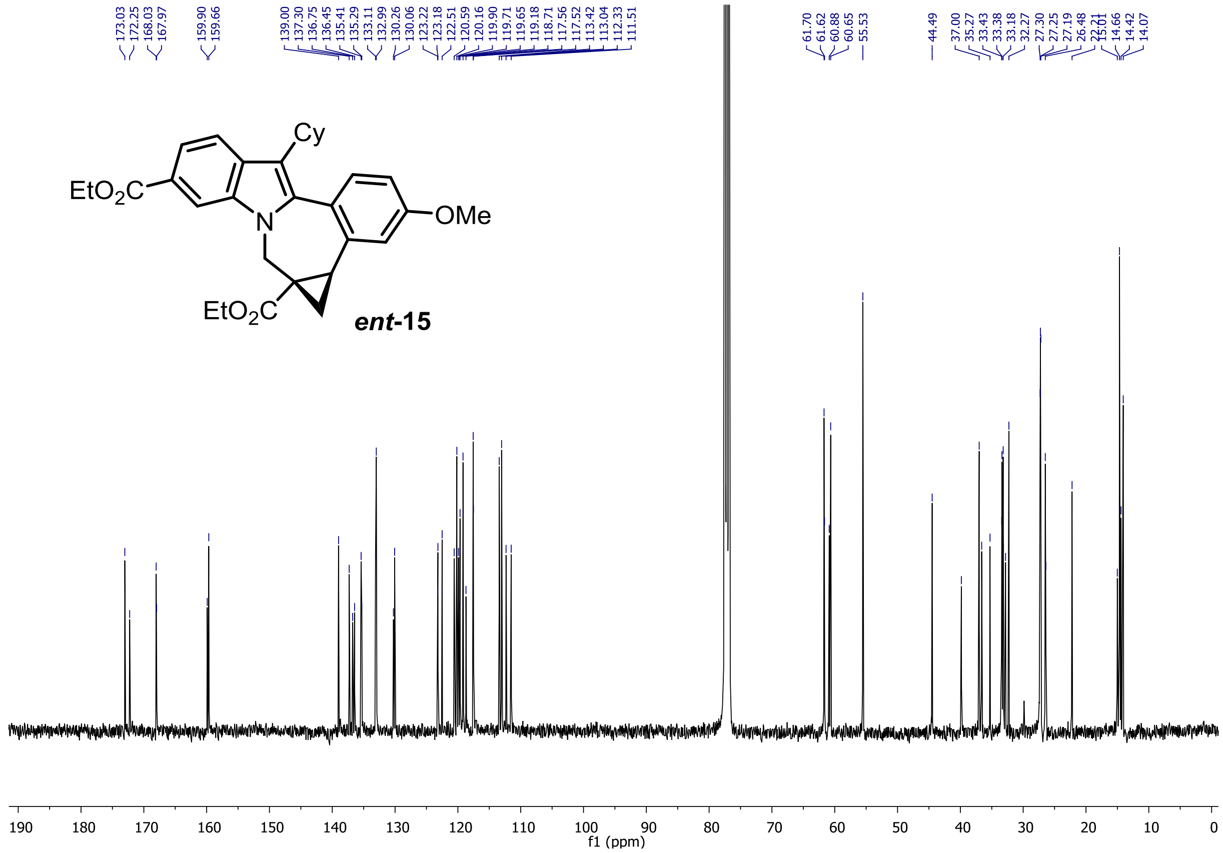
173.03
172.25
168.03
167.97
159.90
159.66
139.00
137.30
136.75
136.45
135.41
135.29
133.11
132.99
130.26
130.06
123.22
123.18
122.51
120.59
120.16
119.90
119.71
119.65
119.18
118.71
117.56
117.52
113.42
113.04
112.33
111.51
61.70
61.62
60.88
60.65
55.53
44.49
37.00
35.27
33.43
33.38
33.18
32.27
27.30
27.25
27.19
26.48
22.21
15.01
14.66
14.42
14.07
Cy
EtO2C
N
OMe
EtO2C
ent-15
190 180 170 160 150 140 130 120 110 100 90 80 70 60 50 40 30 20 10 0
f1 (ppm)

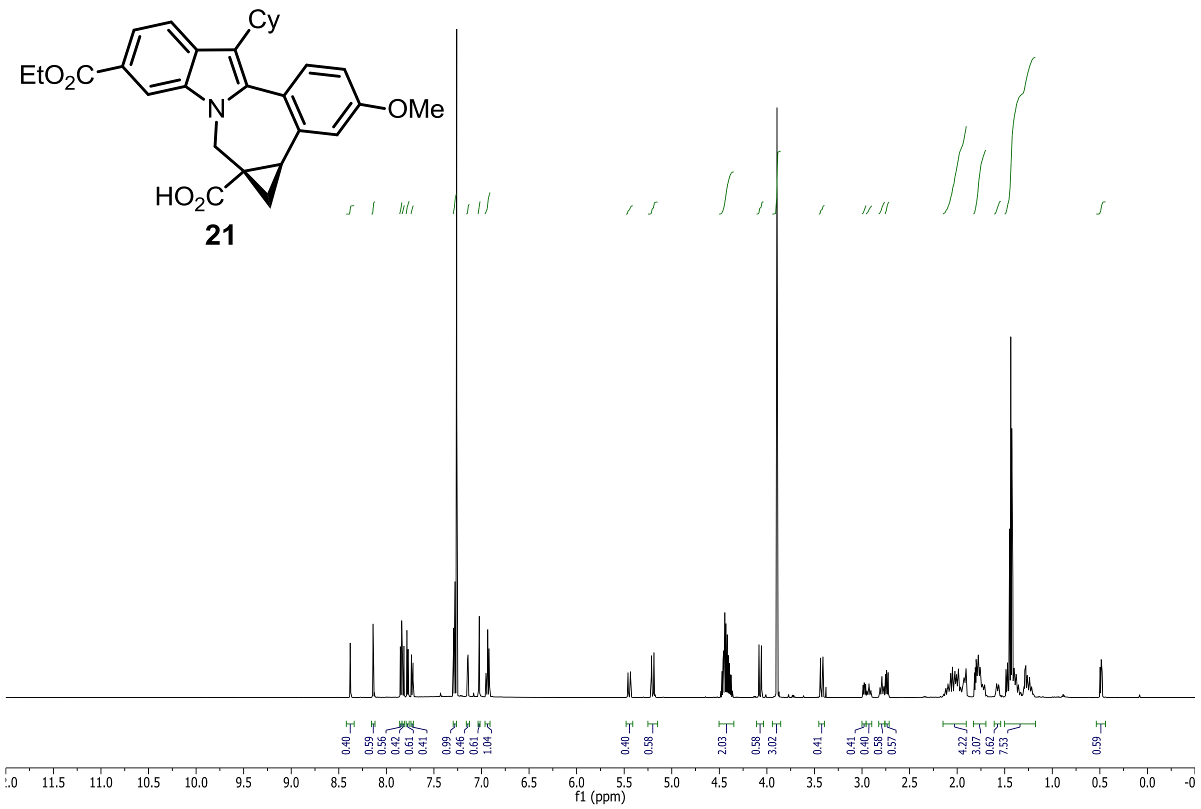

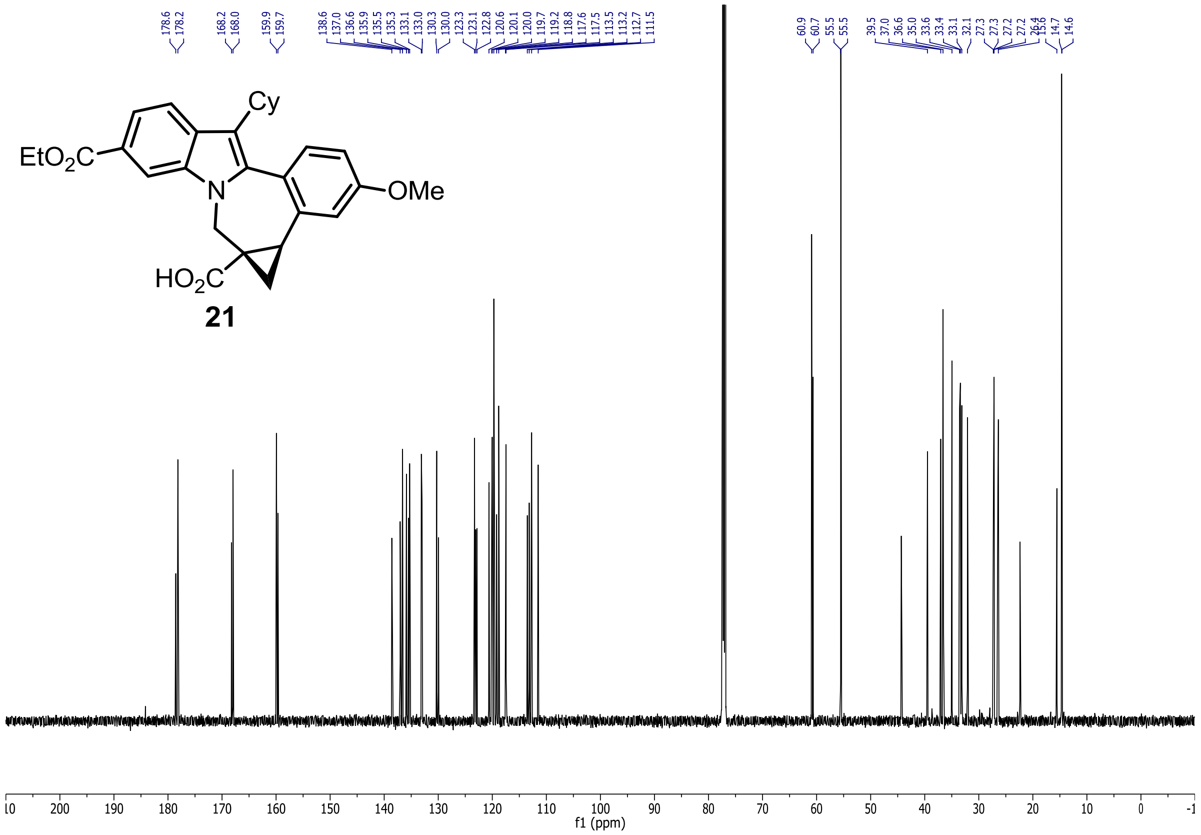

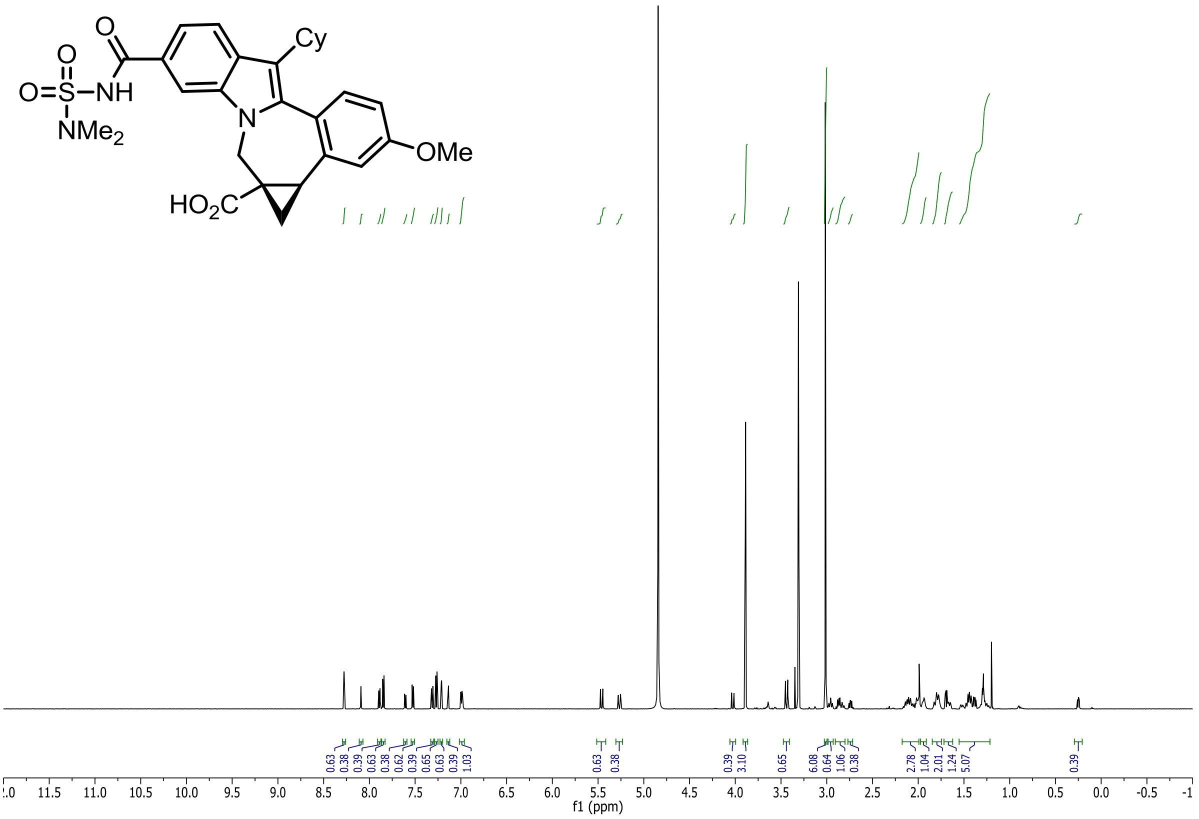
Cy
O
O=S–NH
NMe2
N
OMe
HO2C
f1 (ppm)
11.5 11.0 10.5 10.0 9.5 9.0 8.5 8.0 7.5 7.0 6.5 6.0 5.5 5.0 4.5 4.0 3.5 3.0 2.5 2.0 1.5 1.0 0.5 0.0 -0.5
